# Supplementary material for: FADD is recruited to activated STING oligomers to initiate caspase-mediated NF-κB activation in Drosophila melanogaster
Source: EMBO J. 2026 Mar 28;45(9):2965–90. doi: 10.1038/s44318-026-00761-9 (PMC13144350; doi:10.1038/s44318-026-00761-9)
Supplement: Supplementary file 1 — Appendix [file 44318_2026_761_MOESM1_ESM.pdf]

Appendix for:

FADD is recruited to activated STING oligomers to initiate caspase-mediated NF-κB activation in *Drosophila melanogaster*

Table of Content

Appendix Figure S1: Editing of S2 cell KO pools 2

Appendix Figure S2: AlphaFold model of dFADD in complex with dSTING 4

Appendix Figure S3: AlphaFold model of dFADD in complex with DREDD 6

Appendix Figure S4: AlphaFold model of dFADD in complex with IMD 8

Appendix Figure S5: Luciferase assay of dSTING and Imd mutants 10

A

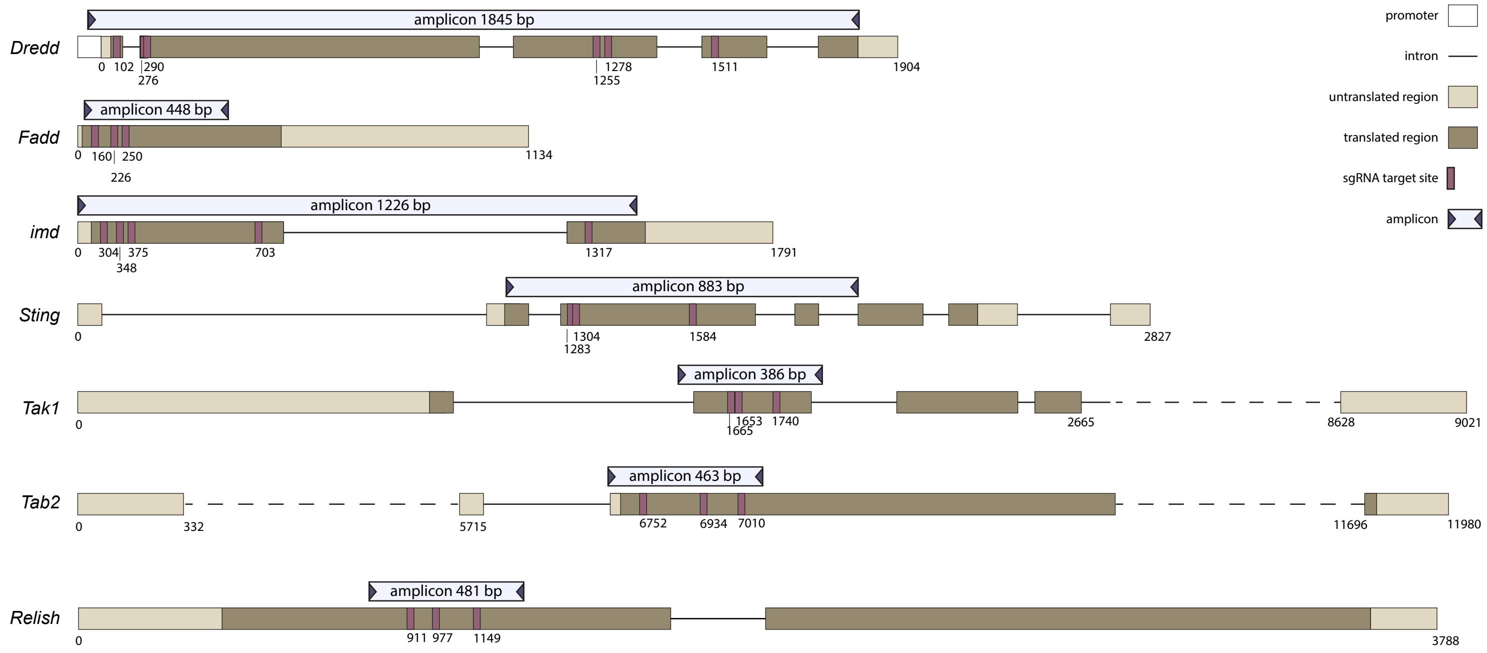

B

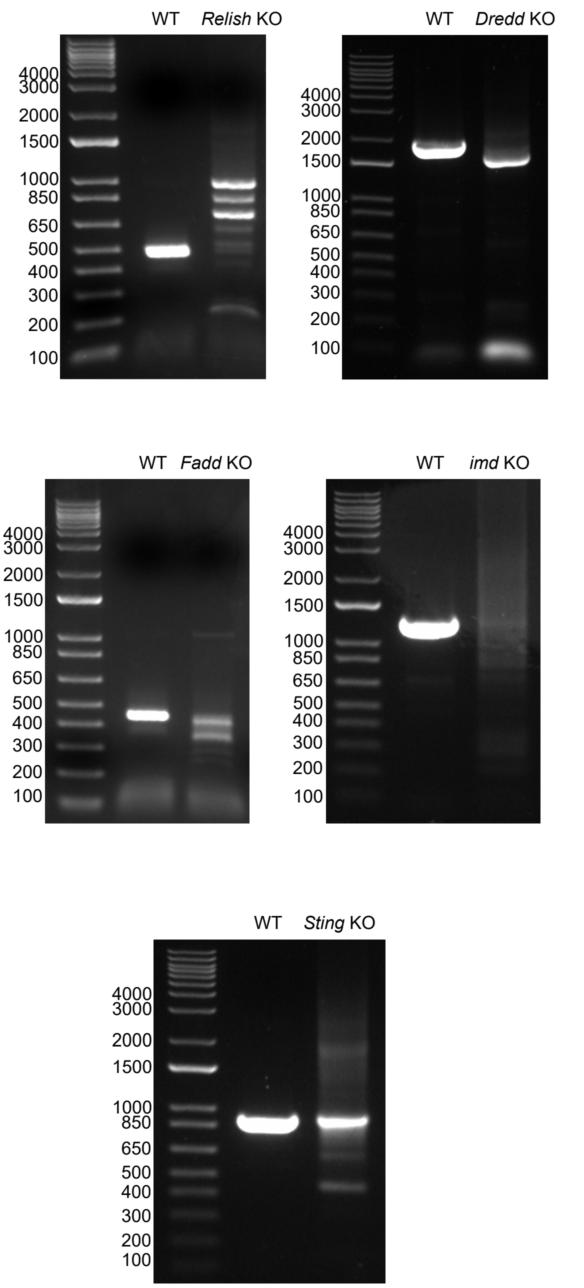

C

Reference position	Consensus pos...	Consensus resi...	Other residues	IUPAC	Status
1565	42	C	Reference: C. Reads: A (1), C (4)	M	conflict
1582	59	T	Reference: T. Reads: G (1), T (4)	K	conflict
1588	65	C	Reference: C. Reads: A (1), C (4)	M	conflict
1626	103	T	Reference: T. Reads: C (1), T (4)	Y	conflict
1630	107	T	Reference: T. Reads: A (1), T (4)	W	conflict
1649	126	G	Reference: G. Reads: G (2), T (1)	K	conflict
1742^1743	220	C	Reference: -. Reads: - (2), C (2)	C	conflict
1743	221	A	Reference: A. Reads: A (2), G (2)	R	conflict
1745	223	C	Reference: C. Reads: C (2), T (2)	Y	conflict
1746	224	A	Reference: A. Reads: A (2), G (2)	R	conflict
1748^1749	227	G	Reference: -. Reads: - (2), G (2)	G	conflict
1750	229	A	Reference: G. Reads: A (2), G (2)	R	conflict
1751^1752	231	A	Reference: -. Reads: - (2), A (2)	A	conflict
1751^1752	232	A	Reference: -. Reads: - (2), A (2)	A	conflict
1752^1753	234	T	Reference: -. Reads: - (2), T (2)	T	conflict
1752^1753	235	G	Reference: -. Reads: - (2), G (2)	G	conflict
1752^1753	236	T	Reference: -. Reads: - (2), T (2)	T	conflict
1752^1753	237	A	Reference: -. Reads: - (2), A (2)	A	conflict

D

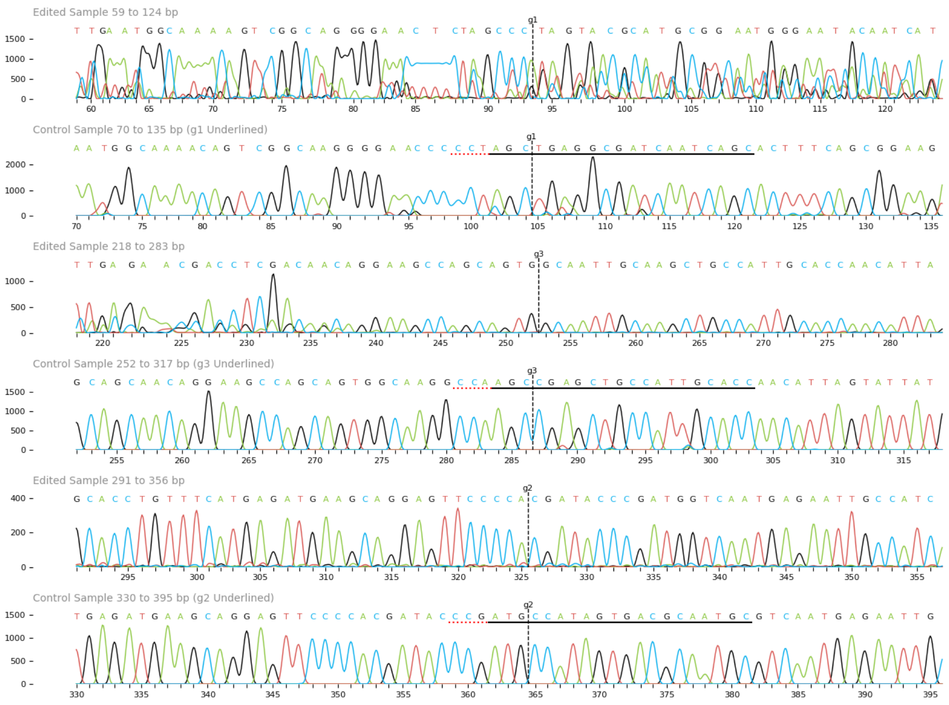

Appendix Figure S1. Editing of S2 cell KO pools. A, Figure shows an overview of genes edited in S2 cells by CRISPR/Cas9 including sgRNA target sites and expected amplicon sizes **B,** Genomic DNA from WT and edited polyclonal S2 cells was purified and the indicated regions in **A** were amplified by PCR and analysed by gel electrophoresis. **C-D,** The PCR products from *Tab2* and *Tak1* KO cells were sanger sequenced either directly (Tab2, panel d) or after cloning (TAK1, panel c).

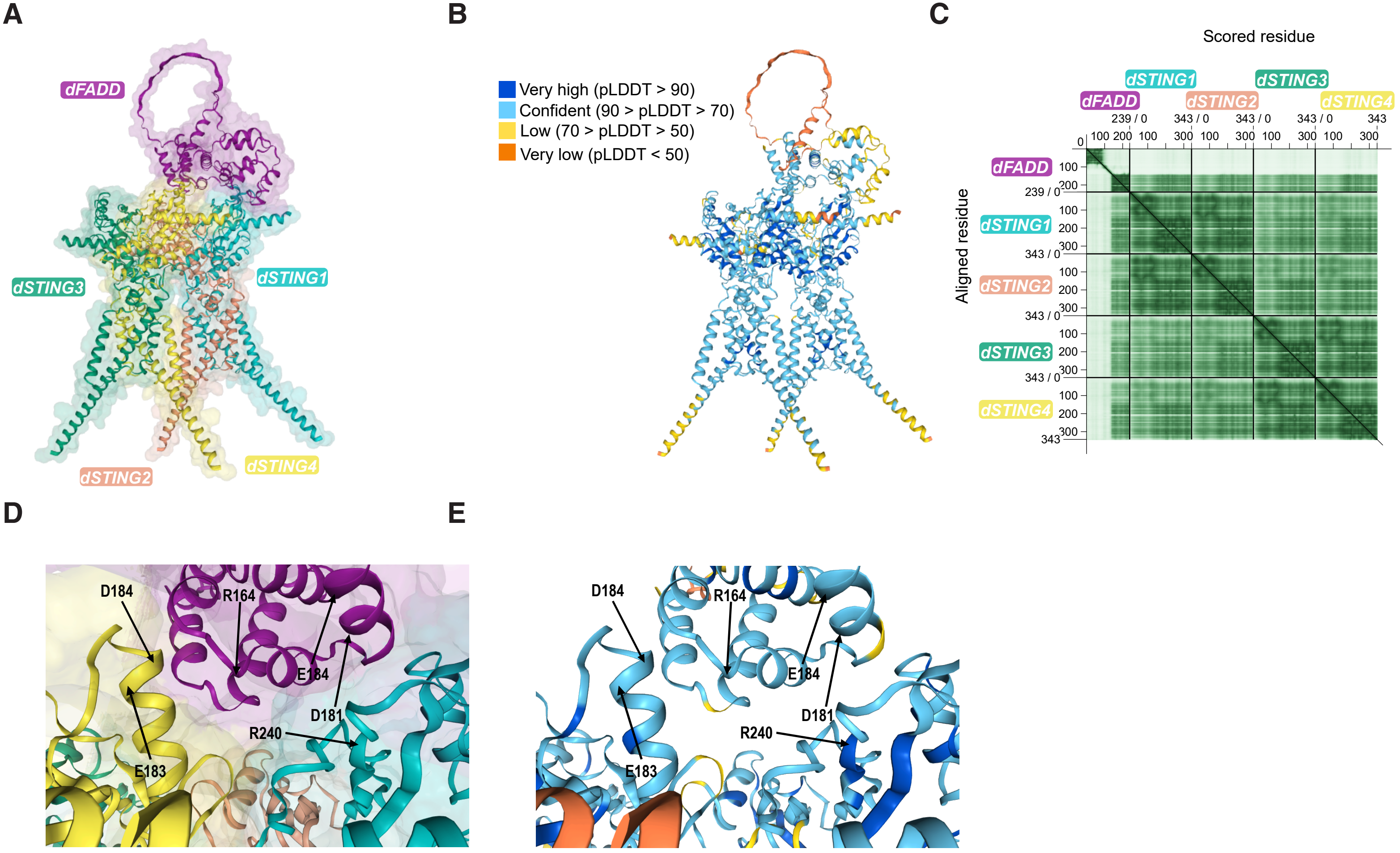
A
B
C
D
E
dFADD
dSTING1
dSTING2
dSTING3
dSTING4
Very high (pLDDT > 90)
Confident (90 > pLDDT > 70)
Low (70 > pLDDT > 50)
Very low (pLDDT < 50)
Scored residue
Aligned residue
239 / 0
343 / 0
343
D184
R164
E184
D181
R240
E183

Appendix Figure S2. AlphaFold model of dFADD in complex with dSTING. A, Predicted structure of the complex between dFADD and a dSTING tetramer colored according to chains. **B,** Predicted structure of the complex between dFADD and a dSTING tetramer colored according to pLDDT score. The complex is predicted with high confidence (scores >70) except for some less ordered regions. **C,** The PAE plot for the complex. Protein chains are indicated by different colors. **D-E,** Zoom of the predicted interaction site between dFADD and dSTING colored by **D,** chains and **E,** pLDDT score. Showing that the interaction site is predicted with high confidence (pLDDT scores > 70). Mutated residues are indicated.

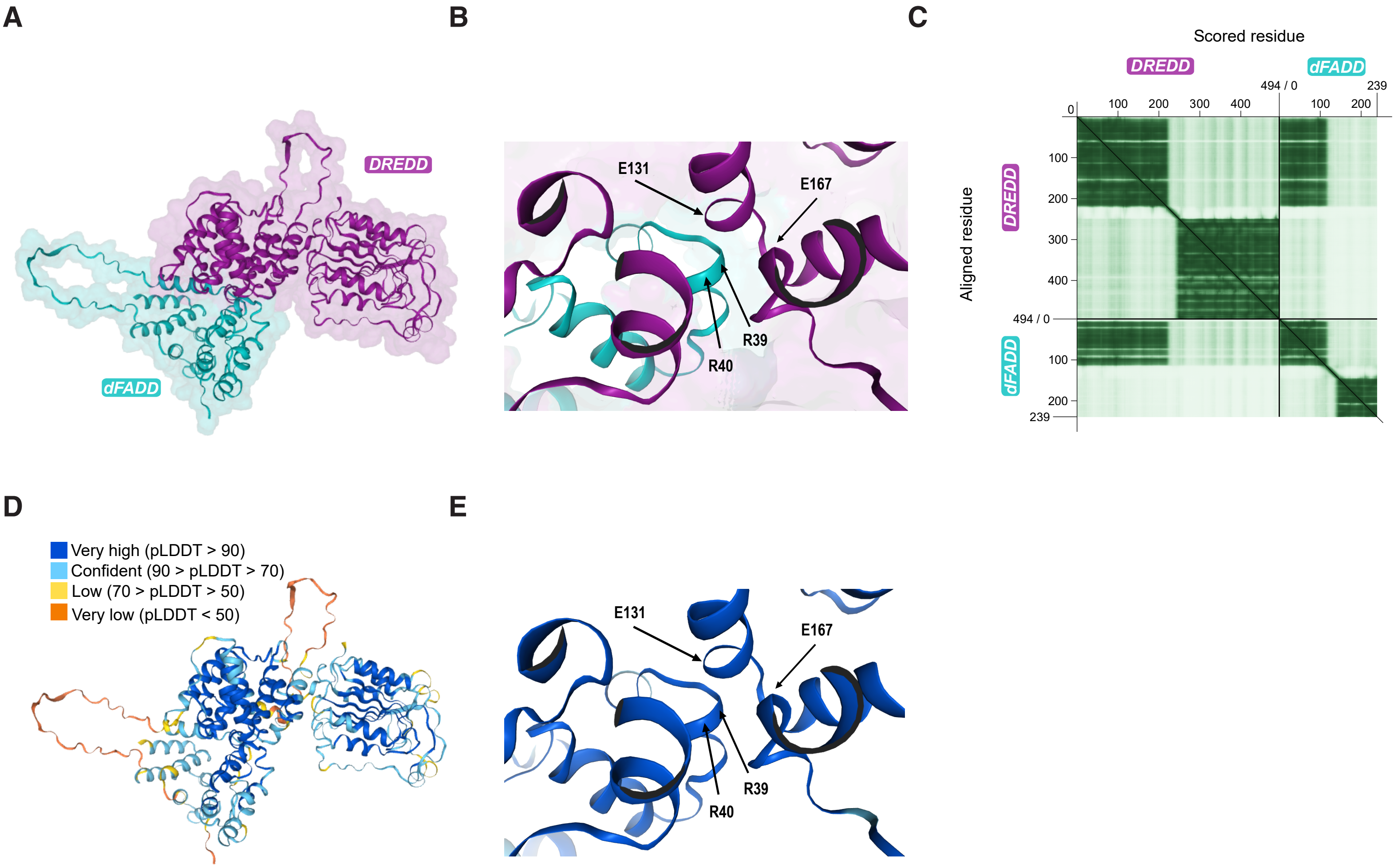
A
DREDD
dFADD
B
E131
E167
R39
R40
C
Scored residue
DREDD
dFADD
494 / 0
239
0
100
200
300
400
100
200
Aligned residue
DREDD
dFADD
494 / 0
239
D
Very high (pLDDT > 90)
Confident (90 > pLDDT > 70)
Low (70 > pLDDT > 50)
Very low (pLDDT < 50)
E
E131
E167
R39
R40

Appendix Figure S3. AlphaFold model of dFADD in complex with DREDD. A, Predicted structure of the complex between dFADD and DREDD colored according to chains. **B,** Zoom of the predicted interaction site between dFADD and DREDD colored by chains. Mutated residues are indicated. **C,** The PAE plot for the complex. Protein chains are indicated by different colors. **D,** Predicted structure of the complex between dFADD and a DREDD colored according to pLDDT score. The complex is predicted with high confidence (scores >70) except for some less ordered regions. **E,** Zoom of the predicted interaction site between dFADD and DREDD colored by pLDDT score. Showing that the interaction site is predicted with very high confidence (pLDDT scores > 90). Mutated residues are indicated.

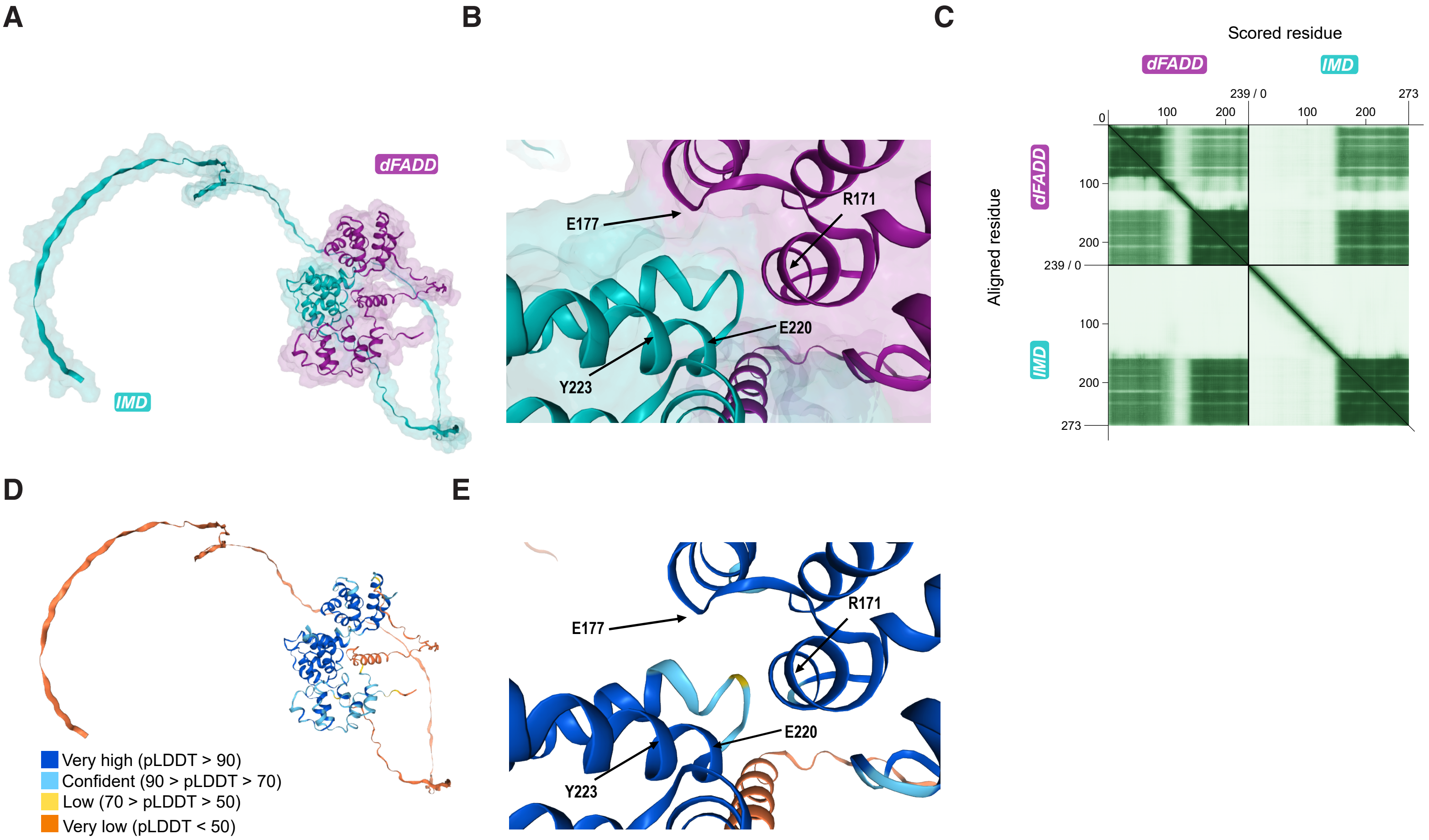

A
dFADD
IMD
B
E177
R171
E220
Y223
C
Scored residue
dFADD
IMD
239 / 0
273
0
100
200
100
200
Aligned residue
dFADD
100
200
239 / 0
100
IMD
200
273
D
Very high (pLDDT > 90)
Confident (90 > pLDDT > 70)
Low (70 > pLDDT > 50)
Very low (pLDDT < 50)
E
E177
R171
E220
Y223

Appendix Figure S4. AlphaFold model of dFADD in complex with IMD. A, Predicted structure of the complex between dFADD and IMD colored according to chains. **B,** Zoom of the predicted interaction site between dFADD and IMD colored by chains. Mutated residues are indicated. **C,** The PAE plot for the complex. Protein chains are indicated by different colors. **D,** Predicted structure of the complex between dFADD and a IMD colored according to pLDDT score. The complex is predicted with high confidence (scores >70) except for the N-terminal part of IMD and the loop between the N- and C-terminal of dFADD which is predicted with very low confidence (pLDDT score < 70 **E,** Zoom of the predicted interaction site between dFADD and IMD colored by pLDDT score. Showing that the interaction site is predicted with very high confidence (pLDDT scores > 90). Mutated residues are indicated.

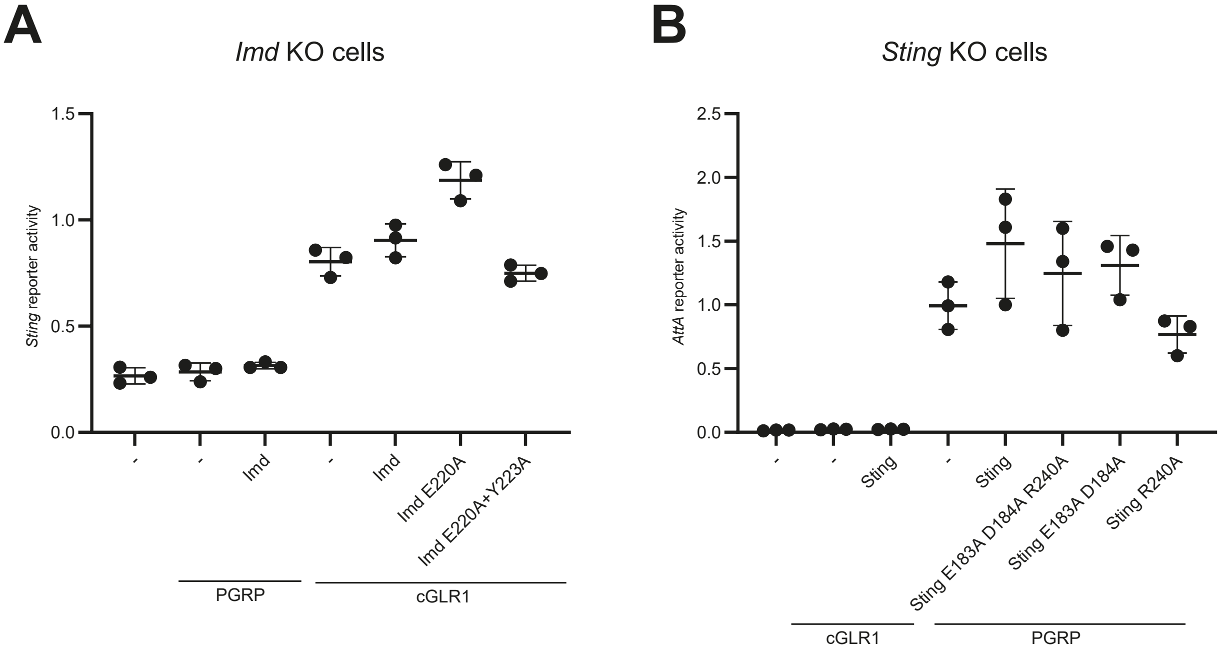
A
Imd KO cells
Sting reporter activity
1.5
1.0
0.5
0.0
-
-
Imd
-
Imd
Imd E220A
Imd E220A+Y223A
PGRP
cGLR1
B
Sting KO cells
AttA reporter activity
2.5
2.0
1.5
1.0
0.5
0.0
-
-
Sting
-
Sting
Sting E183A D184A R240A
Sting E183A D184A
Sting R240A
cGLR1
PGRP

Appendix Figure S5. Luciferase assay of dSTING and Imd mutants. A, mutating E220A alone or both E220A and Y223A in IMD does not impact dSTING signalling (*Sting* reporter activity) in *Imd* KO cells. **B,** Likewise, the dSTING E183A/D184A/R240 mutant, or the respective single mutants, have no or little effect on IMD signalling (*AttA* reporter activity) in *Sting* KO cells. **A-B,** data from one independent experiment performed in biological triplicates, are shown with mean and bars ndicating standard deviation (n = 3).
