# Supplementary figures and images for: FADD is recruited to activated STING oligomers to initiate caspase-mediated NF-κB activation in Drosophila melanogaster (part 1 of 2)
Source: EMBO J. 2026 Mar 28;45(9):2965–90. doi: 10.1038/s44318-026-00761-9 (PMC13144350; doi:10.1038/s44318-026-00761-9)

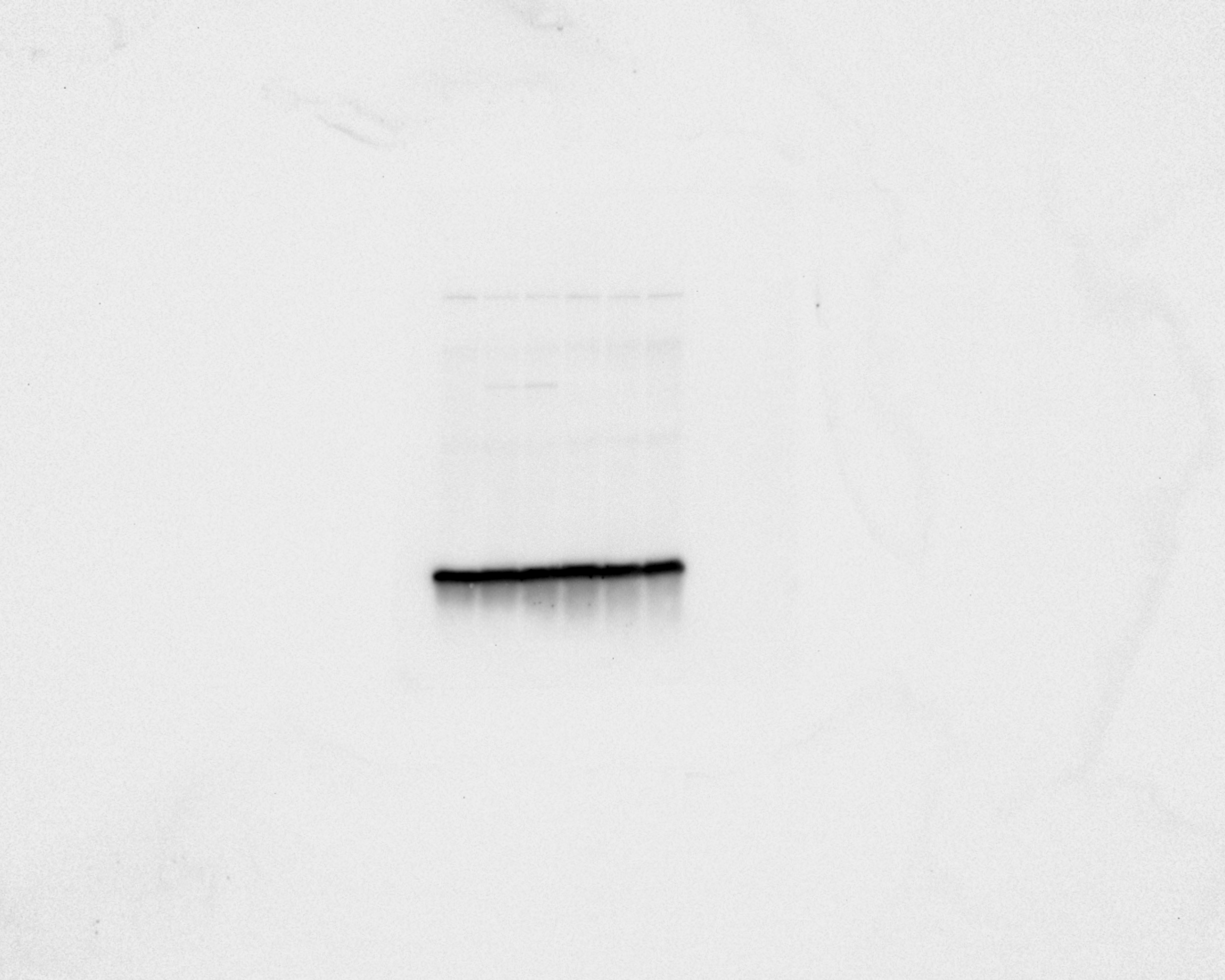

Supplement: Supplementary file 3 — Source data Fig. 1 [file 44318_2026_761_MOESM3_ESM.zip › Figure 1/1A/Western blot histone H3.tif]

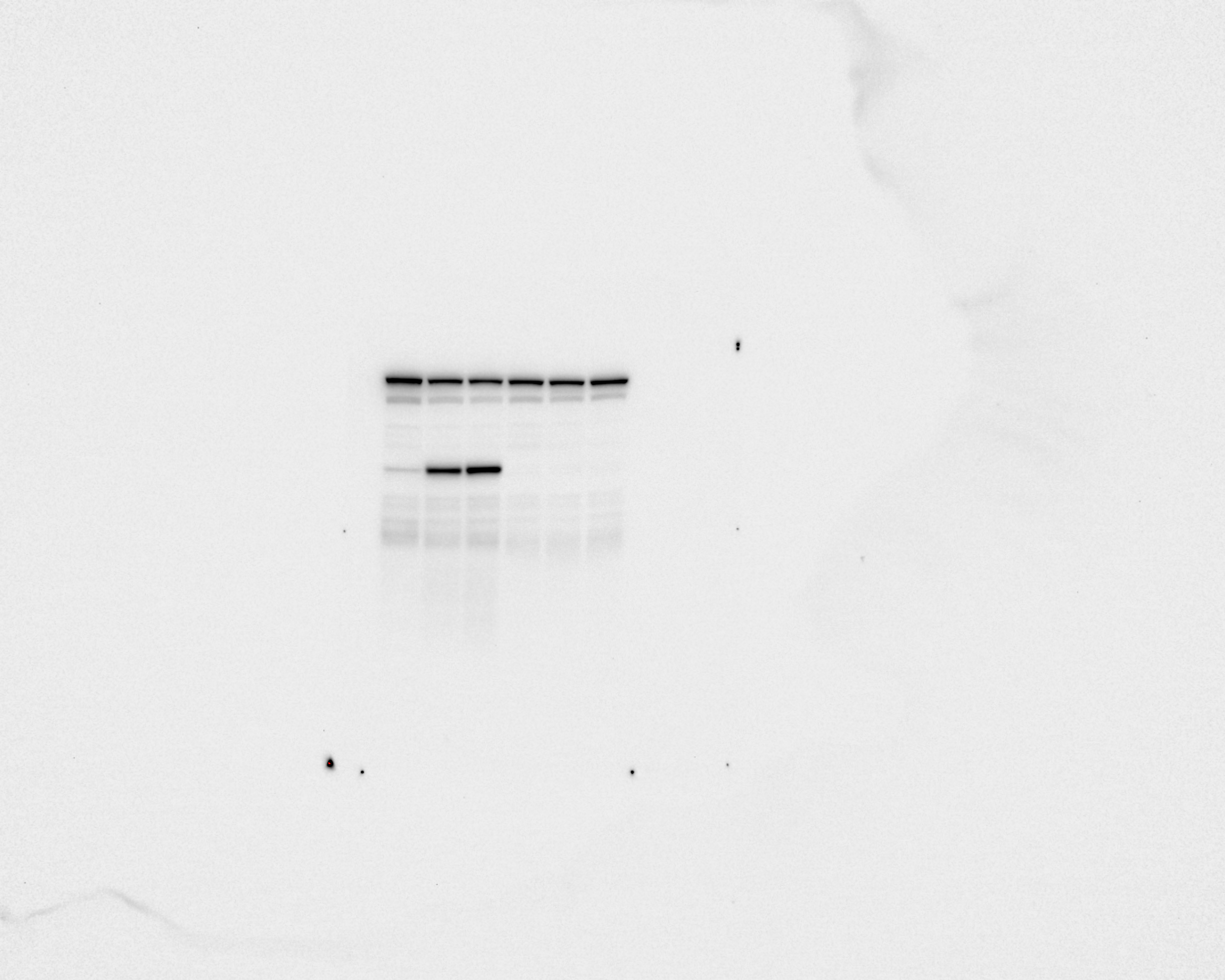

Supplement: Supplementary file 3 — Source data Fig. 1 [file 44318_2026_761_MOESM3_ESM.zip › Figure 1/1A/Western blot V5.tif]

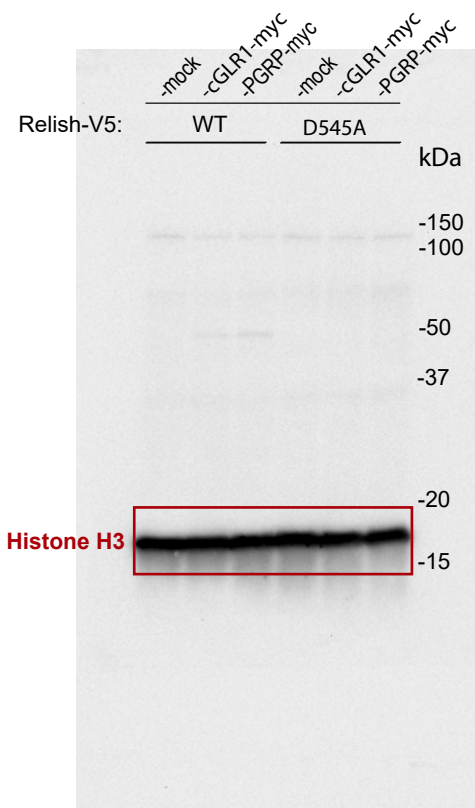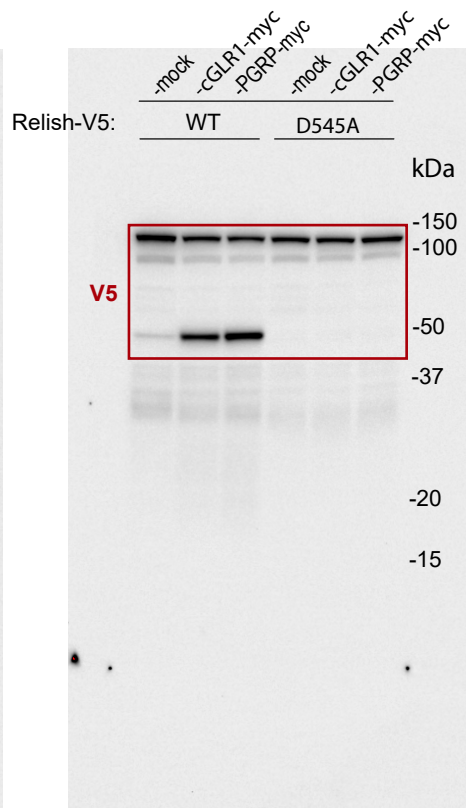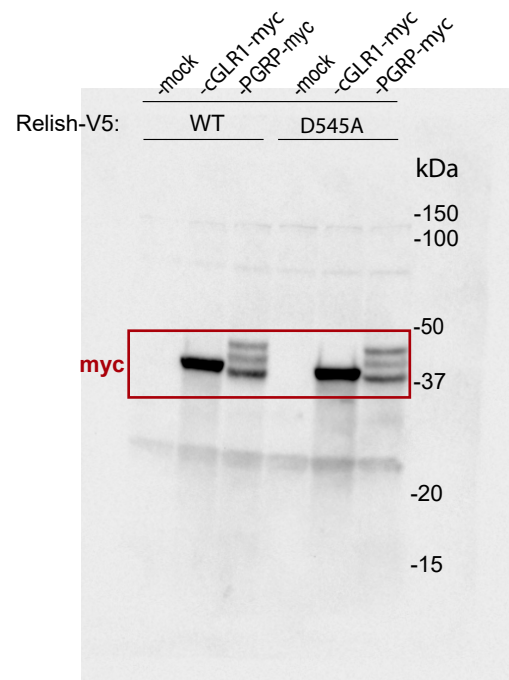

Supplement: Supplementary file 3 — Source data Fig. 1 [file 44318_2026_761_MOESM3_ESM.zip › Figure 1/1A/Annotation.pdf]

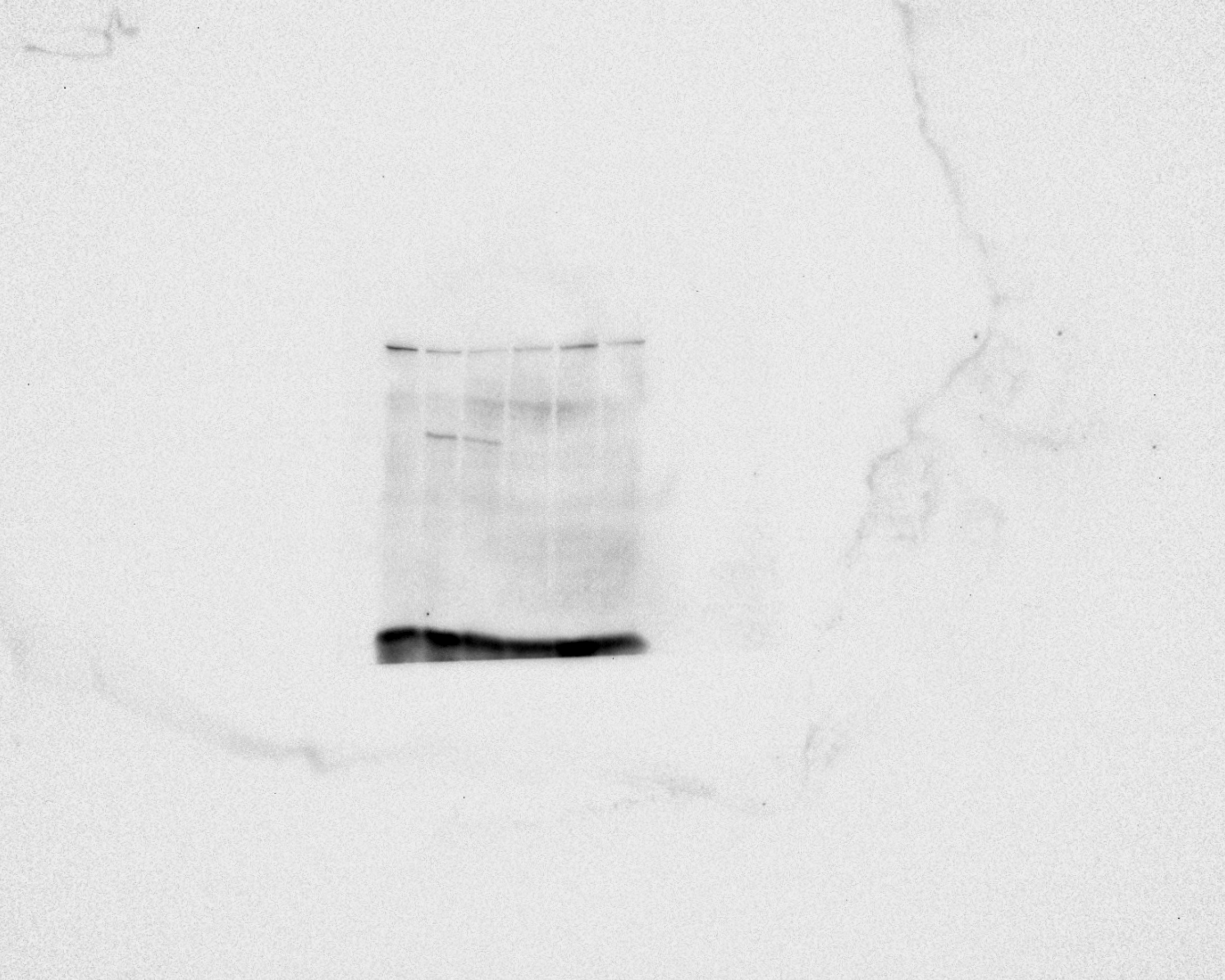

Supplement: Supplementary file 3 — Source data Fig. 1 [file 44318_2026_761_MOESM3_ESM.zip › Figure 1/1A/Repeat B/western blot histone H3.tif]

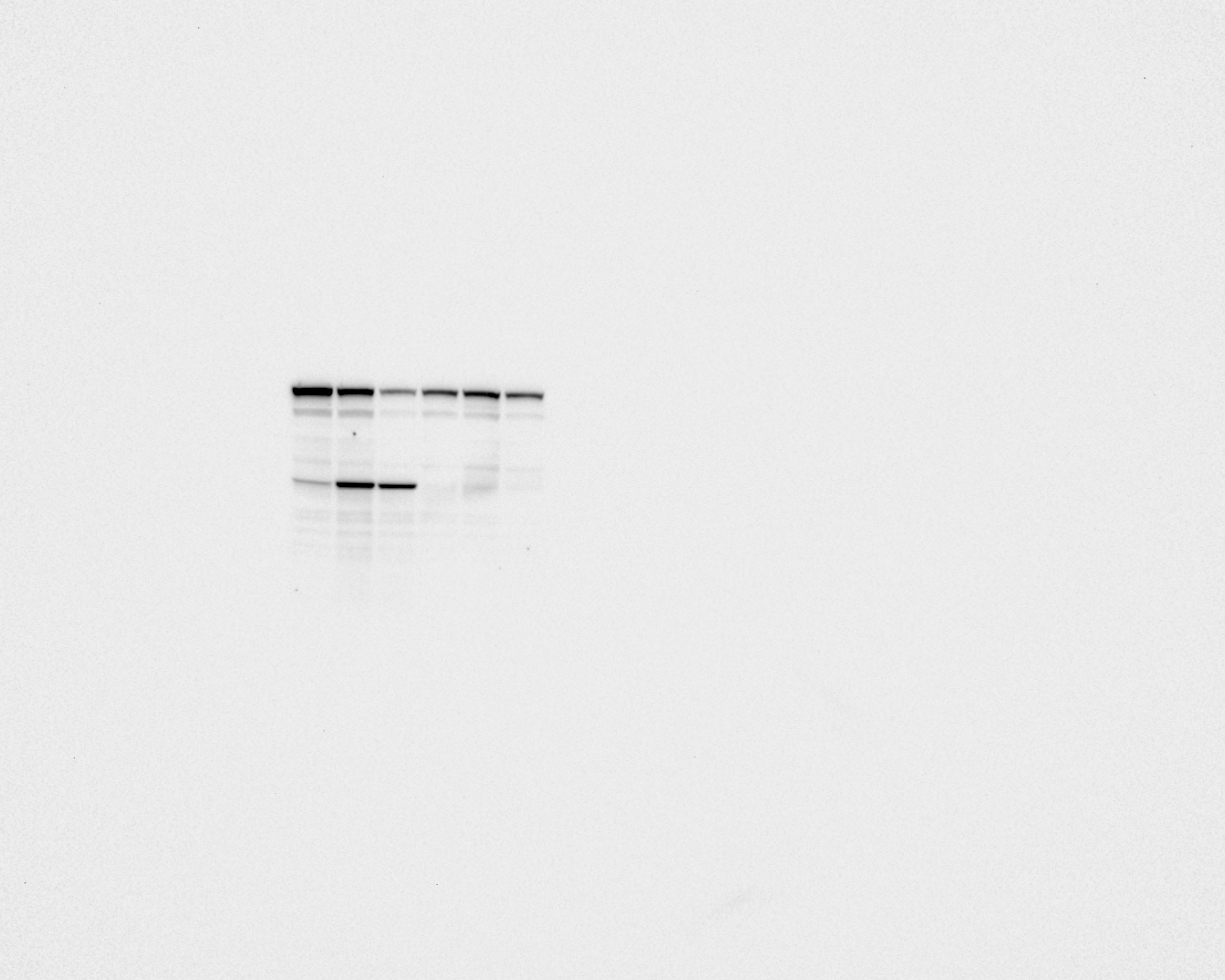

Supplement: Supplementary file 3 — Source data Fig. 1 [file 44318_2026_761_MOESM3_ESM.zip › Figure 1/1A/Repeat B/western blot V5.tif]

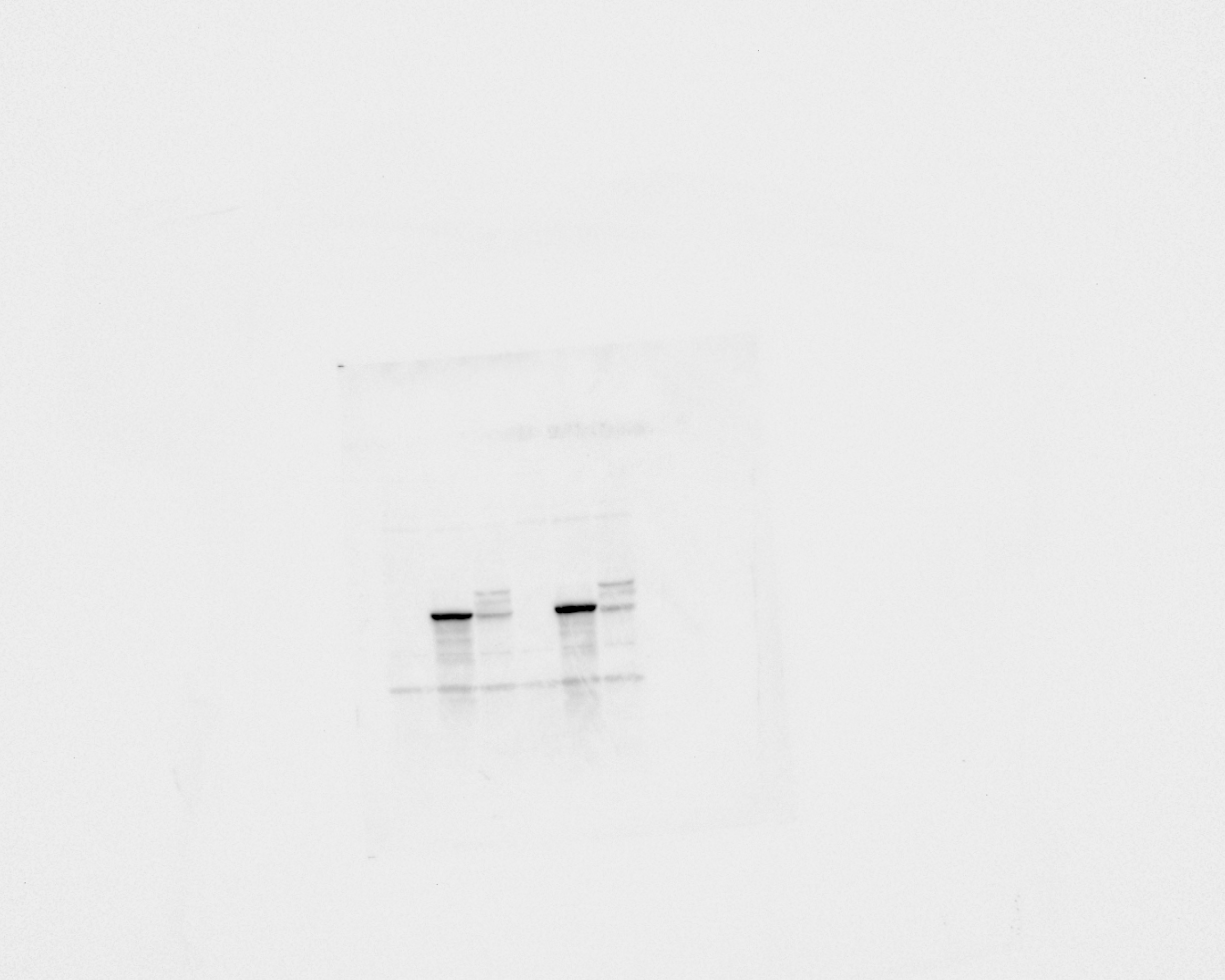

Supplement: Supplementary file 3 — Source data Fig. 1 [file 44318_2026_761_MOESM3_ESM.zip › Figure 1/1A/Repeat B/western blot myc.tif]

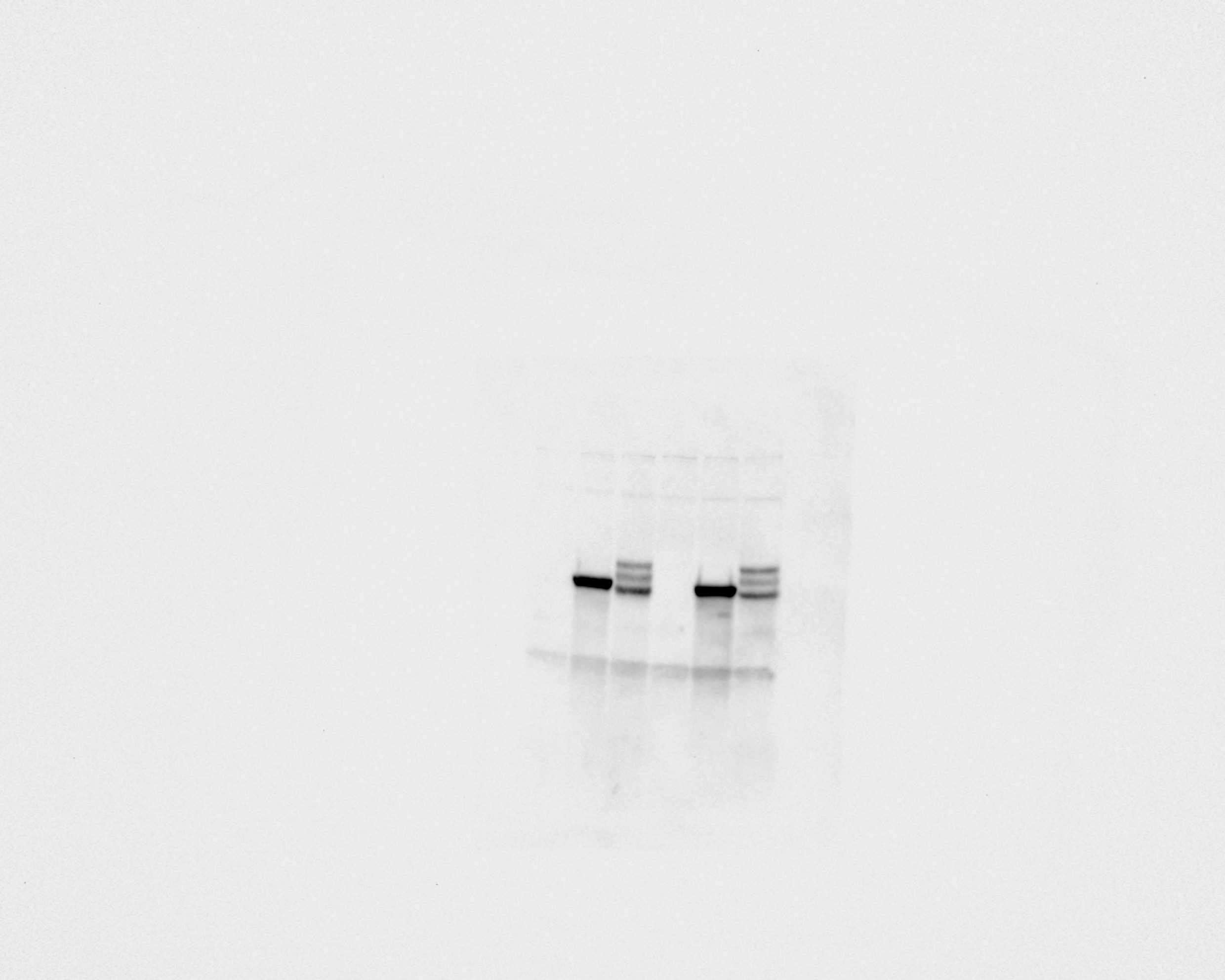

Supplement: Supplementary file 3 — Source data Fig. 1 [file 44318_2026_761_MOESM3_ESM.zip › Figure 1/1A/Western blot myc.tif]

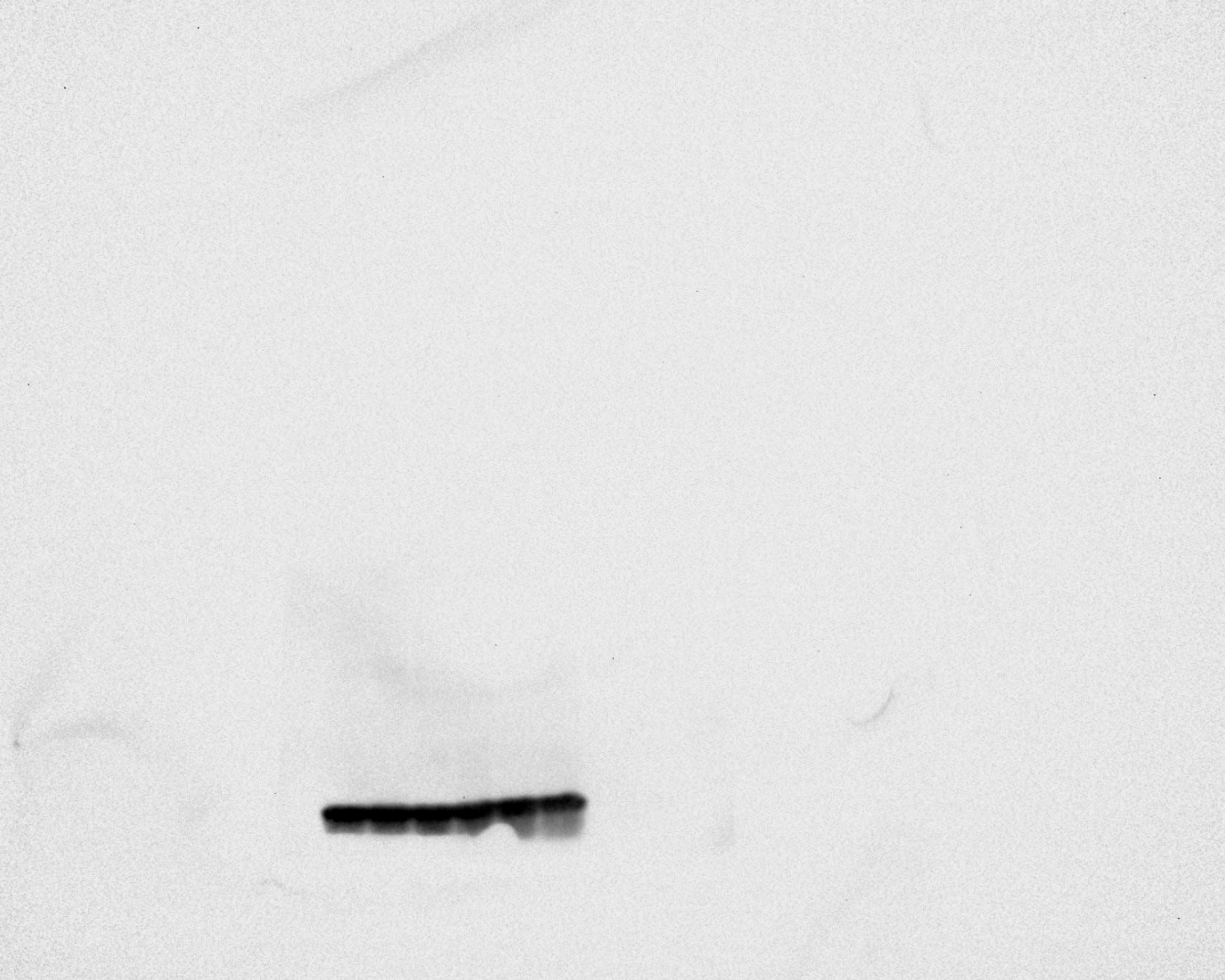

Supplement: Supplementary file 3 — Source data Fig. 1 [file 44318_2026_761_MOESM3_ESM.zip › Figure 1/1A/Repeat A/western blot histone H3.tif]

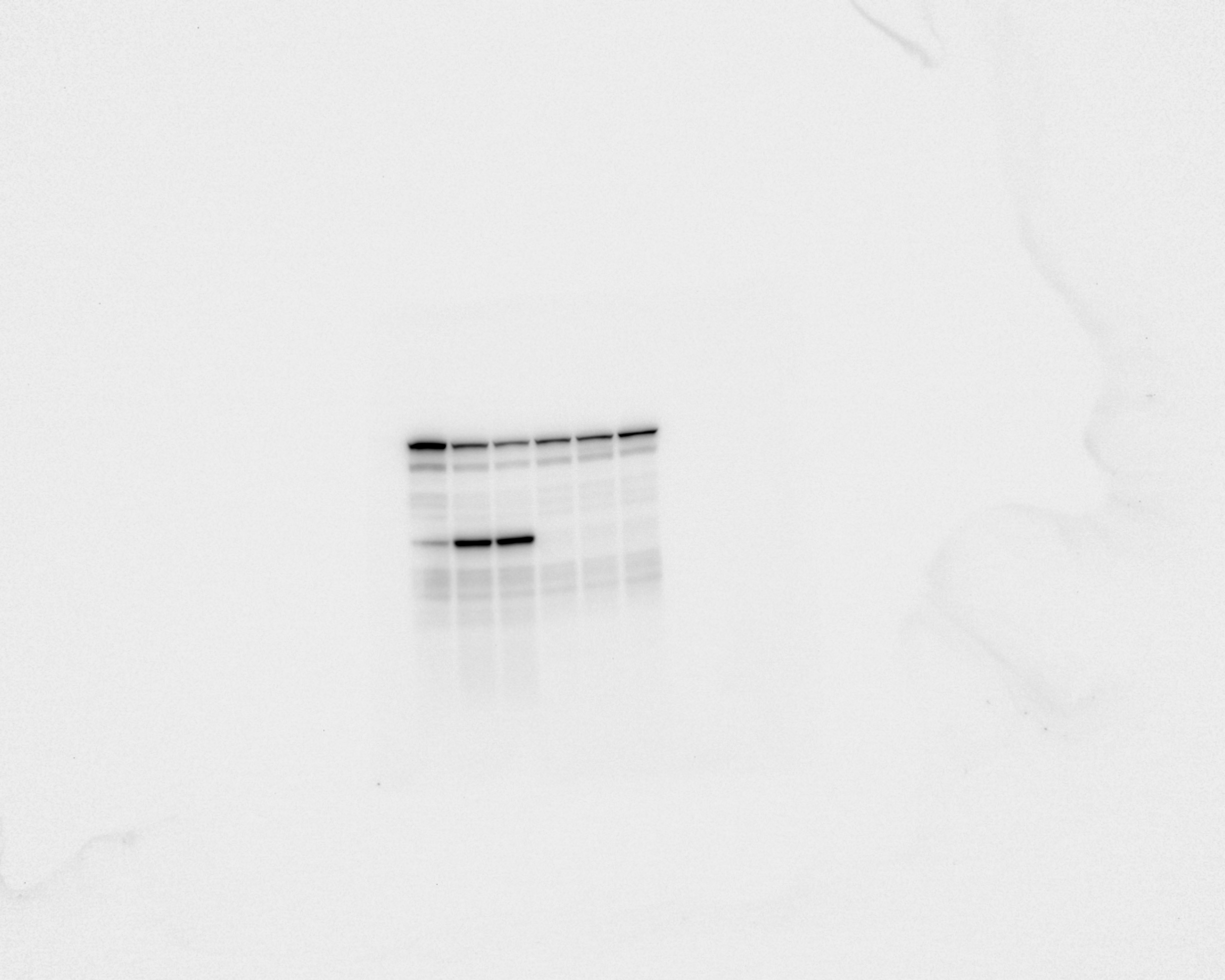

Supplement: Supplementary file 3 — Source data Fig. 1 [file 44318_2026_761_MOESM3_ESM.zip › Figure 1/1A/Repeat A/western blot V5.tif]

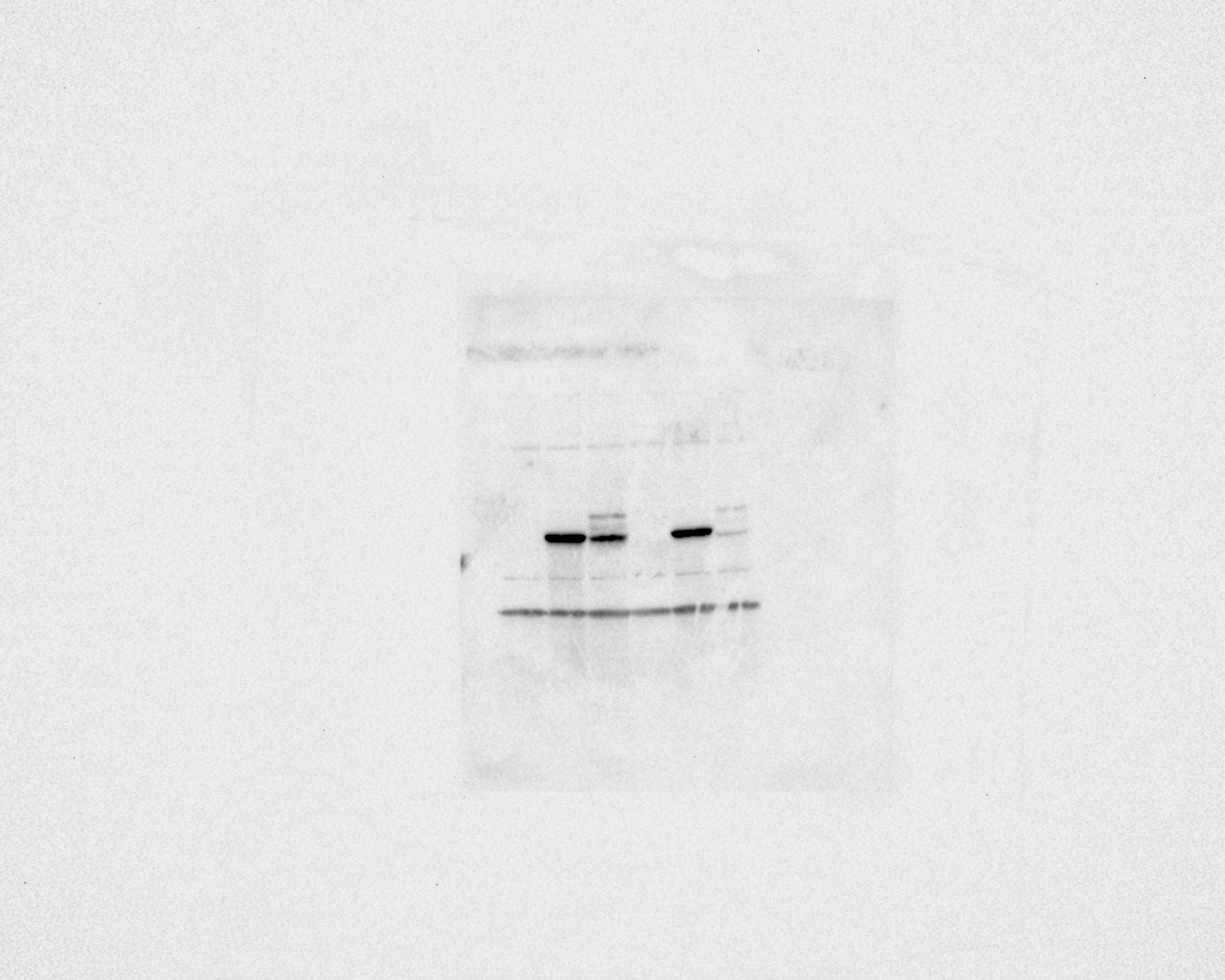

Supplement: Supplementary file 3 — Source data Fig. 1 [file 44318_2026_761_MOESM3_ESM.zip › Figure 1/1A/Repeat A/western blot myc.tif]

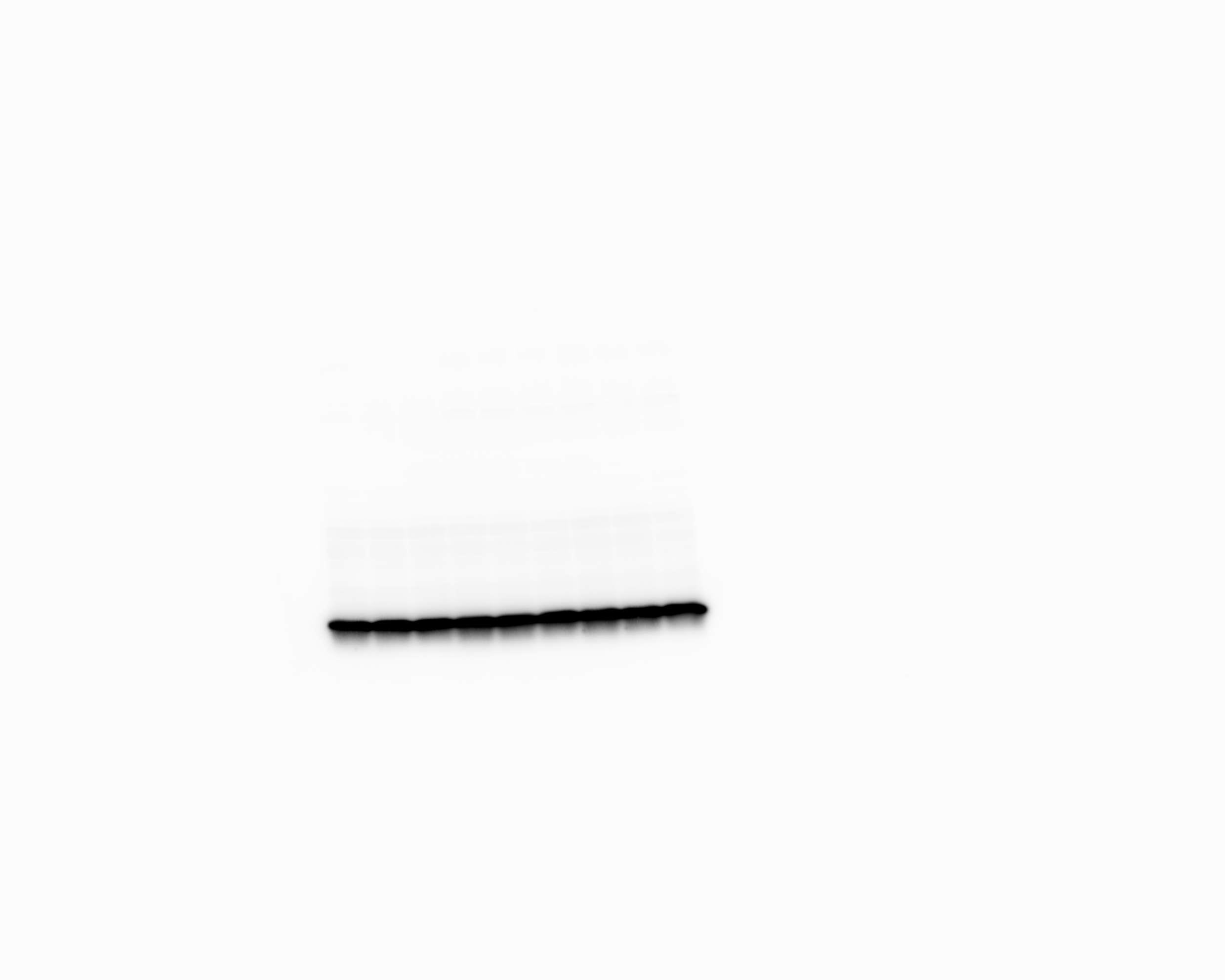

Supplement: Supplementary file 4 — Source data Fig. 2 [file 44318_2026_761_MOESM4_ESM.zip › Figure 2/2D/western blot histone H3.tif]

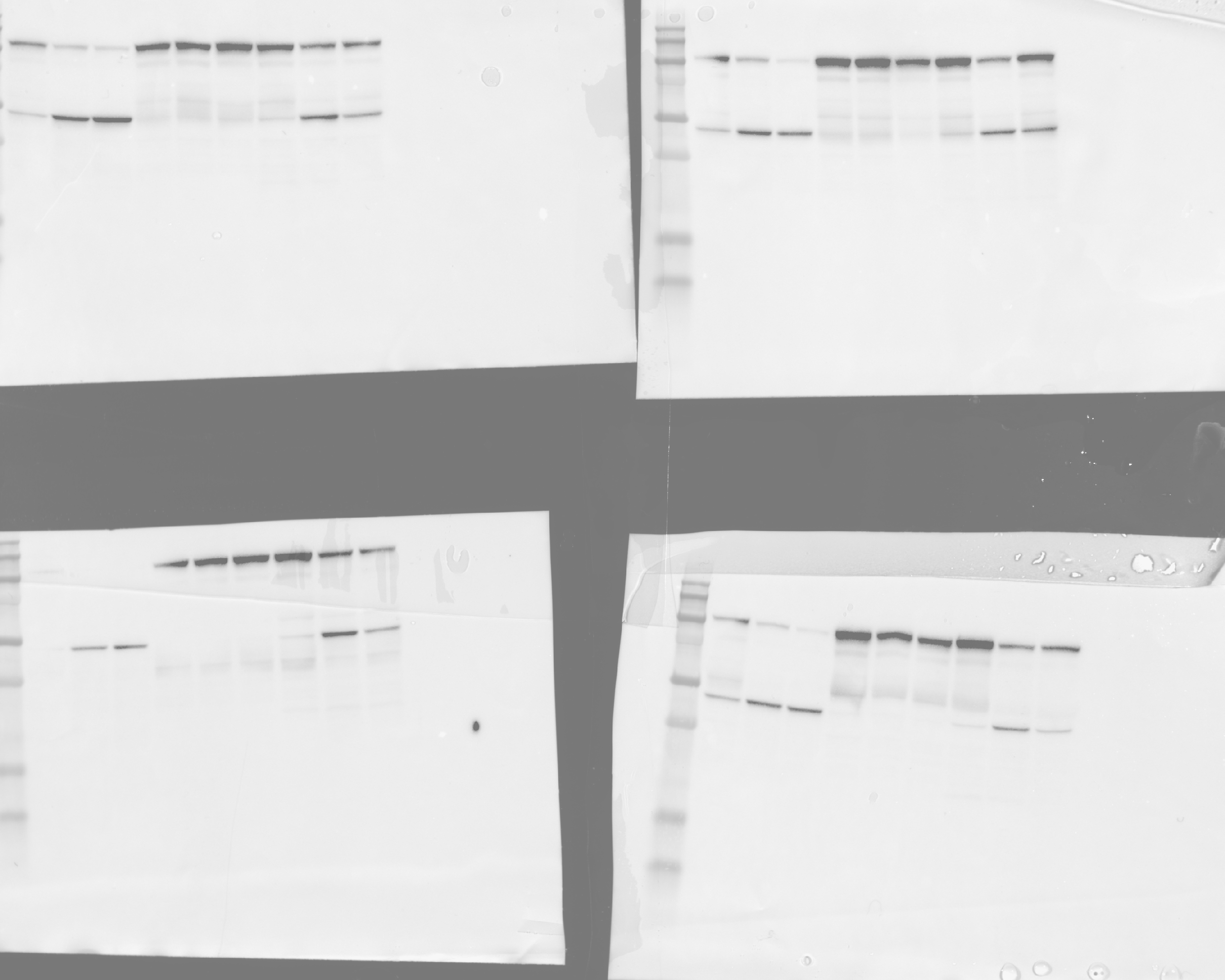

Supplement: Supplementary file 4 — Source data Fig. 2 [file 44318_2026_761_MOESM4_ESM.zip › Figure 2/2D/Kasper 2023-09-15 14h21m00s(Composite).tif]

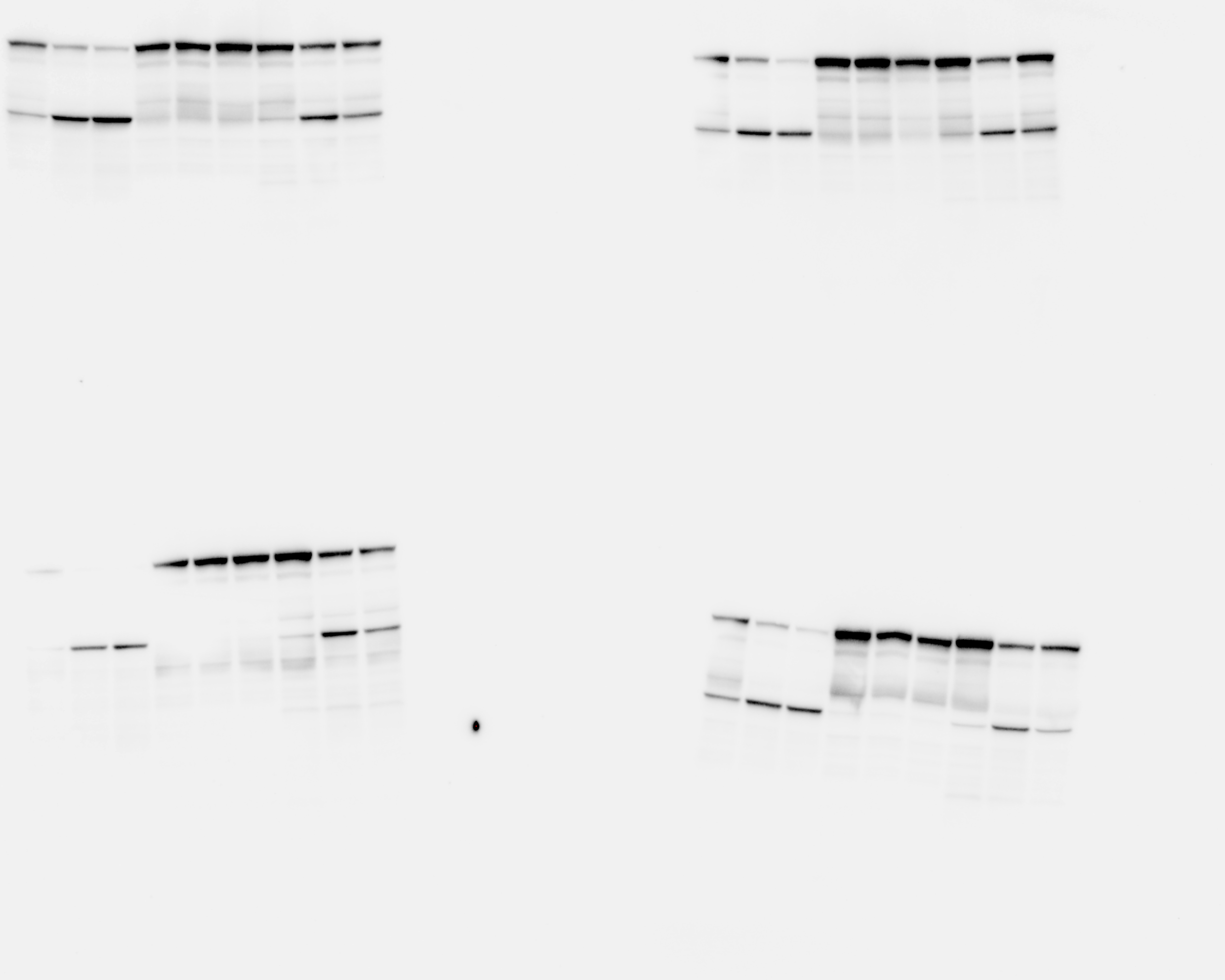

Supplement: Supplementary file 4 — Source data Fig. 2 [file 44318_2026_761_MOESM4_ESM.zip › Figure 2/2D/western blot V5 (top right).tif]

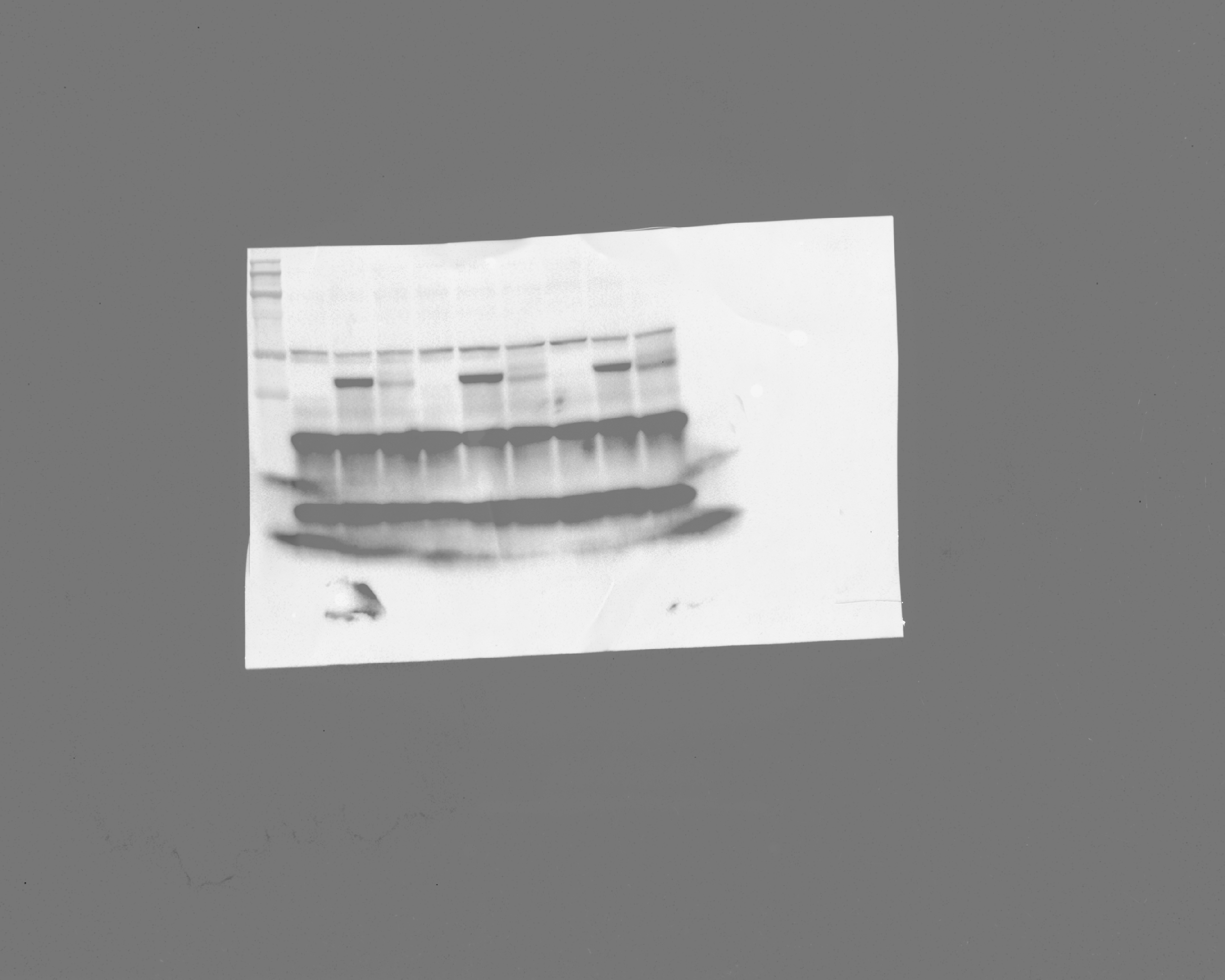

Supplement: Supplementary file 4 — Source data Fig. 2 [file 44318_2026_761_MOESM4_ESM.zip › Figure 2/2D/Kasper 2023-09-11 17h28m46s(Composite).tif]

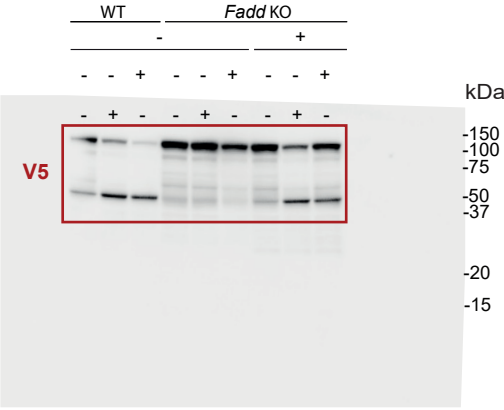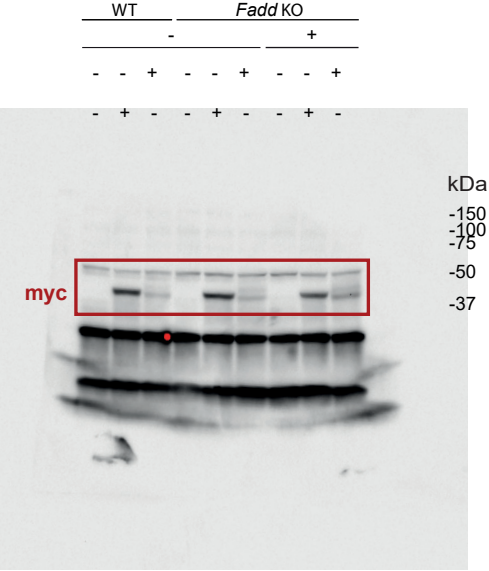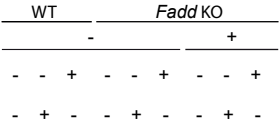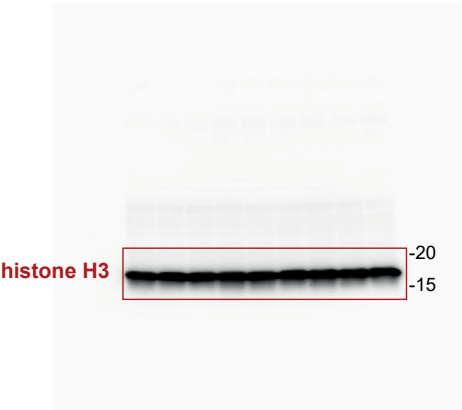

Supplement: Supplementary file 4 — Source data Fig. 2 [file 44318_2026_761_MOESM4_ESM.zip › Figure 2/2D/Annotation.pdf]

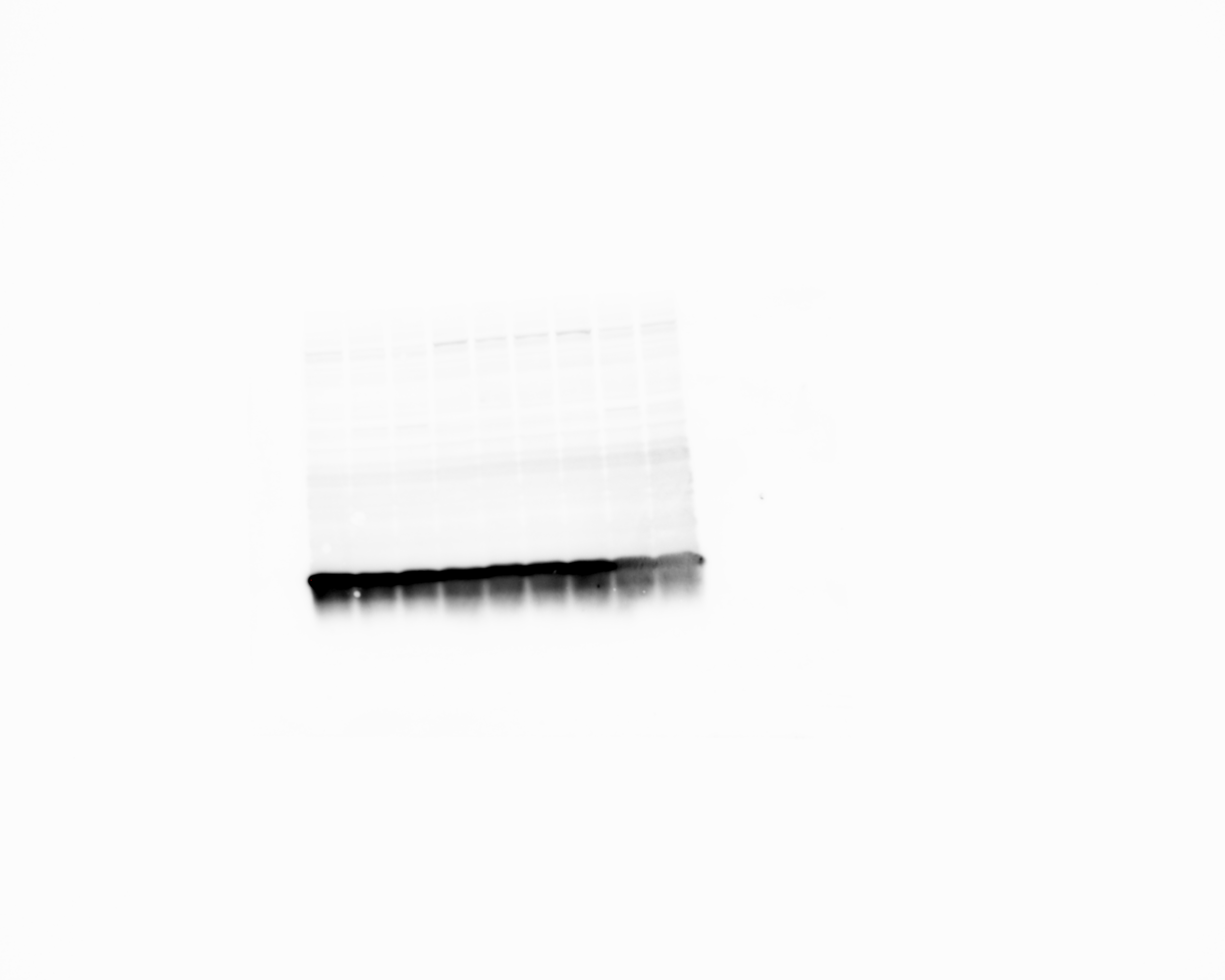

Supplement: Supplementary file 4 — Source data Fig. 2 [file 44318_2026_761_MOESM4_ESM.zip › Figure 2/2D/Repeat B/western blot histone H3.tif]

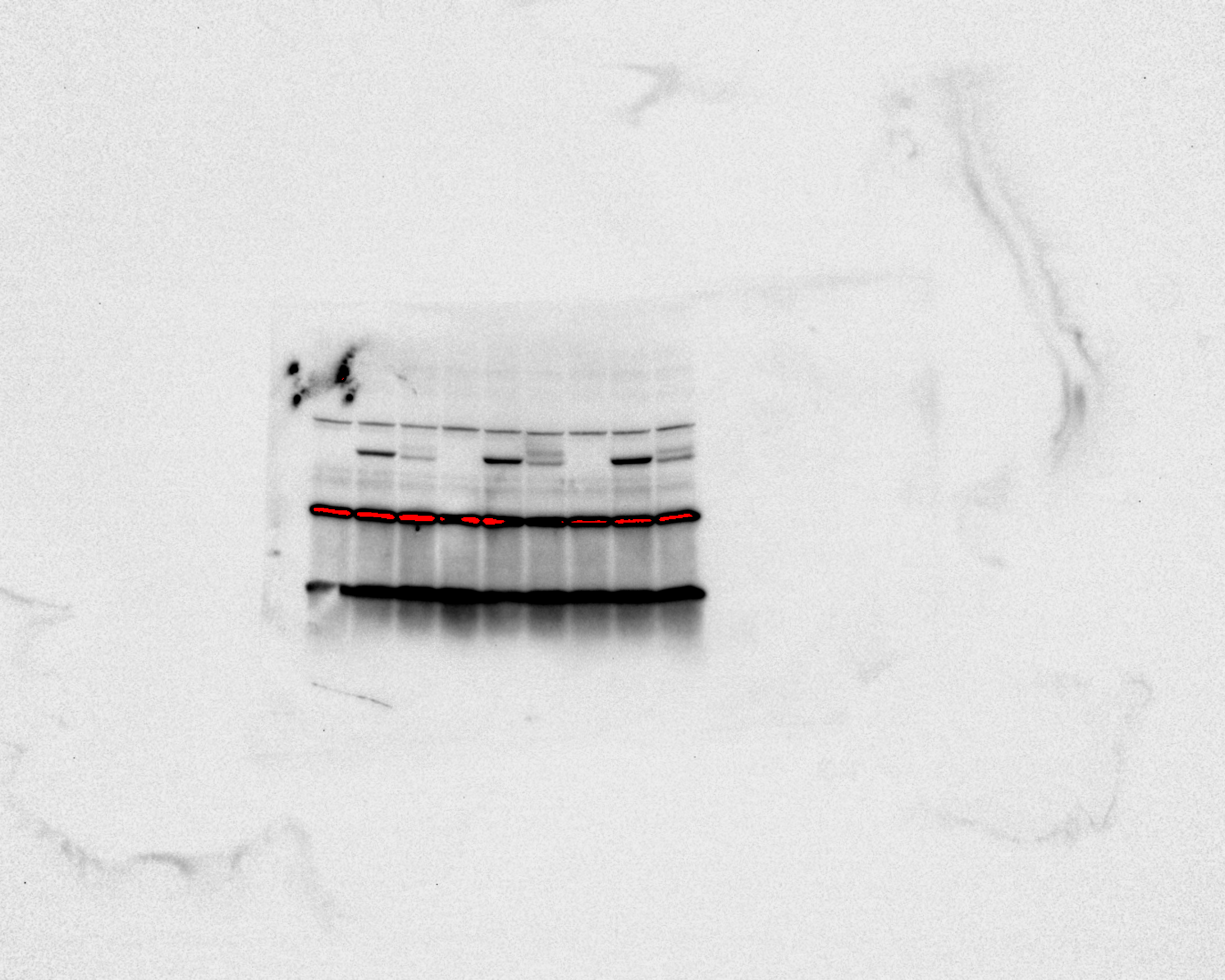

Supplement: Supplementary file 4 — Source data Fig. 2 [file 44318_2026_761_MOESM4_ESM.zip › Figure 2/2D/Repeat B/western blot myc.tif]

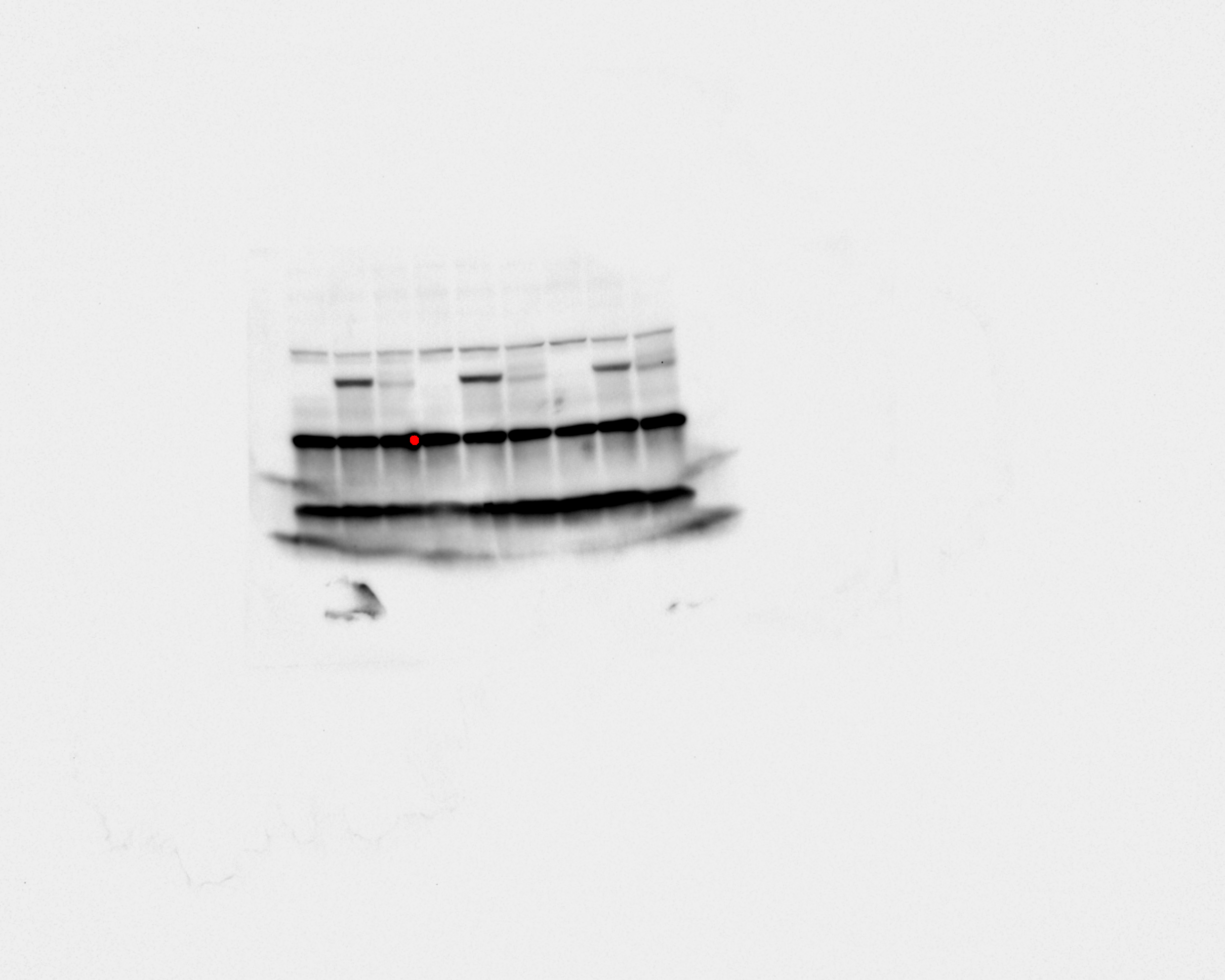

Supplement: Supplementary file 4 — Source data Fig. 2 [file 44318_2026_761_MOESM4_ESM.zip › Figure 2/2D/western blot myc.tif]

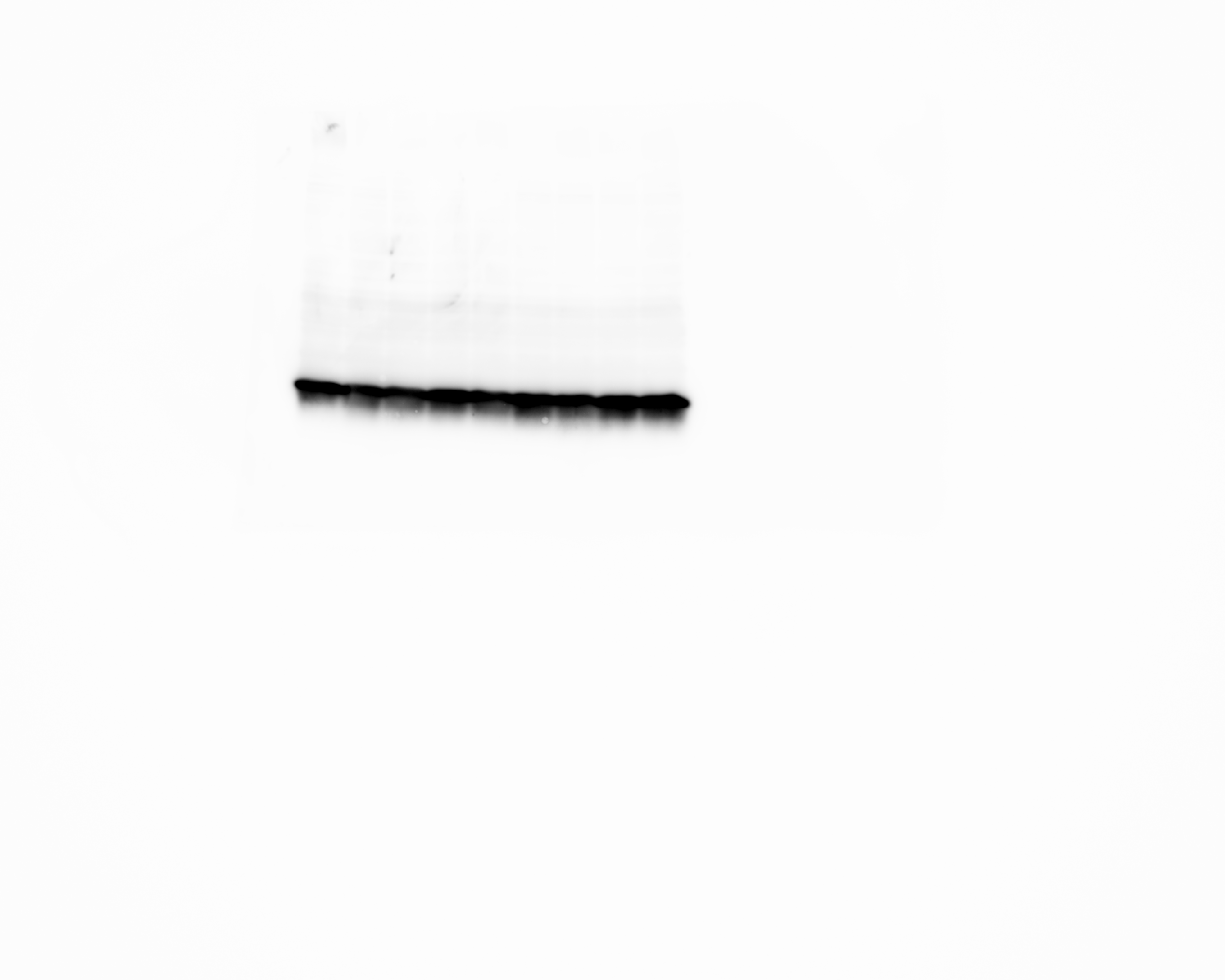

Supplement: Supplementary file 4 — Source data Fig. 2 [file 44318_2026_761_MOESM4_ESM.zip › Figure 2/2D/Repeat A/western blot histone H3.tif]

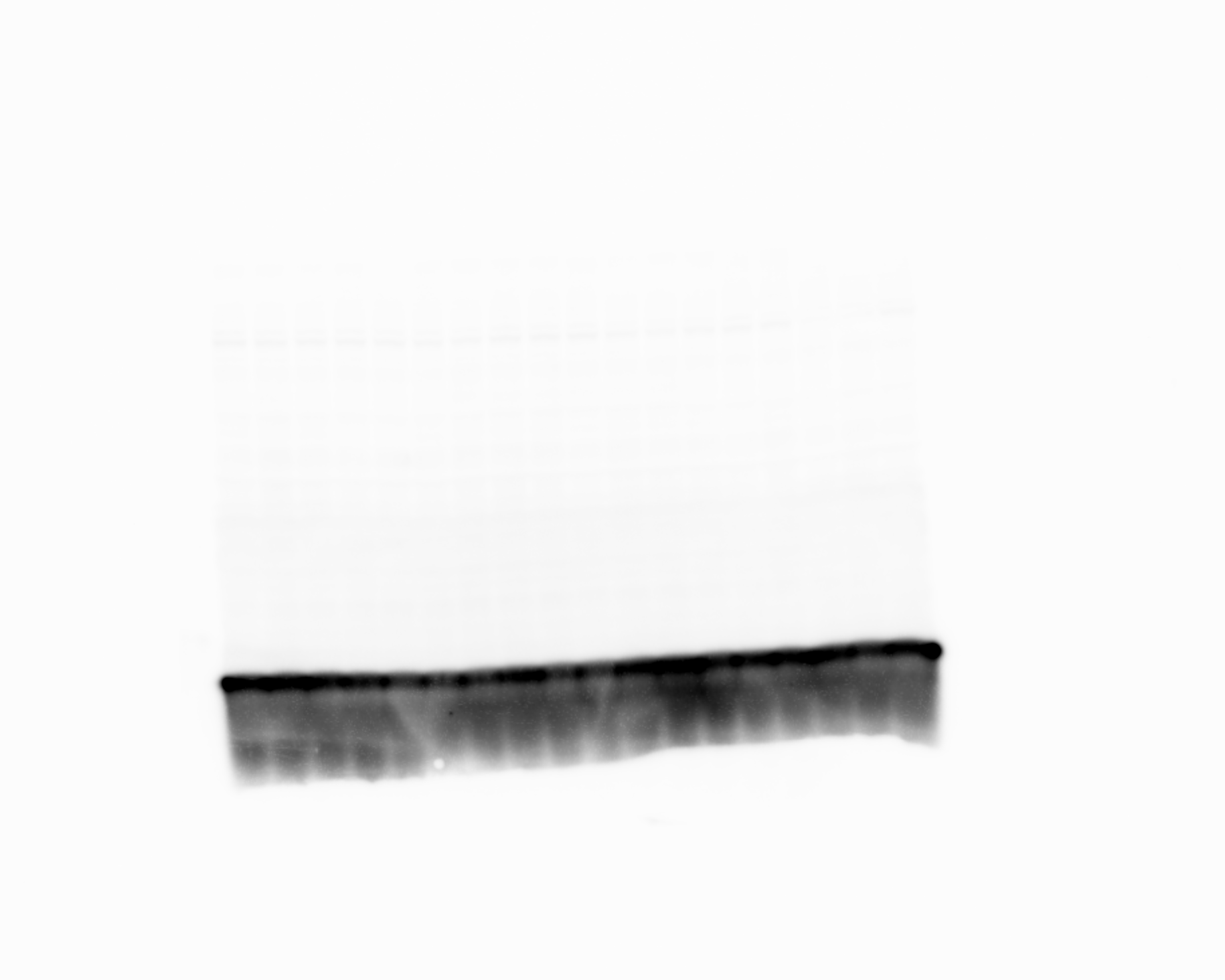

Supplement: Supplementary file 4 — Source data Fig. 2 [file 44318_2026_761_MOESM4_ESM.zip › Figure 2/2A/western blot histone H3.tif]

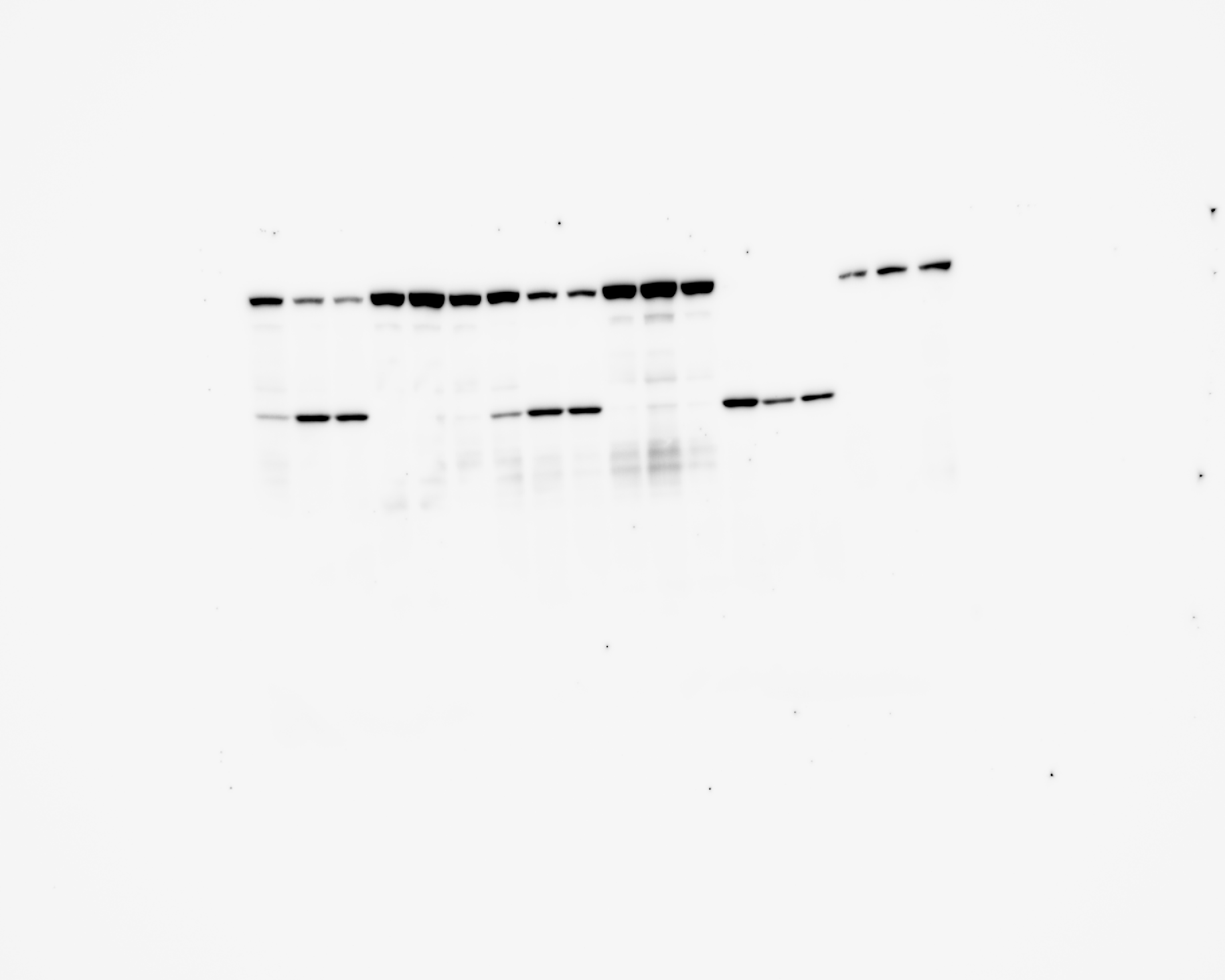

Supplement: Supplementary file 4 — Source data Fig. 2 [file 44318_2026_761_MOESM4_ESM.zip › Figure 2/2A/western blot V5.tif]

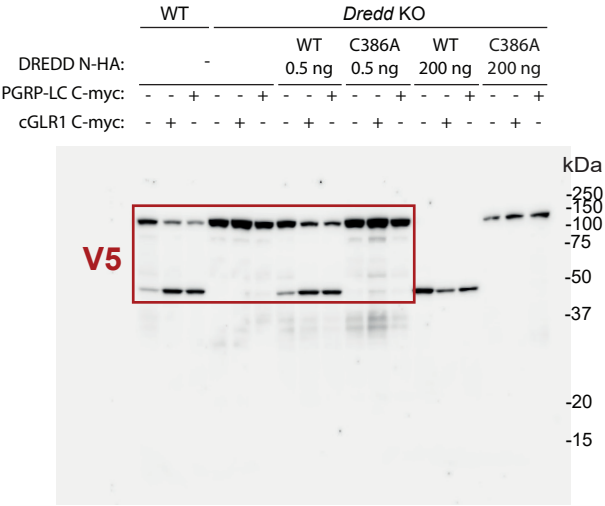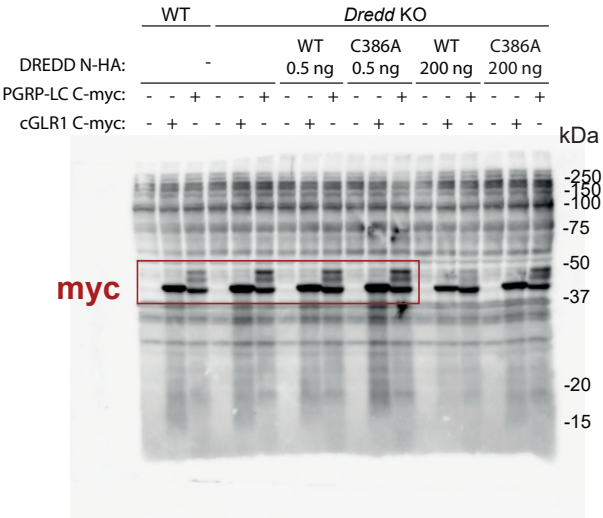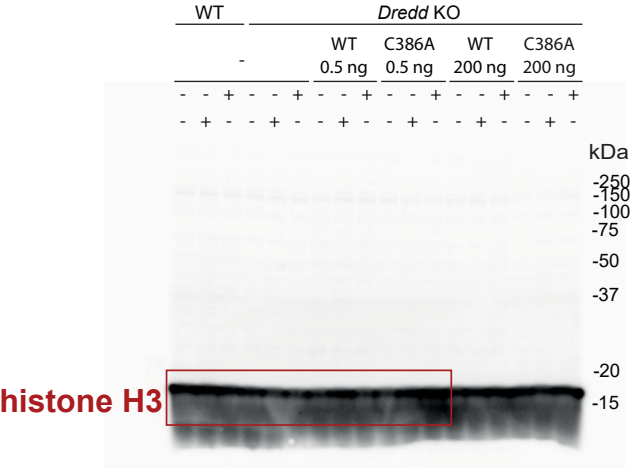

Supplement: Supplementary file 4 — Source data Fig. 2 [file 44318_2026_761_MOESM4_ESM.zip › Figure 2/2A/Annotation.pdf]

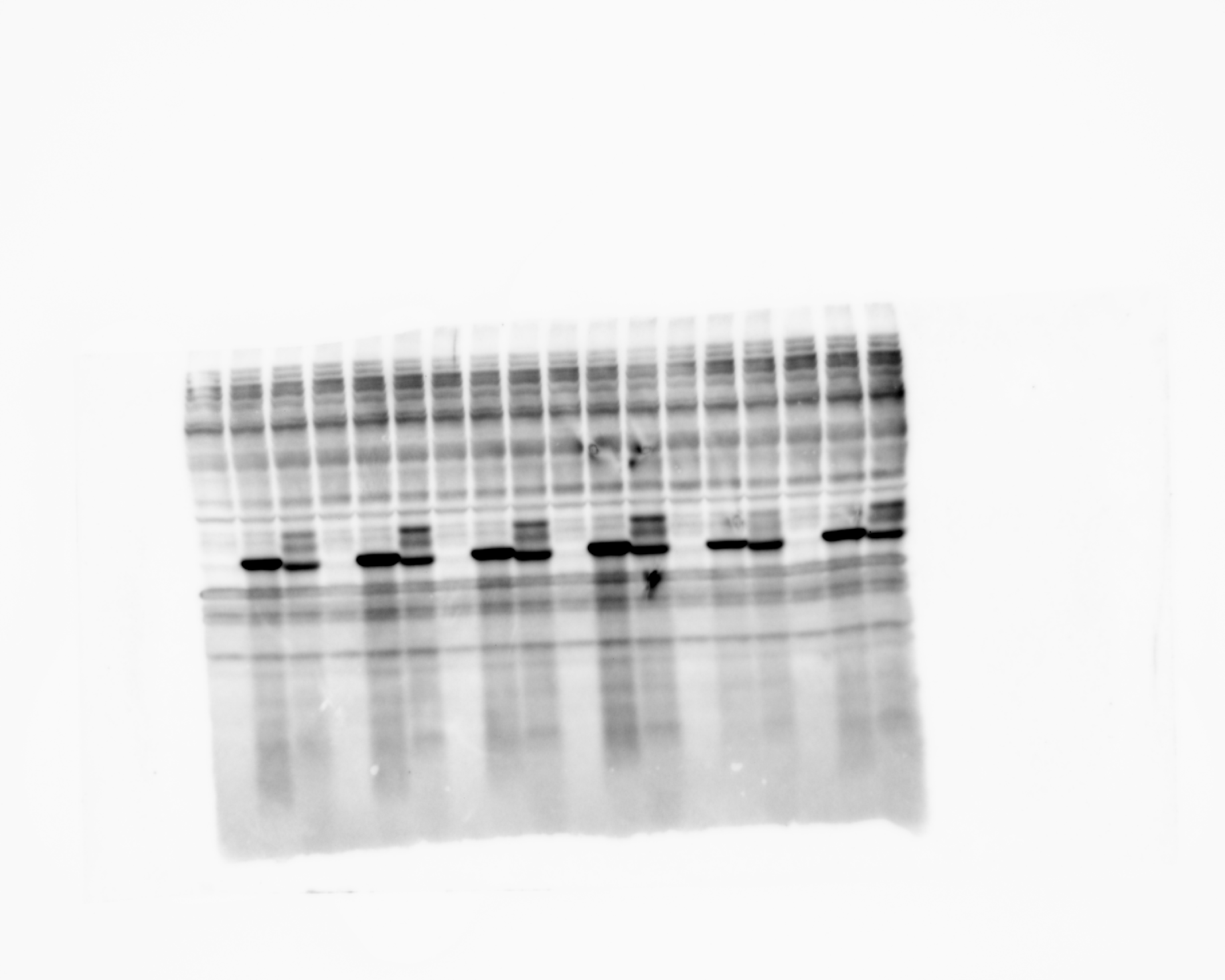

Supplement: Supplementary file 4 — Source data Fig. 2 [file 44318_2026_761_MOESM4_ESM.zip › Figure 2/2A/western blot myc.tif]

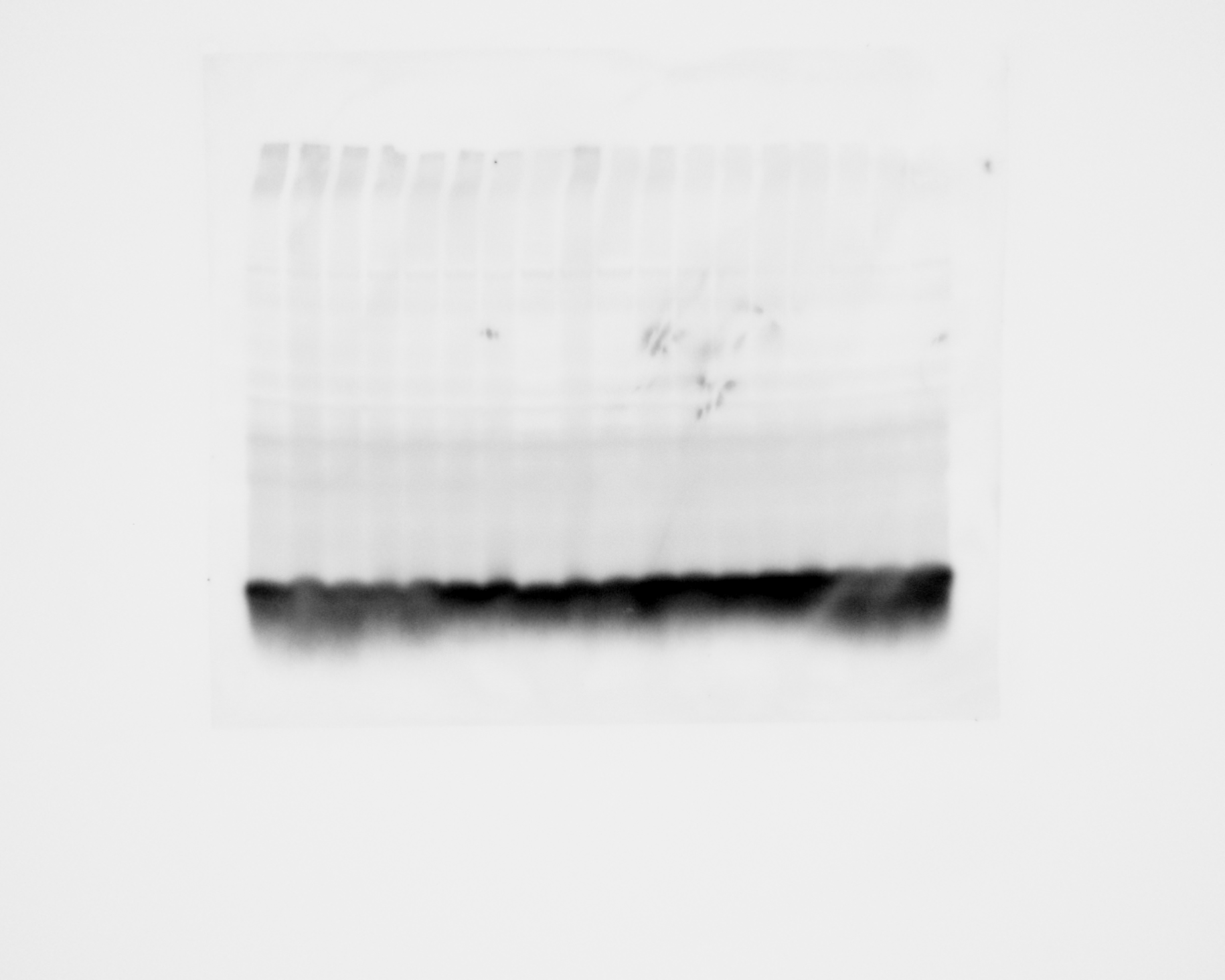

Supplement: Supplementary file 4 — Source data Fig. 2 [file 44318_2026_761_MOESM4_ESM.zip › Figure 2/2A/Repeat A/western blot histone H3.tif]

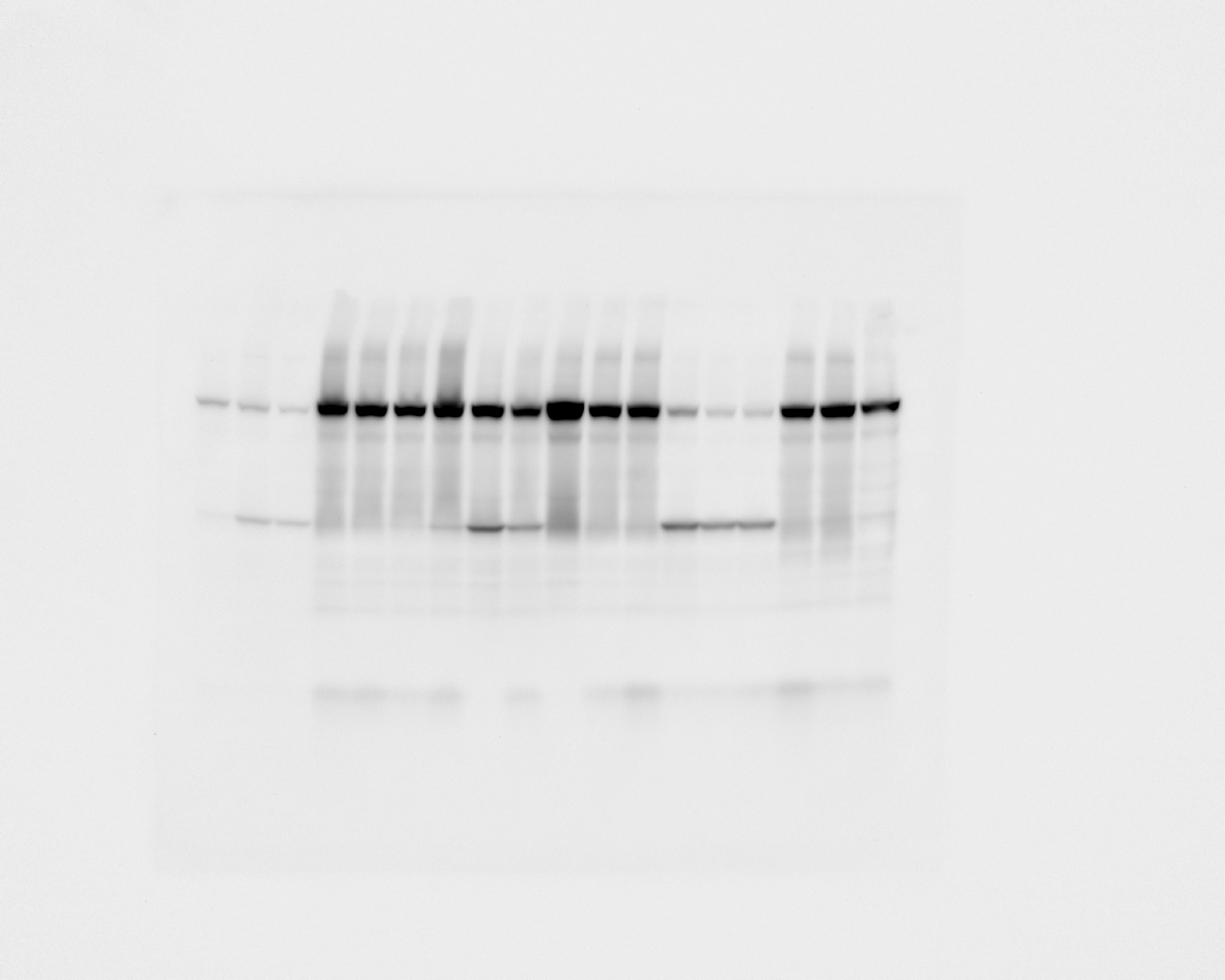

Supplement: Supplementary file 4 — Source data Fig. 2 [file 44318_2026_761_MOESM4_ESM.zip › Figure 2/2A/Repeat A/western blot V5.tif]

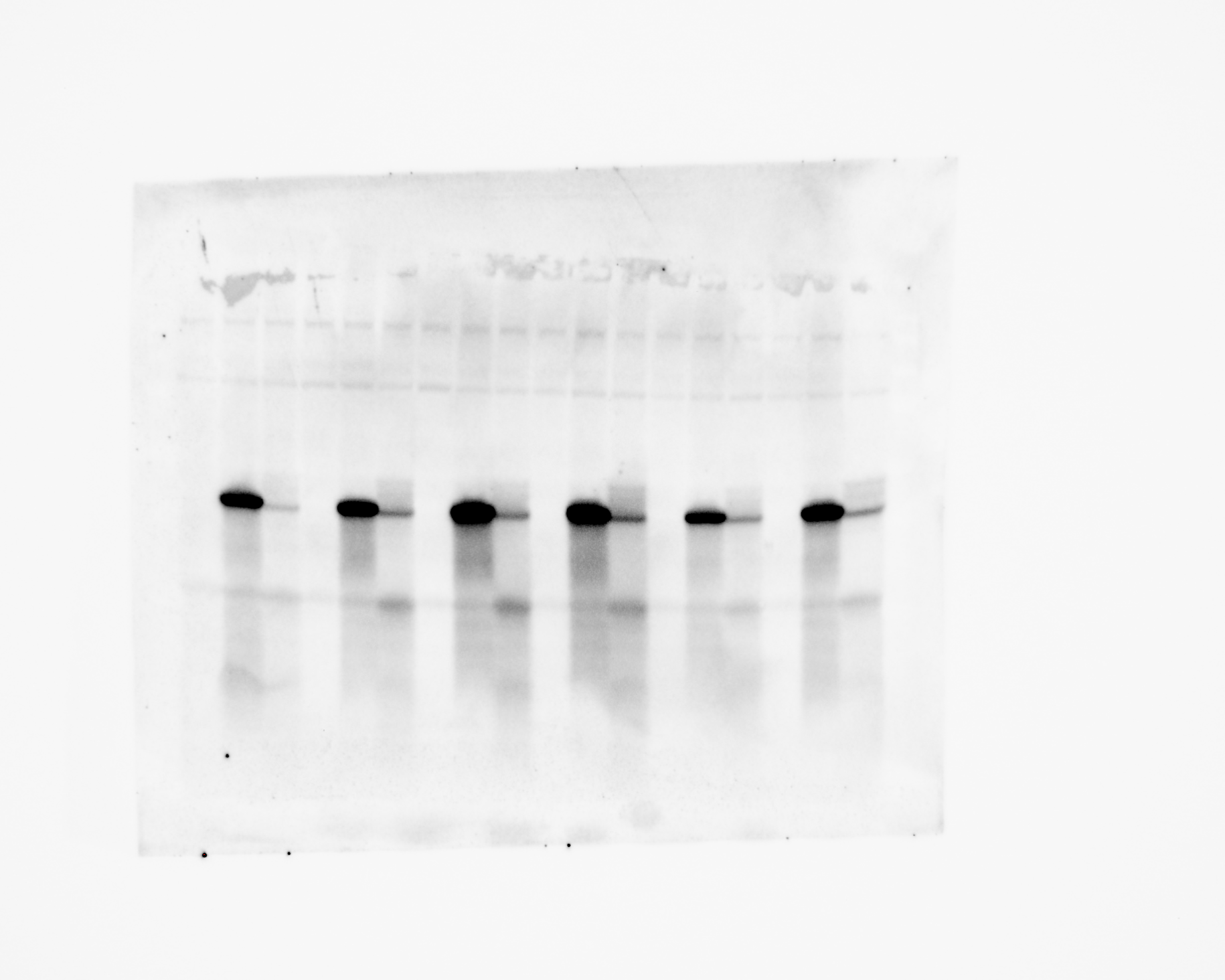

Supplement: Supplementary file 4 — Source data Fig. 2 [file 44318_2026_761_MOESM4_ESM.zip › Figure 2/2A/Repeat A/western blot myc.tif]

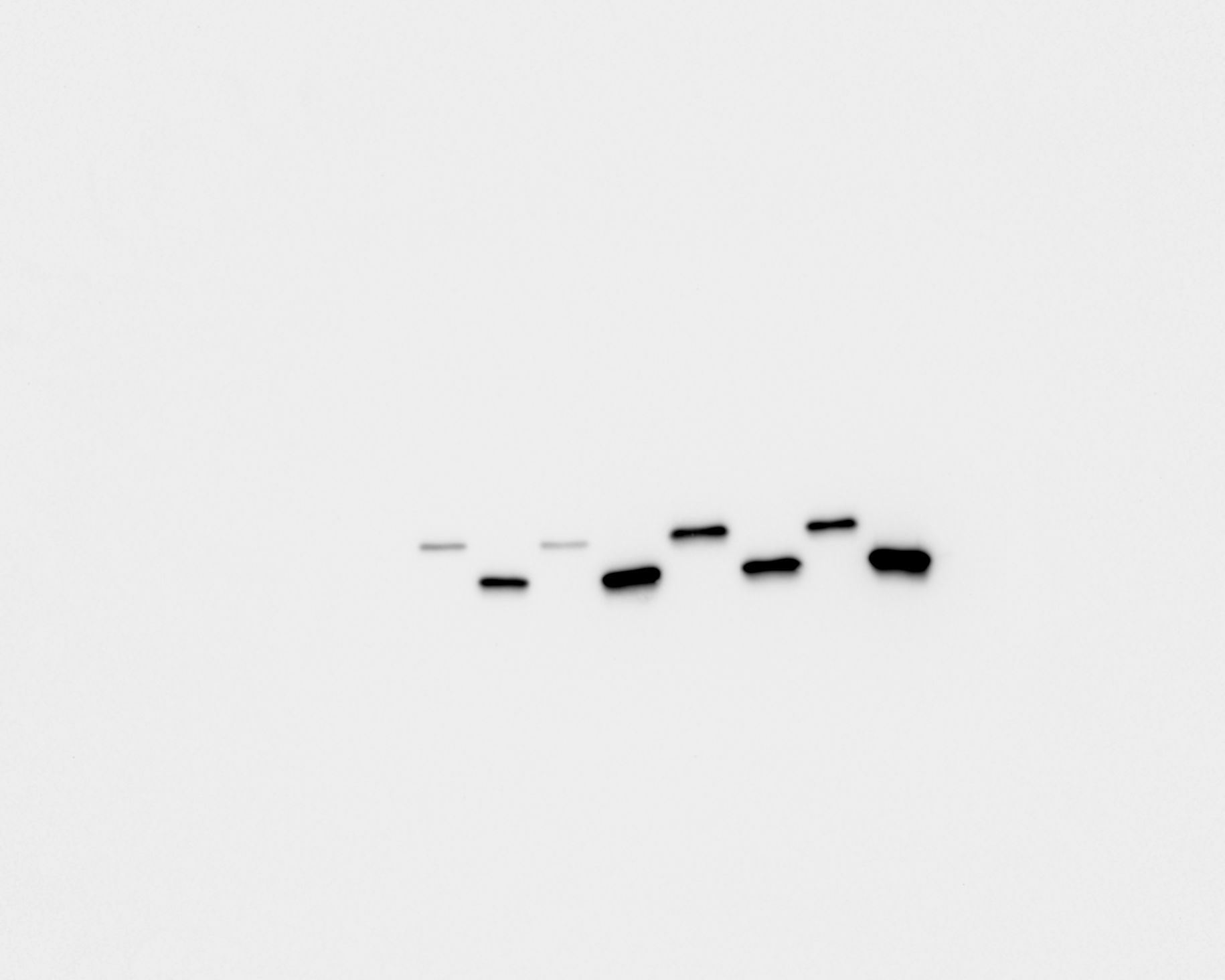

Supplement: Supplementary file 5 — Source data Fig. 3 [file 44318_2026_761_MOESM5_ESM.zip › Figure 3/3E/western blot V5.tif]

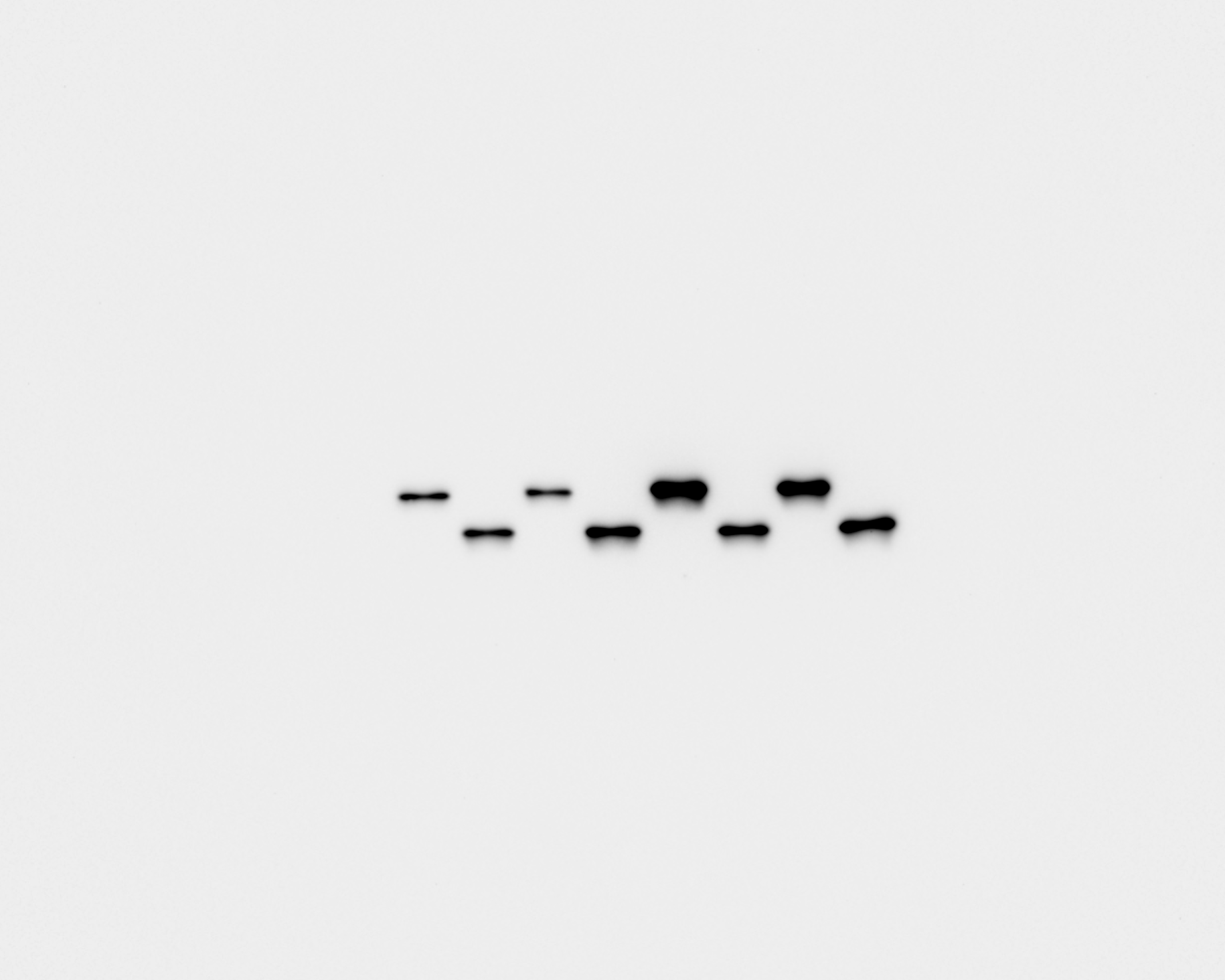

Supplement: Supplementary file 5 — Source data Fig. 3 [file 44318_2026_761_MOESM5_ESM.zip › Figure 3/3E/Repeat B/western blot V5.tif]

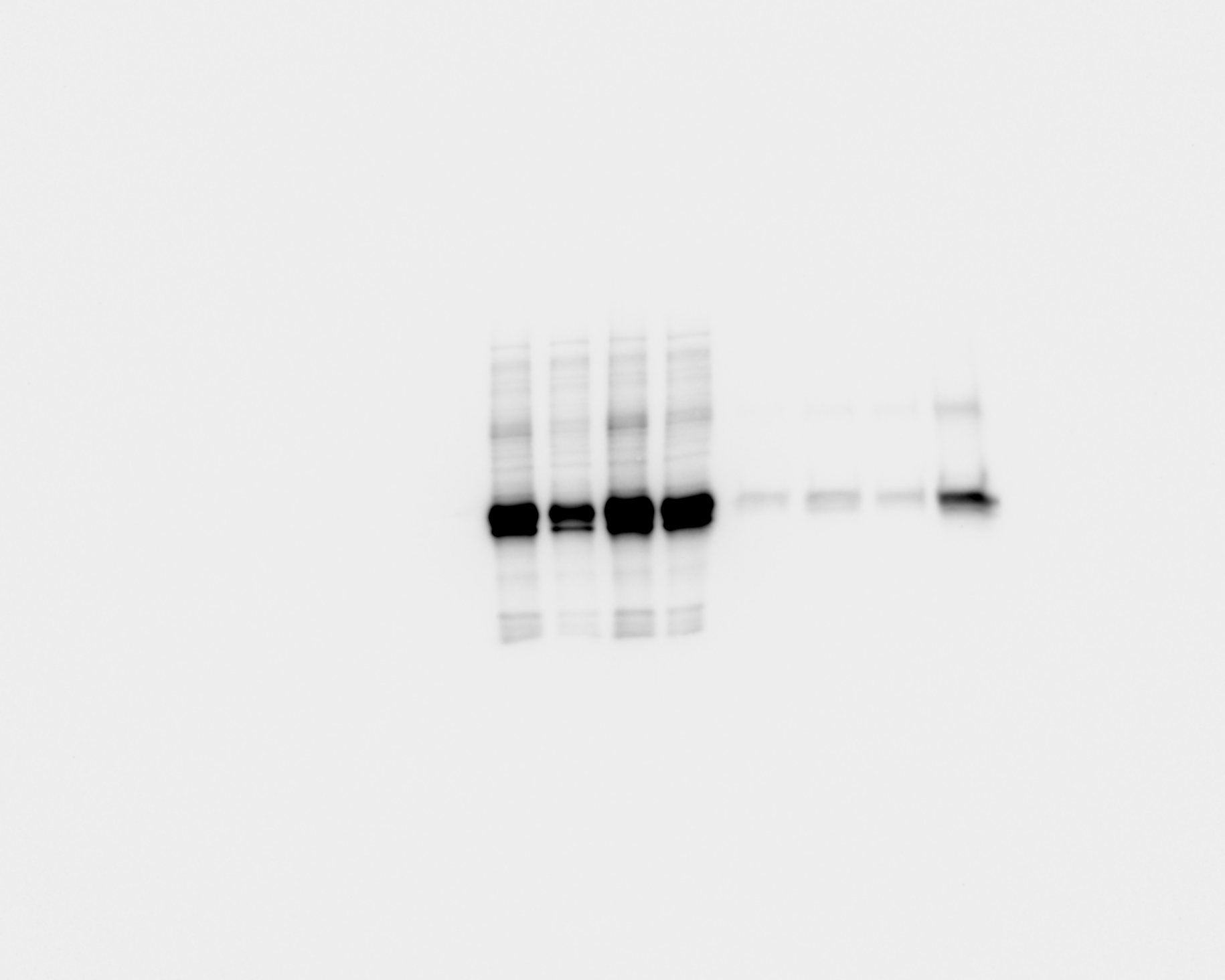

Supplement: Supplementary file 5 — Source data Fig. 3 [file 44318_2026_761_MOESM5_ESM.zip › Figure 3/3E/Repeat B/western blot HA.tif]

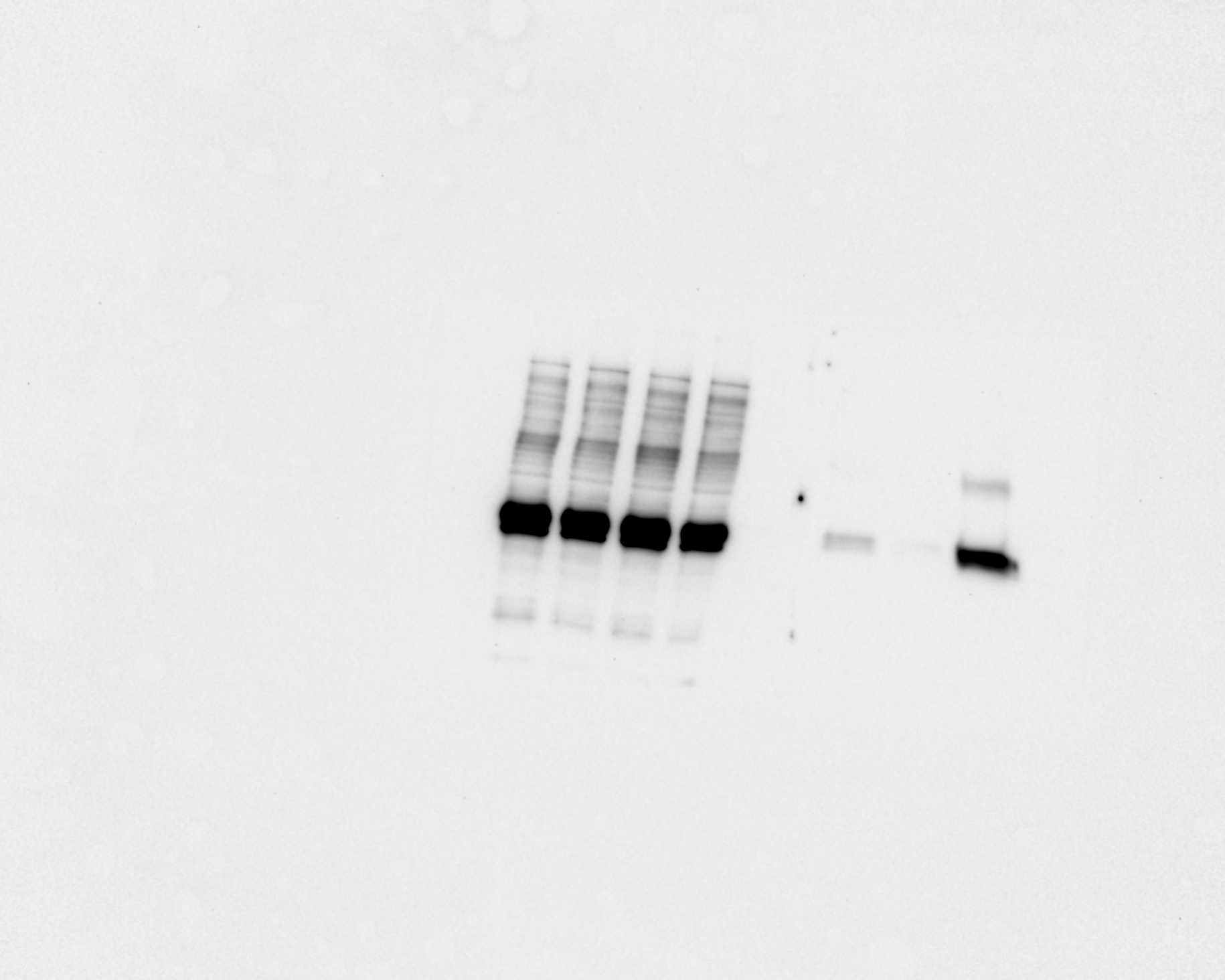

Supplement: Supplementary file 5 — Source data Fig. 3 [file 44318_2026_761_MOESM5_ESM.zip › Figure 3/3E/western blot HA.tif]

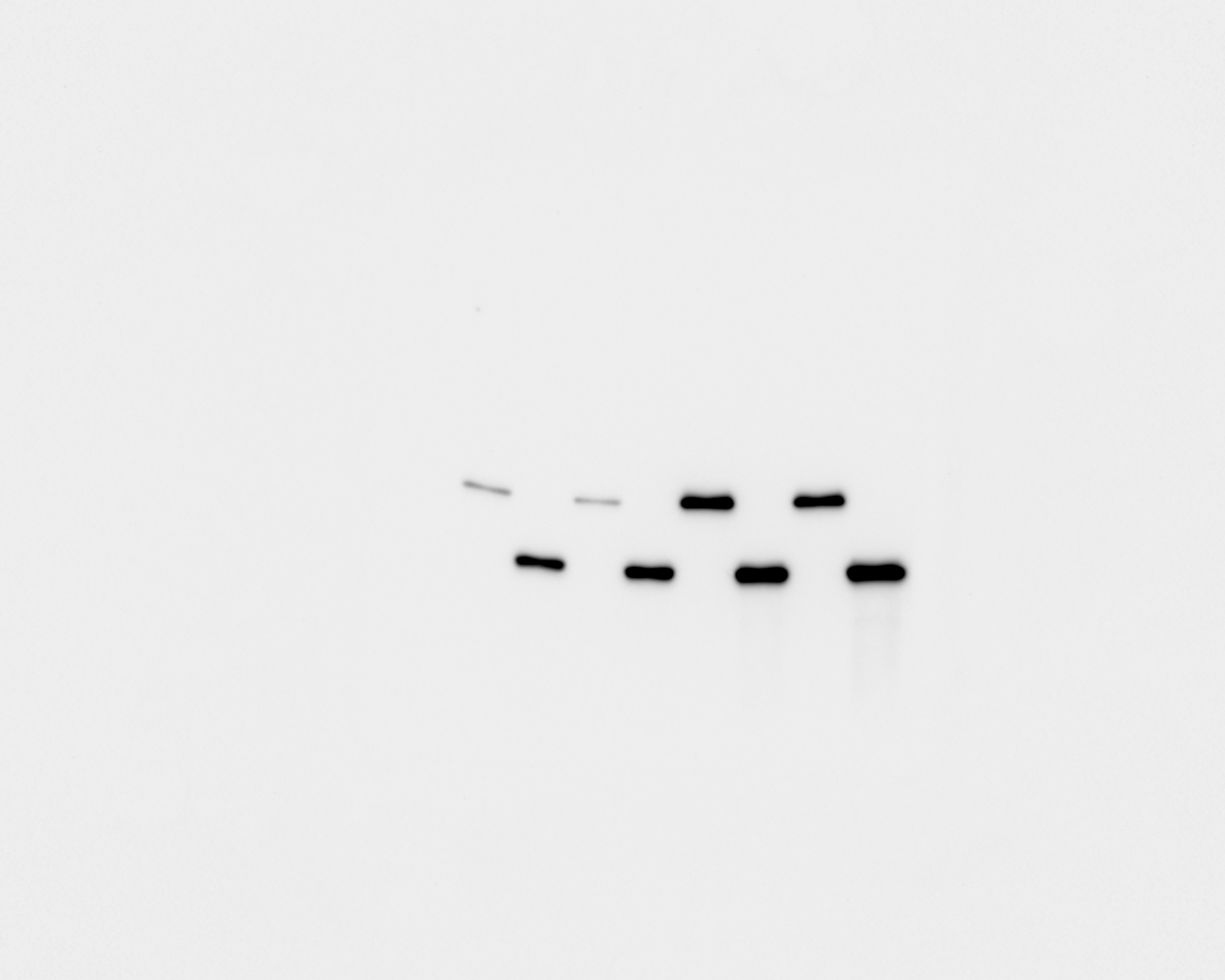

Supplement: Supplementary file 5 — Source data Fig. 3 [file 44318_2026_761_MOESM5_ESM.zip › Figure 3/3E/Repeat A/western blot V5.tif]

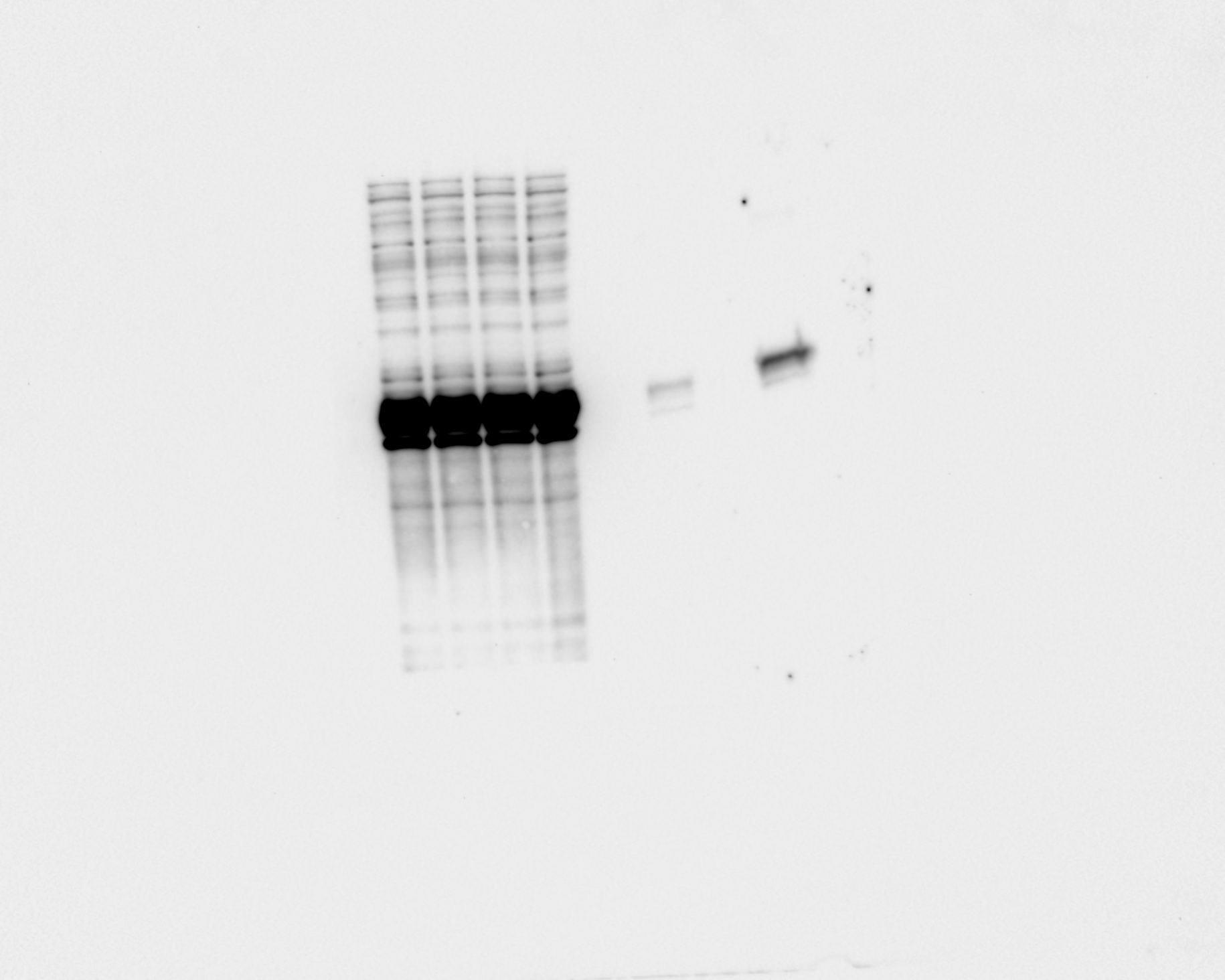

Supplement: Supplementary file 5 — Source data Fig. 3 [file 44318_2026_761_MOESM5_ESM.zip › Figure 3/3E/Repeat A/western blot HA.tif]

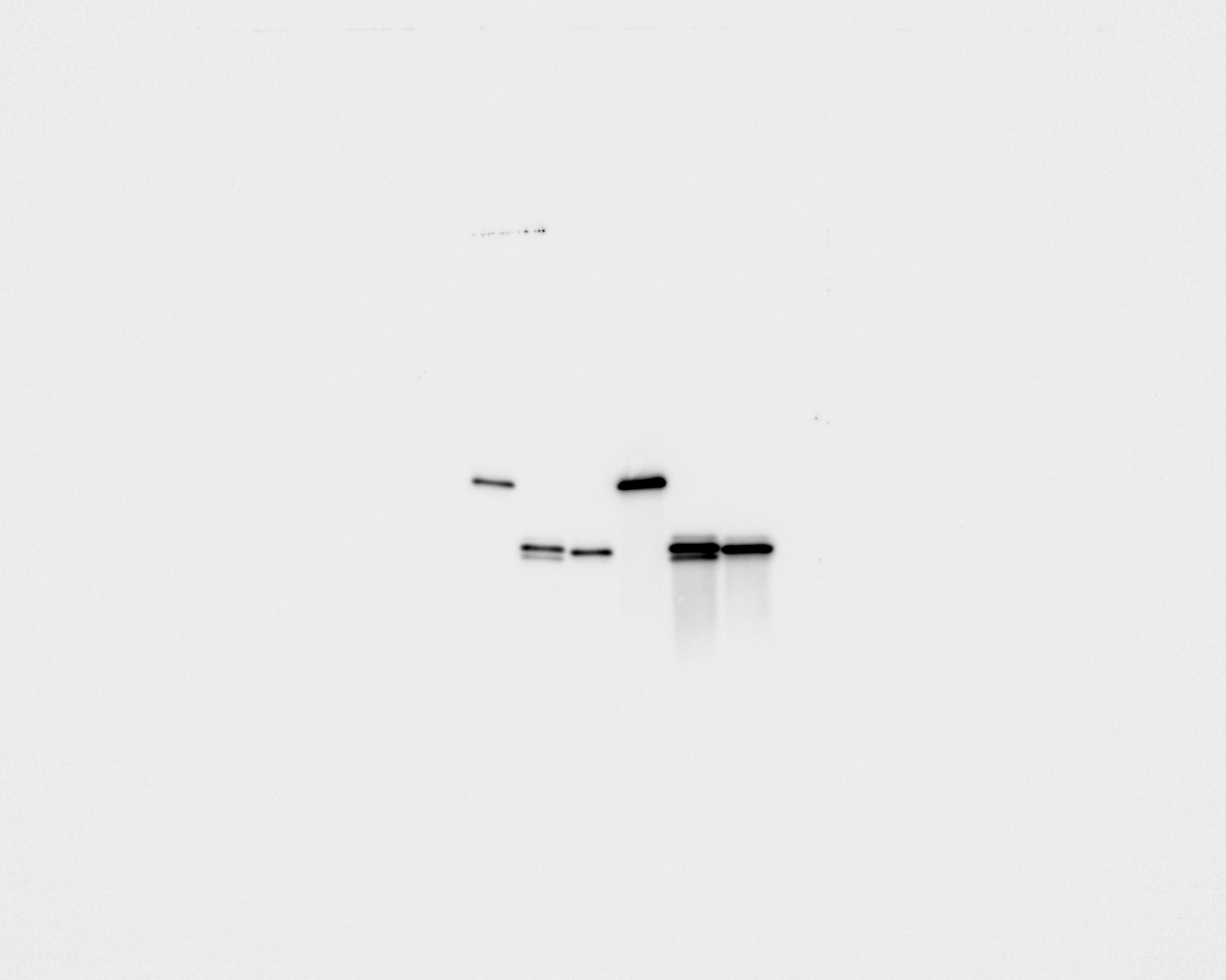

Supplement: Supplementary file 6 — Source data Fig. 4 [file 44318_2026_761_MOESM6_ESM.zip › Figure 4/4D/western blot V5.tif]

RLuc-V5  
Fadd-V5  
R39A/R40A-V5  
RLuc-V5  
Fadd-V5  
R39A/R40A-V5

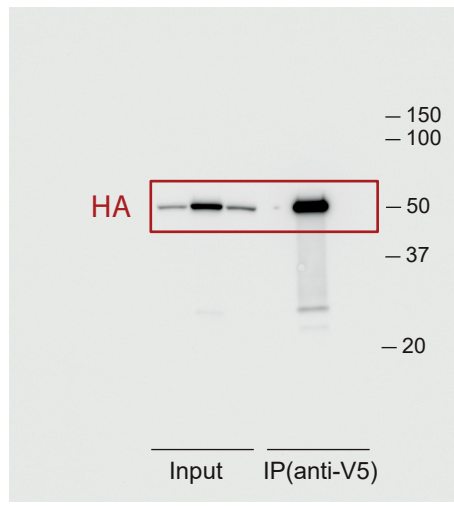

RLuc-V5  
Fadd-V5  
R39A/R40A-V5  
RLuc-V5  
Fadd-V5  
R39A/R40A-V5

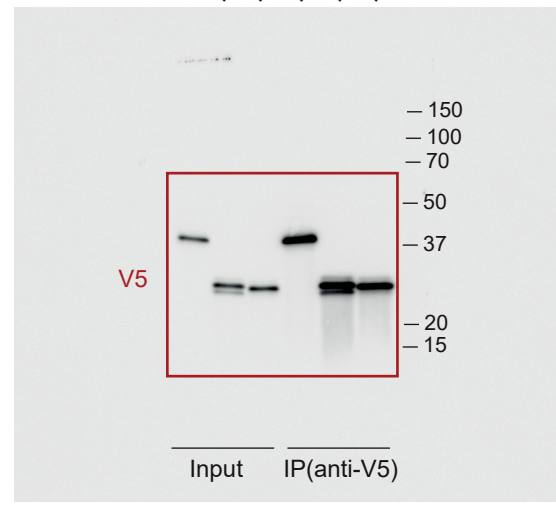

Supplement: Supplementary file 6 — Source data Fig. 4 [file 44318_2026_761_MOESM6_ESM.zip › Figure 4/4D/Annotation.pdf]

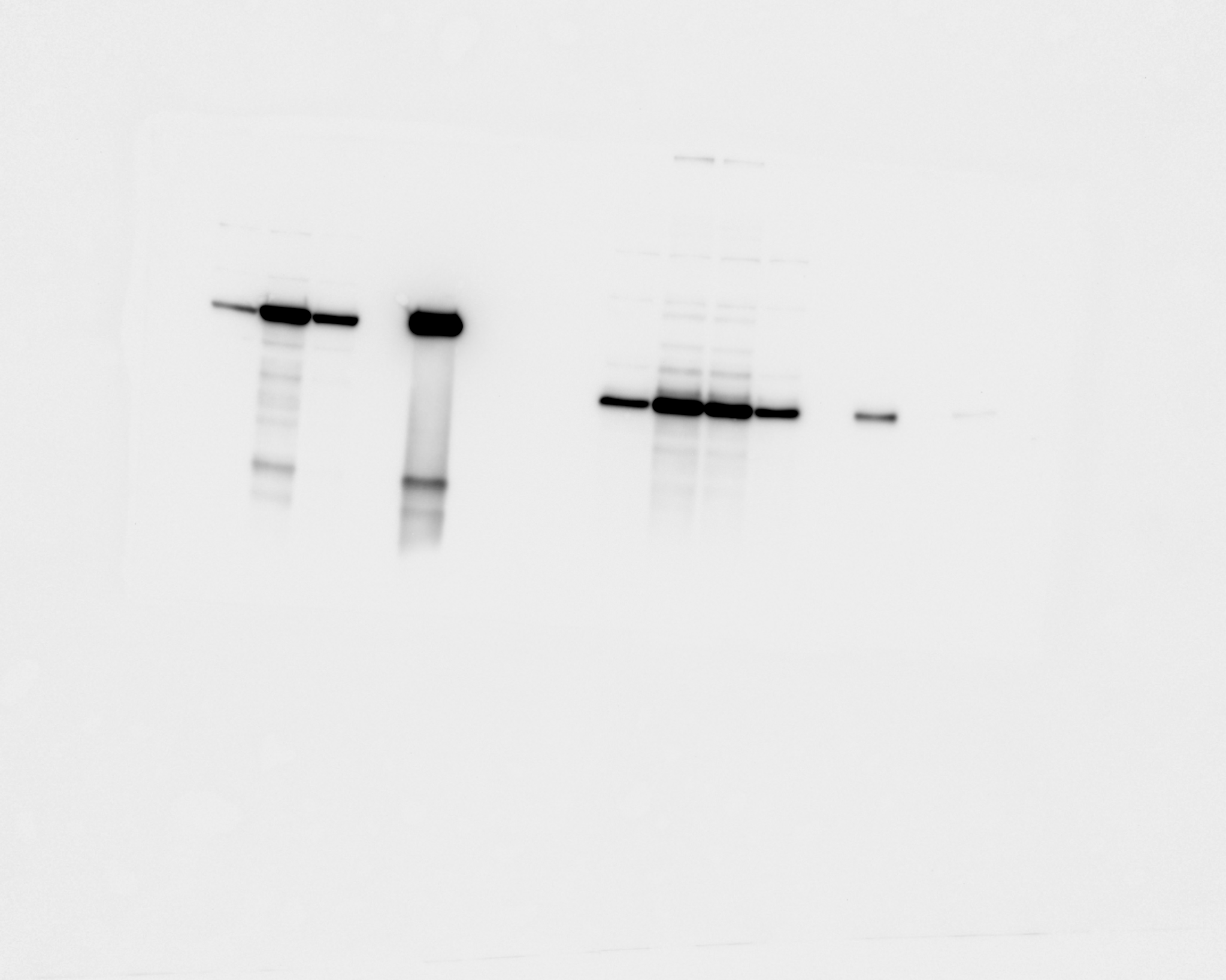

Supplement: Supplementary file 6 — Source data Fig. 4 [file 44318_2026_761_MOESM6_ESM.zip › Figure 4/4D/Repeat B/western blot HA (left 6 lanes).tif]

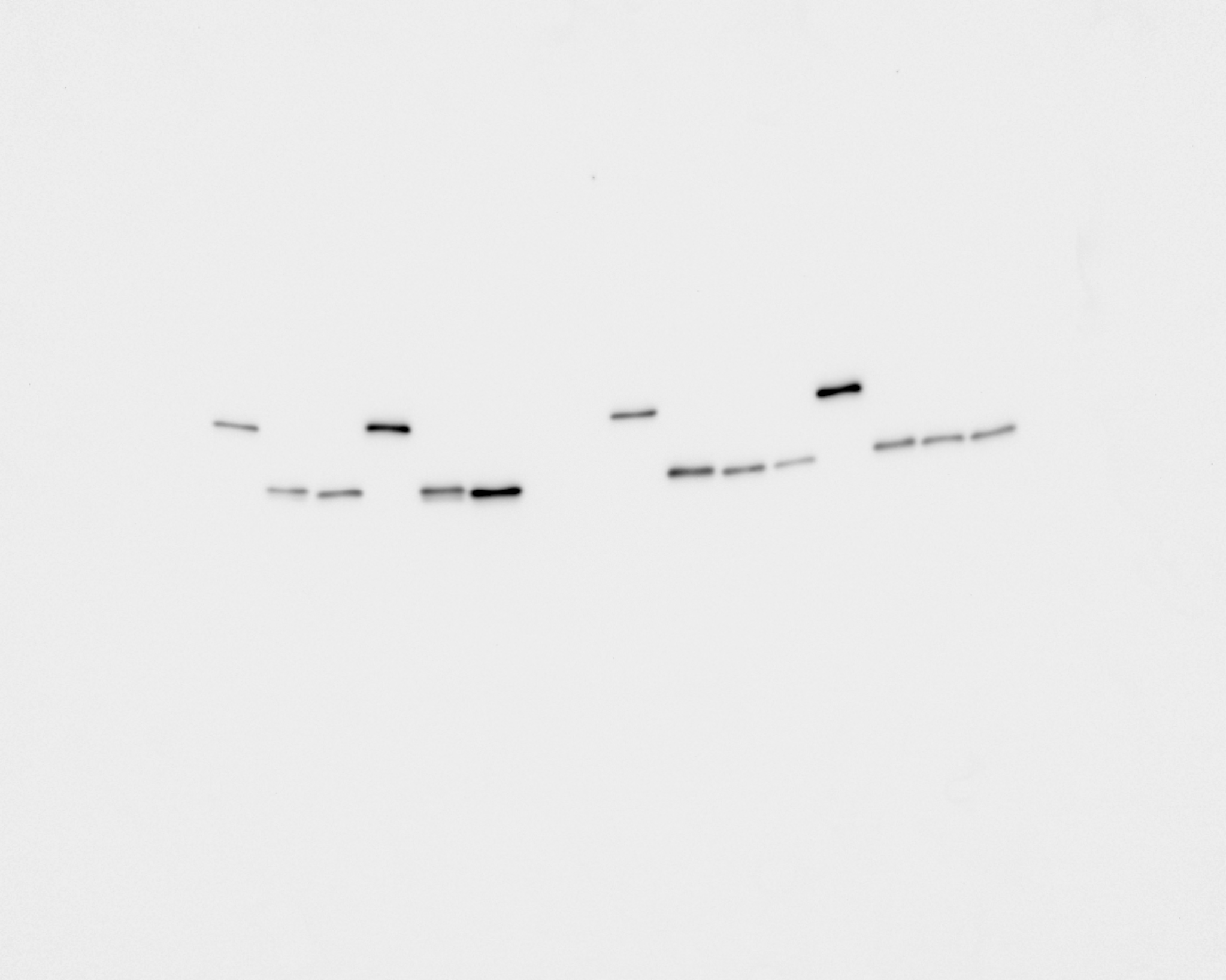

Supplement: Supplementary file 6 — Source data Fig. 4 [file 44318_2026_761_MOESM6_ESM.zip › Figure 4/4D/Repeat B/wetsern blot V5`(left 6 lanes).tif]

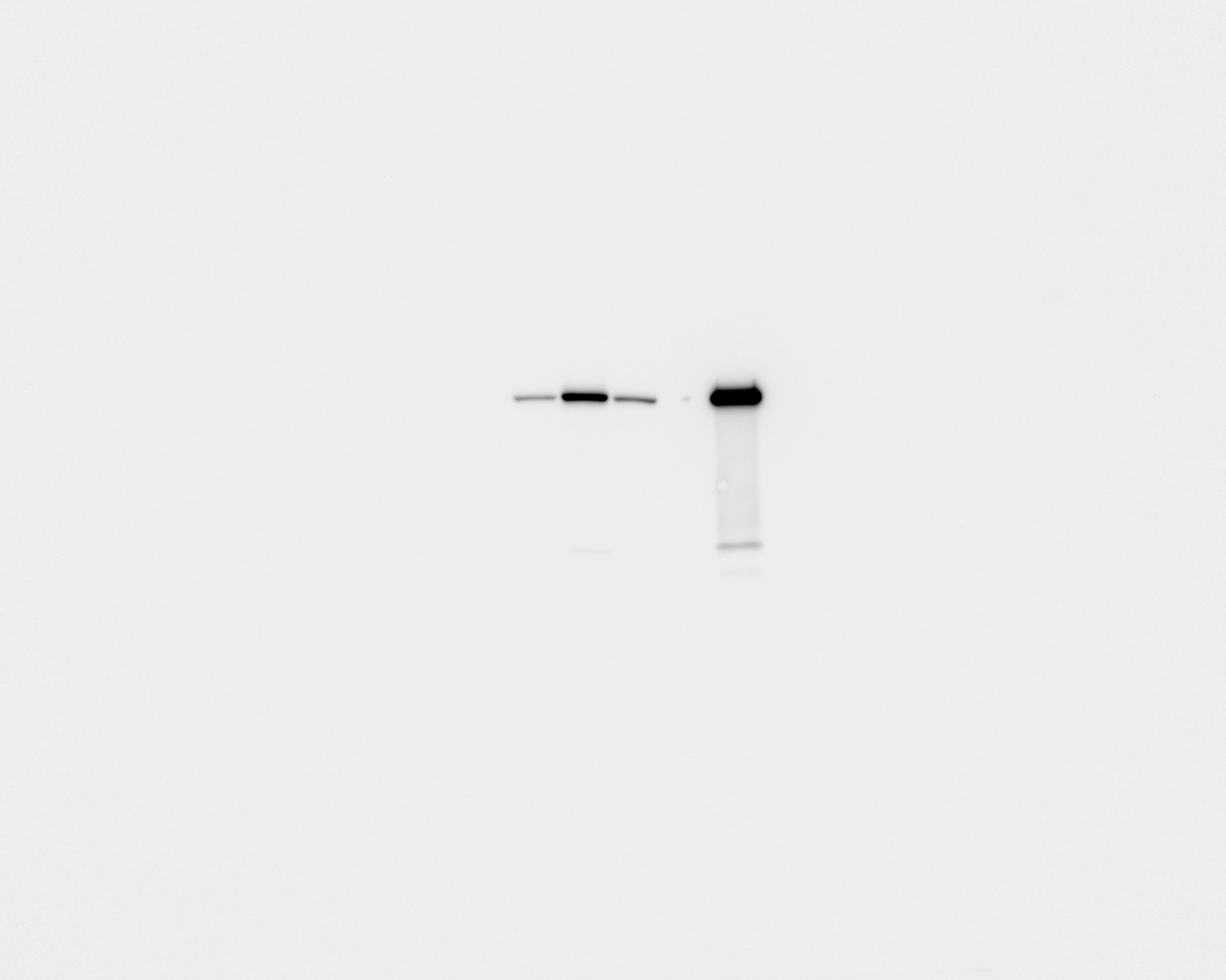

Supplement: Supplementary file 6 — Source data Fig. 4 [file 44318_2026_761_MOESM6_ESM.zip › Figure 4/4D/western blot HA.tif]

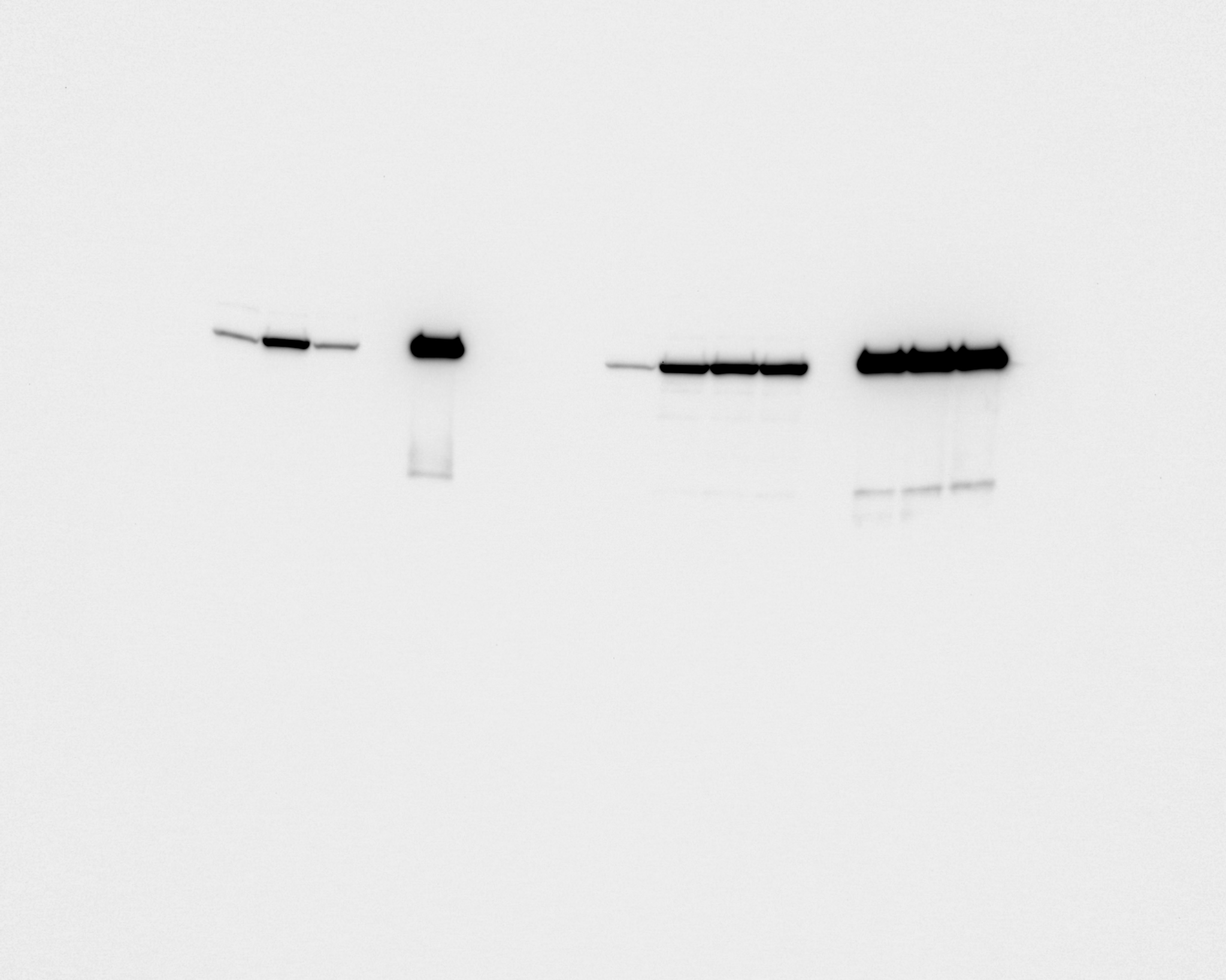

Supplement: Supplementary file 6 — Source data Fig. 4 [file 44318_2026_761_MOESM6_ESM.zip › Figure 4/4D/Repeat A/western blot HA (left 6 lanes).tif]

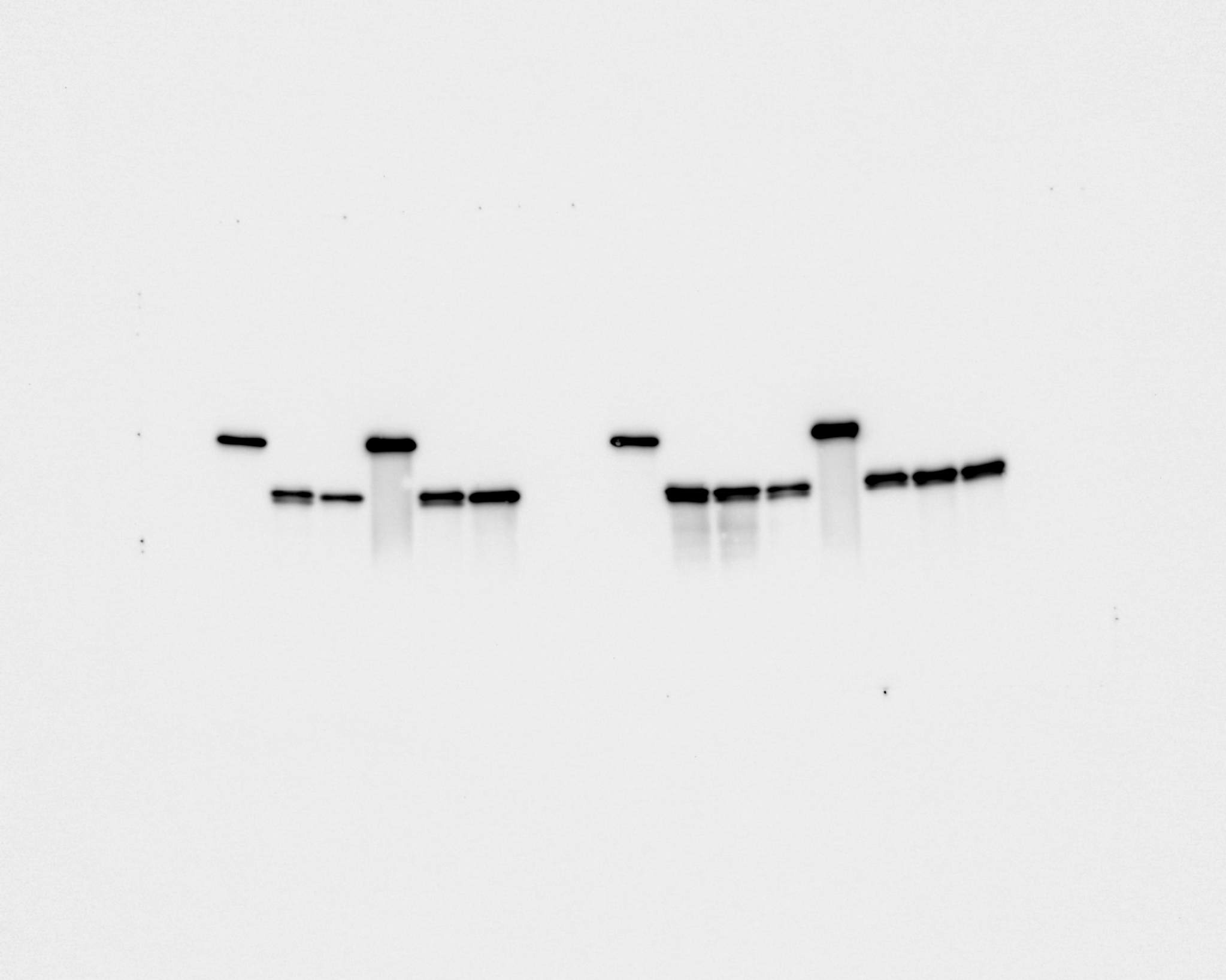

Supplement: Supplementary file 6 — Source data Fig. 4 [file 44318_2026_761_MOESM6_ESM.zip › Figure 4/4D/Repeat A/western blot V5 (left 6 lanes).tif]

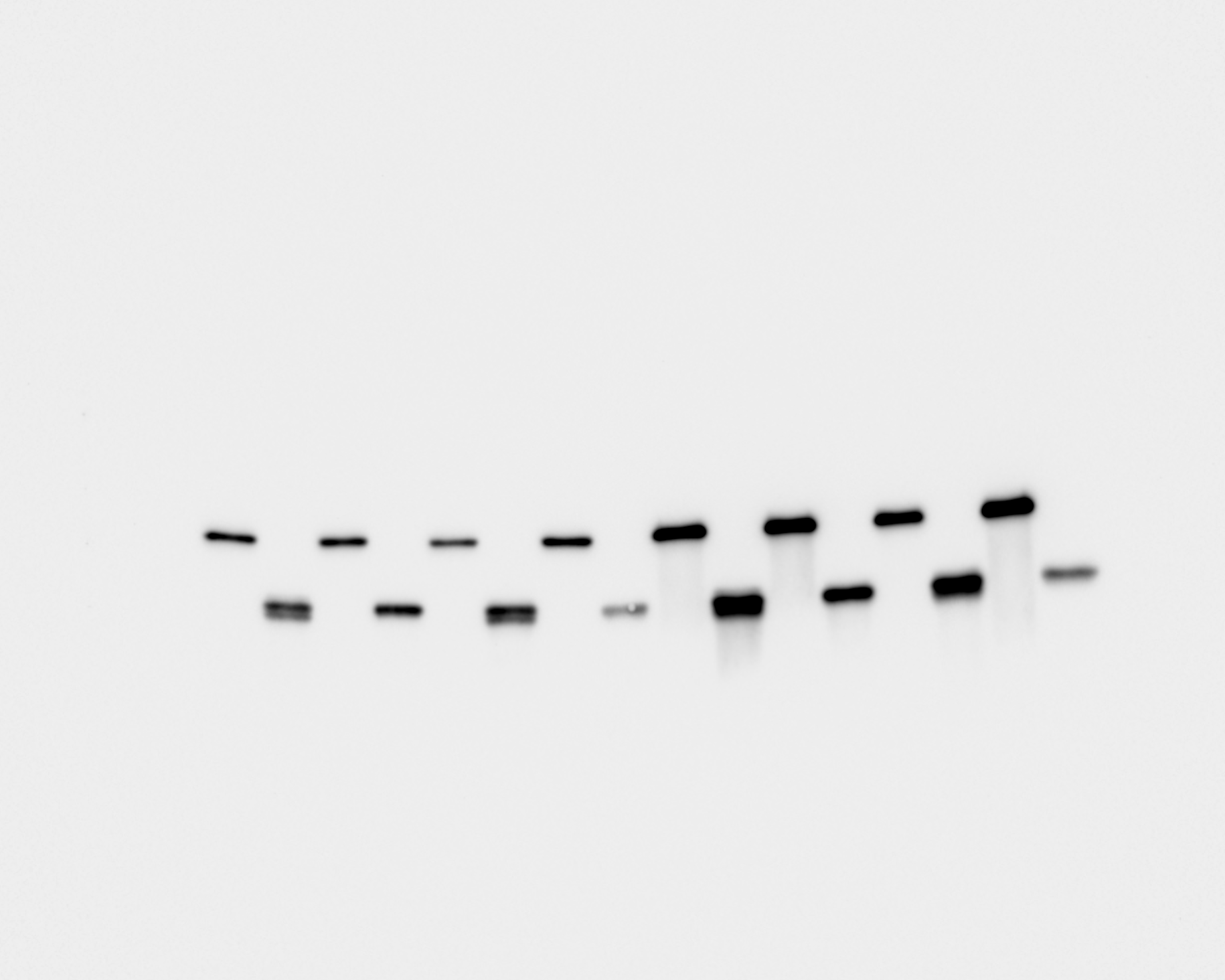

Supplement: Supplementary file 6 — Source data Fig. 4 [file 44318_2026_761_MOESM6_ESM.zip › Figure 4/4G/western blot V5.tif]

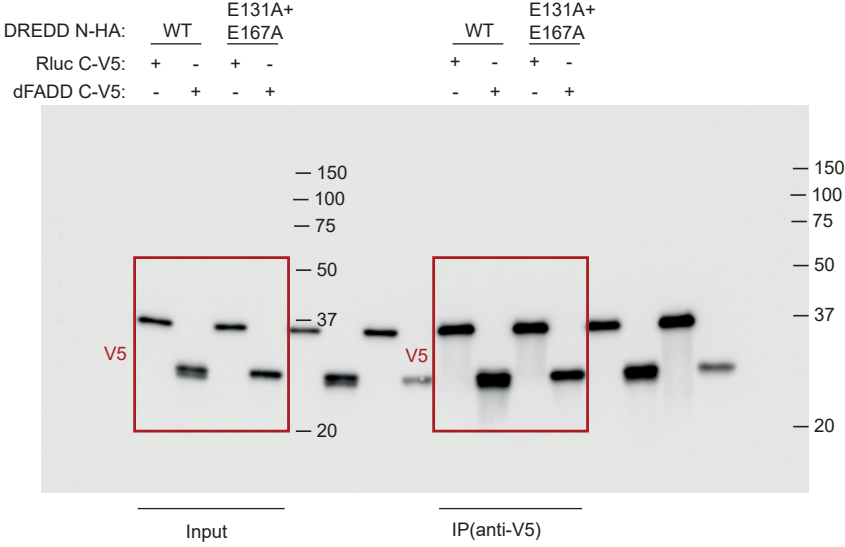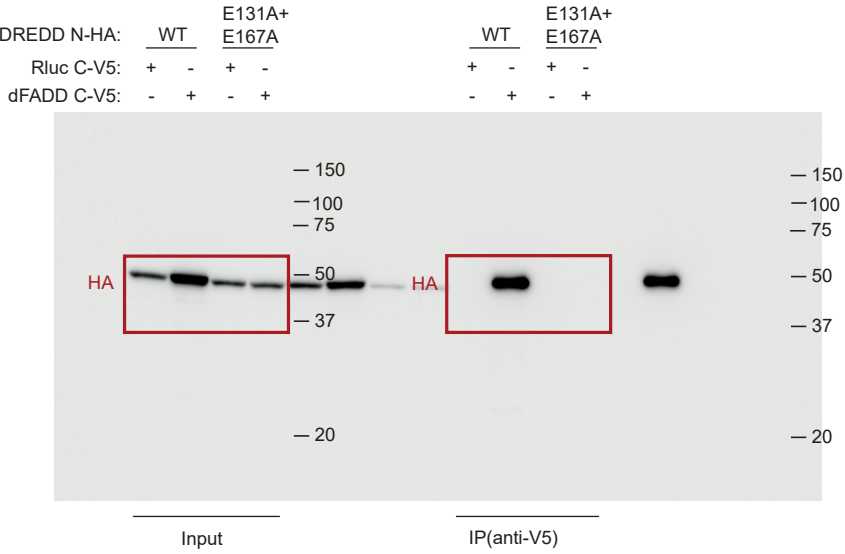

Supplement: Supplementary file 6 — Source data Fig. 4 [file 44318_2026_761_MOESM6_ESM.zip › Figure 4/4G/Annotation.pdf]

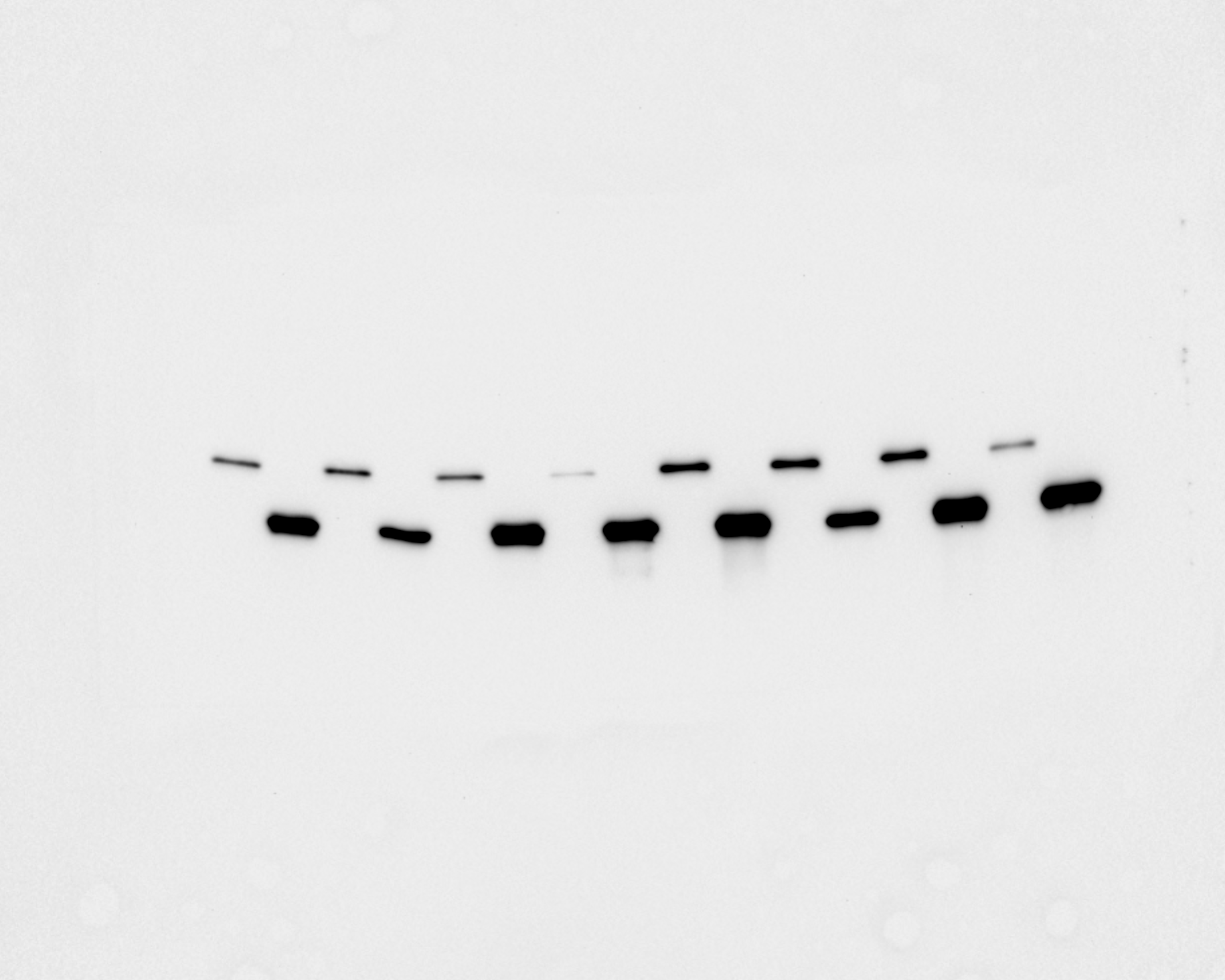

Supplement: Supplementary file 6 — Source data Fig. 4 [file 44318_2026_761_MOESM6_ESM.zip › Figure 4/4G/Repeat B/western blot V5.tif]

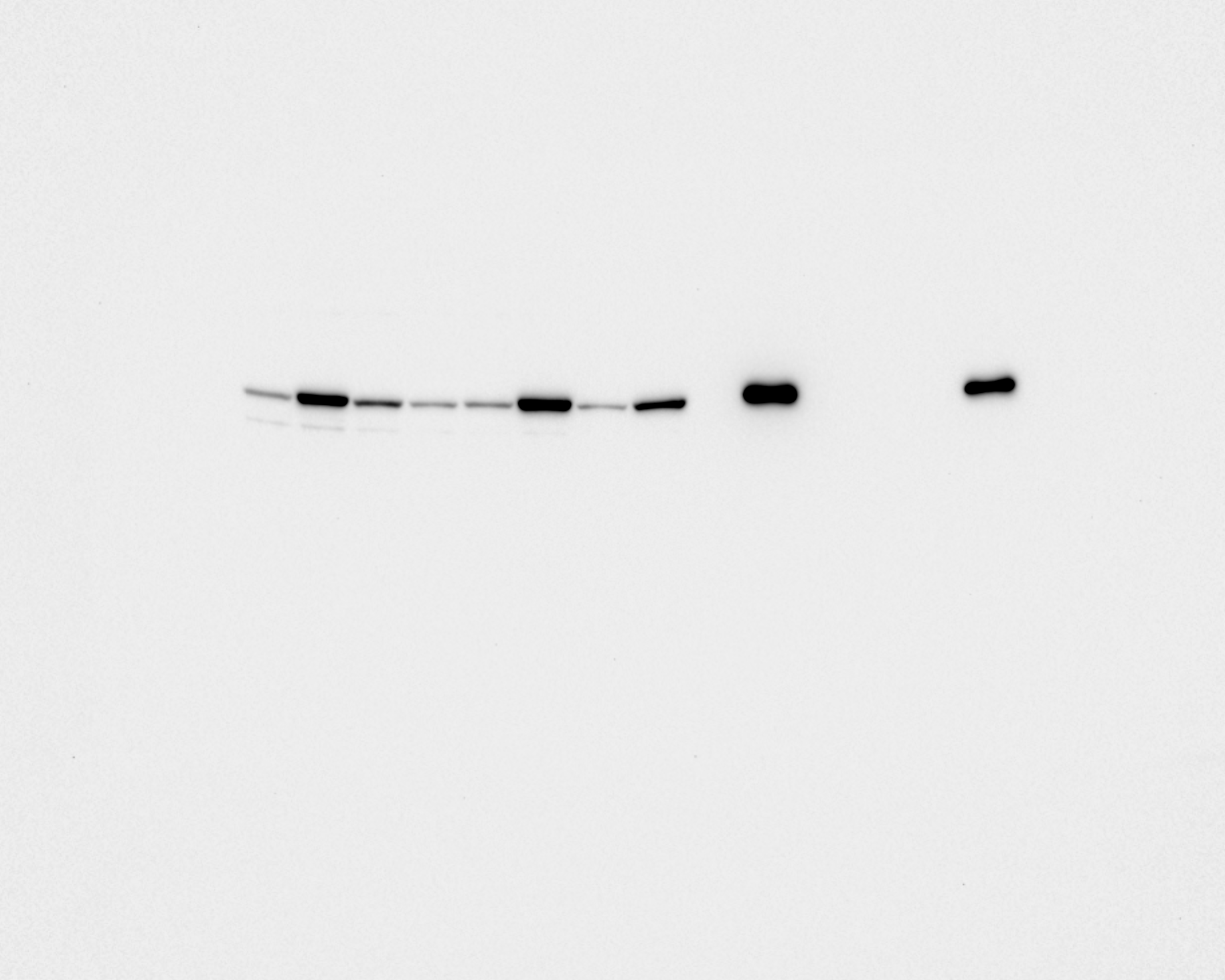

Supplement: Supplementary file 6 — Source data Fig. 4 [file 44318_2026_761_MOESM6_ESM.zip › Figure 4/4G/Repeat B/western blot HA.tif]

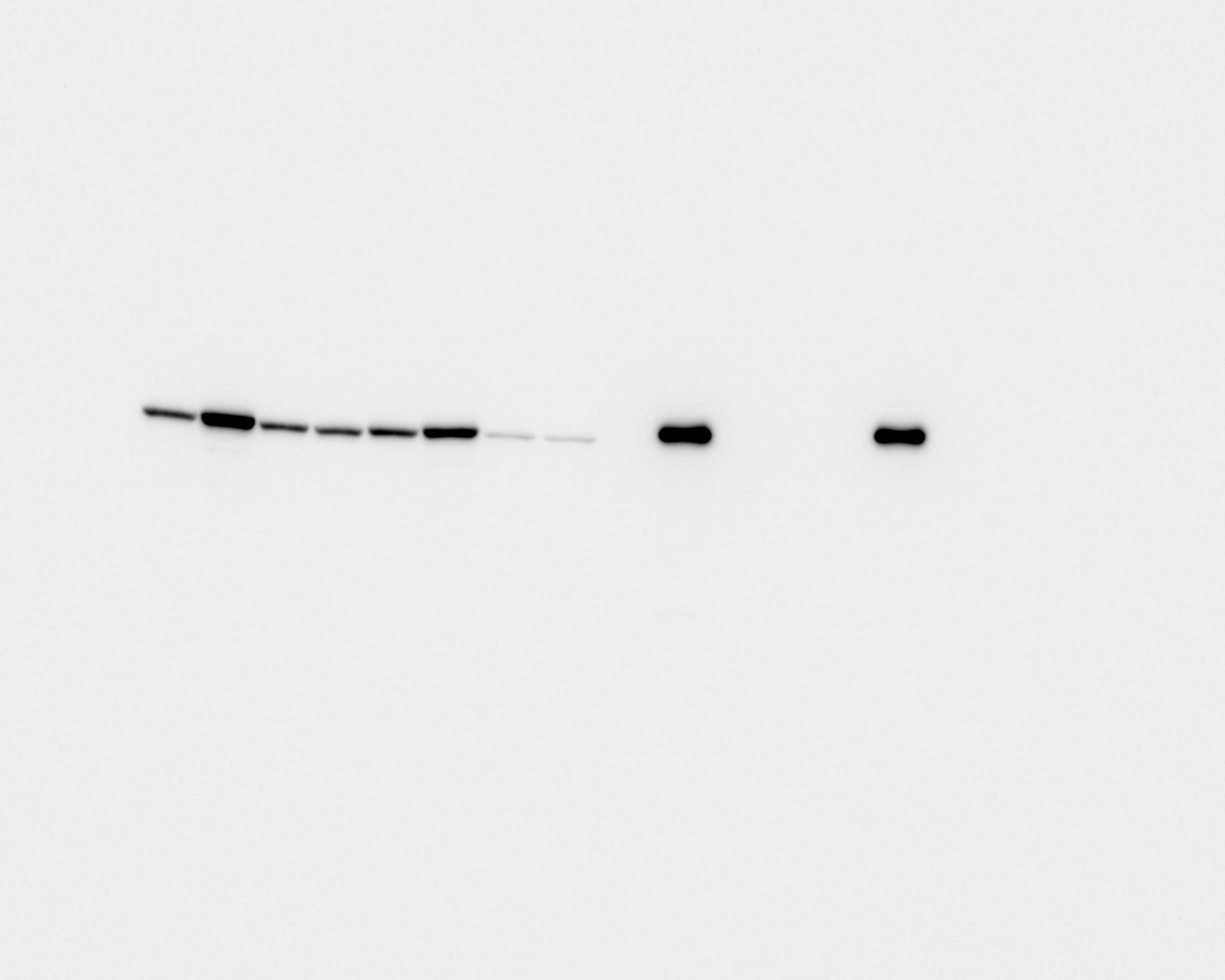

Supplement: Supplementary file 6 — Source data Fig. 4 [file 44318_2026_761_MOESM6_ESM.zip › Figure 4/4G/western blot HA.tif]

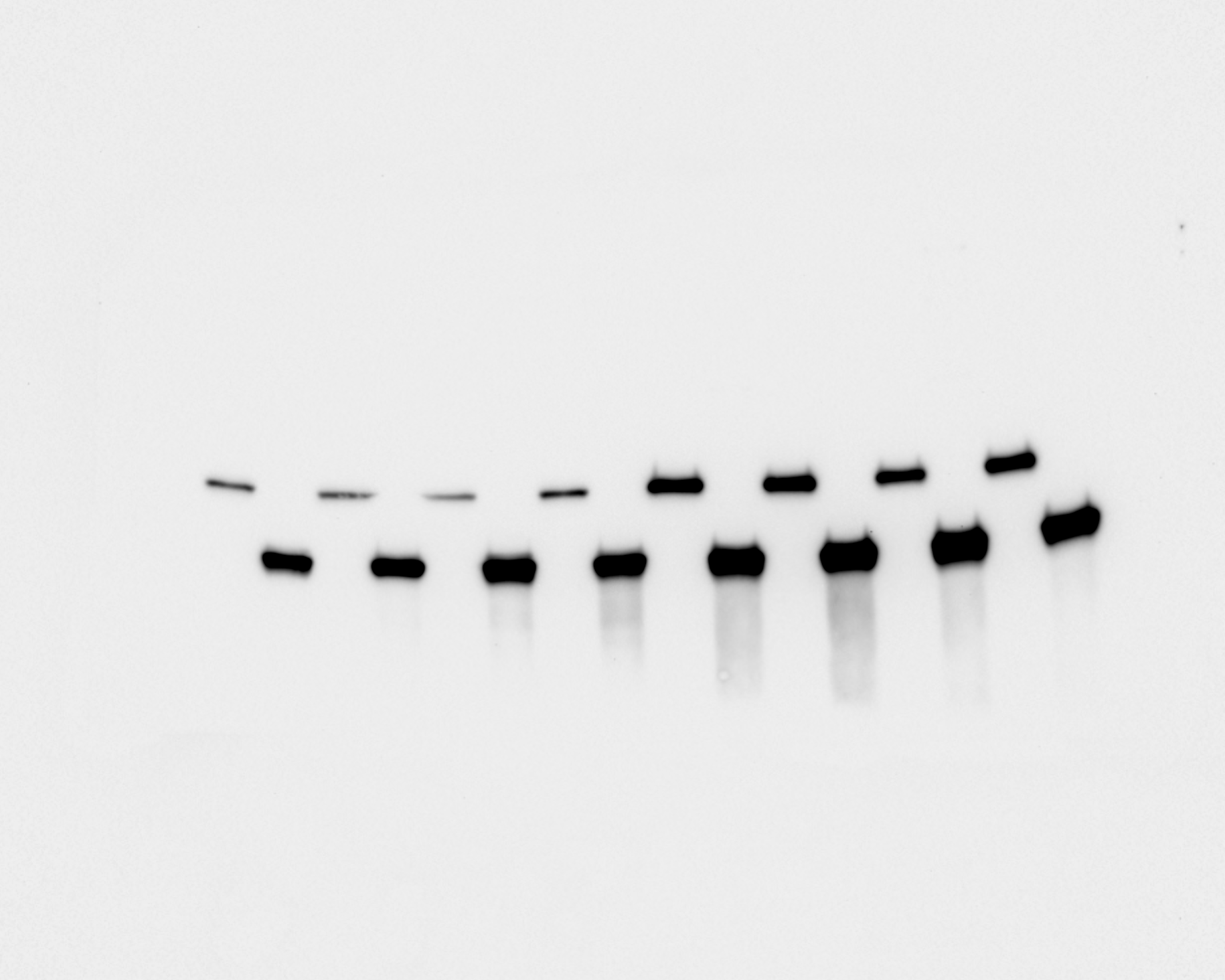

Supplement: Supplementary file 6 — Source data Fig. 4 [file 44318_2026_761_MOESM6_ESM.zip › Figure 4/4G/Repeat A/western blot V5.tif]

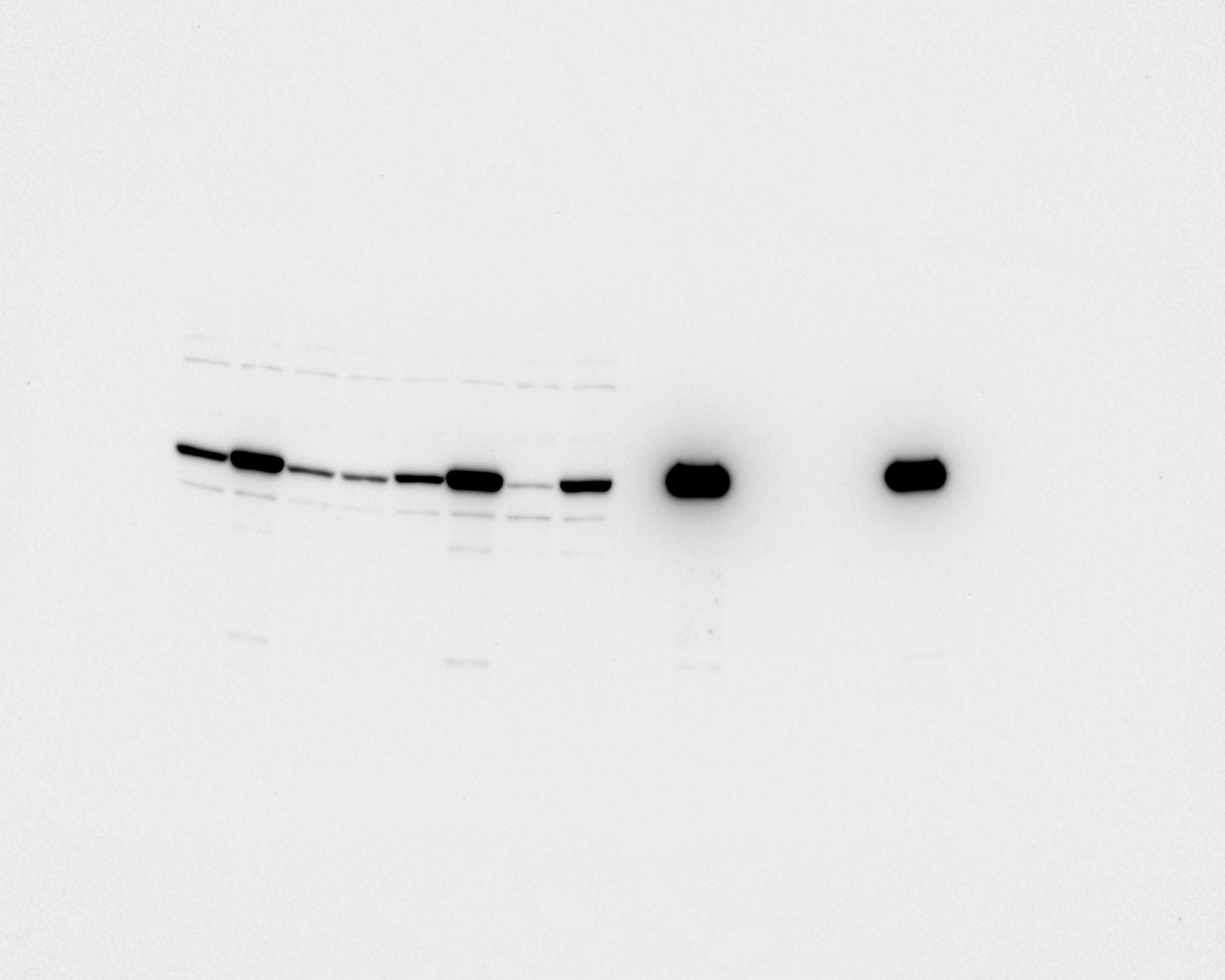

Supplement: Supplementary file 6 — Source data Fig. 4 [file 44318_2026_761_MOESM6_ESM.zip › Figure 4/4G/Repeat A/western blot HA.tif]

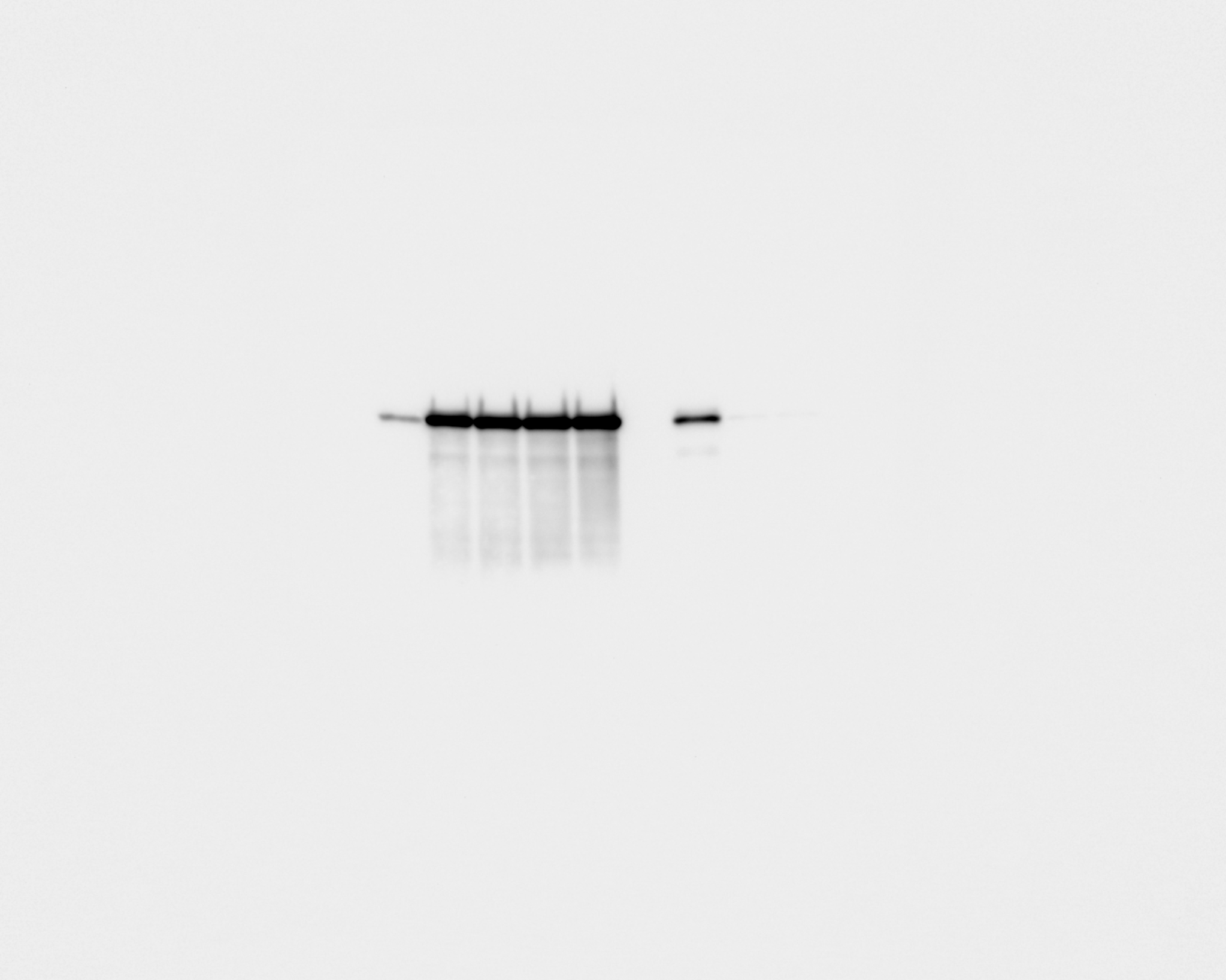

Supplement: Supplementary file 7 — Source data Fig. 5 [file 44318_2026_761_MOESM7_ESM.zip › Figure 5/5C/western blot V5.tif]

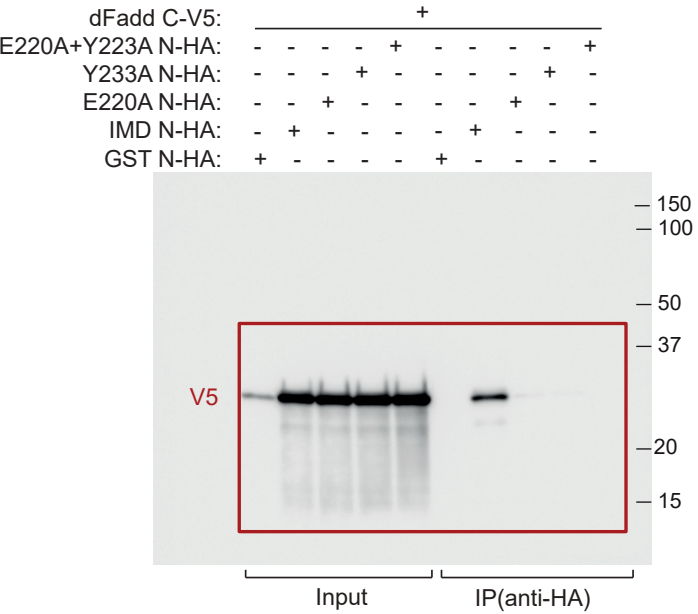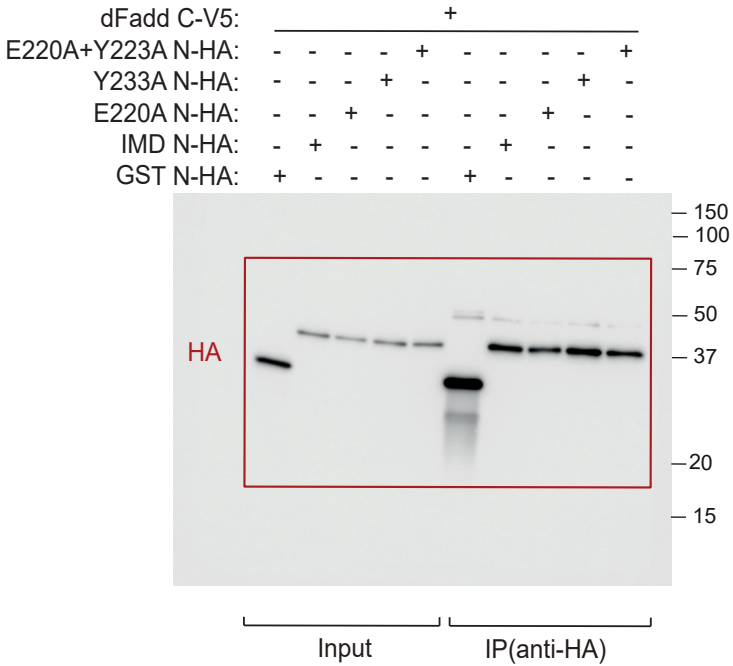

Supplement: Supplementary file 7 — Source data Fig. 5 [file 44318_2026_761_MOESM7_ESM.zip › Figure 5/5C/Annotation.pdf]

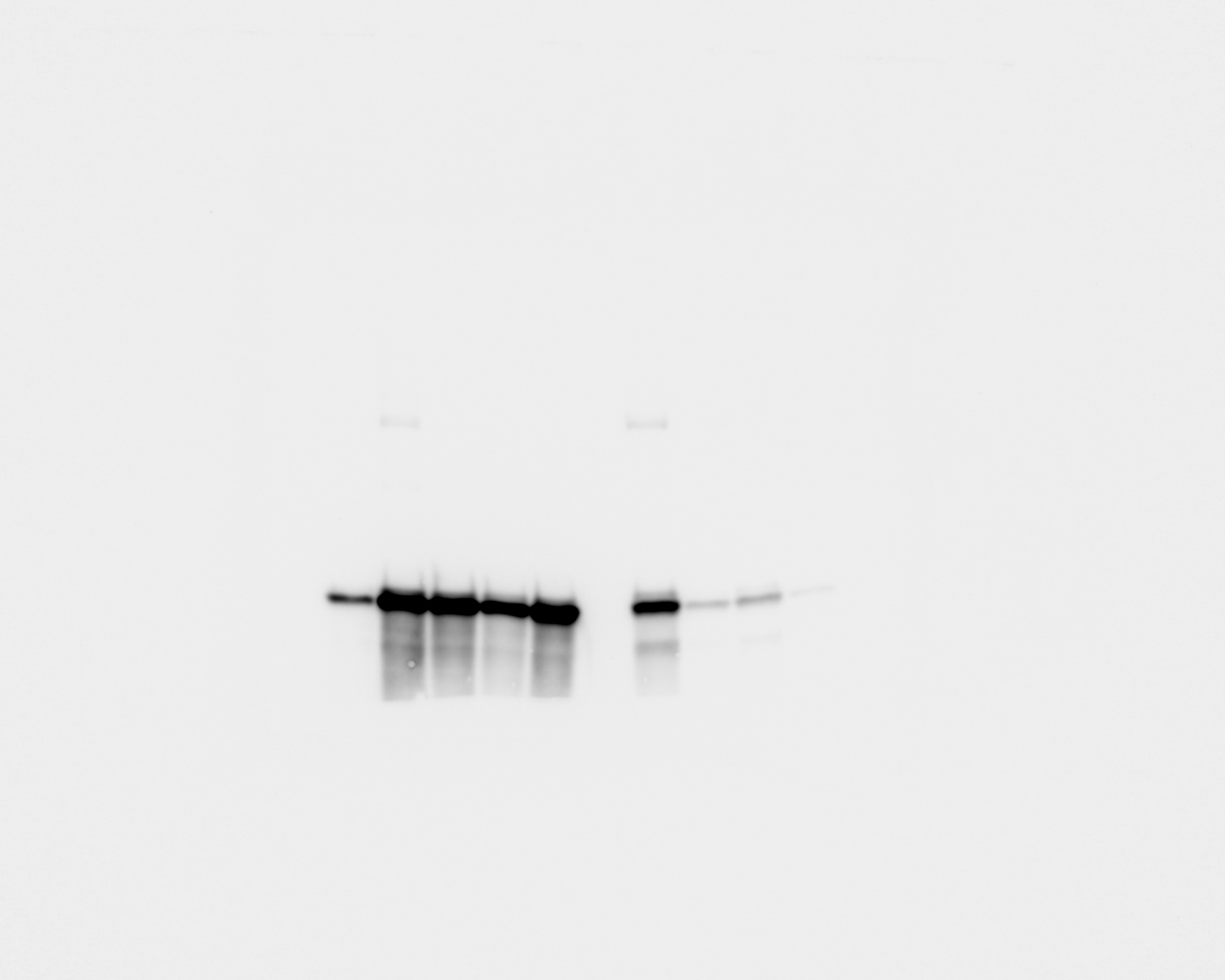

Supplement: Supplementary file 7 — Source data Fig. 5 [file 44318_2026_761_MOESM7_ESM.zip › Figure 5/5C/Repeat B/western blot V5.tif]

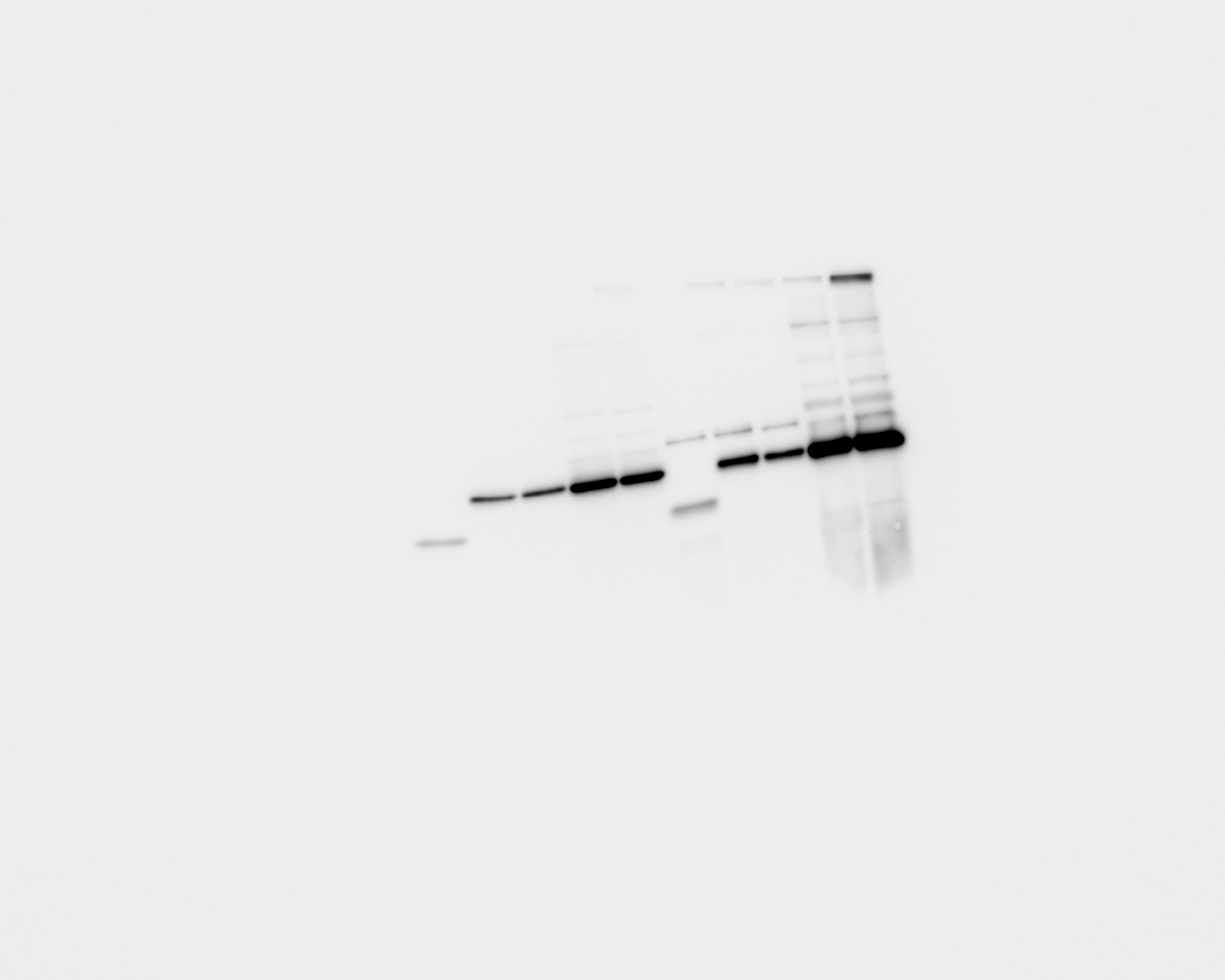

Supplement: Supplementary file 7 — Source data Fig. 5 [file 44318_2026_761_MOESM7_ESM.zip › Figure 5/5C/Repeat B/western blot HA.tif]

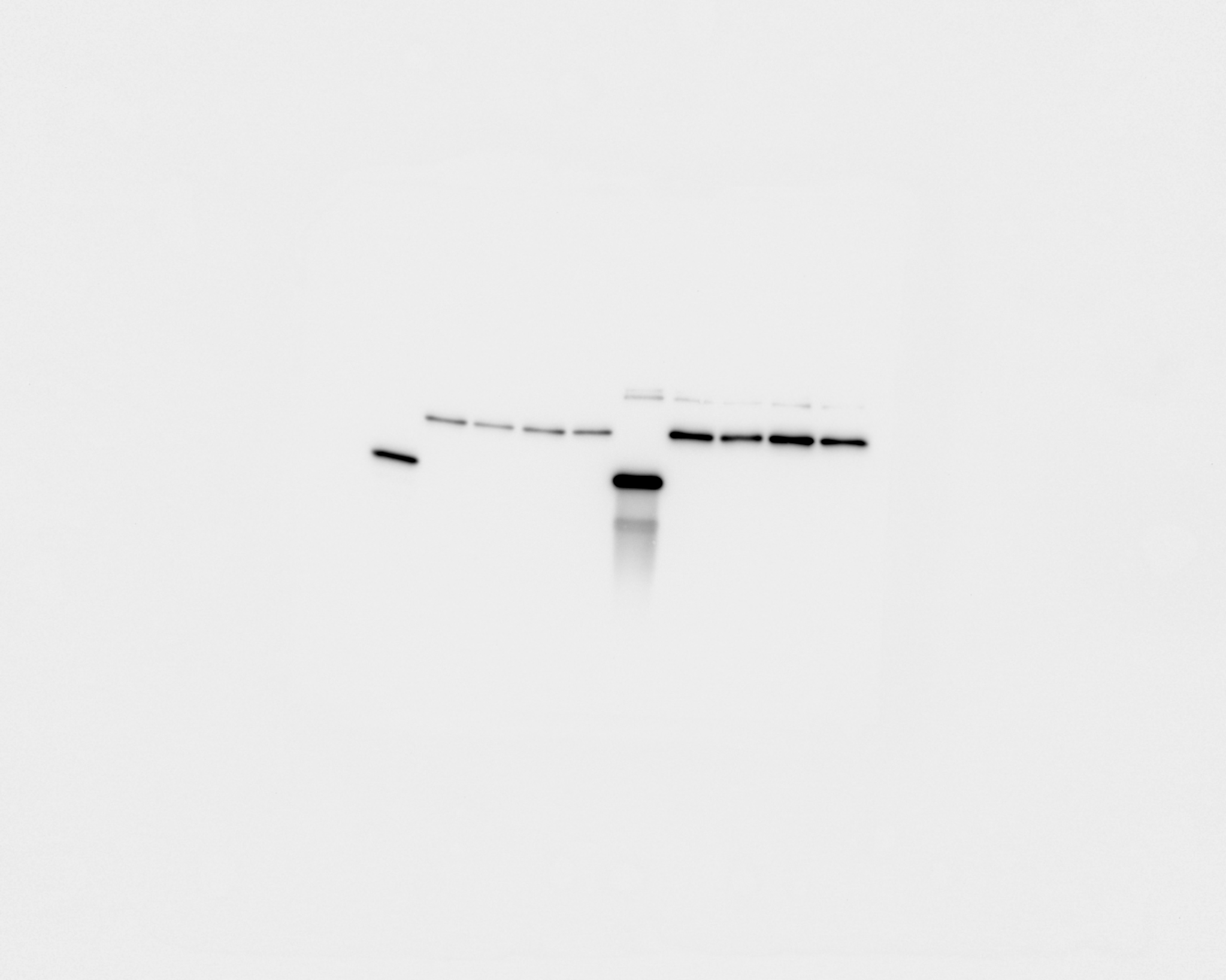

Supplement: Supplementary file 7 — Source data Fig. 5 [file 44318_2026_761_MOESM7_ESM.zip › Figure 5/5C/western blot HA.tif]

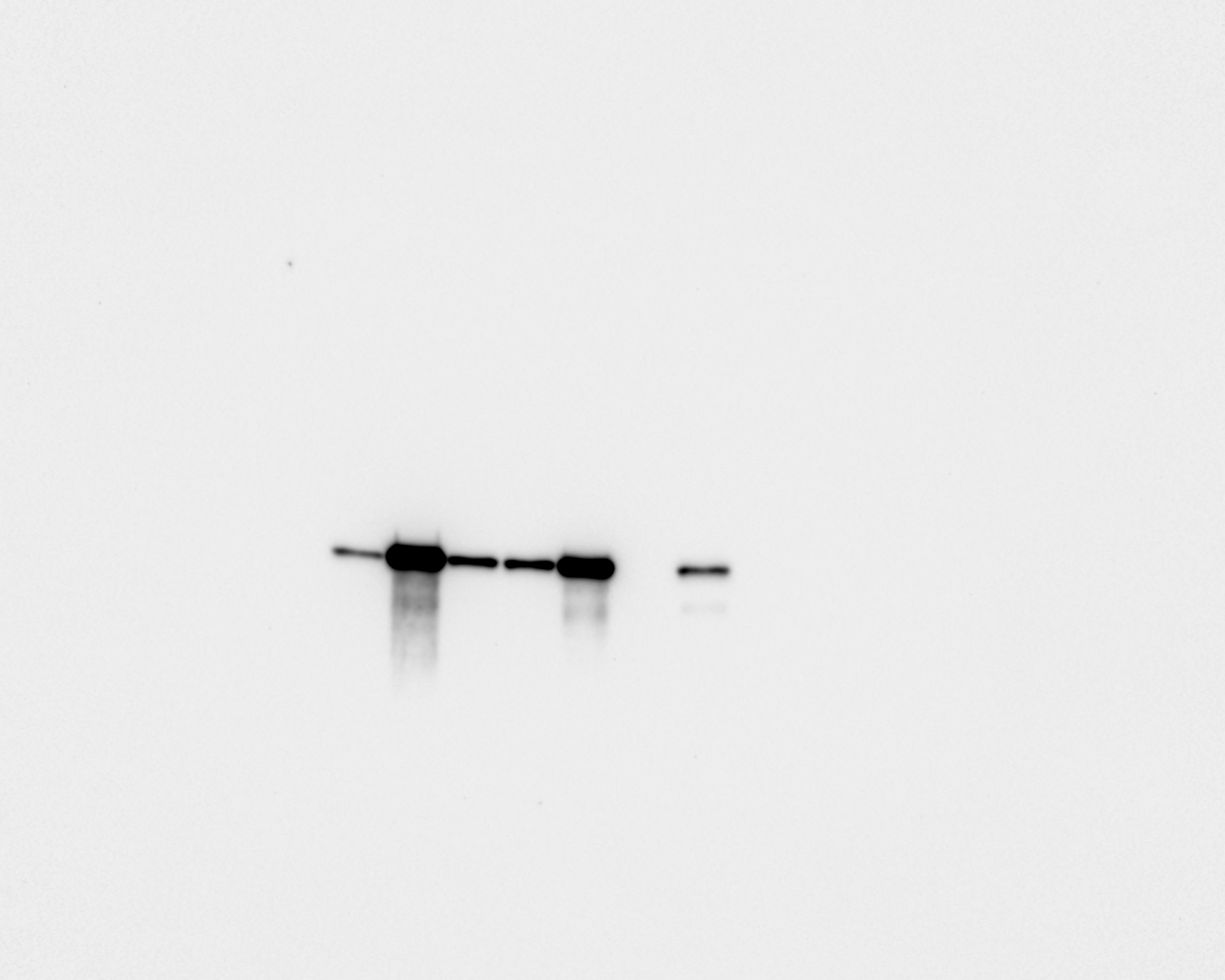

Supplement: Supplementary file 7 — Source data Fig. 5 [file 44318_2026_761_MOESM7_ESM.zip › Figure 5/5C/Repeat A/western blot V5.tif]

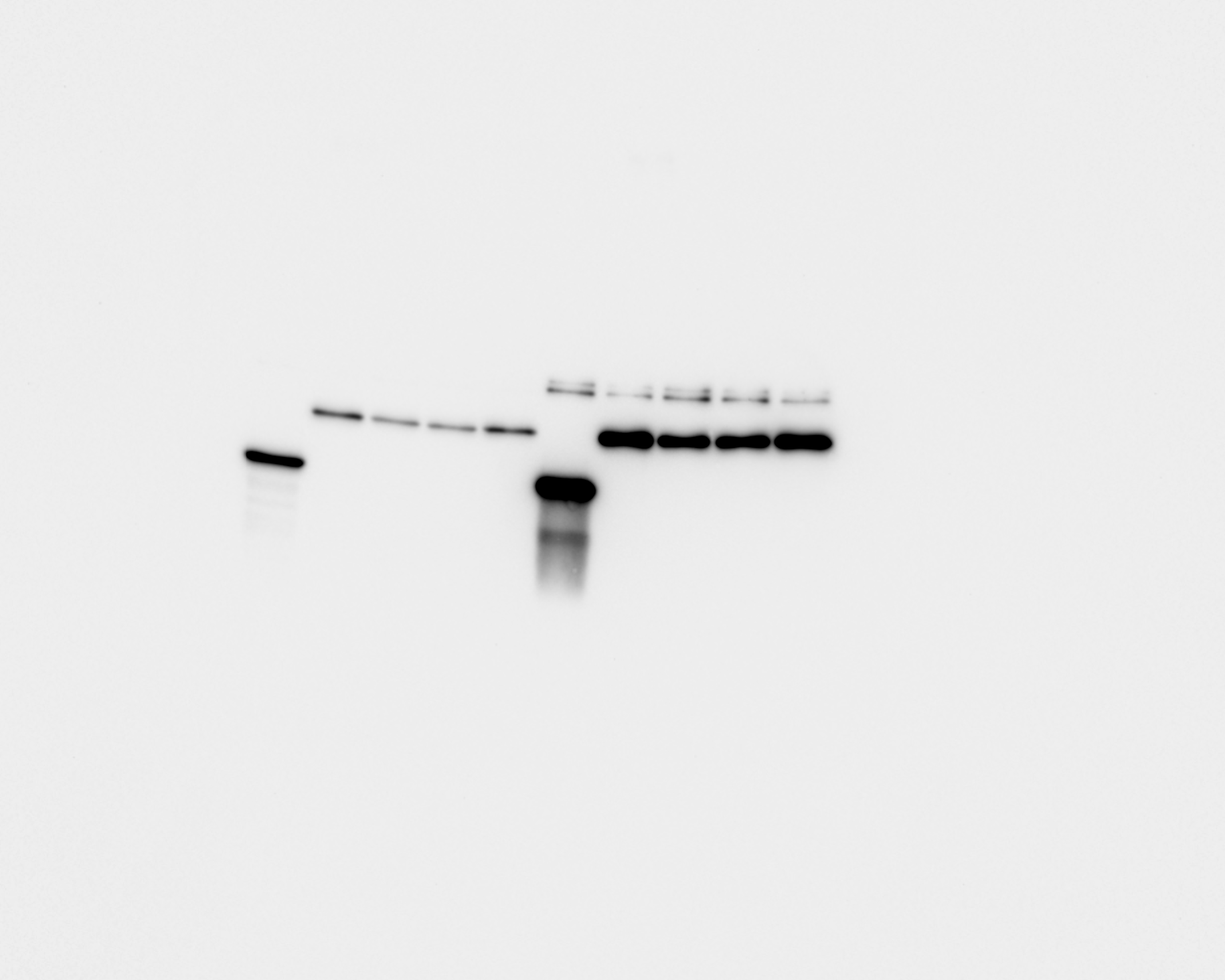

Supplement: Supplementary file 7 — Source data Fig. 5 [file 44318_2026_761_MOESM7_ESM.zip › Figure 5/5C/Repeat A/western blot HA.tif]

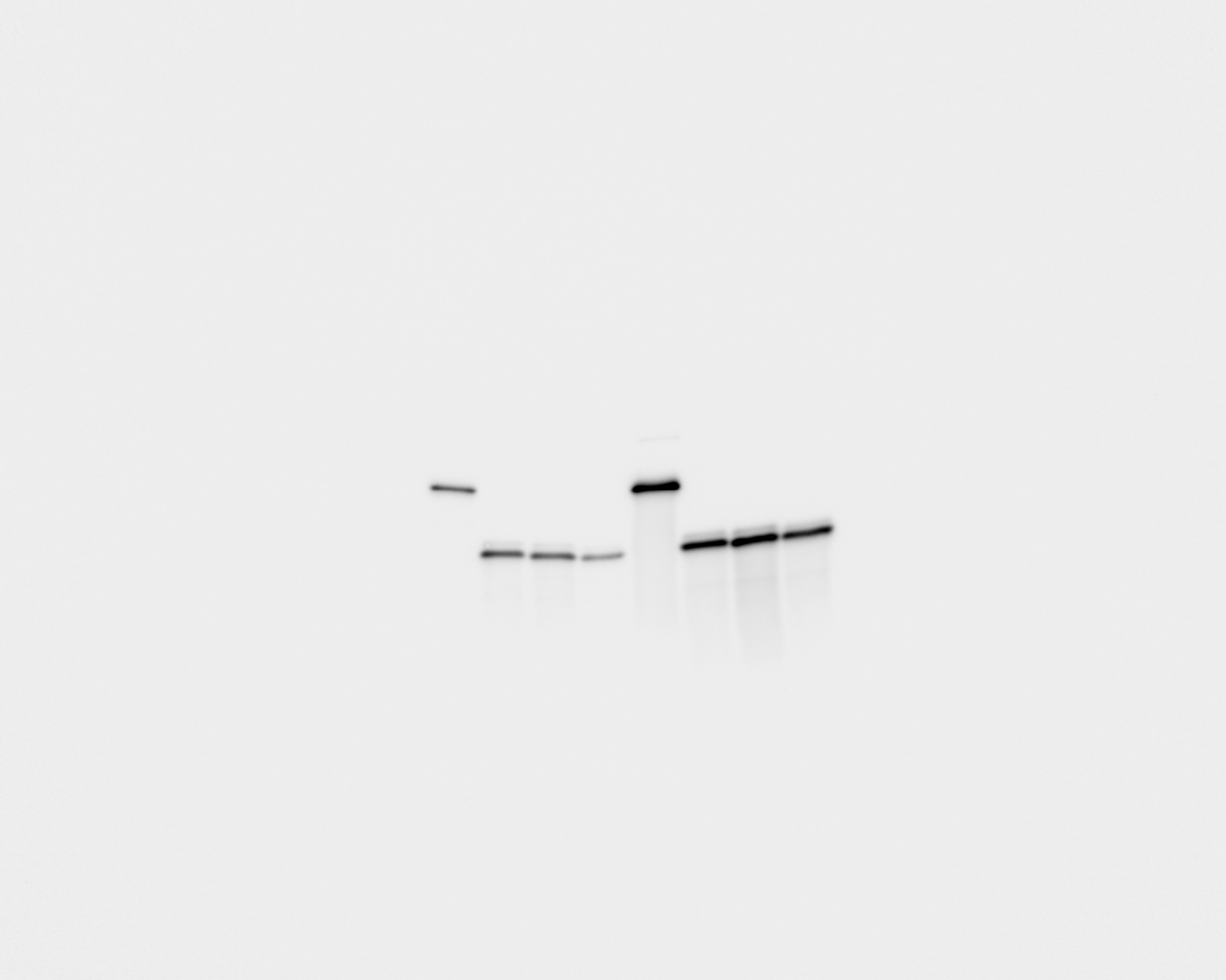

Supplement: Supplementary file 7 — Source data Fig. 5 [file 44318_2026_761_MOESM7_ESM.zip › Figure 5/5E/western blot V5.tif]

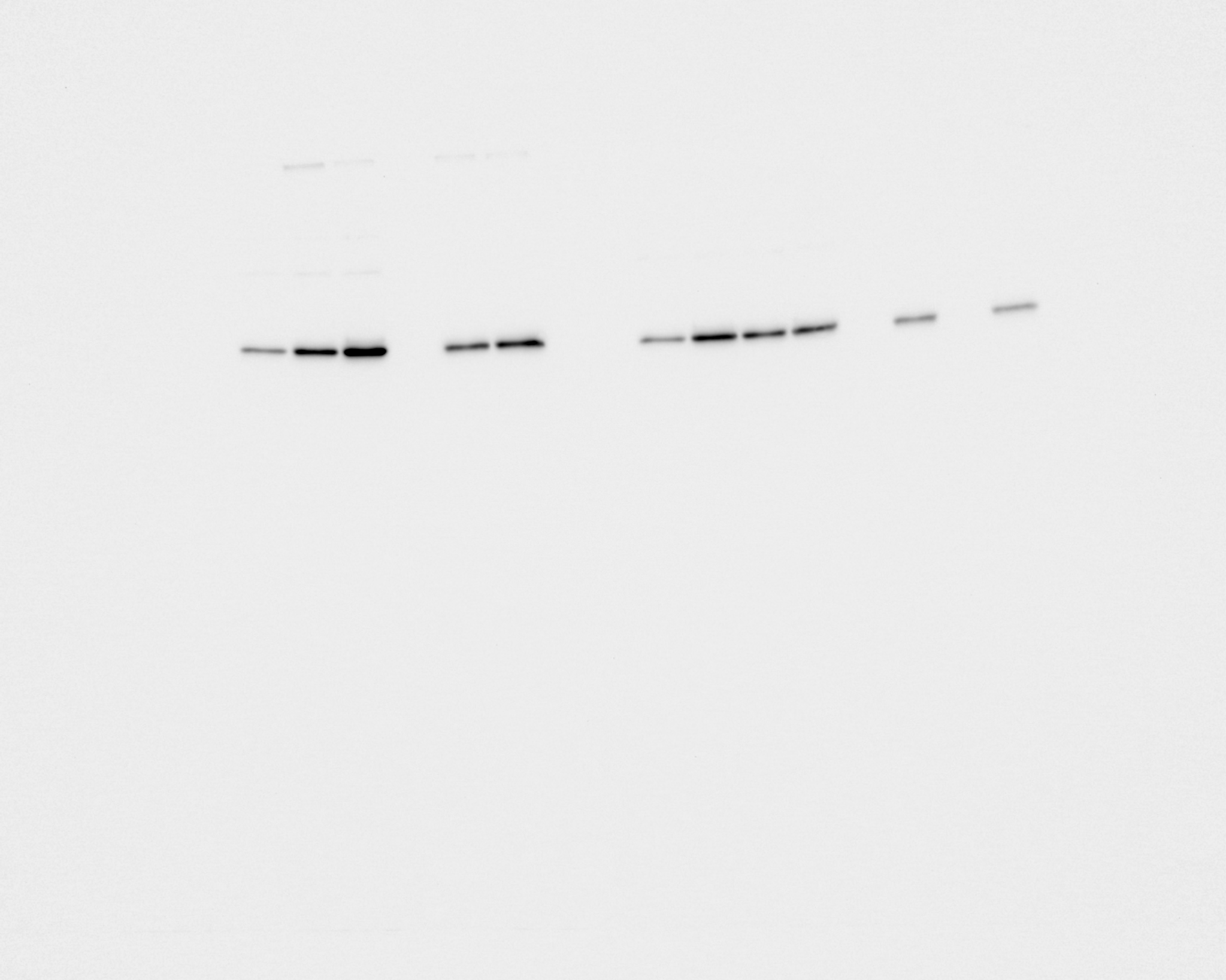

Supplement: Supplementary file 7 — Source data Fig. 5 [file 44318_2026_761_MOESM7_ESM.zip › Figure 5/5E/Repeat B/western blot HA (right 8 lanes).tif]

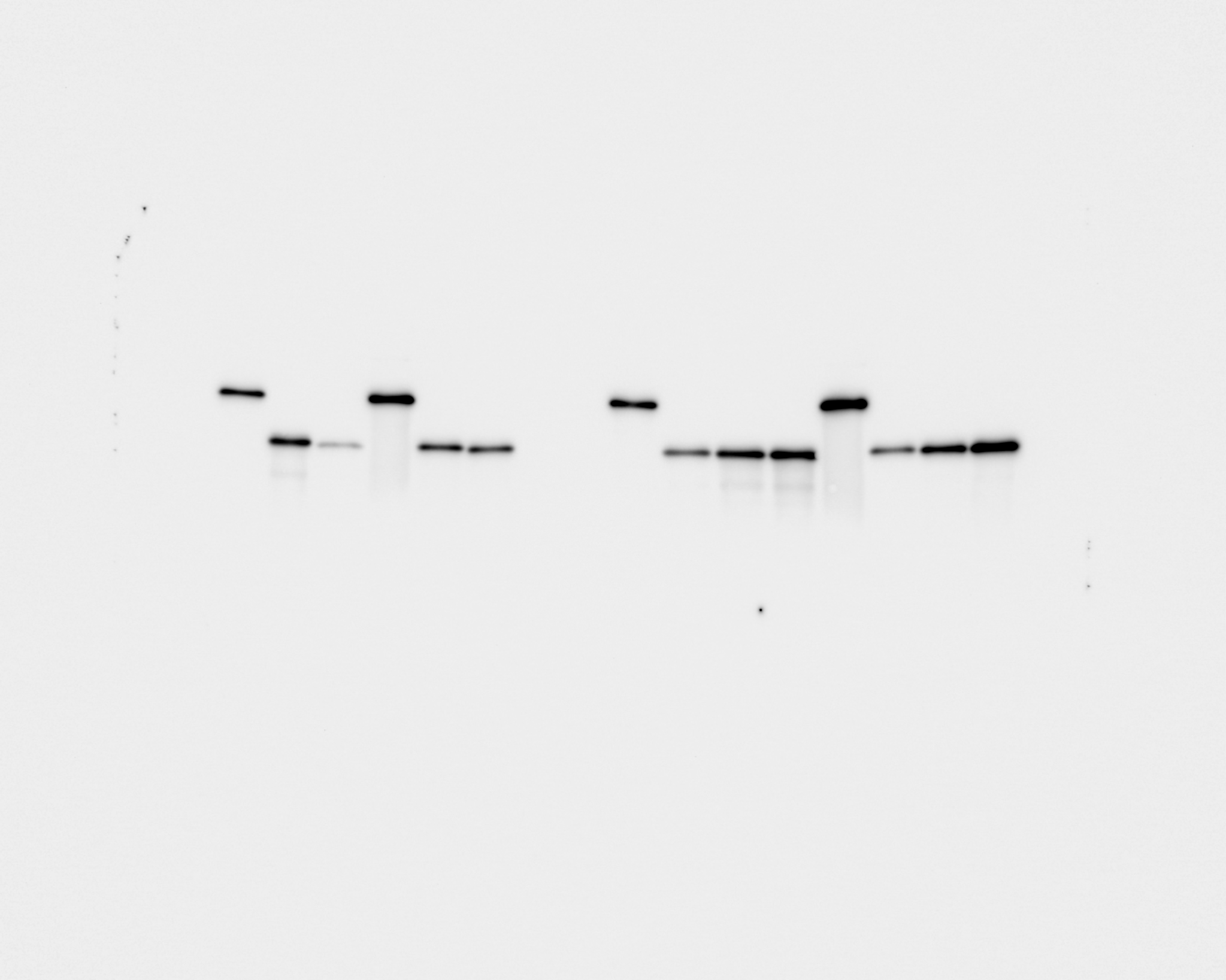

Supplement: Supplementary file 7 — Source data Fig. 5 [file 44318_2026_761_MOESM7_ESM.zip › Figure 5/5E/Repeat B/western blot V5 (right 8 lanes).tif]

|             |   |   |   |   |   |   |   |   |
|-------------|---|---|---|---|---|---|---|---|
| HA-IMD:     | + |   |   |   |   |   |   |   |
| E177A C-V5: | - | - | - | + | - | - | - | + |
| R171A C-V5: | - | - | + | - | - | - | + | - |
| dFADD C-V5: | - | + | - | - | - | + | - | - |
| RLuc C-V5:  | + | - | - | - | + | - | - | - |

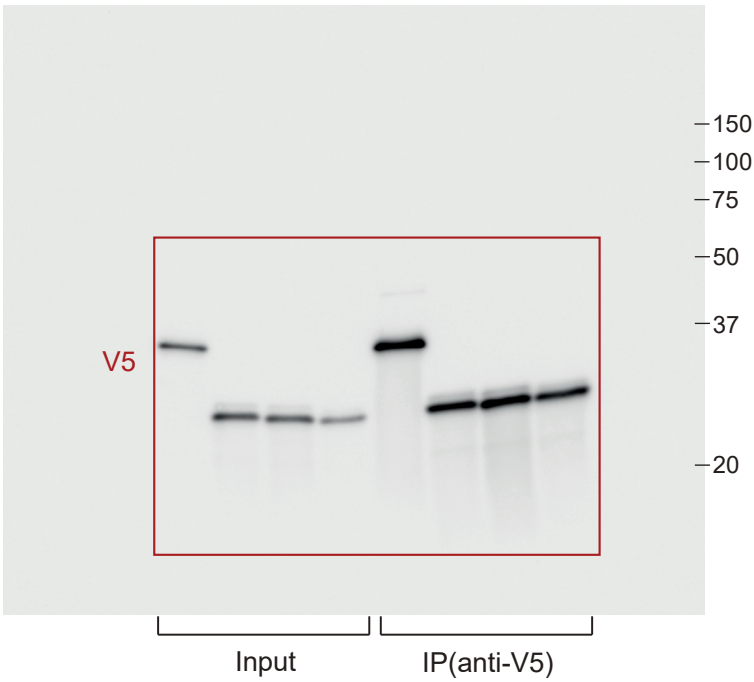

|             |   |   |   |   |   |   |   |   |
|-------------|---|---|---|---|---|---|---|---|
| HA-IMD:     | + |   |   |   |   |   |   |   |
| E177A C-V5: | - | - | - | + | - | - | - | + |
| R171A C-V5: | - | - | + | - | - | - | + | - |
| dFADD C-V5: | - | + | - | - | - | + | - | - |
| RLuc C-V5:  | + | - | - | - | + | - | - | - |

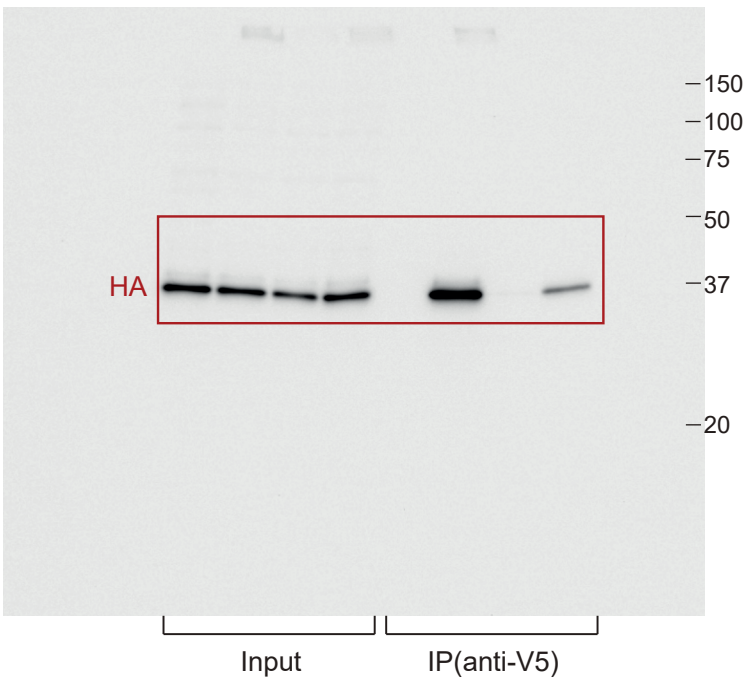

Supplement: Supplementary file 7 — Source data Fig. 5 [file 44318_2026_761_MOESM7_ESM.zip › Figure 5/5E/Annotations.pdf]

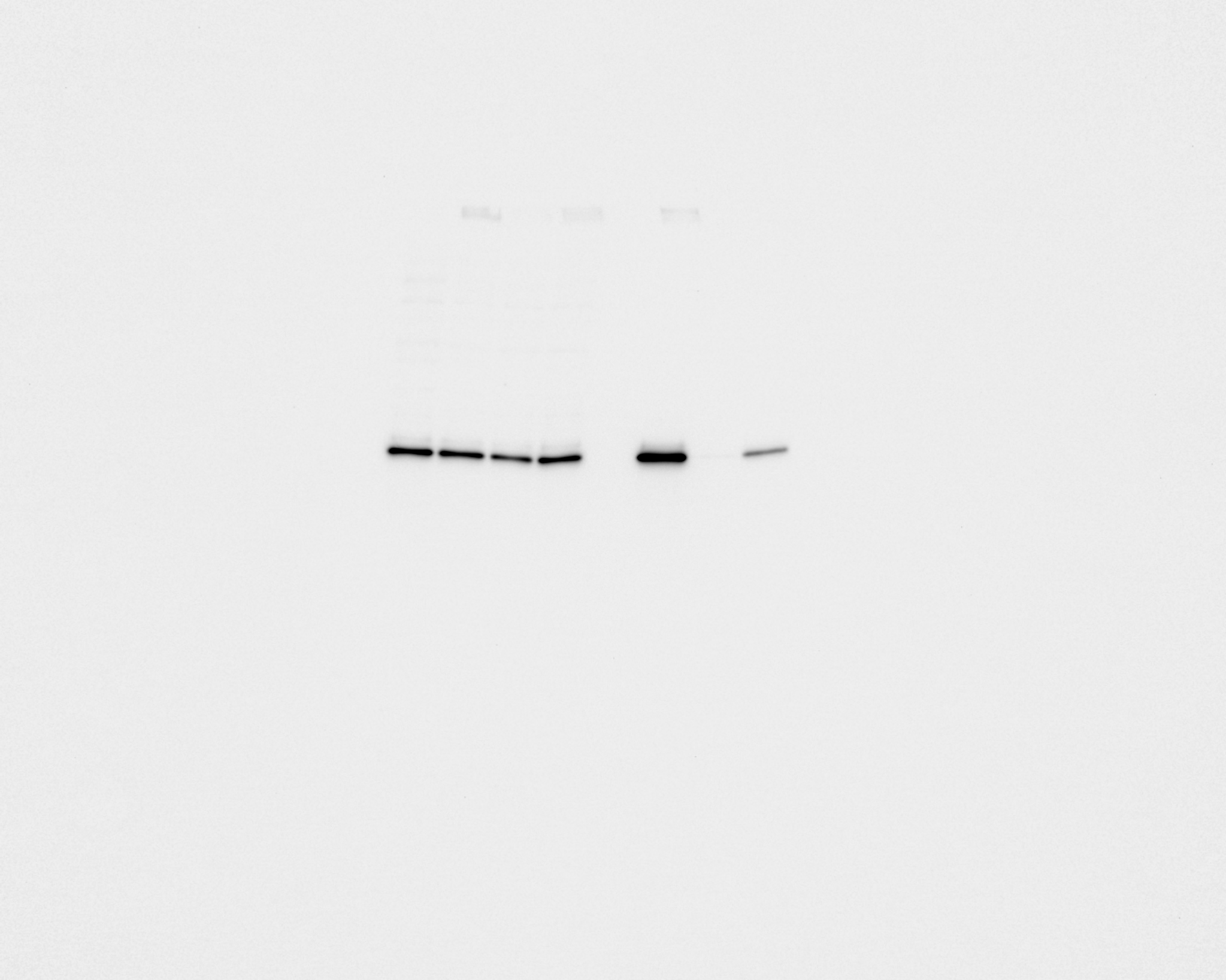

Supplement: Supplementary file 7 — Source data Fig. 5 [file 44318_2026_761_MOESM7_ESM.zip › Figure 5/5E/western blot HA.tif]

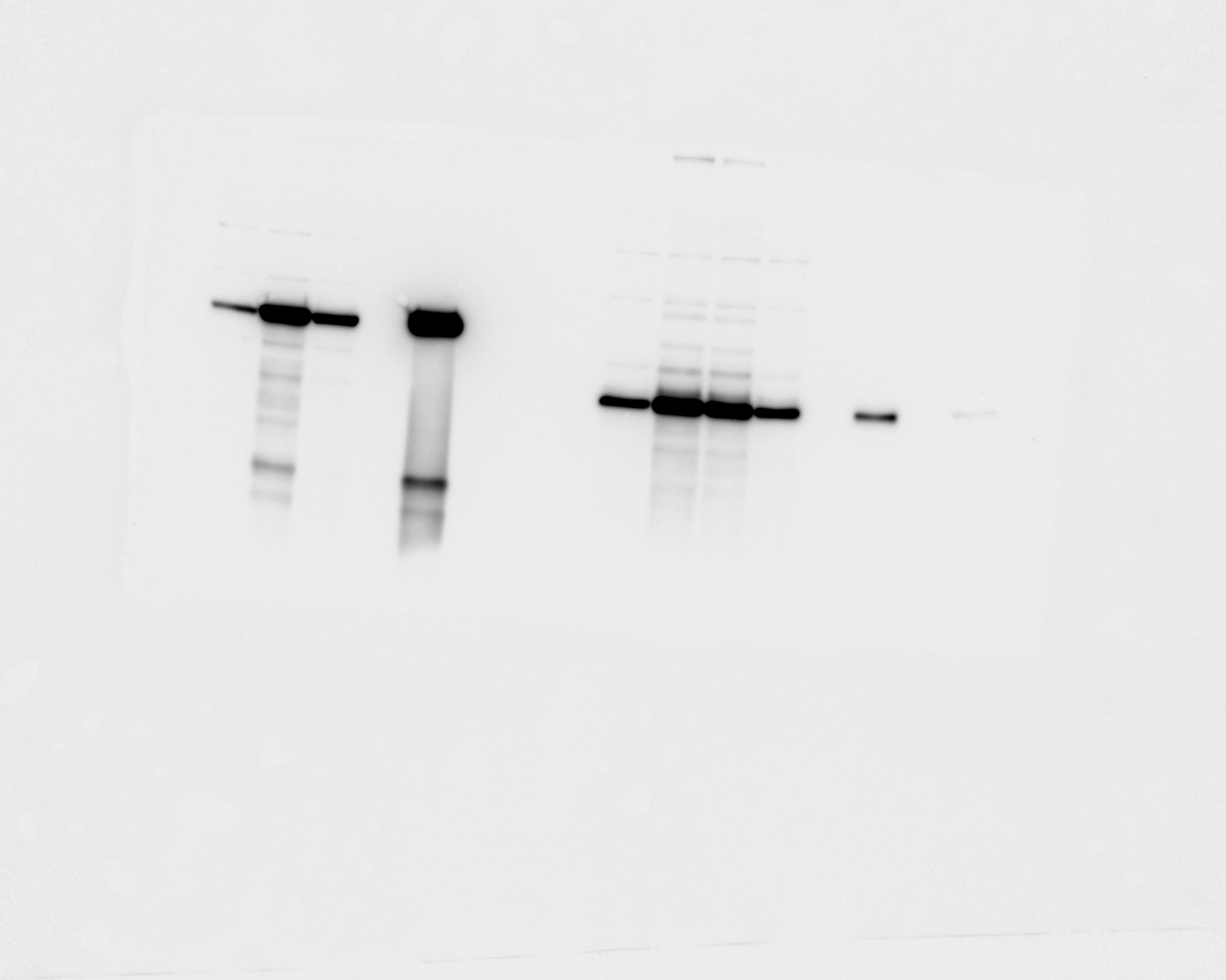

Supplement: Supplementary file 7 — Source data Fig. 5 [file 44318_2026_761_MOESM7_ESM.zip › Figure 5/5E/Repeat A/western blot HA (right 8 lanes).tif]

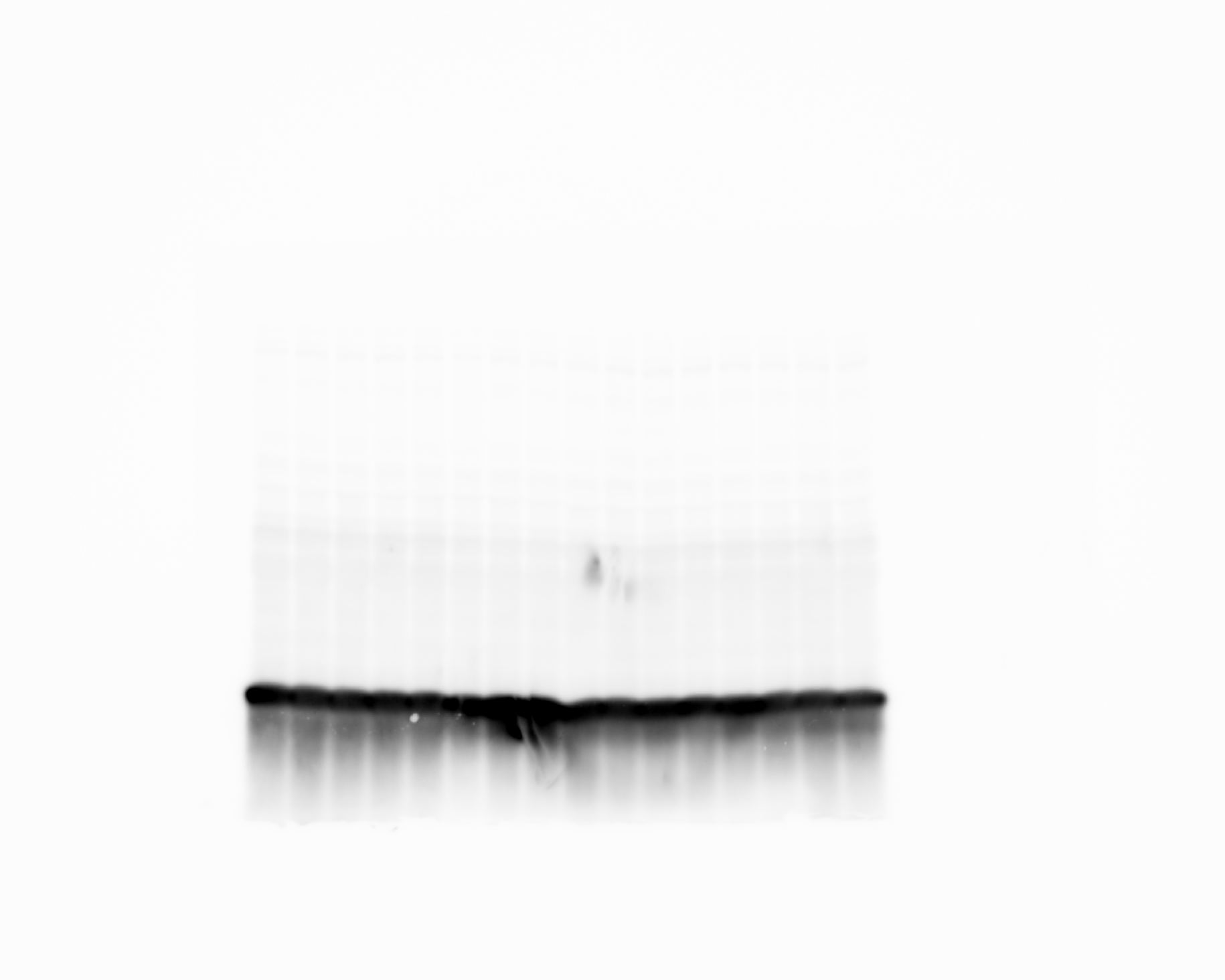

Supplement: Supplementary file 8 — Figure Source Data all EV figs [file 44318_2026_761_MOESM8_ESM.zip › EV Figures/EV1B/Western blot histone H3.tif]

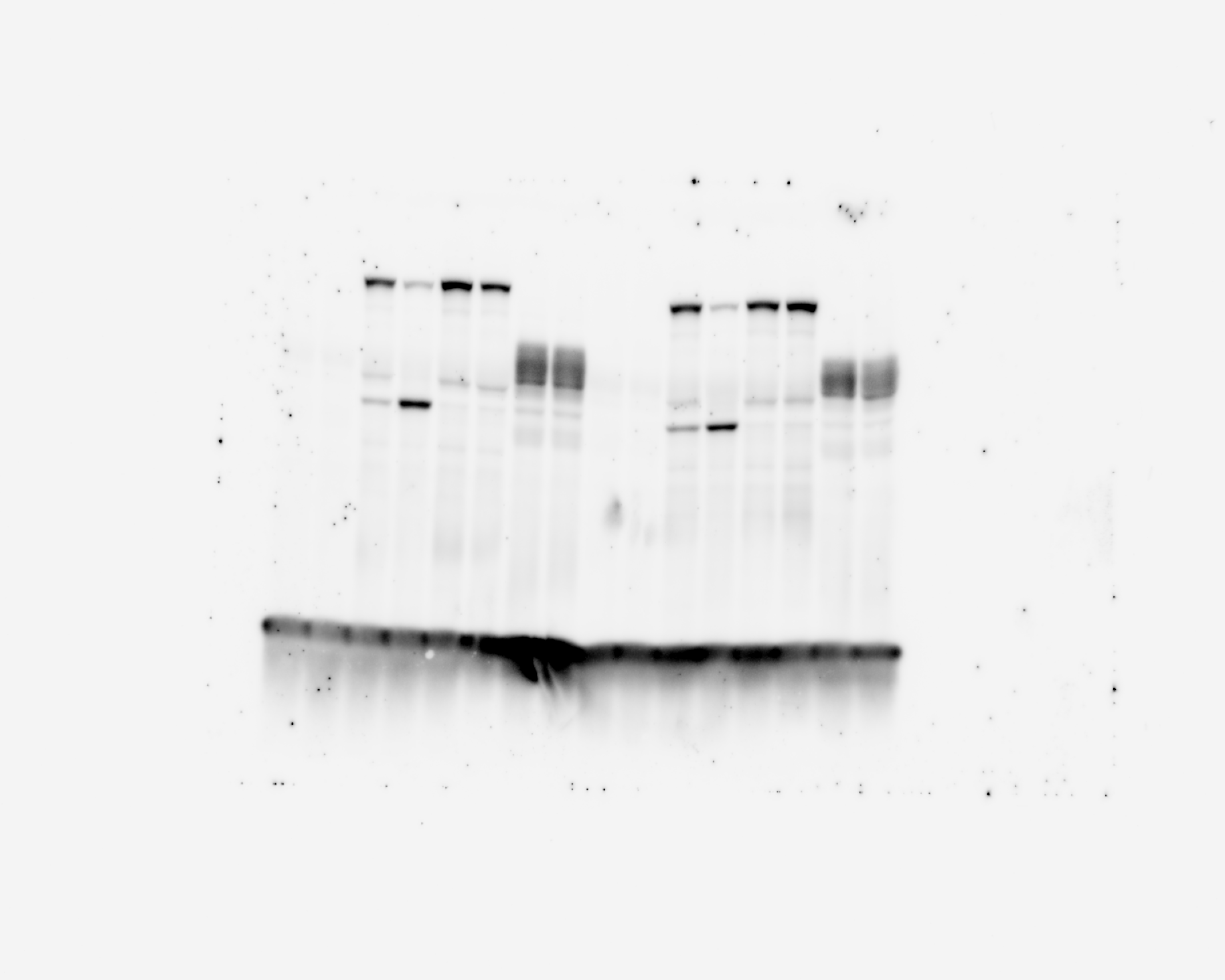

Supplement: Supplementary file 8 — Figure Source Data all EV figs [file 44318_2026_761_MOESM8_ESM.zip › EV Figures/EV1B/Western blot V5.tif]

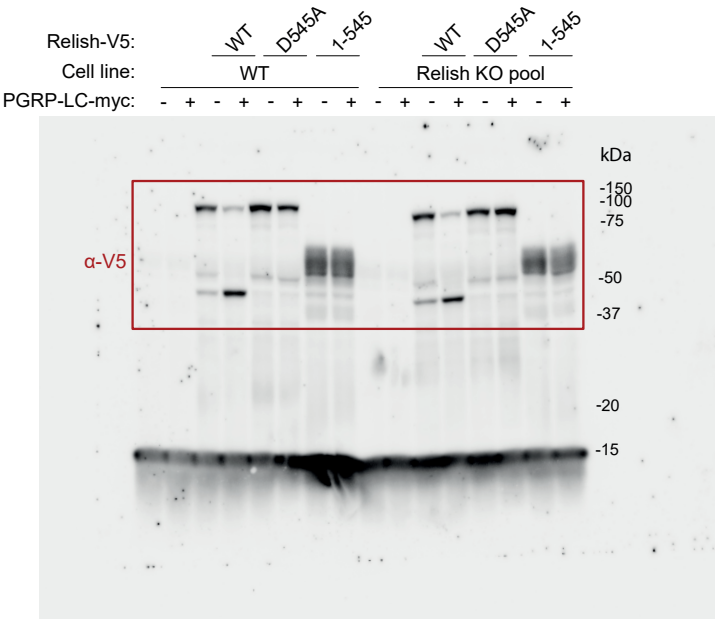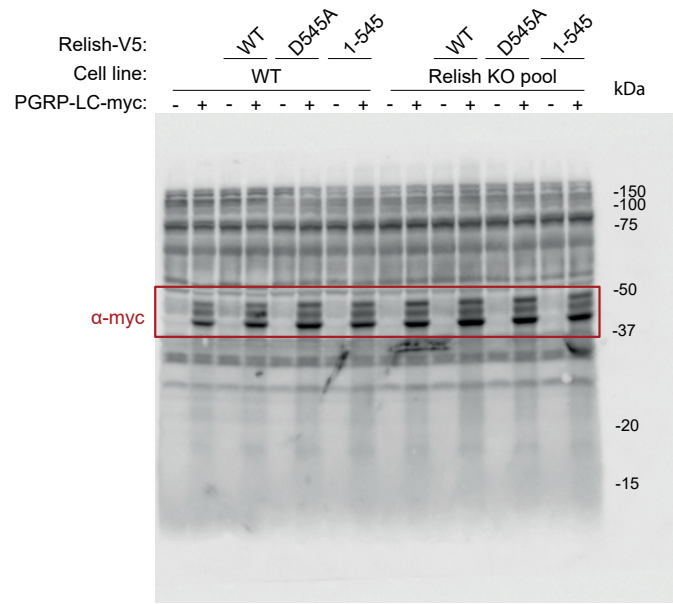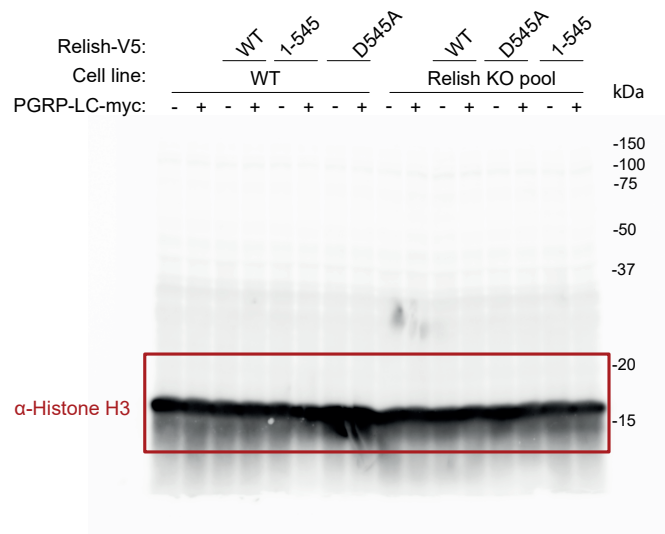

Supplement: Supplementary file 8 — Figure Source Data all EV figs [file 44318_2026_761_MOESM8_ESM.zip › EV Figures/EV1B/Annotation.pdf]

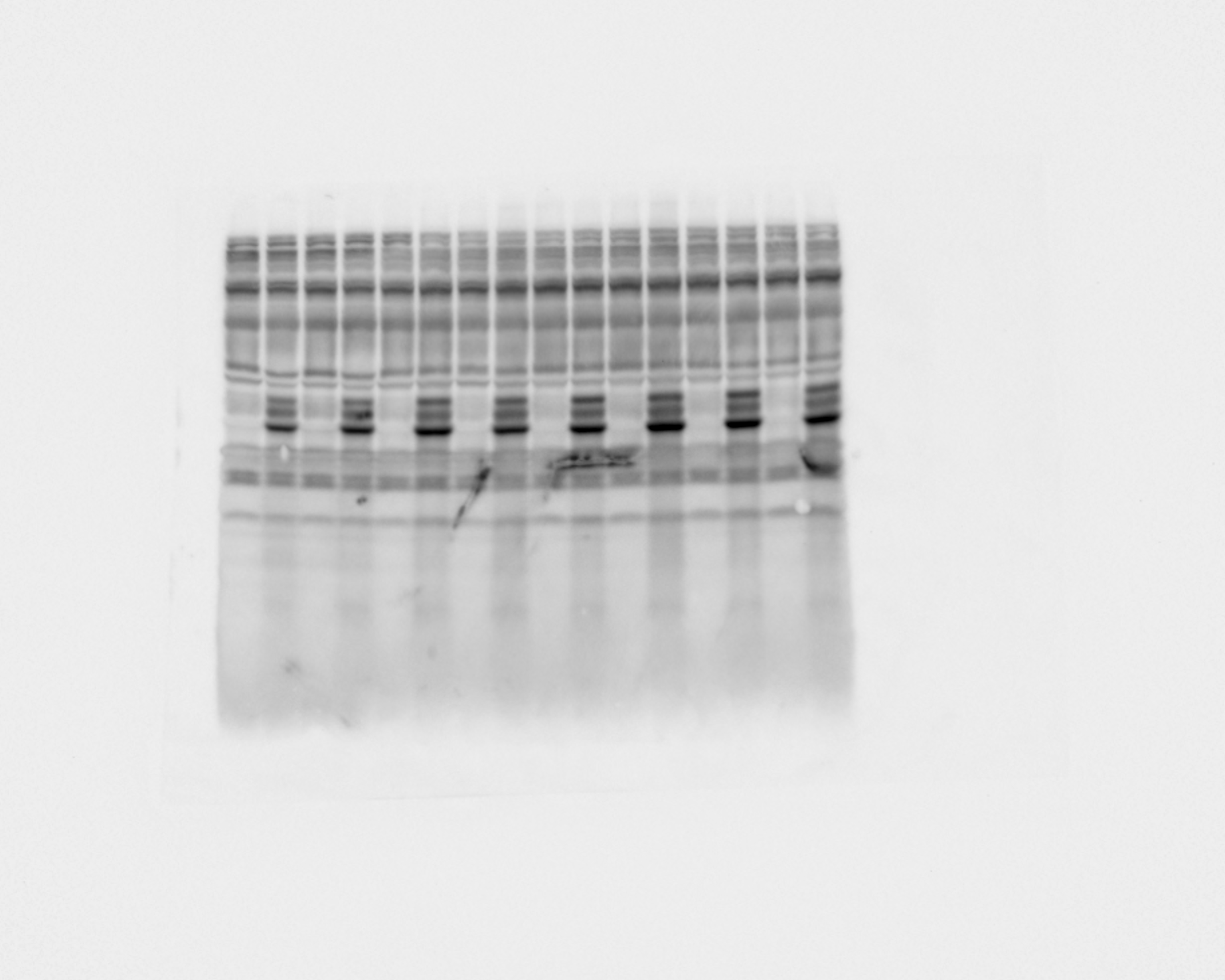

Supplement: Supplementary file 8 — Figure Source Data all EV figs [file 44318_2026_761_MOESM8_ESM.zip › EV Figures/EV1B/Western blot myc.tif]

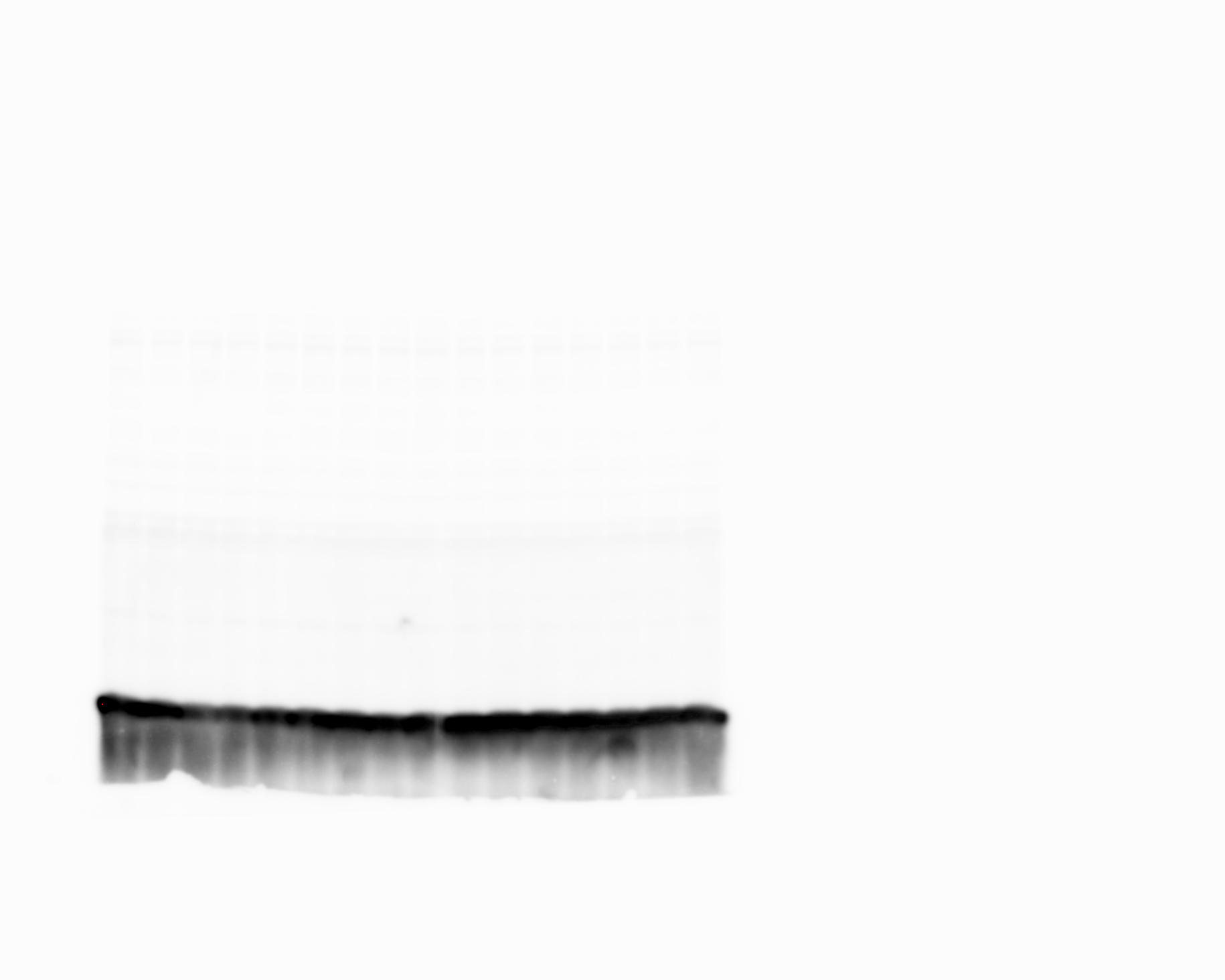

Supplement: Supplementary file 8 — Figure Source Data all EV figs [file 44318_2026_761_MOESM8_ESM.zip › EV Figures/EV1B/Repeat A/Western blot histone H3.tif]

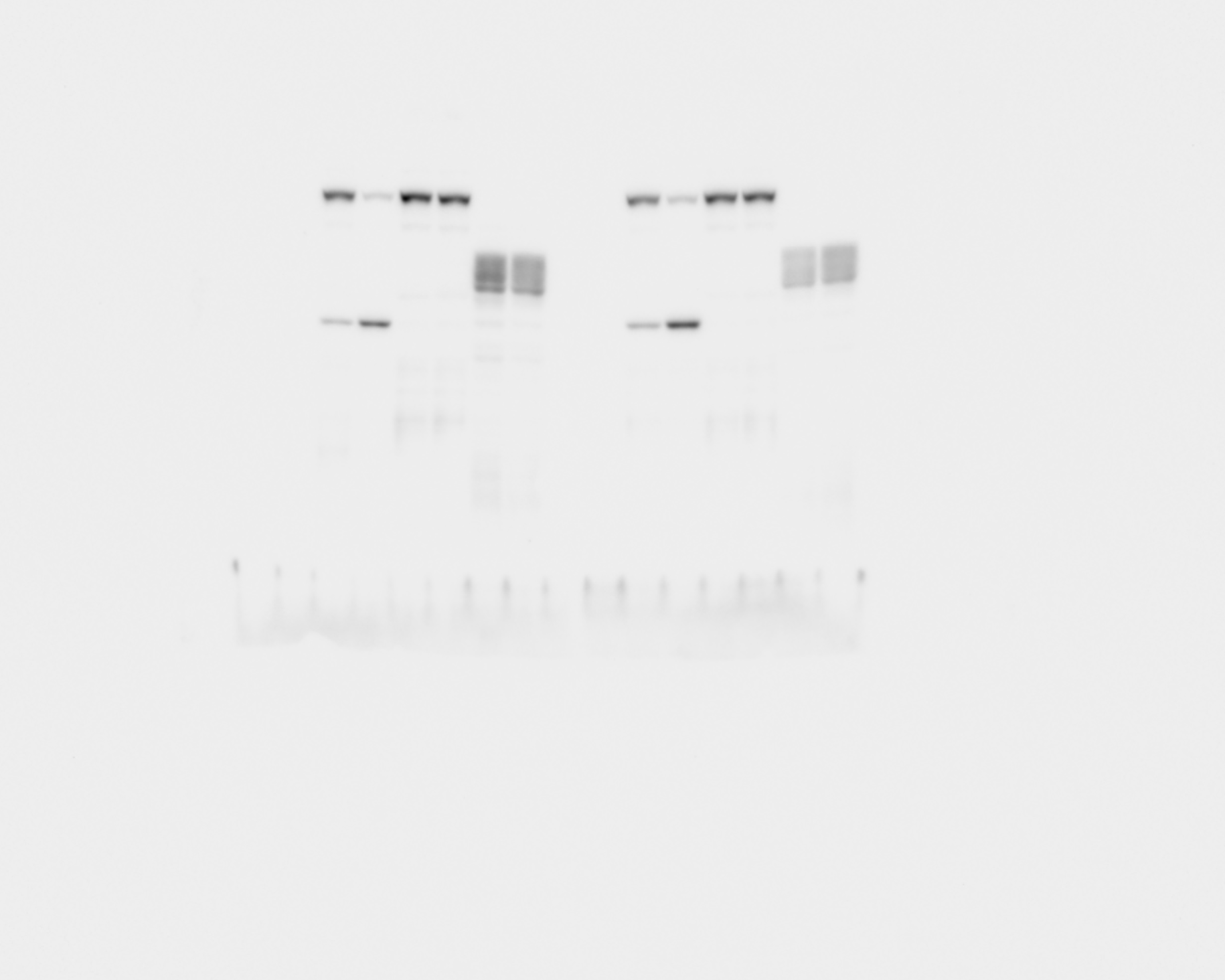

Supplement: Supplementary file 8 — Figure Source Data all EV figs [file 44318_2026_761_MOESM8_ESM.zip › EV Figures/EV1B/Repeat A/Western blot V5.tif]

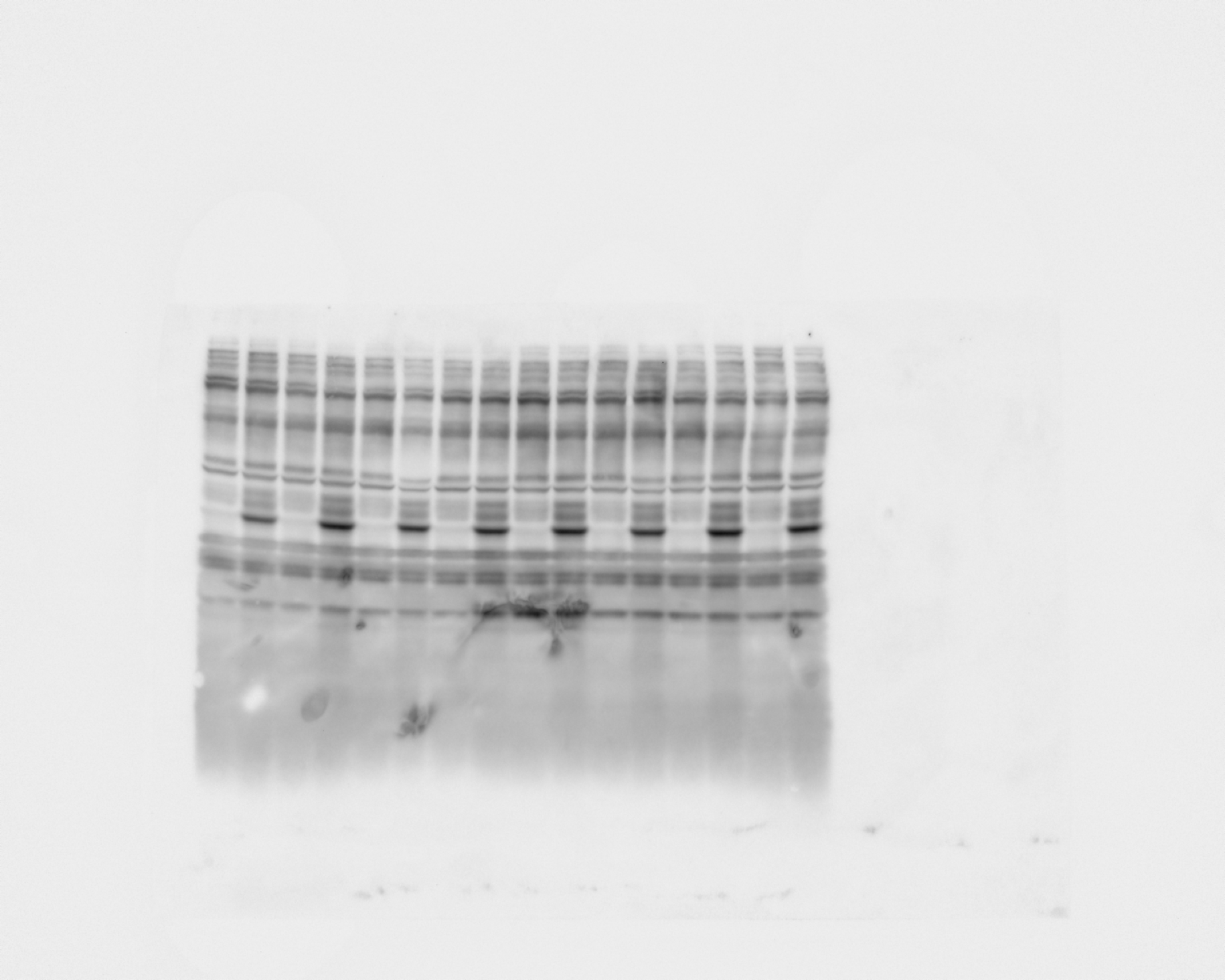

Supplement: Supplementary file 8 — Figure Source Data all EV figs [file 44318_2026_761_MOESM8_ESM.zip › EV Figures/EV1B/Repeat A/Western blot myc.tif]

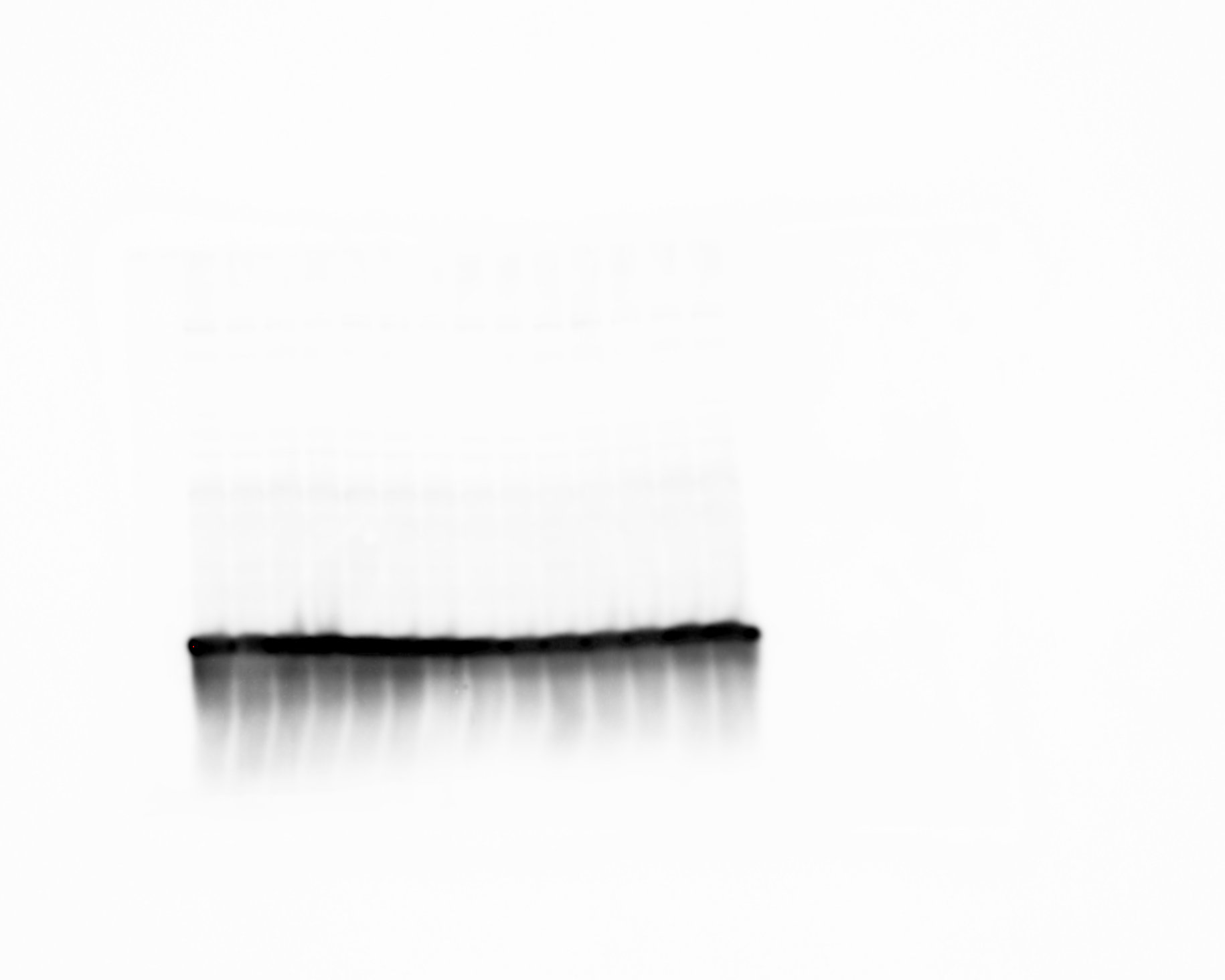

Supplement: Supplementary file 8 — Figure Source Data all EV figs [file 44318_2026_761_MOESM8_ESM.zip › EV Figures/EV4C/Western blot histone H3.tif]

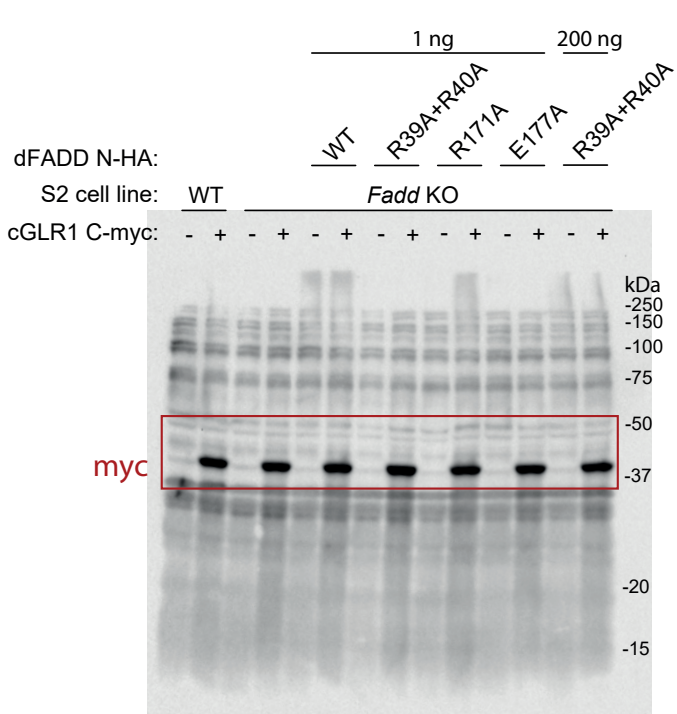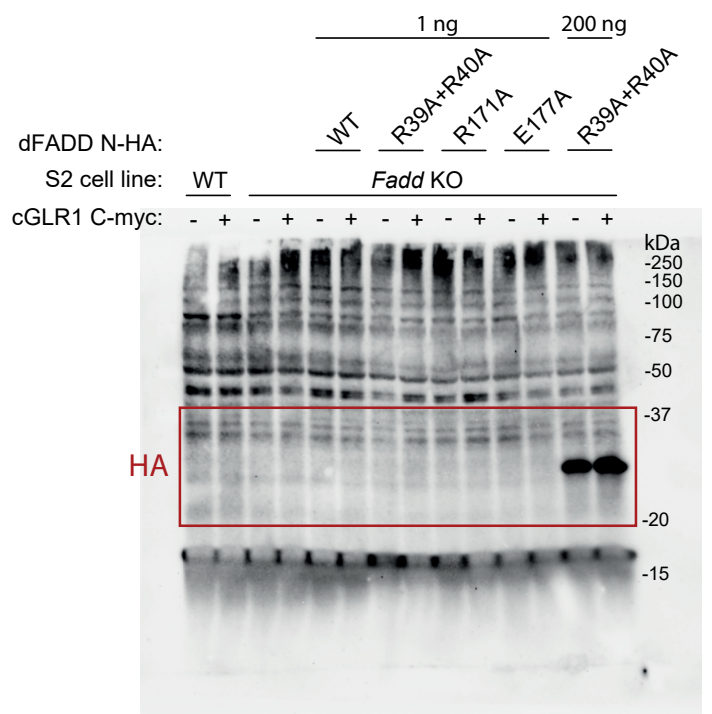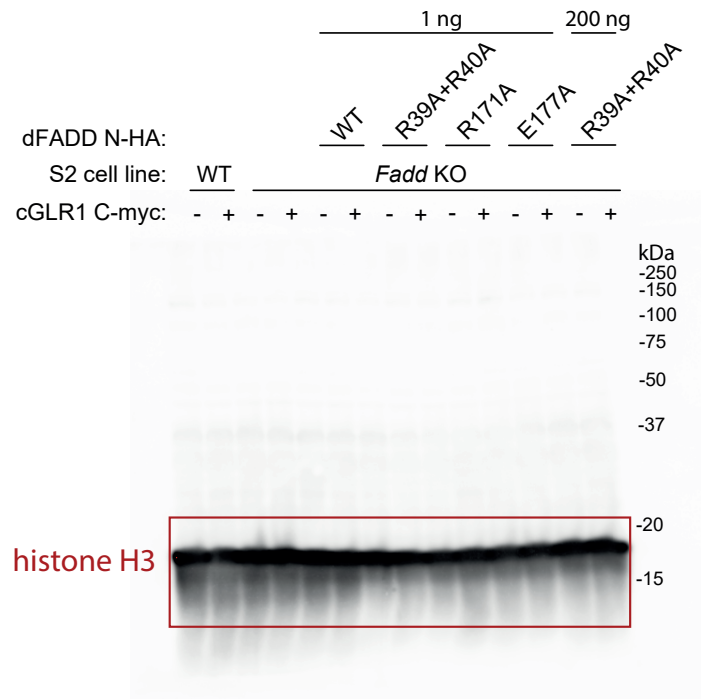

Supplement: Supplementary file 8 — Figure Source Data all EV figs [file 44318_2026_761_MOESM8_ESM.zip › EV Figures/EV4C/Annotation.pdf]

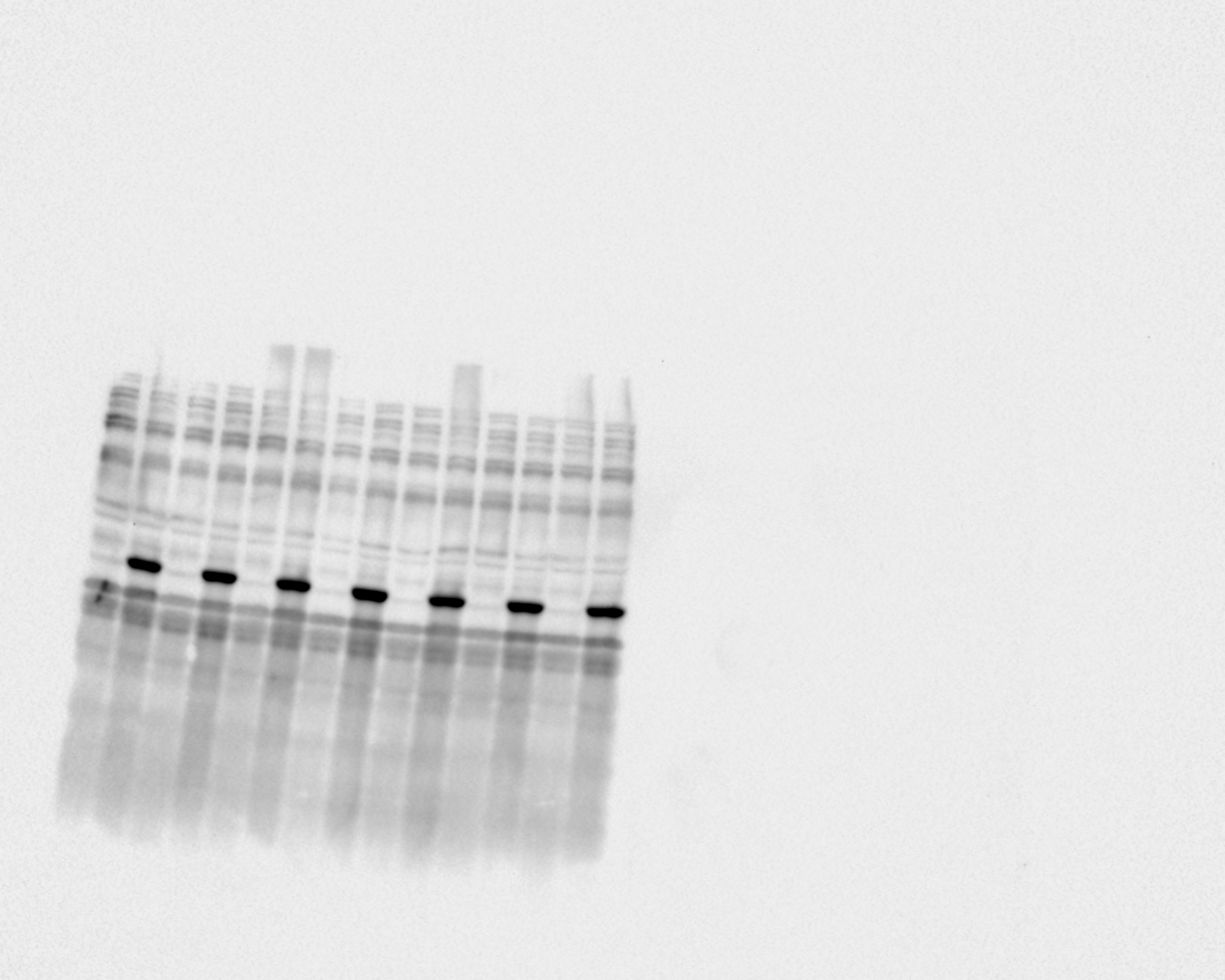

Supplement: Supplementary file 8 — Figure Source Data all EV figs [file 44318_2026_761_MOESM8_ESM.zip › EV Figures/EV4C/Western blot myc.tif]

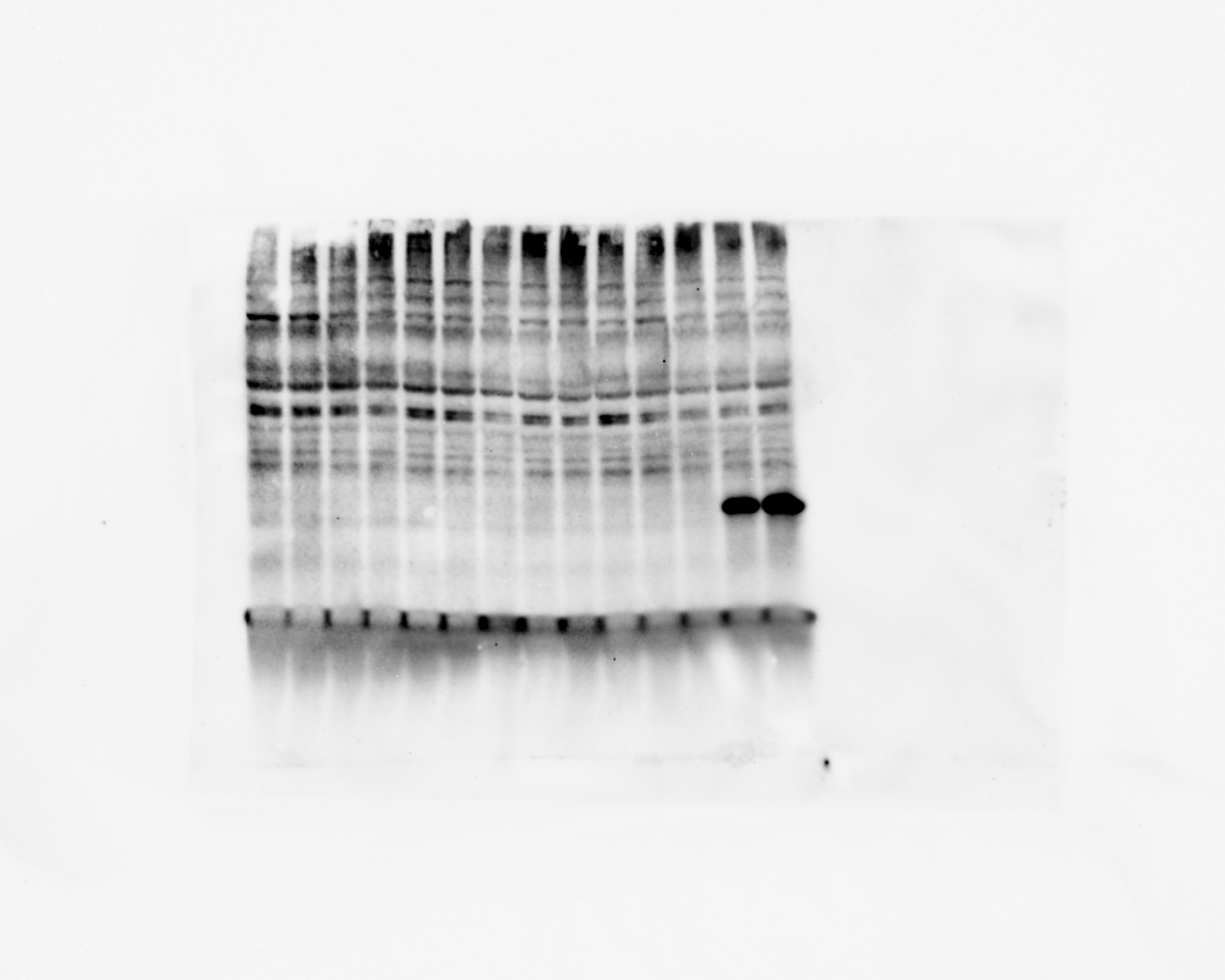

Supplement: Supplementary file 8 — Figure Source Data all EV figs [file 44318_2026_761_MOESM8_ESM.zip › EV Figures/EV4C/Western blot HA.tif]

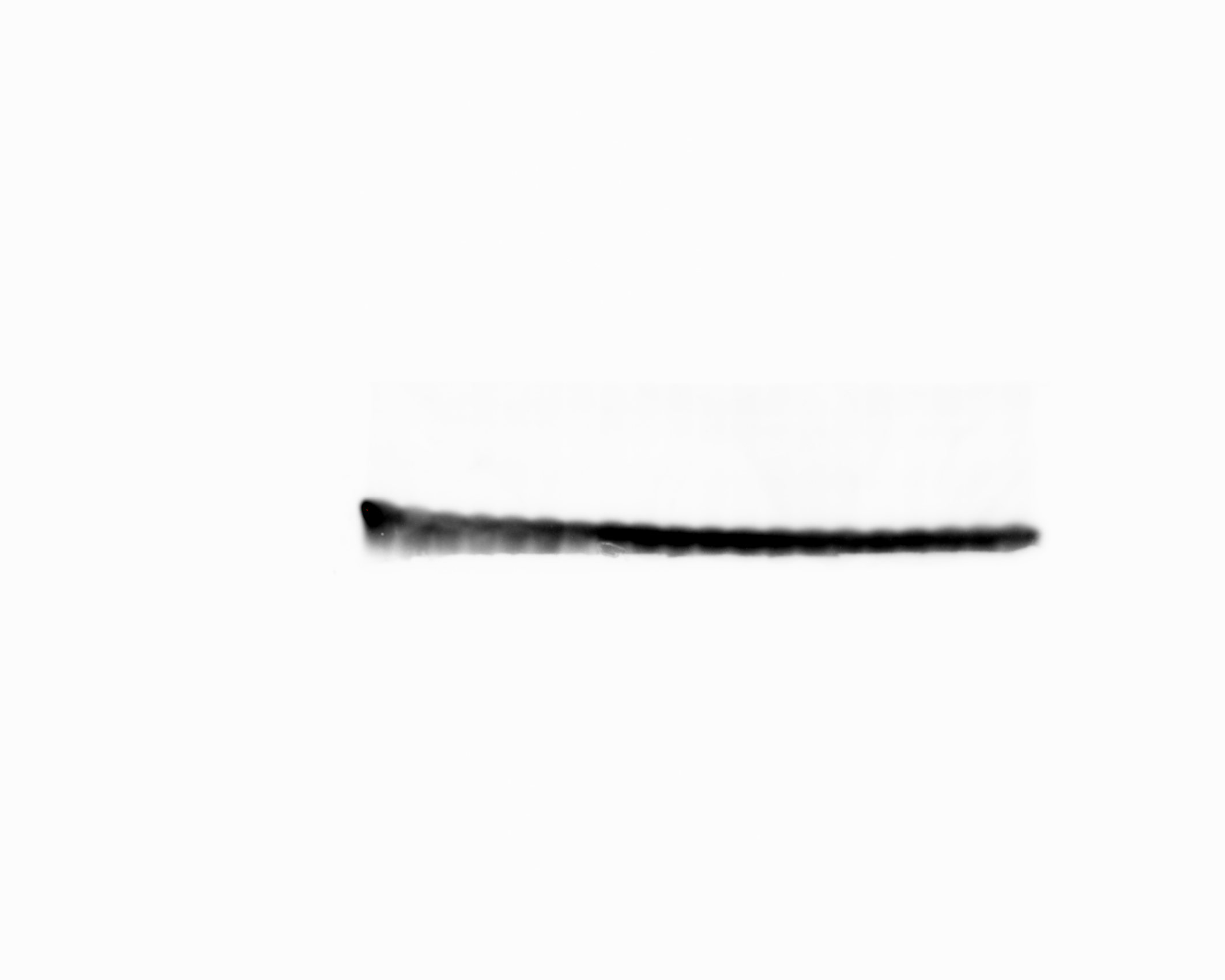

Supplement: Supplementary file 8 — Figure Source Data all EV figs [file 44318_2026_761_MOESM8_ESM.zip › EV Figures/EV4E/Western blot histone H3.tif]

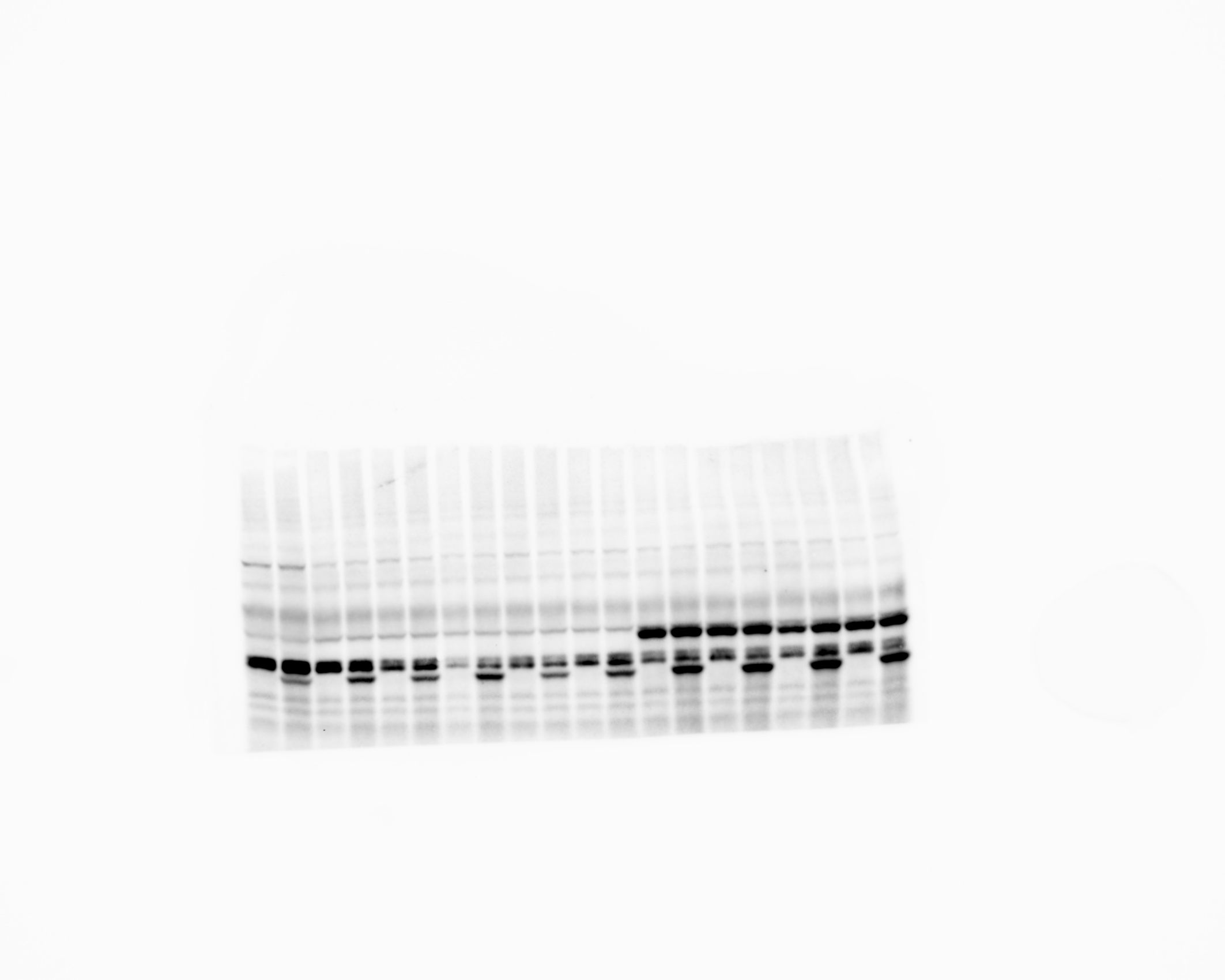

Supplement: Supplementary file 8 — Figure Source Data all EV figs [file 44318_2026_761_MOESM8_ESM.zip › EV Figures/EV4E/Western blot HA and myc.tif]

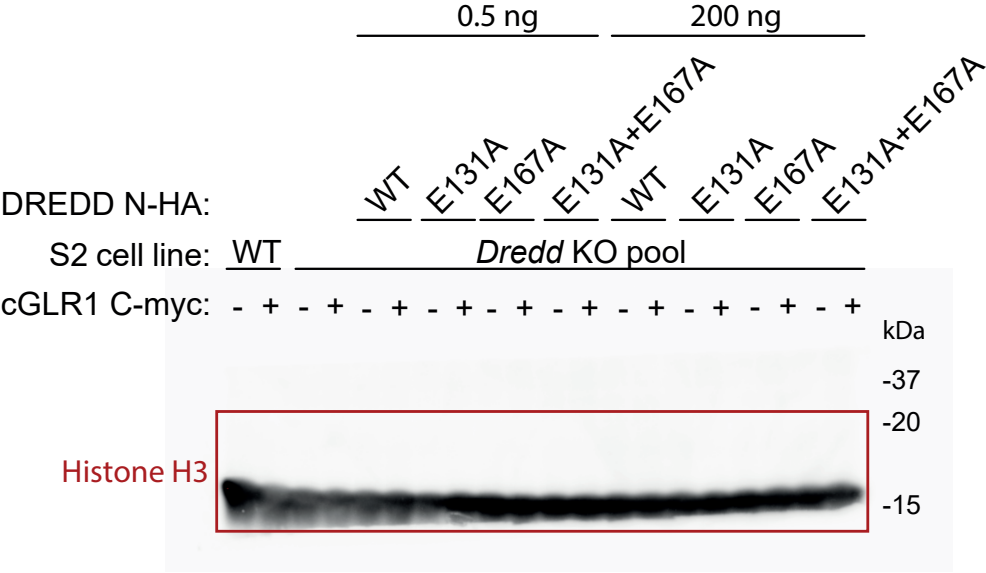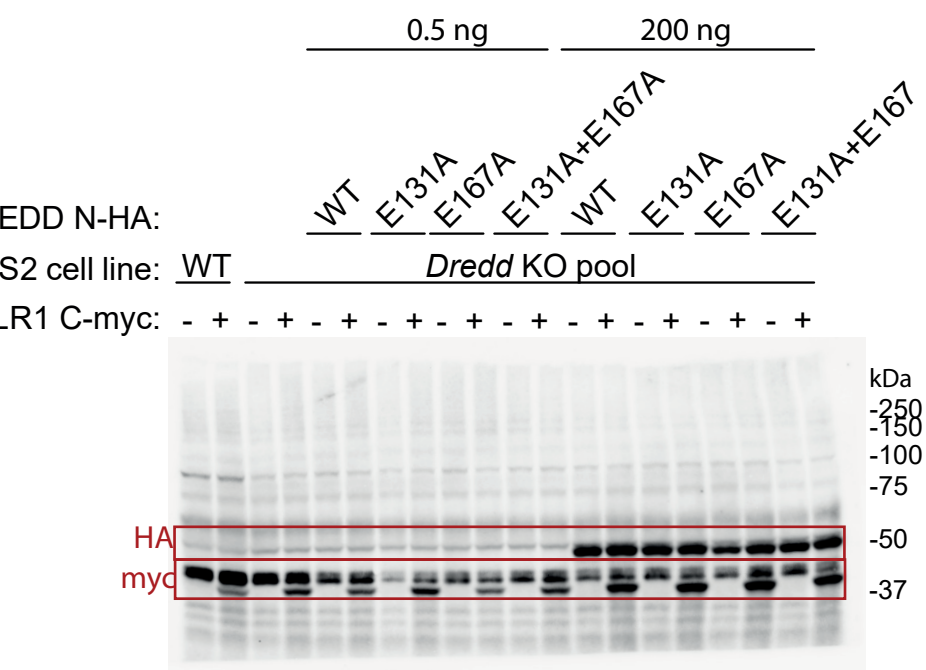

Supplement: Supplementary file 8 — Figure Source Data all EV figs [file 44318_2026_761_MOESM8_ESM.zip › EV Figures/EV4E/Annotation.pdf]

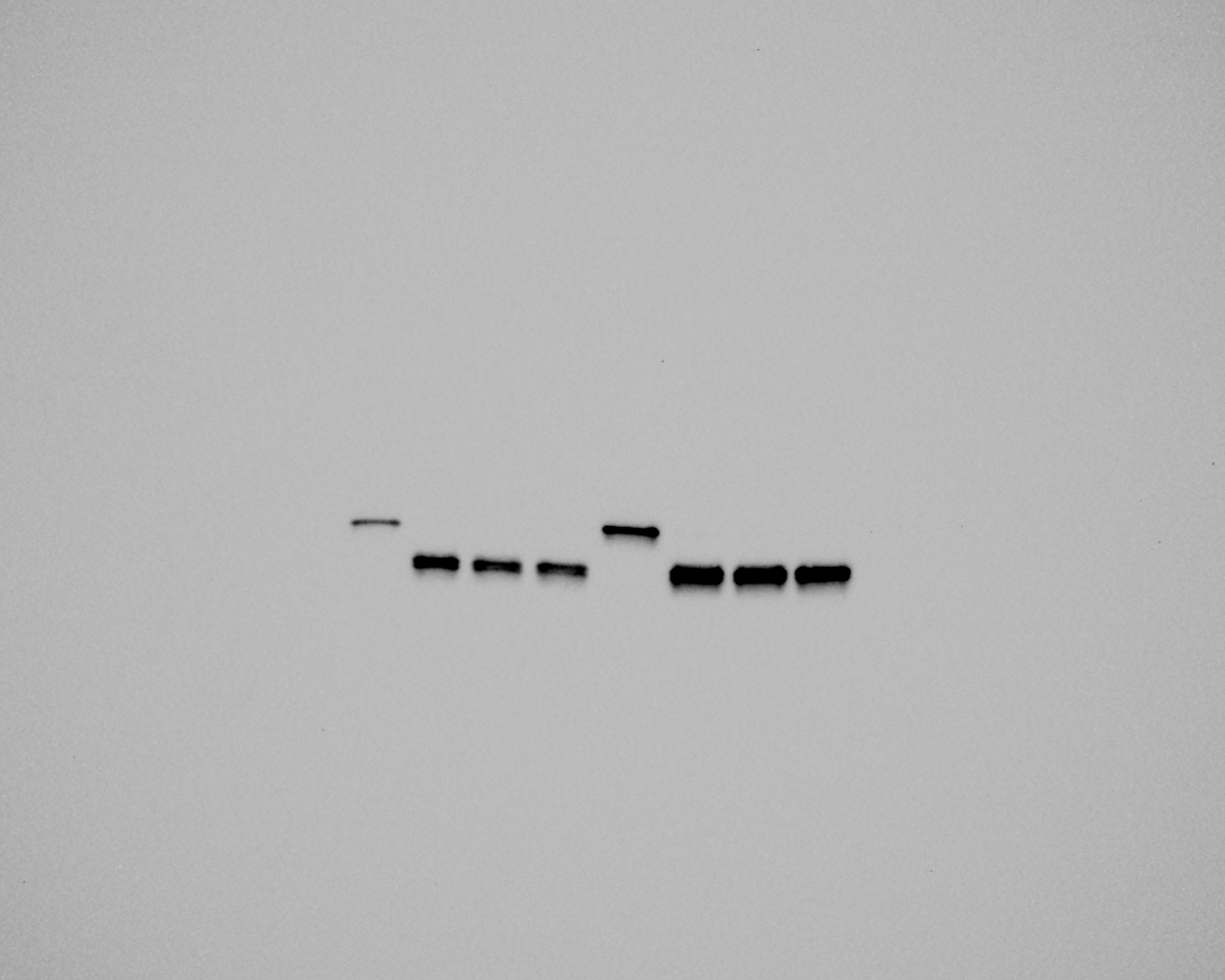

Supplement: Supplementary file 8 — Figure Source Data all EV figs [file 44318_2026_761_MOESM8_ESM.zip › EV Figures/EV5C/Western blot V5.tif]

|             |   |   |   |   |   |   |   |
|-------------|---|---|---|---|---|---|---|
| DREDD N-HA: |   |   |   |   |   |   |   |
|             |   |   |   |   |   |   | + |
| E177A C-V5: | - | - | - | + | - | - | + |
| R171A C-V5: | - | - | + | - | - | - | + |
| dFADD C-V5: | - | + | - | - | - | + | - |
| RLuc C-V5:  | + | - | - | - | + | - | - |

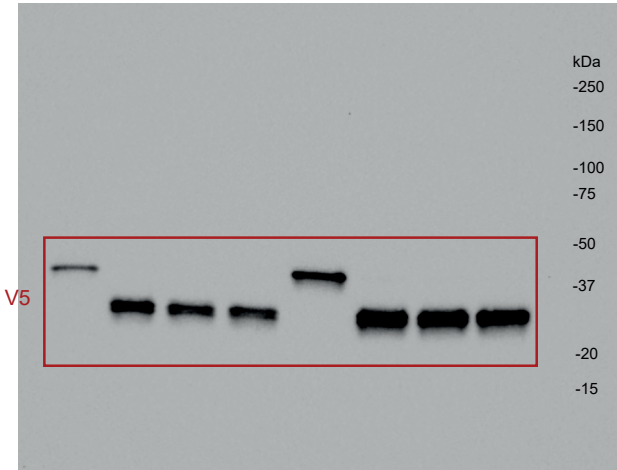

|             |   |   |   |   |   |   |   |
|-------------|---|---|---|---|---|---|---|
| DREDD N-HA: |   |   |   |   |   |   |   |
|             |   |   |   |   |   |   | + |
| E177A C-V5: | - | - | - | + | - | - | + |
| R171A C-V5: | - | - | + | - | - | - | + |
| dFADD C-V5: | - | + | - | - | - | + | - |
| RLuc C-V5:  | + | - | - | - | + | - | - |

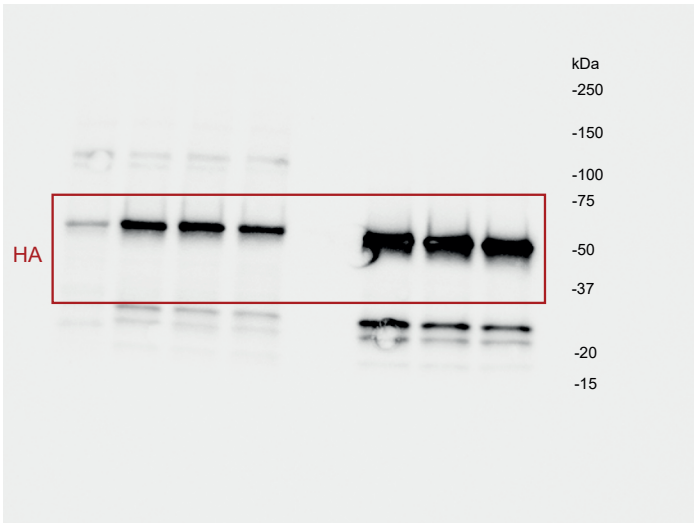

Supplement: Supplementary file 8 — Figure Source Data all EV figs [file 44318_2026_761_MOESM8_ESM.zip › EV Figures/EV5C/Annotation.pdf]

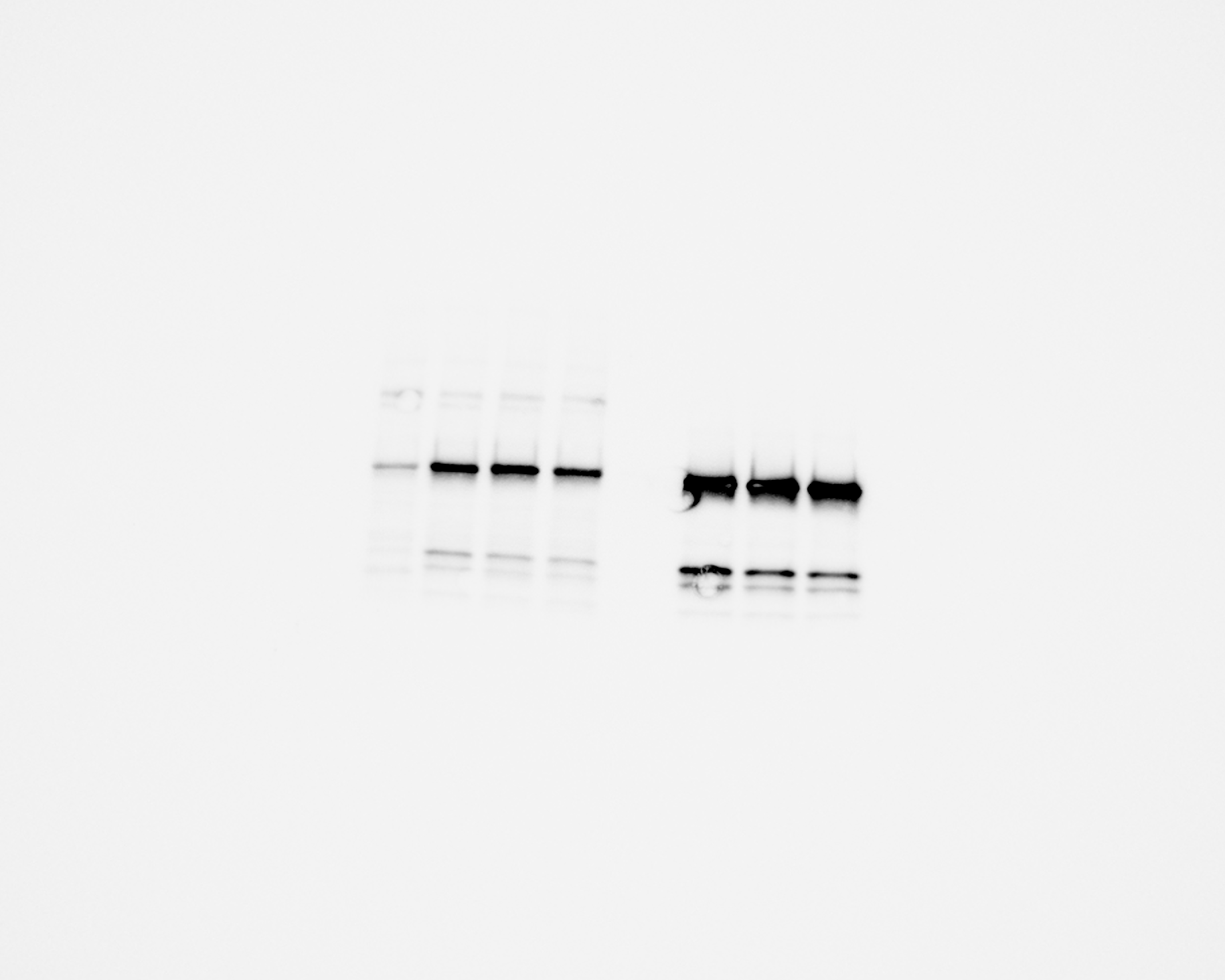

Supplement: Supplementary file 8 — Figure Source Data all EV figs [file 44318_2026_761_MOESM8_ESM.zip › EV Figures/EV5C/Western blot HA.tif]

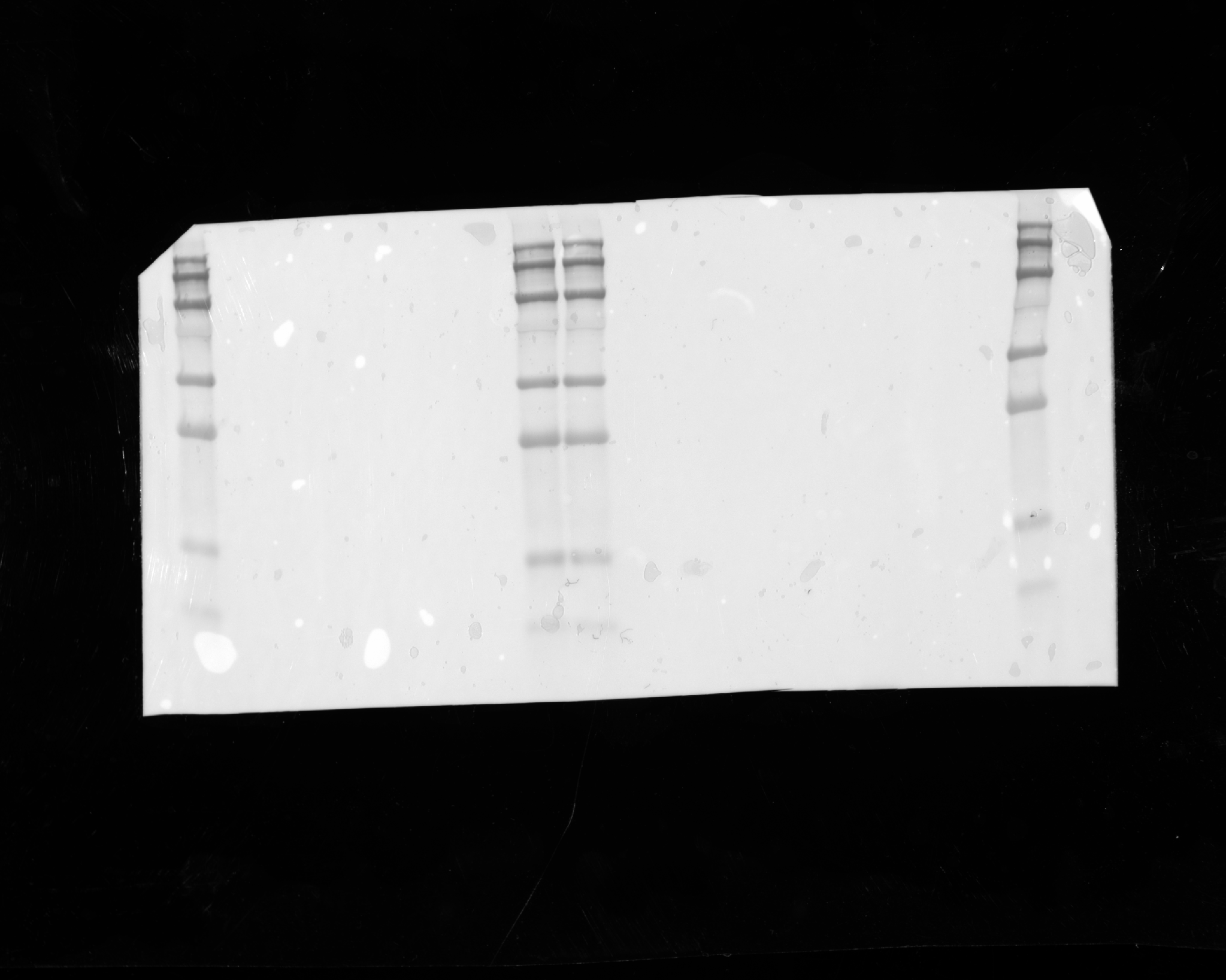

Supplement: Supplementary file 8 — Figure Source Data all EV figs [file 44318_2026_761_MOESM8_ESM.zip › EV Figures/EV5C/Repeat A/Marker V5.tif]

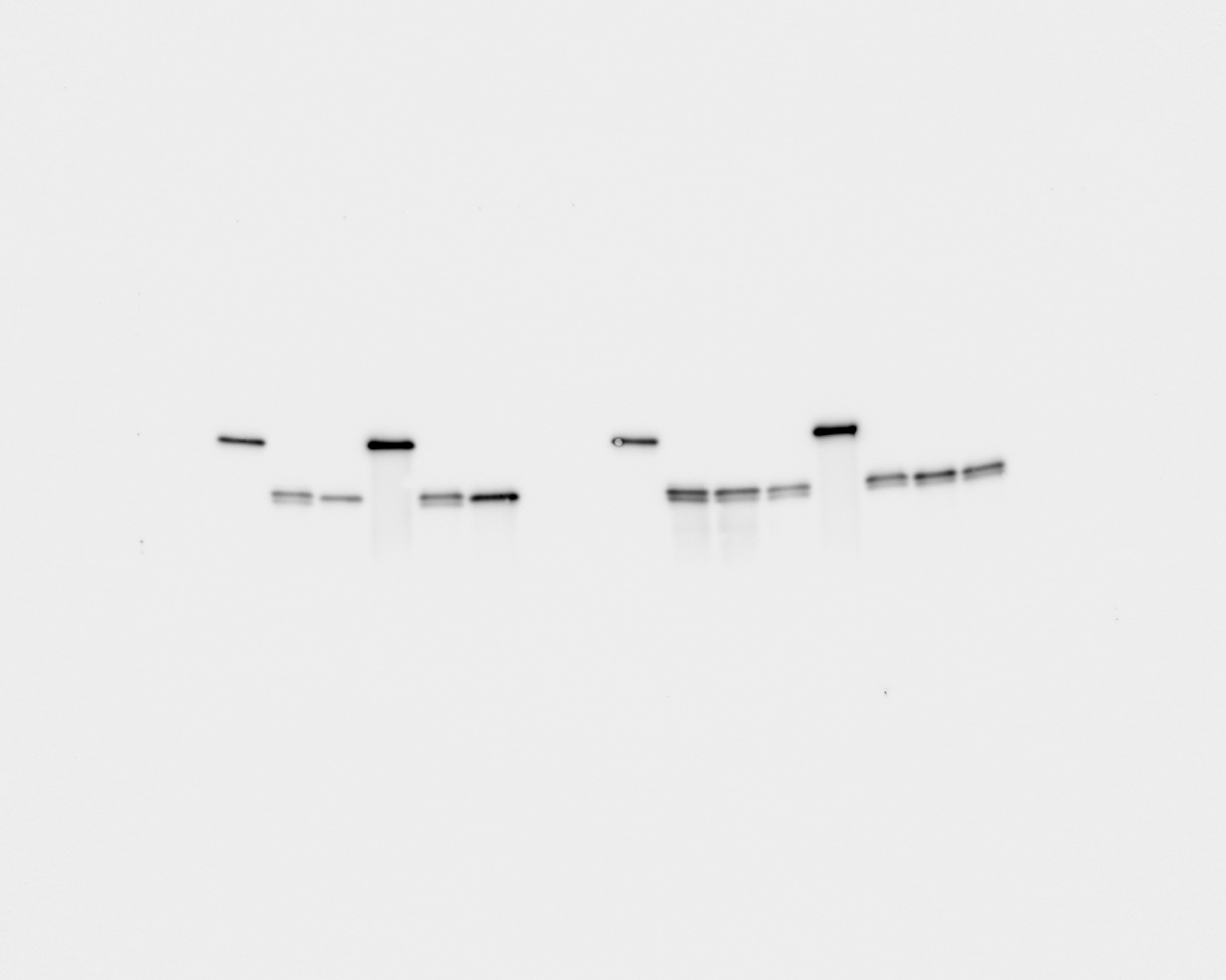

Supplement: Supplementary file 8 — Figure Source Data all EV figs [file 44318_2026_761_MOESM8_ESM.zip › EV Figures/EV5C/Repeat A/Western blot V5 (right side).tif]

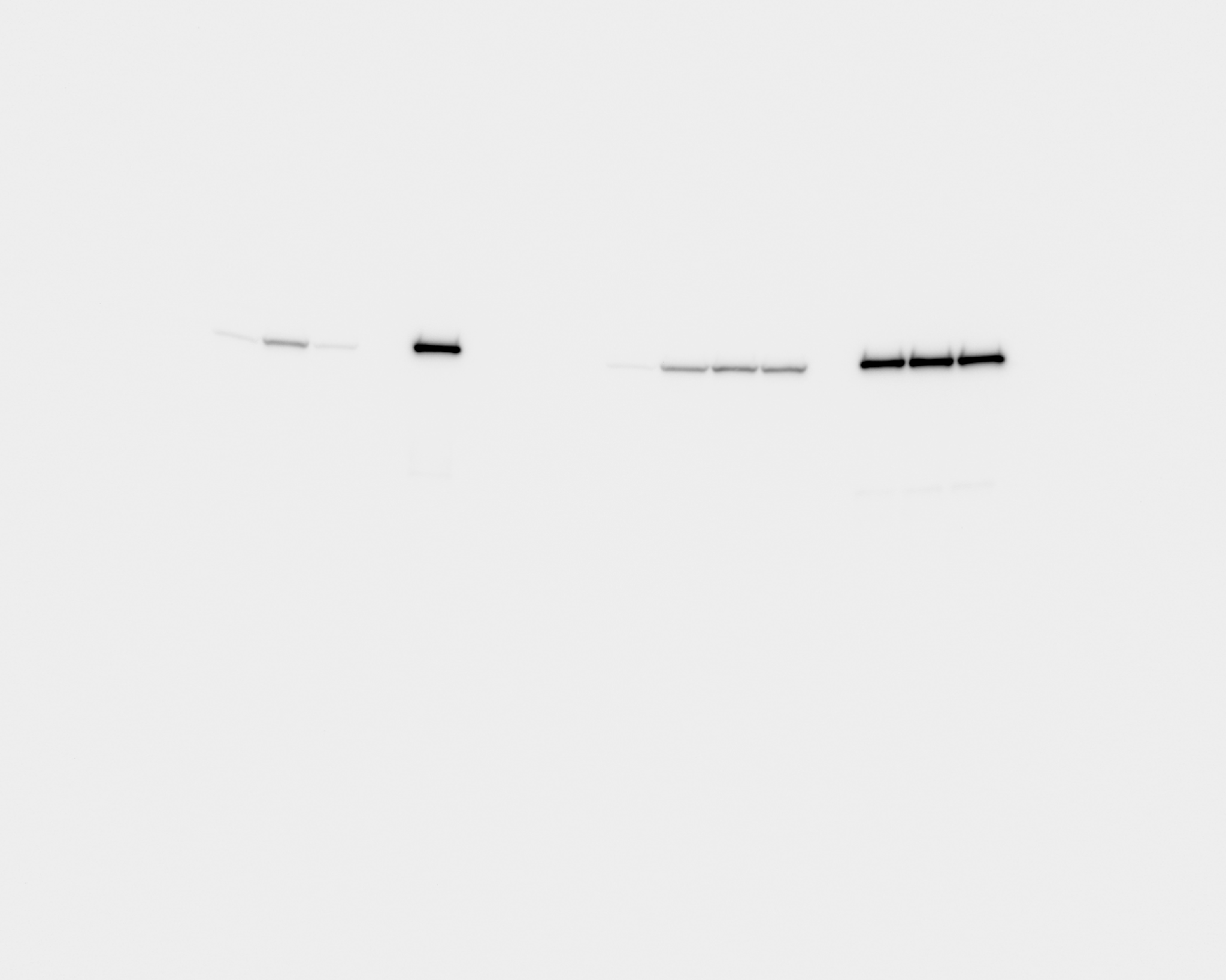

Supplement: Supplementary file 8 — Figure Source Data all EV figs [file 44318_2026_761_MOESM8_ESM.zip › EV Figures/EV5C/Repeat A/Western blot HA (right side).tif]

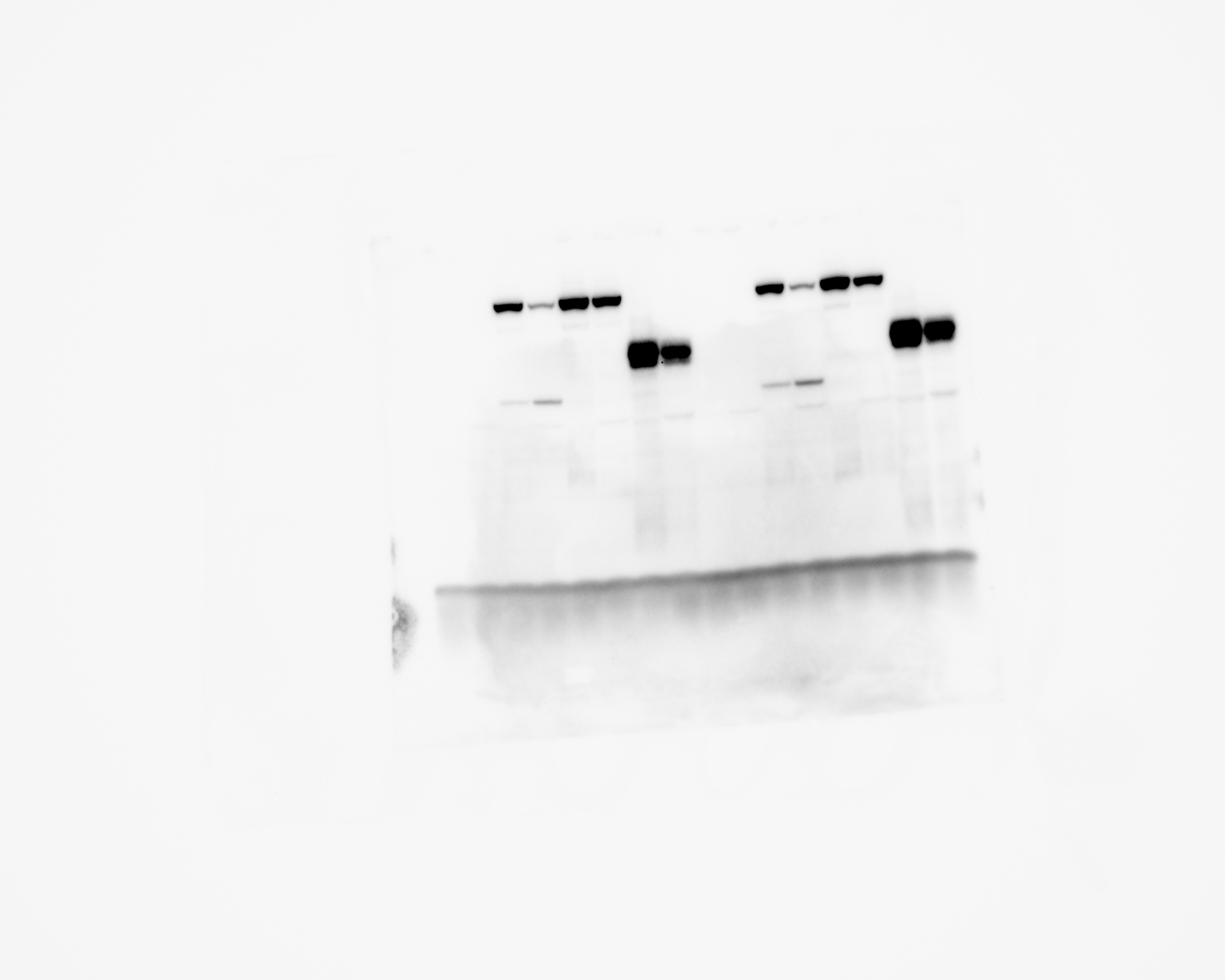

Supplement: Supplementary file 8 — Figure Source Data all EV figs [file 44318_2026_761_MOESM8_ESM.zip › EV Figures/EV1D/Western blot histone H3.tif]

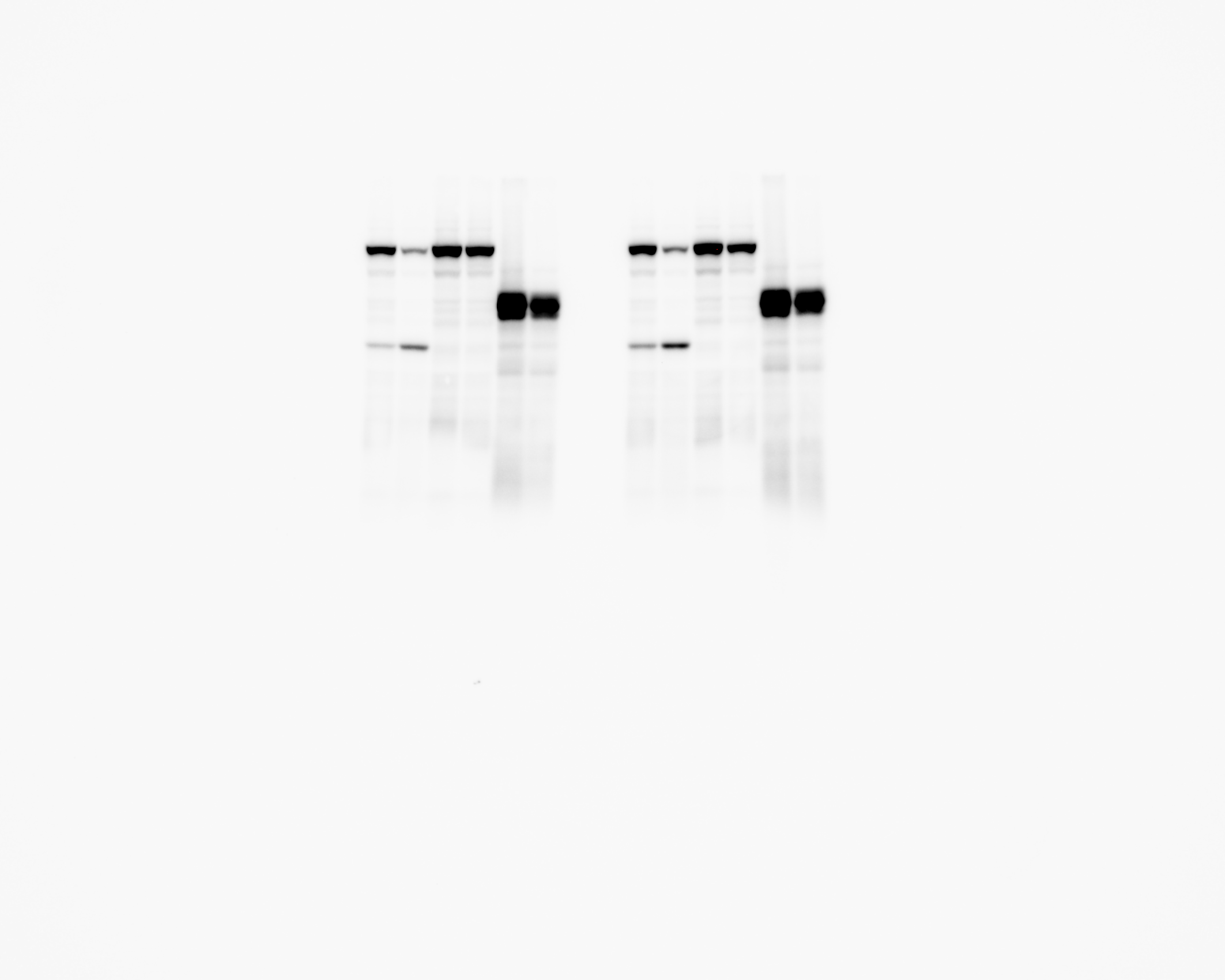

Supplement: Supplementary file 8 — Figure Source Data all EV figs [file 44318_2026_761_MOESM8_ESM.zip › EV Figures/EV1D/Western blot V5.tif]

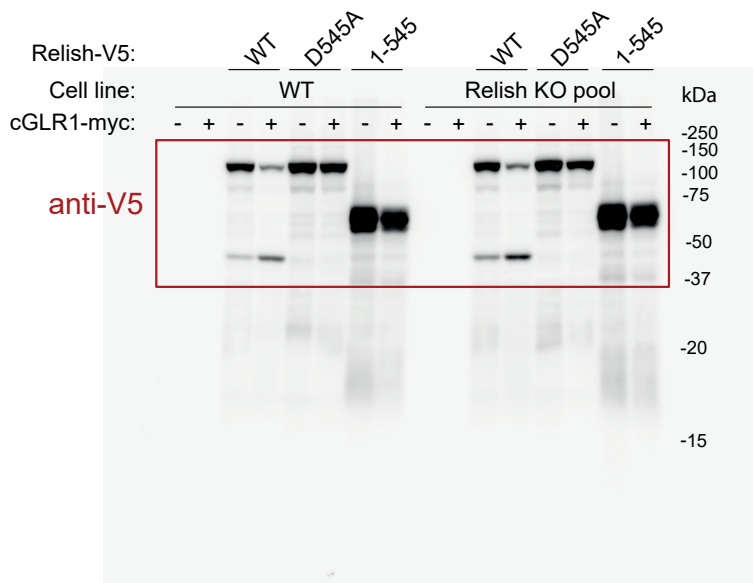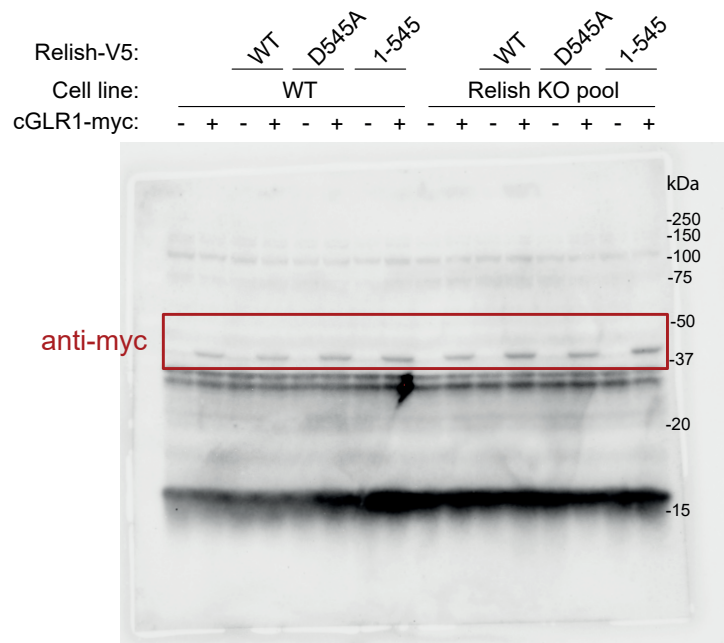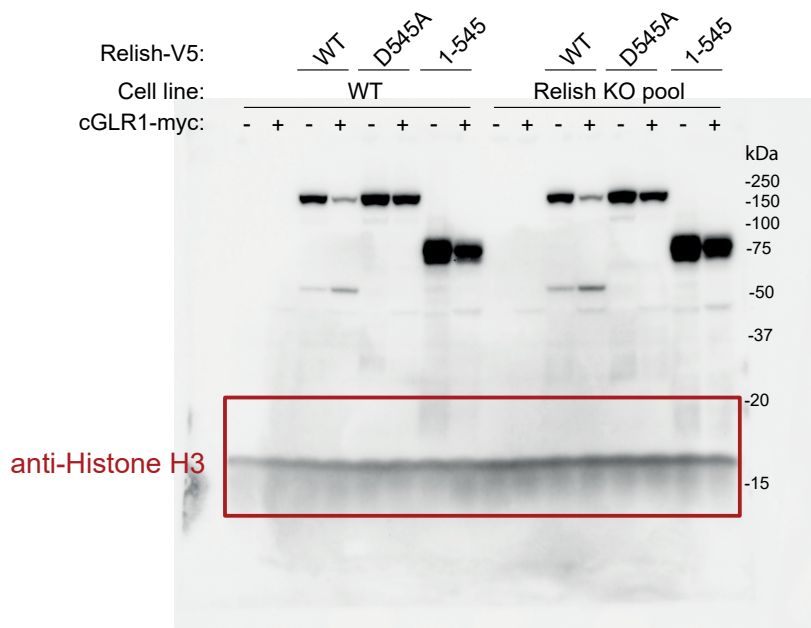

Supplement: Supplementary file 8 — Figure Source Data all EV figs [file 44318_2026_761_MOESM8_ESM.zip › EV Figures/EV1D/Annotation.pdf]

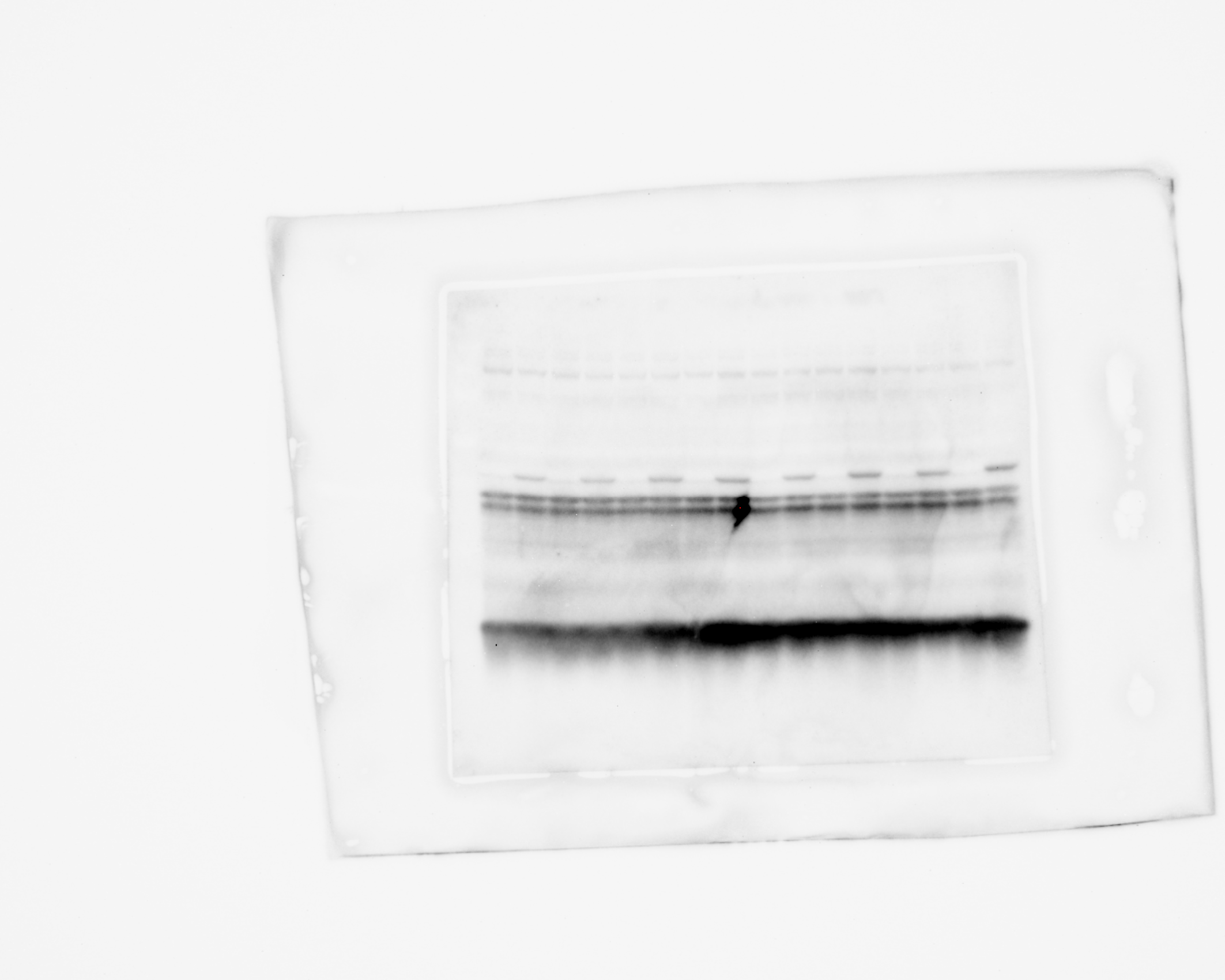

Supplement: Supplementary file 8 — Figure Source Data all EV figs [file 44318_2026_761_MOESM8_ESM.zip › EV Figures/EV1D/Western blot myc.tif]

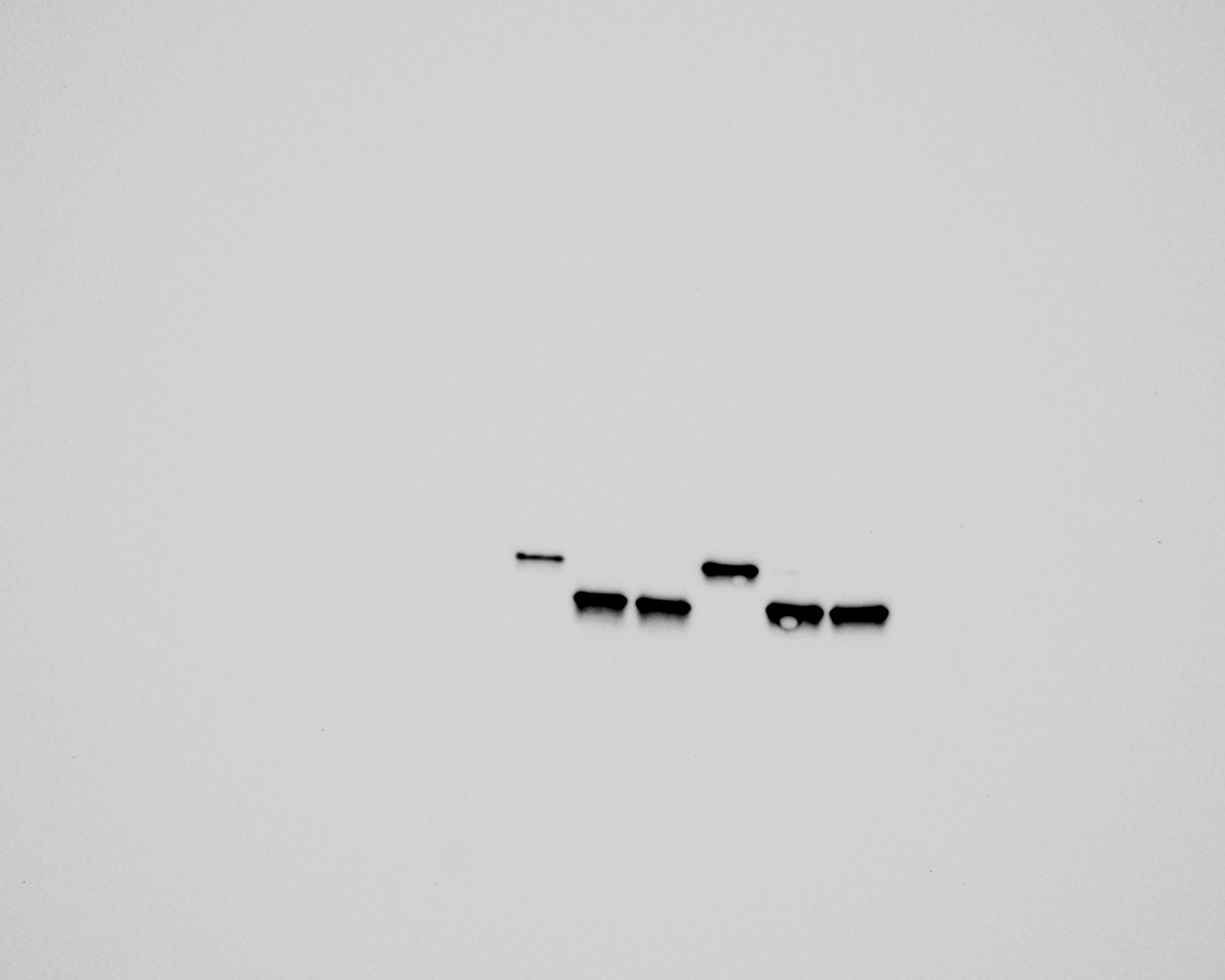

Supplement: Supplementary file 8 — Figure Source Data all EV figs [file 44318_2026_761_MOESM8_ESM.zip › EV Figures/EV5D/Western blot V5.tif]

|                 |   |   |   |   |   |   |
|-----------------|---|---|---|---|---|---|
| IMD N-HA:       | + |   |   |   |   |   |
| R39A+R40A C-V5: | - | - | + | - | - | + |
| dFADD C-V5:     | - | + | - | - | + | - |
| RLuc C-V5:      | + | - | - | + | - | - |

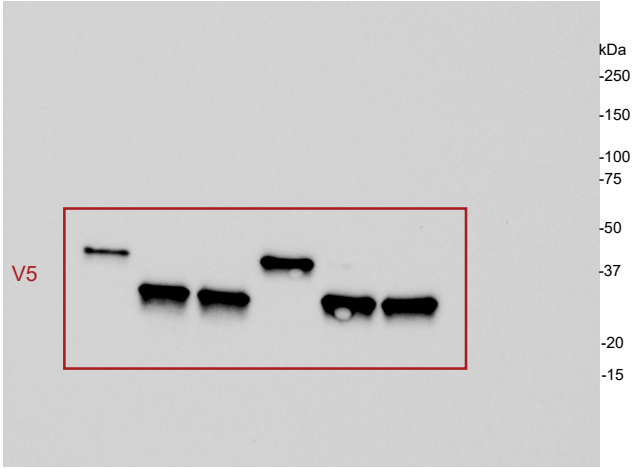

|                 |   |   |   |   |   |   |
|-----------------|---|---|---|---|---|---|
| IMD N-HA:       | + |   |   |   |   |   |
| R39A+R40A C-V5: | - | - | + | - | - | + |
| dFADD C-V5:     | - | + | - | - | + | - |
| RLuc C-V5:      | + | - | - | + | - | - |

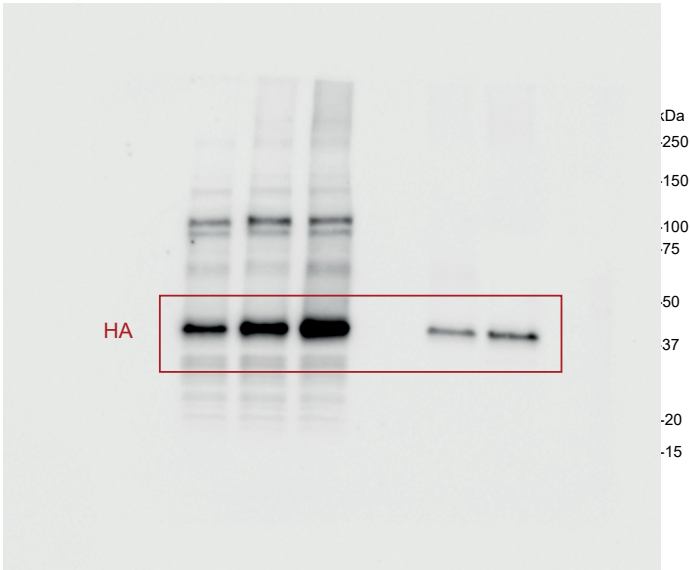

Supplement: Supplementary file 8 — Figure Source Data all EV figs [file 44318_2026_761_MOESM8_ESM.zip › EV Figures/EV5D/Annotation.pdf]

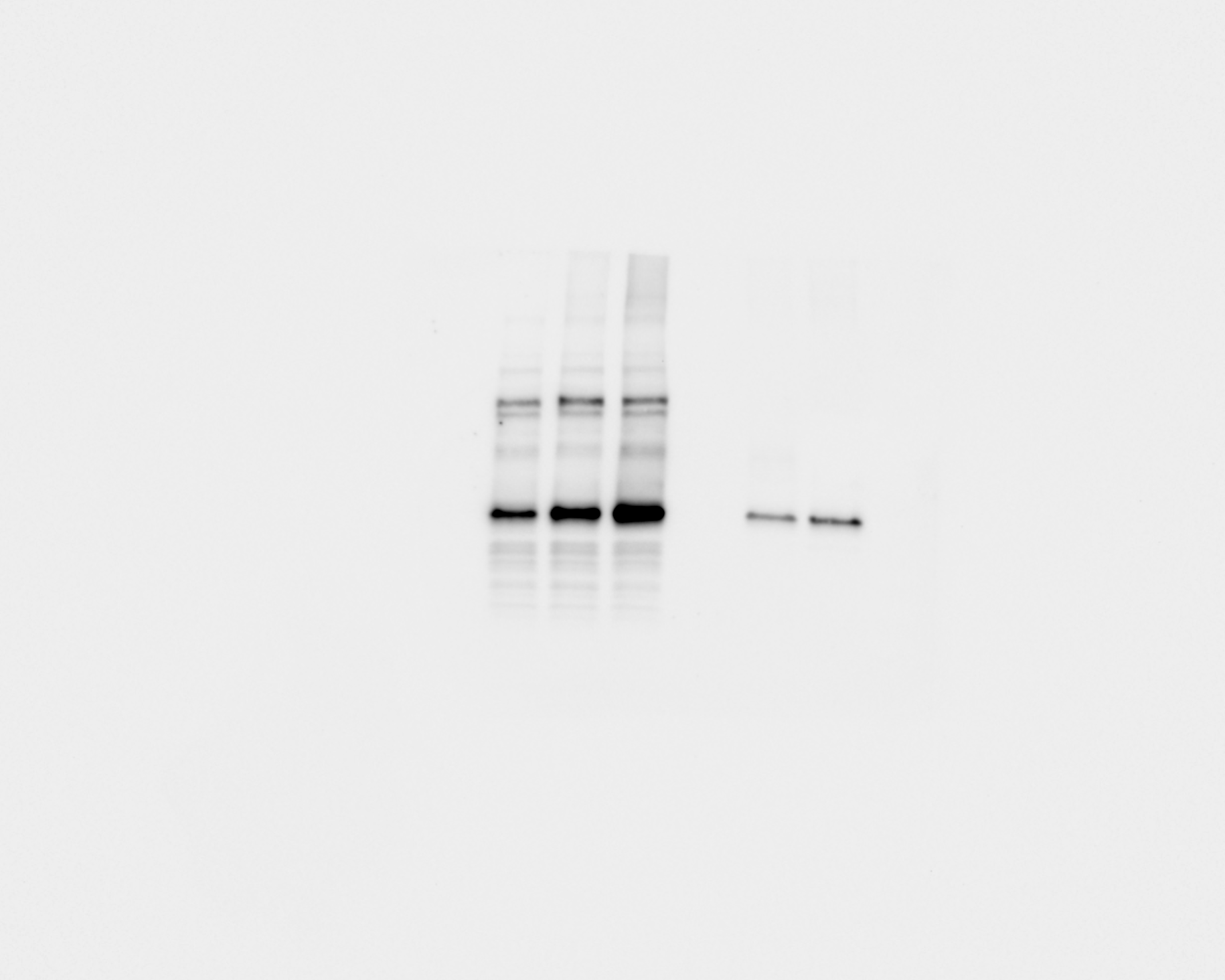

Supplement: Supplementary file 8 — Figure Source Data all EV figs [file 44318_2026_761_MOESM8_ESM.zip › EV Figures/EV5D/Western blot HA.tif]

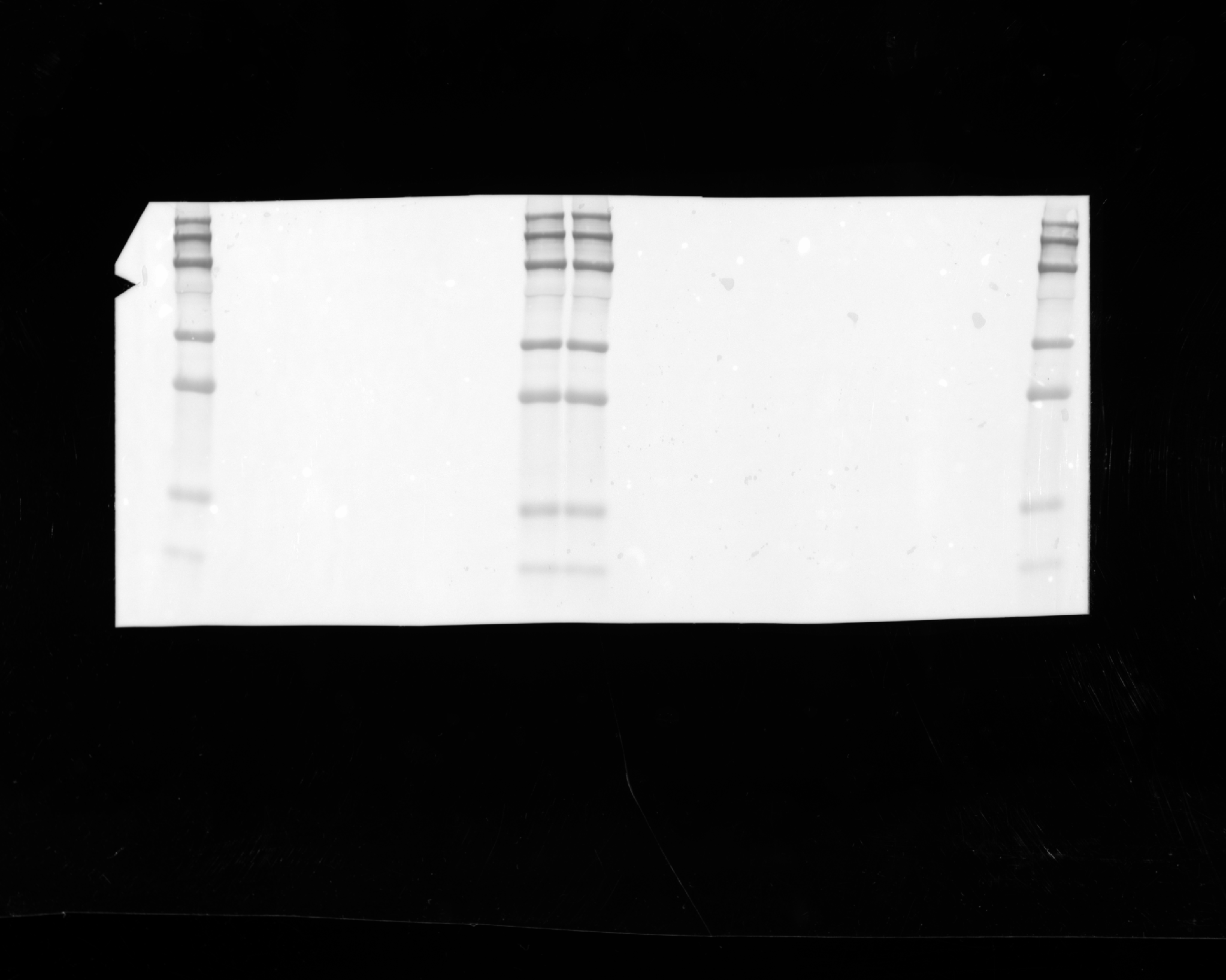

Supplement: Supplementary file 8 — Figure Source Data all EV figs [file 44318_2026_761_MOESM8_ESM.zip › EV Figures/EV5D/Repeat A/Marker V5.tif]

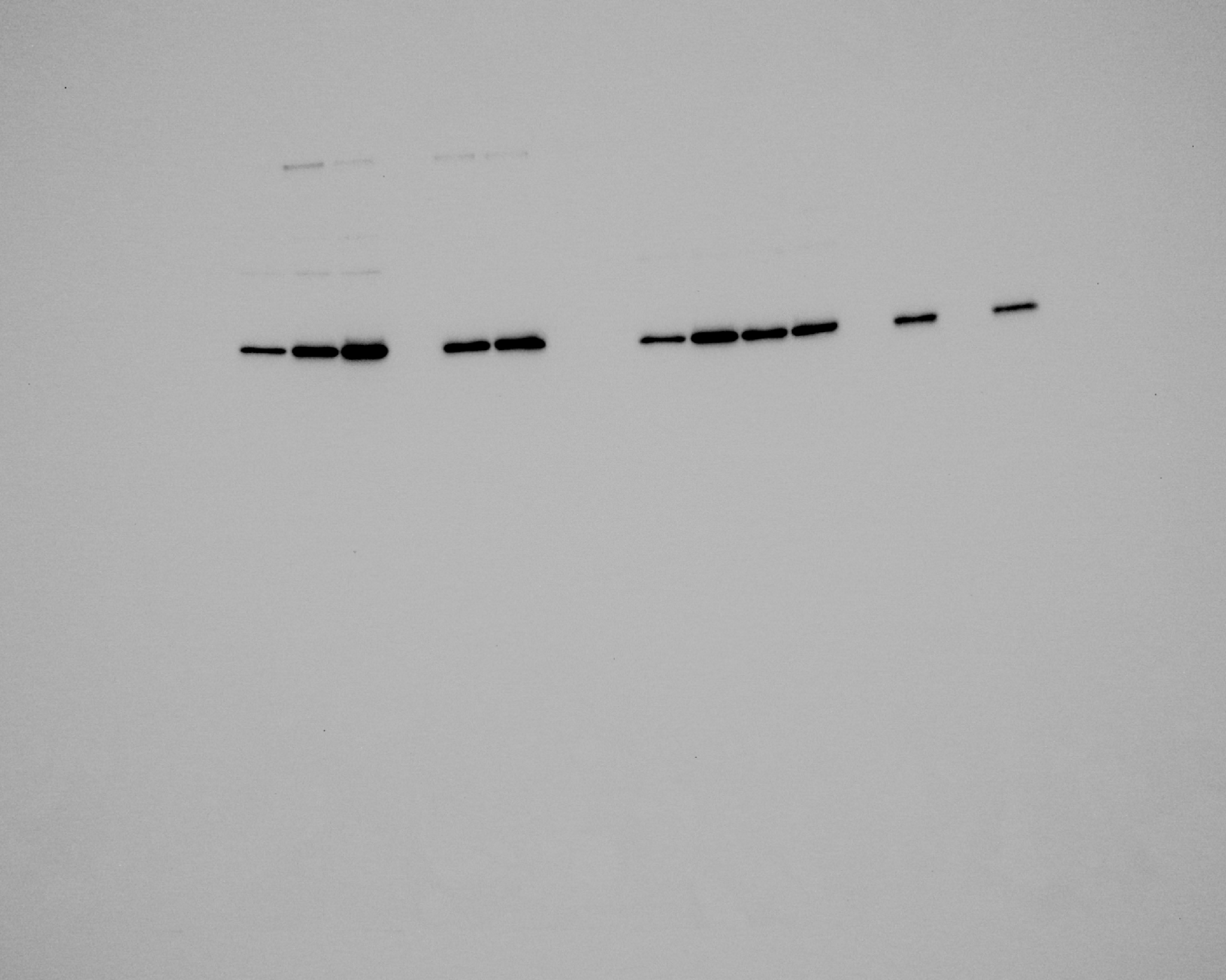

Supplement: Supplementary file 8 — Figure Source Data all EV figs [file 44318_2026_761_MOESM8_ESM.zip › EV Figures/EV5D/Repeat A/Western blot HA (left side).tif]

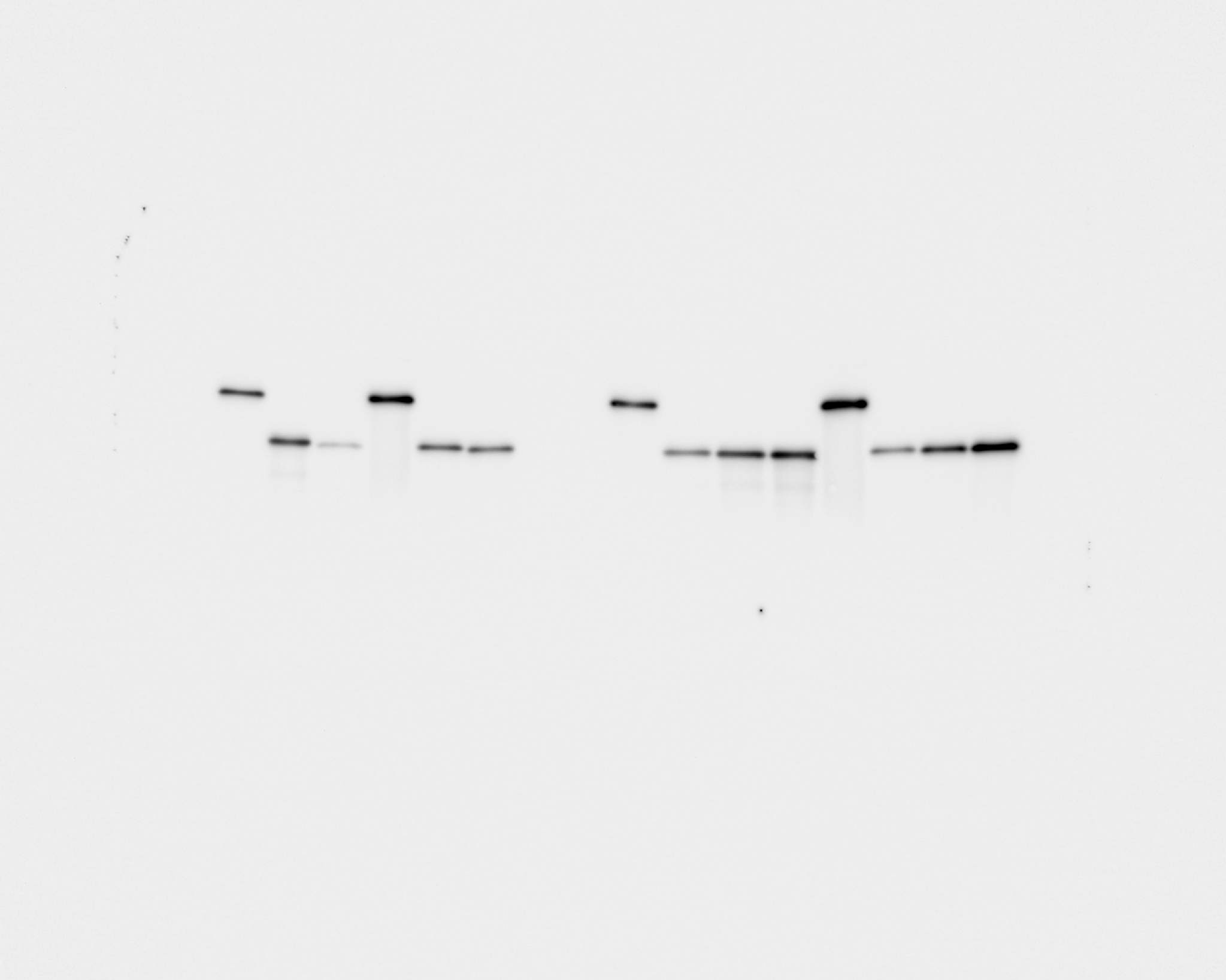

Supplement: Supplementary file 8 — Figure Source Data all EV figs [file 44318_2026_761_MOESM8_ESM.zip › EV Figures/EV5D/Repeat A/Western blot V5 (left side).tif]

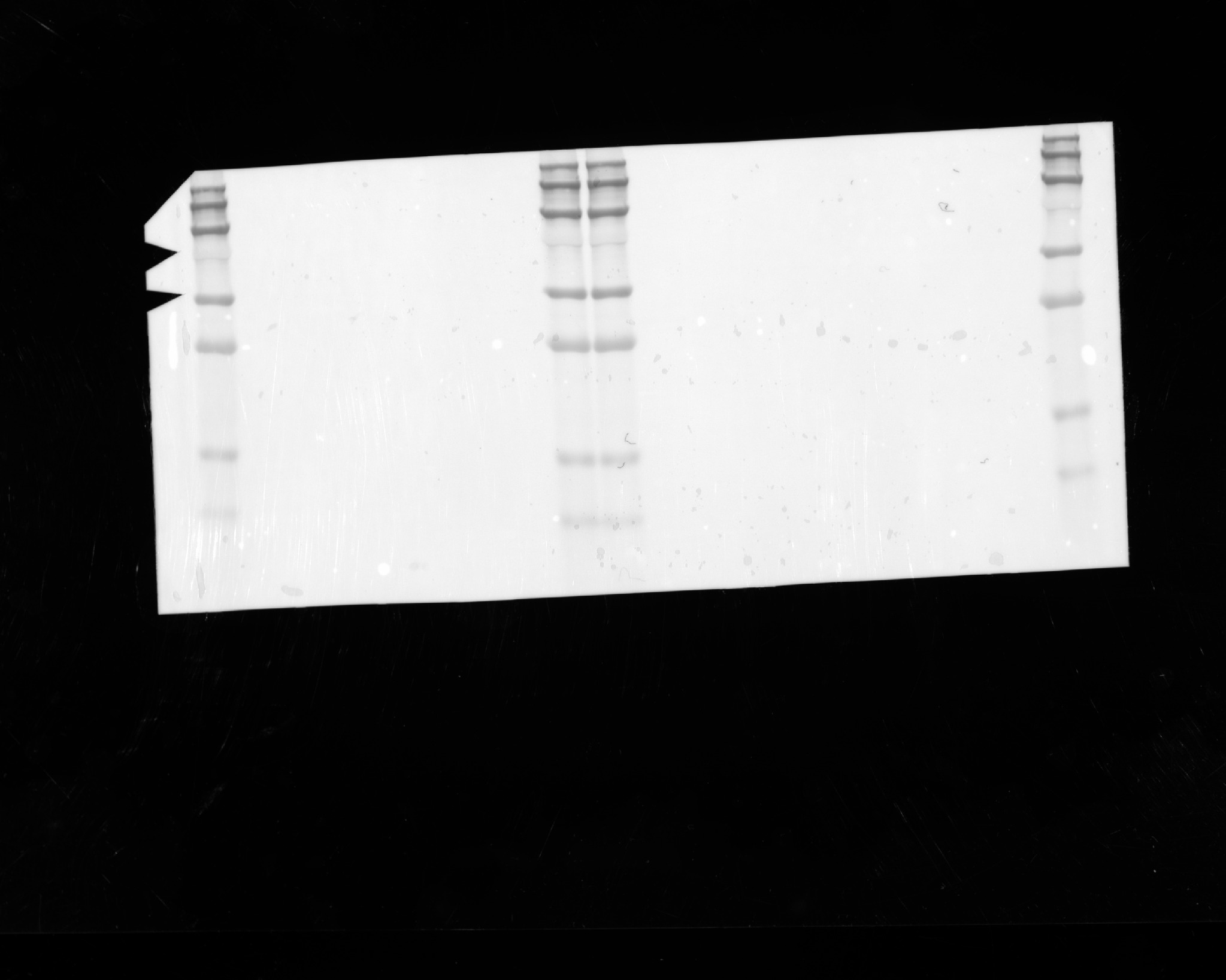

Supplement: Supplementary file 8 — Figure Source Data all EV figs [file 44318_2026_761_MOESM8_ESM.zip › EV Figures/EV5D/Repeat A/Marker HA.tif]

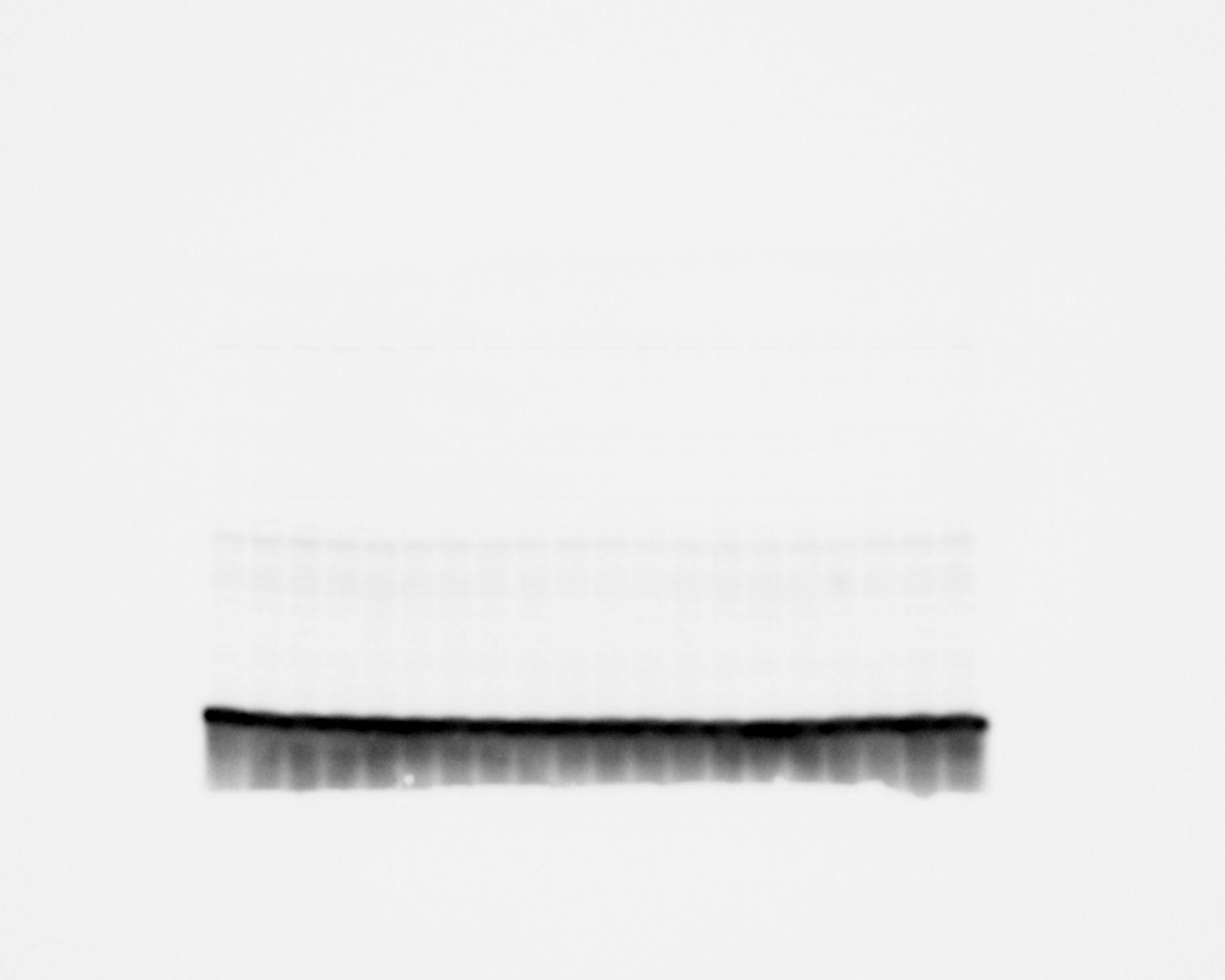

Supplement: Supplementary file 8 — Figure Source Data all EV figs [file 44318_2026_761_MOESM8_ESM.zip › EV Figures/EV5A/Western blot histone H3.tif]

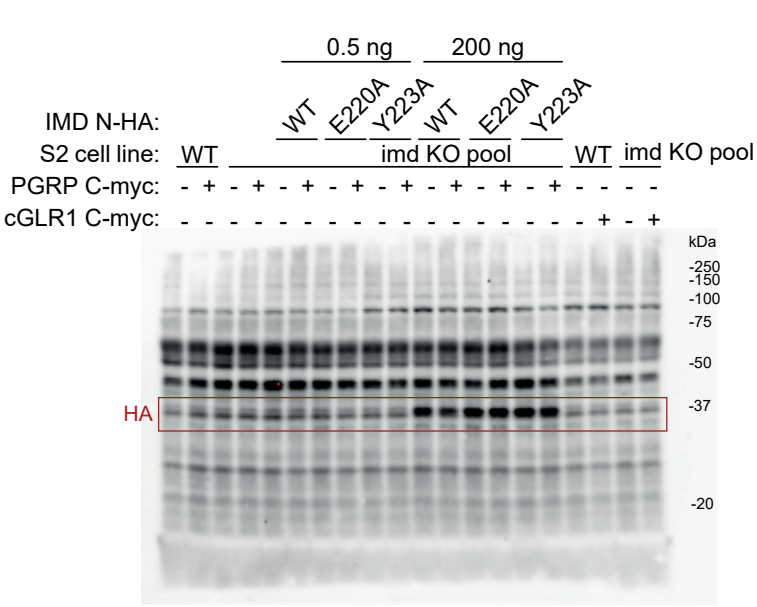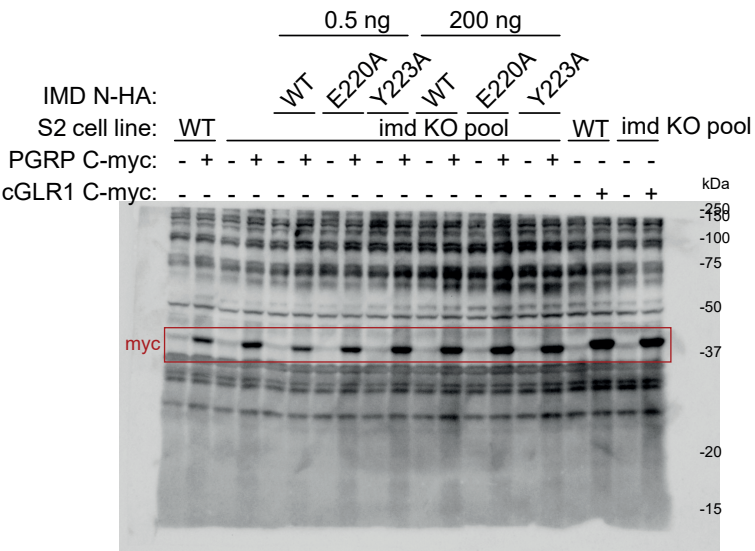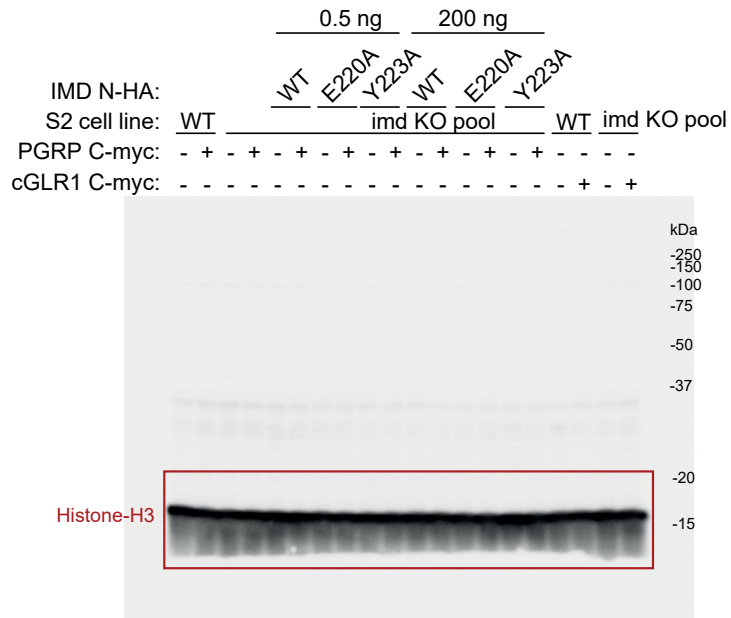

Supplement: Supplementary file 8 — Figure Source Data all EV figs [file 44318_2026_761_MOESM8_ESM.zip › EV Figures/EV5A/Annotation.pdf]

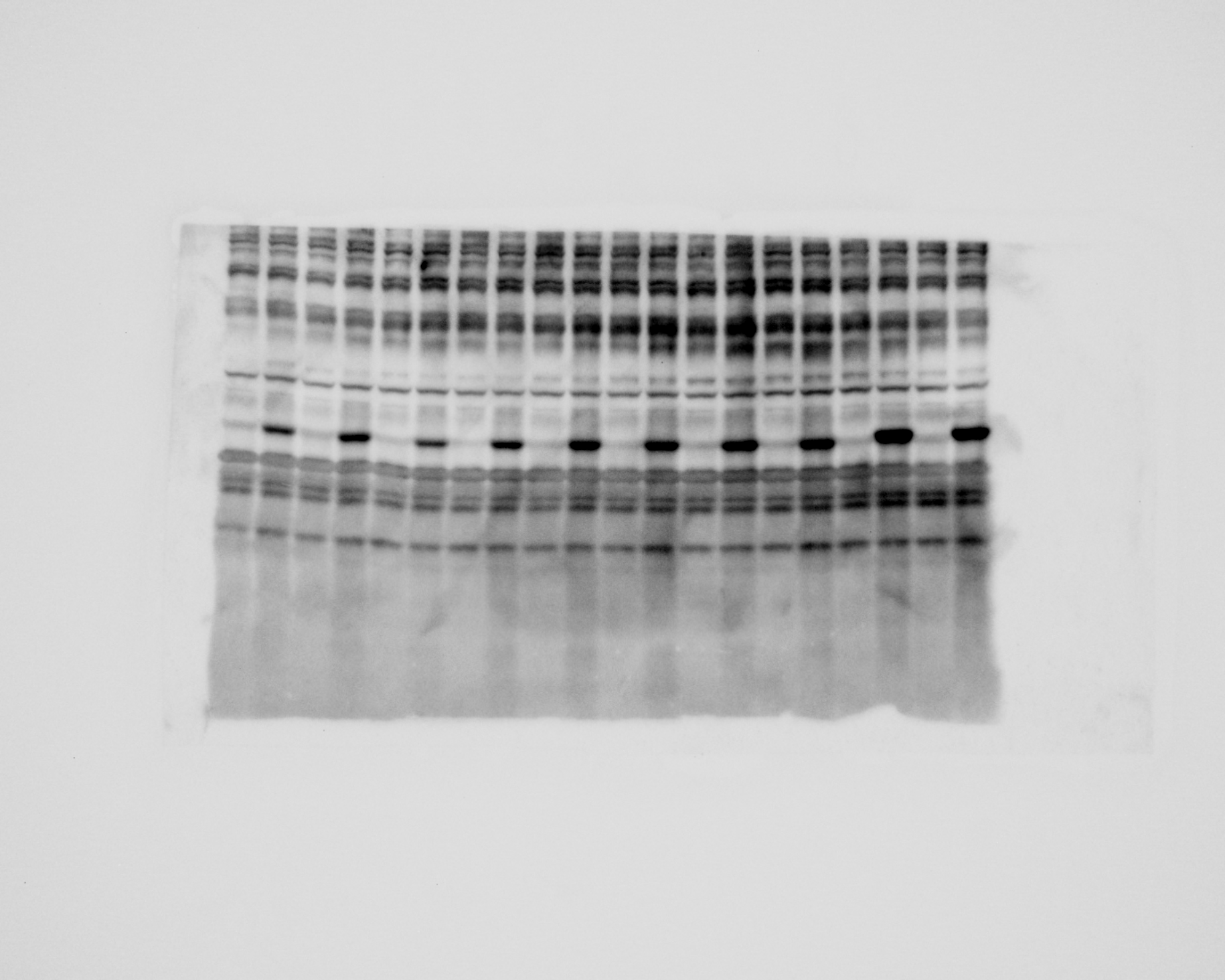

Supplement: Supplementary file 8 — Figure Source Data all EV figs [file 44318_2026_761_MOESM8_ESM.zip › EV Figures/EV5A/Western blot myc.tif]

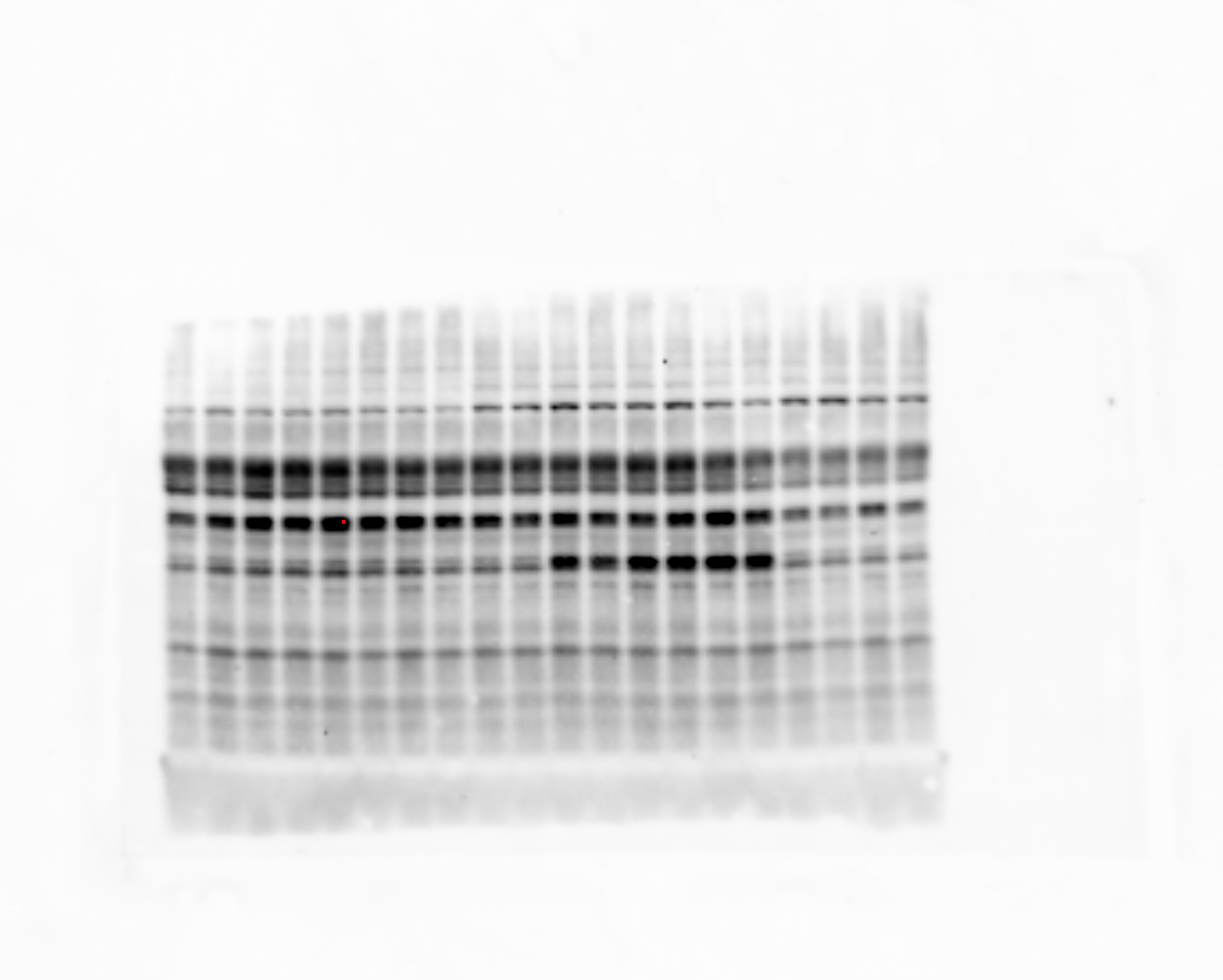

Supplement: Supplementary file 8 — Figure Source Data all EV figs [file 44318_2026_761_MOESM8_ESM.zip › EV Figures/EV5A/Western blot HA.tif]

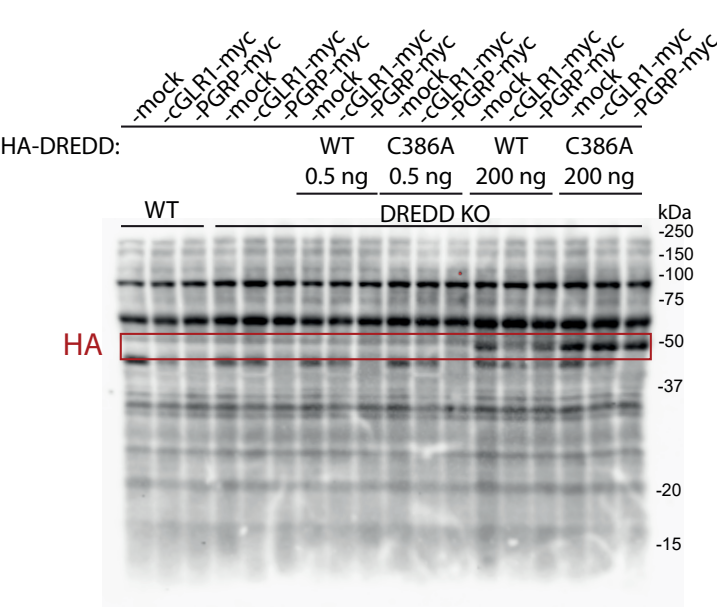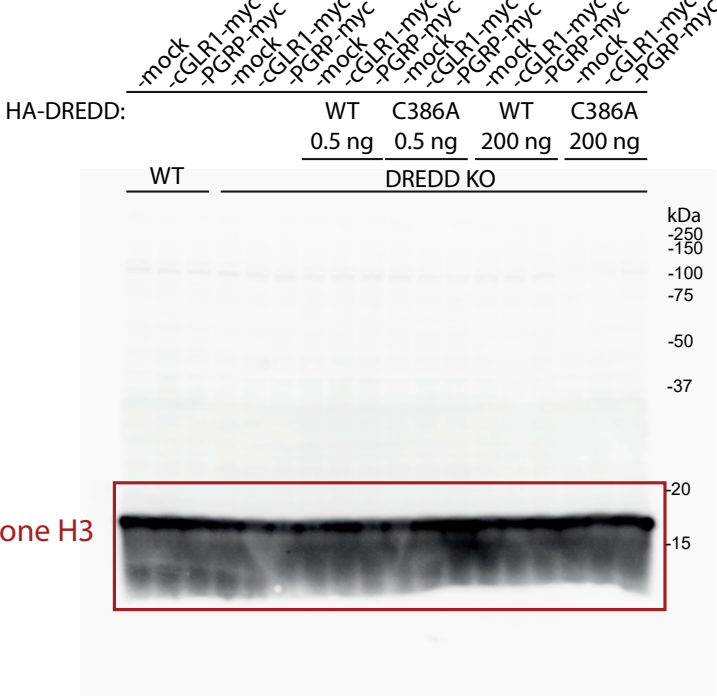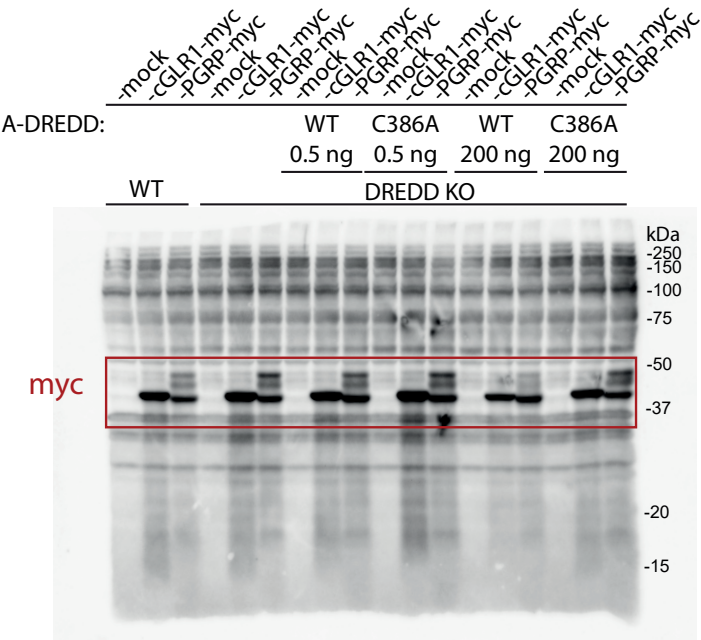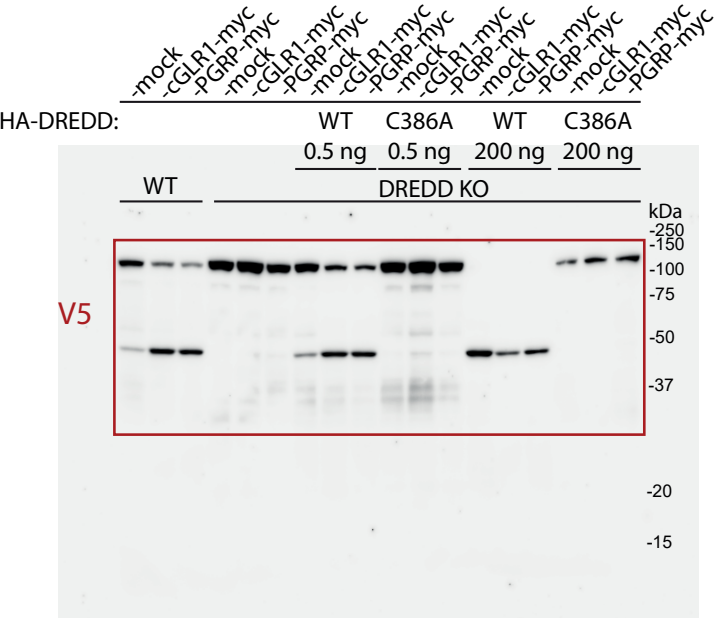

Supplement: Supplementary file 8 — Figure Source Data all EV figs [file 44318_2026_761_MOESM8_ESM.zip › EV Figures/EV3A/Annotation.pdf]

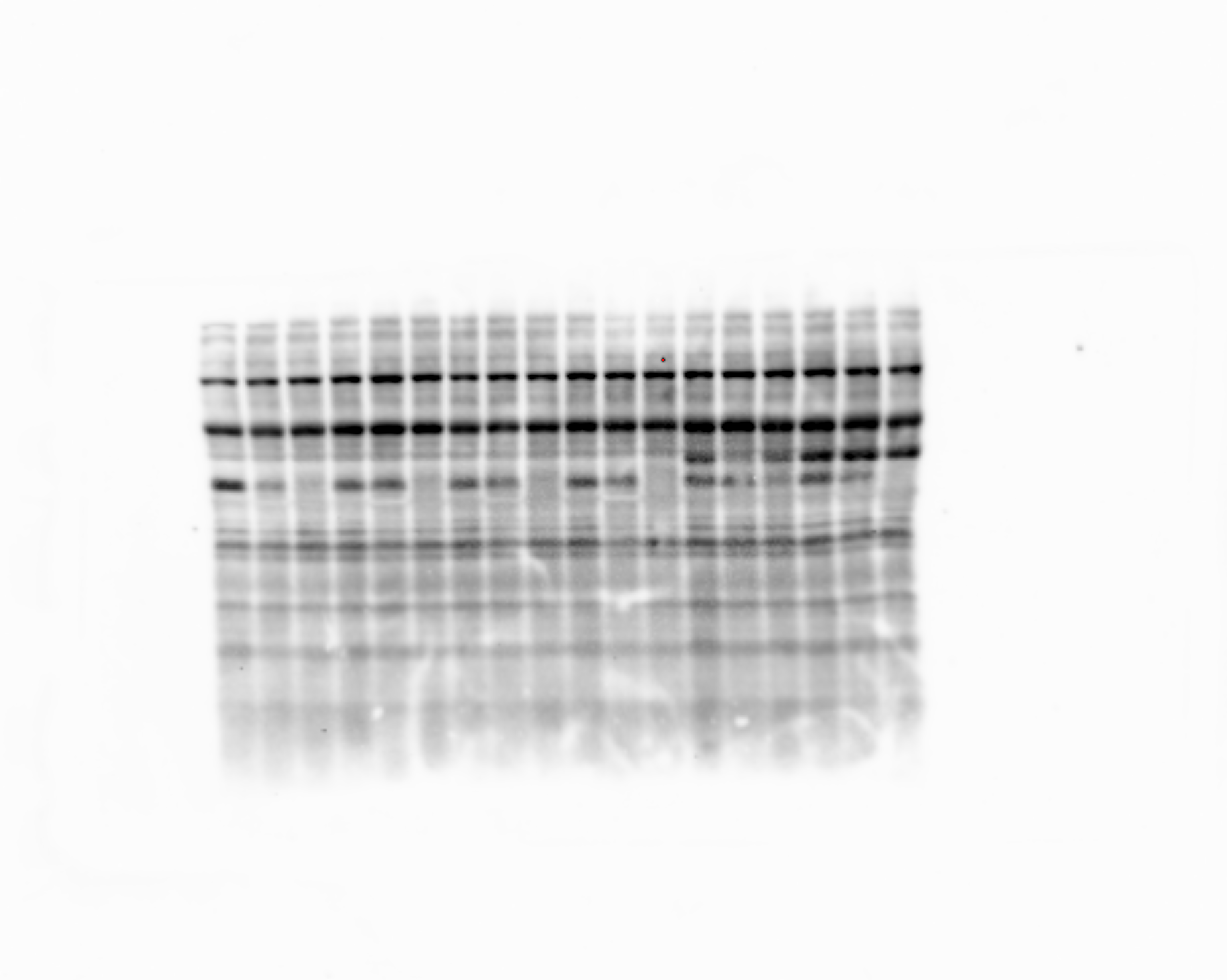

Supplement: Supplementary file 8 — Figure Source Data all EV figs [file 44318_2026_761_MOESM8_ESM.zip › EV Figures/EV3A/Western blot HA.tif]

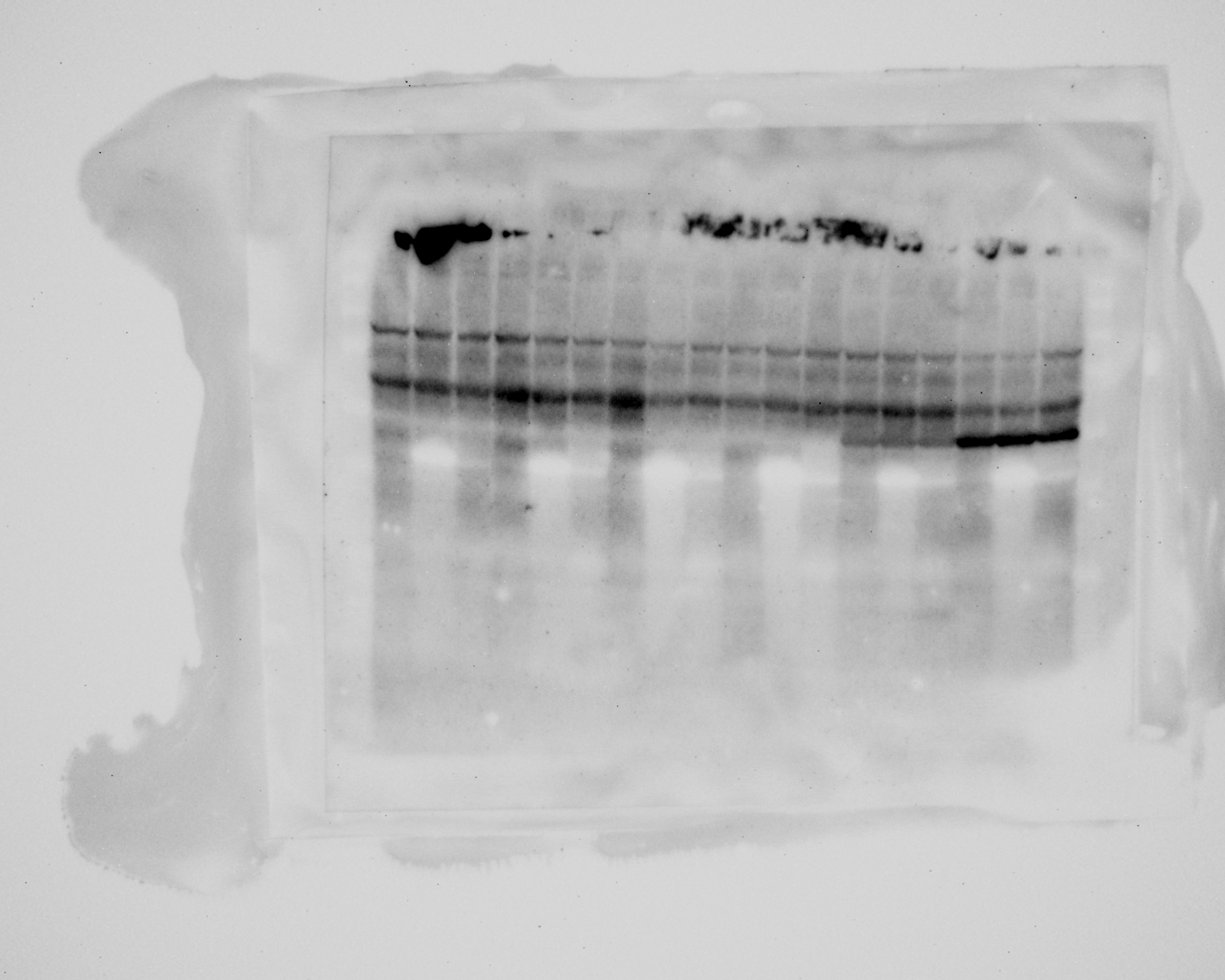

Supplement: Supplementary file 8 — Figure Source Data all EV figs [file 44318_2026_761_MOESM8_ESM.zip › EV Figures/EV3A/Repeat A/Western blot HA.tif]

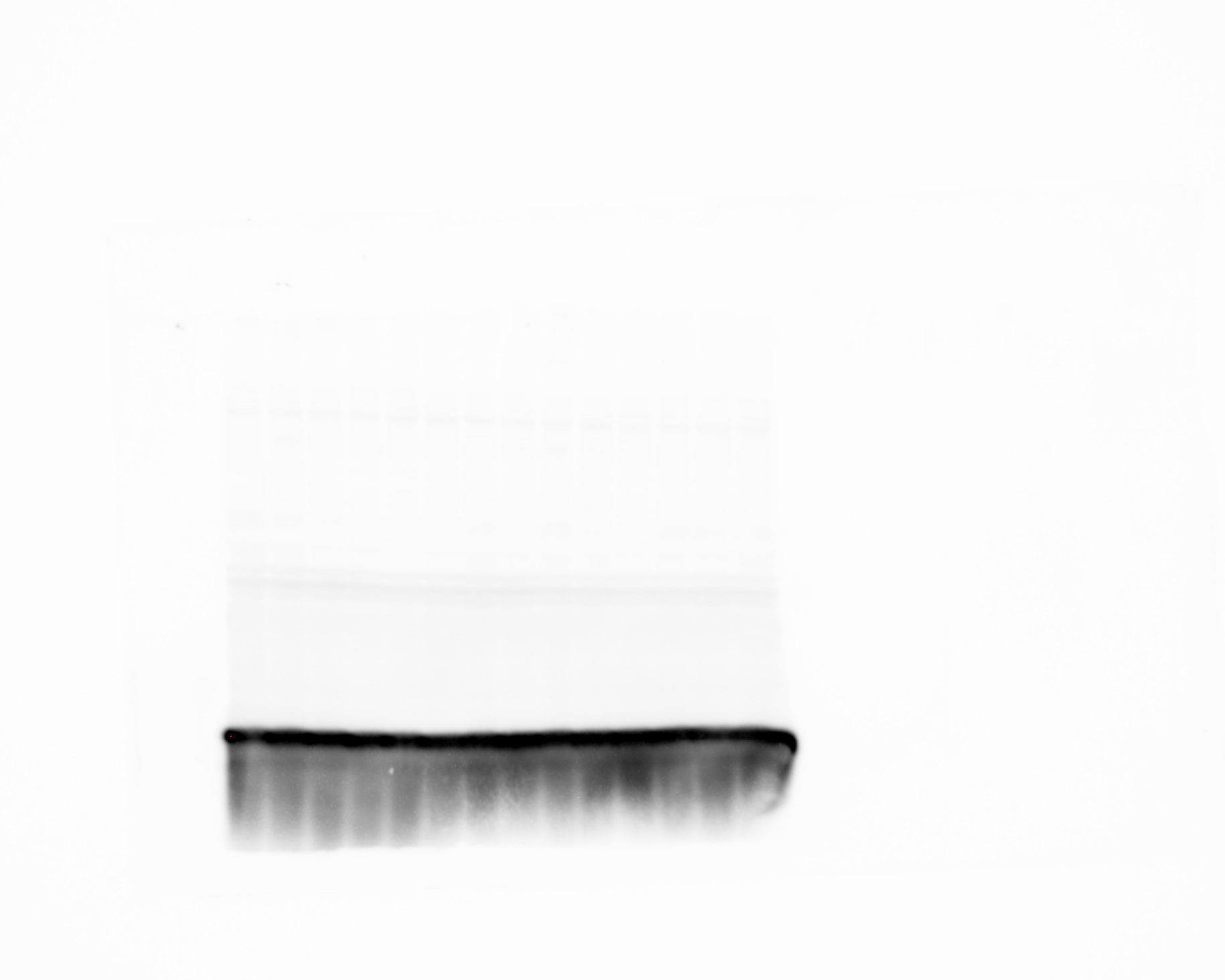

Supplement: Supplementary file 8 — Figure Source Data all EV figs [file 44318_2026_761_MOESM8_ESM.zip › EV Figures/EV4A/Western blot histone H3.tif]

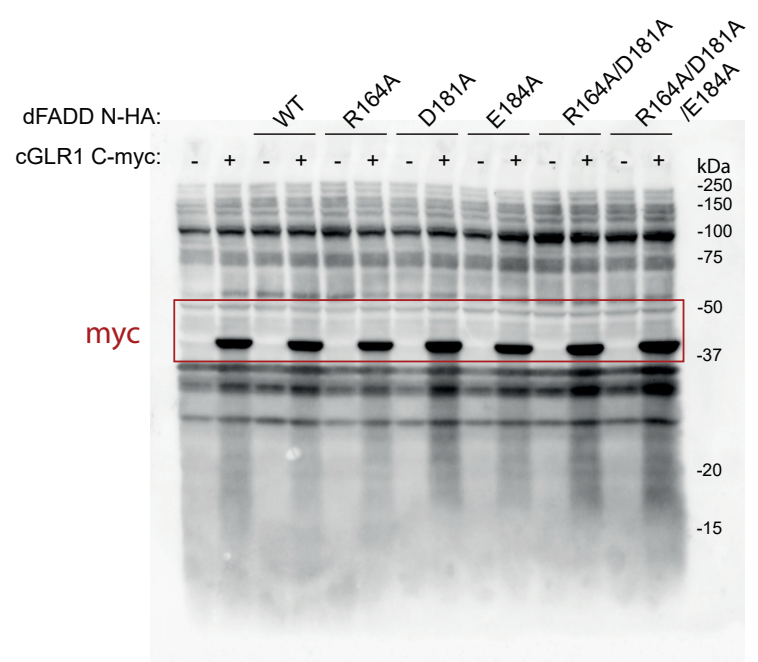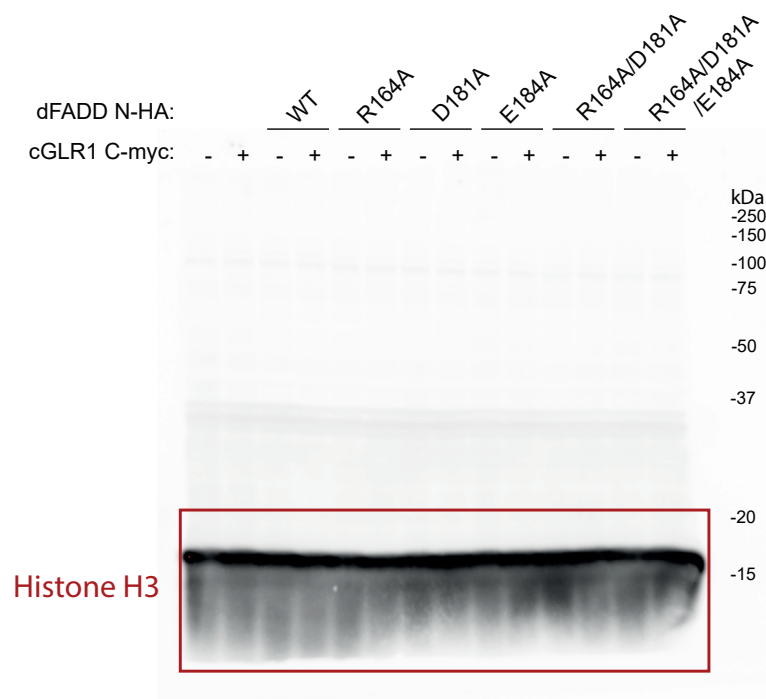

Supplement: Supplementary file 8 — Figure Source Data all EV figs [file 44318_2026_761_MOESM8_ESM.zip › EV Figures/EV4A/Annotation.pdf]

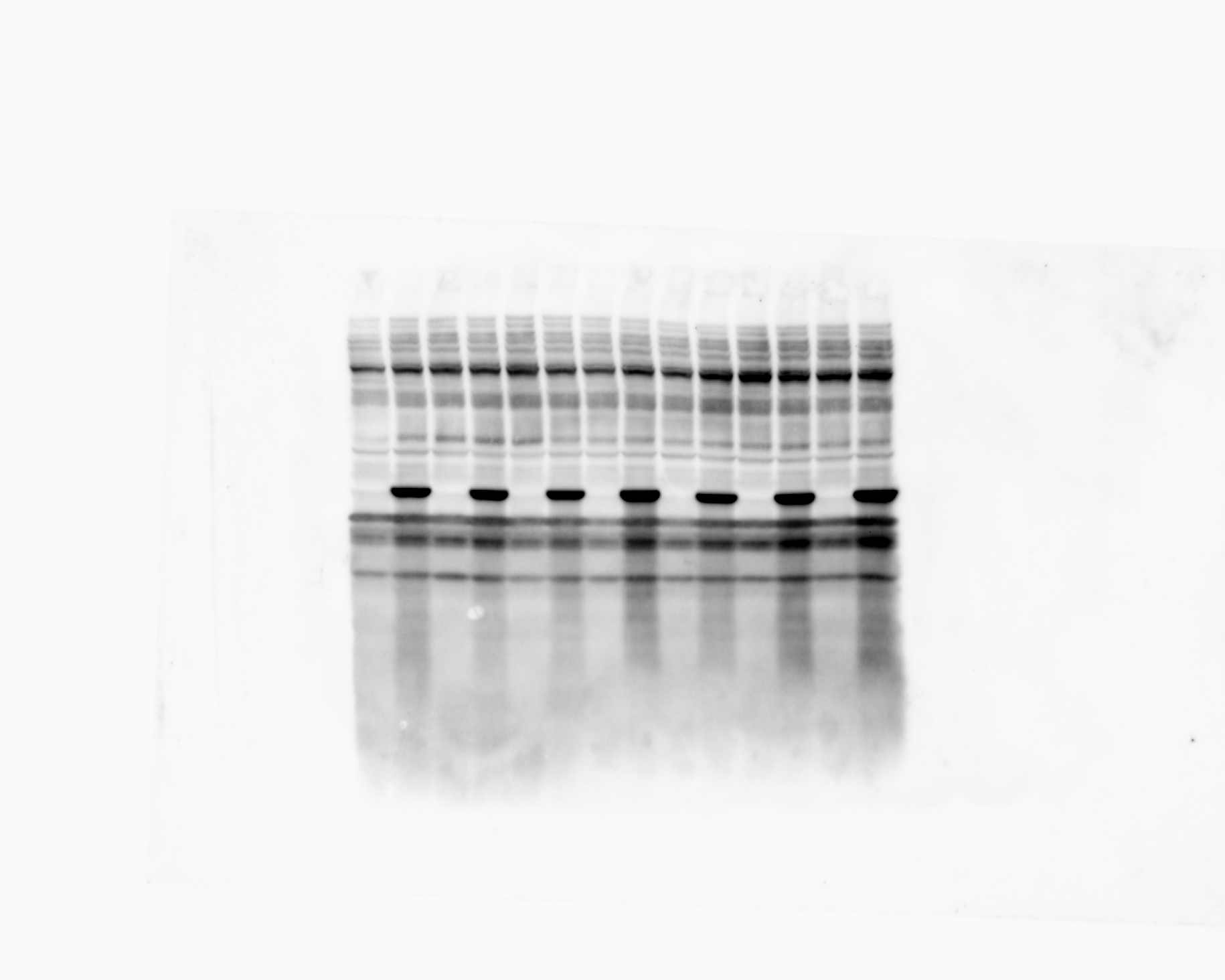

Supplement: Supplementary file 8 — Figure Source Data all EV figs [file 44318_2026_761_MOESM8_ESM.zip › EV Figures/EV4A/Western blot myc.tif]
