# Supplementary material for: FADD is recruited to activated STING oligomers to initiate caspase-mediated NF-κB activation in Drosophila melanogaster
Source: EMBO J. 2026 Mar 28;45(9):2965–90. doi: 10.1038/s44318-026-00761-9 (PMC13144350; doi:10.1038/s44318-026-00761-9)
Supplement: Supplementary file 5 — Source data Fig. 3 [file 44318_2026_761_MOESM5_ESM.zip › Figure 3/3E/Annotation.pdf]

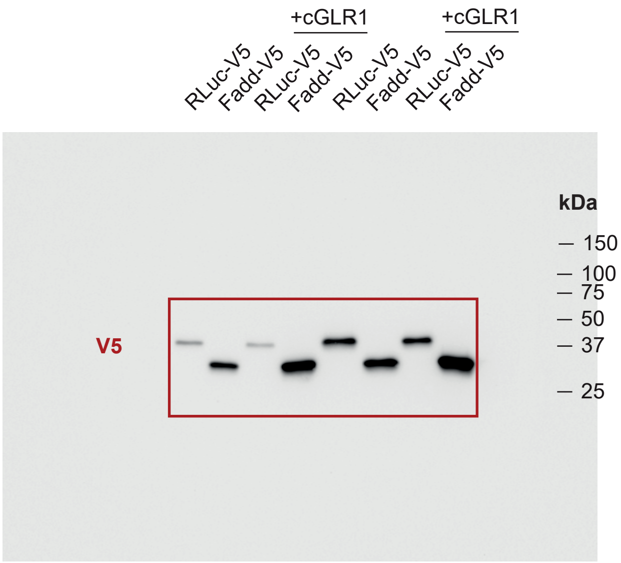
+cGLR1
+cGLR1
RLuc-V5
Fadd-V5
RLuc-V5
Fadd-V5
RLuc-V5
Fadd-V5
RLuc-V5
Fadd-V5
kDa
150
100
75
50
37
25
V5

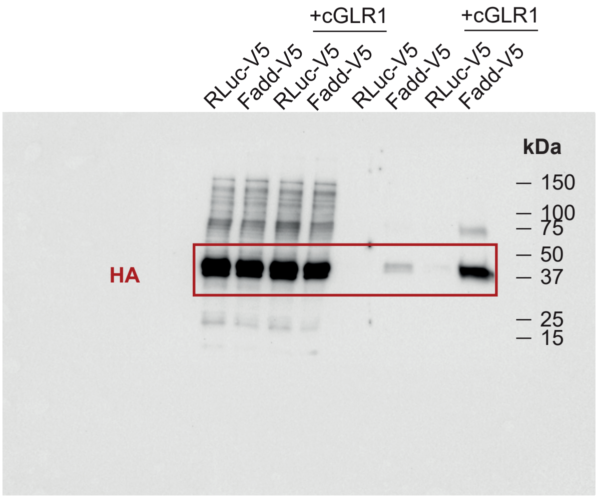
+cGLR1
+cGLR1
RLuc-V5
Fadd-V5
RLuc-V5
Fadd-V5
RLuc-V5
Fadd-V5
RLuc-V5
Fadd-V5
kDa
150
100
75
50
37
25
15
HA
