# Supplementary figures and images for: FADD is recruited to activated STING oligomers to initiate caspase-mediated NF-κB activation in Drosophila melanogaster (part 2 of 2)
Source: EMBO J. 2026 Mar 28;45(9):2965–90. doi: 10.1038/s44318-026-00761-9 (PMC13144350; doi:10.1038/s44318-026-00761-9)

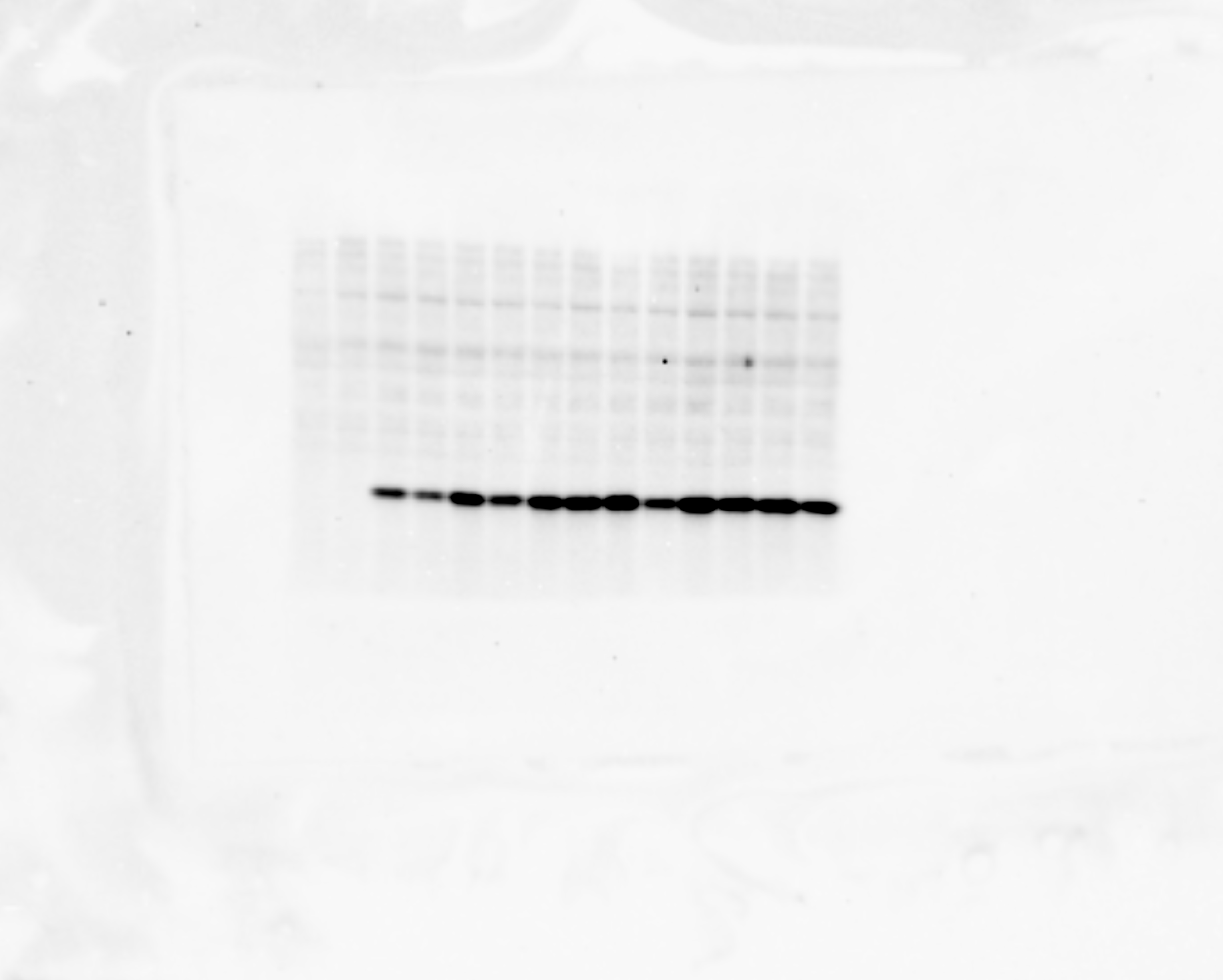

Supplement: Supplementary file 8 — Figure Source Data all EV figs [file 44318_2026_761_MOESM8_ESM.zip › EV Figures/EV4A/Western blot HA.tif]

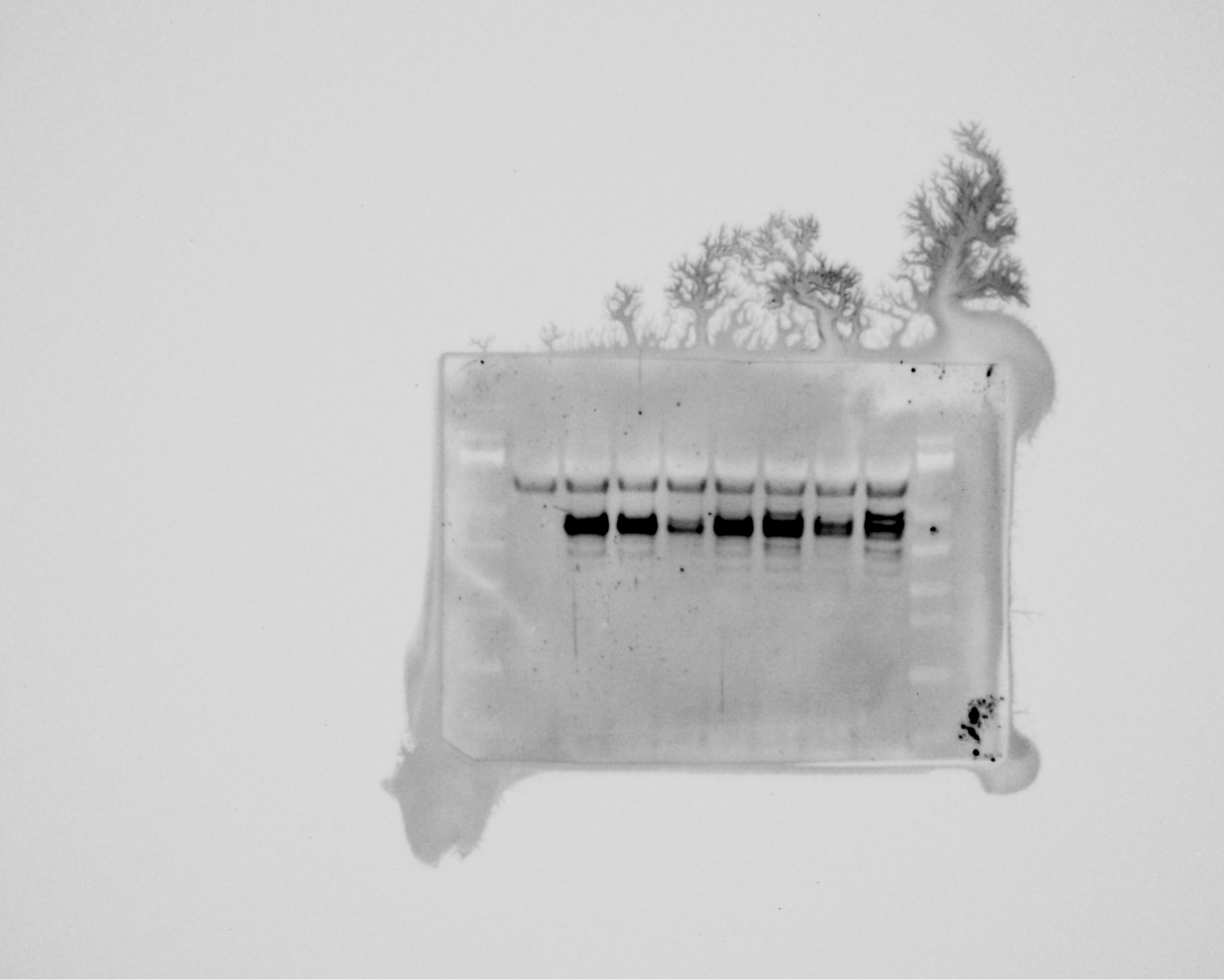

Supplement: Supplementary file 8 — Figure Source Data all EV figs [file 44318_2026_761_MOESM8_ESM.zip › EV Figures/EV4B/Western blot V5.tif]

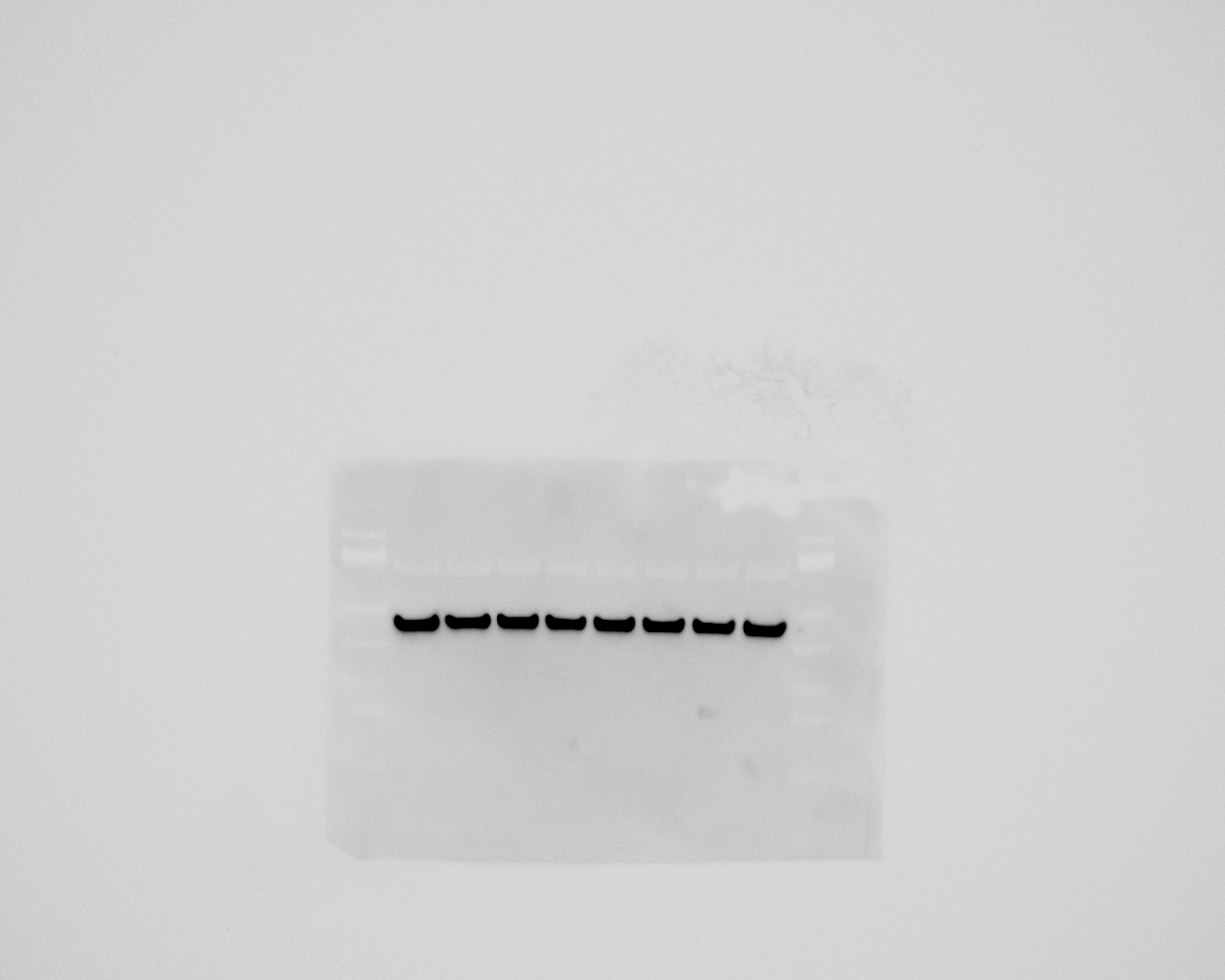

Supplement: Supplementary file 8 — Figure Source Data all EV figs [file 44318_2026_761_MOESM8_ESM.zip › EV Figures/EV4B/Western blot actin.tif]

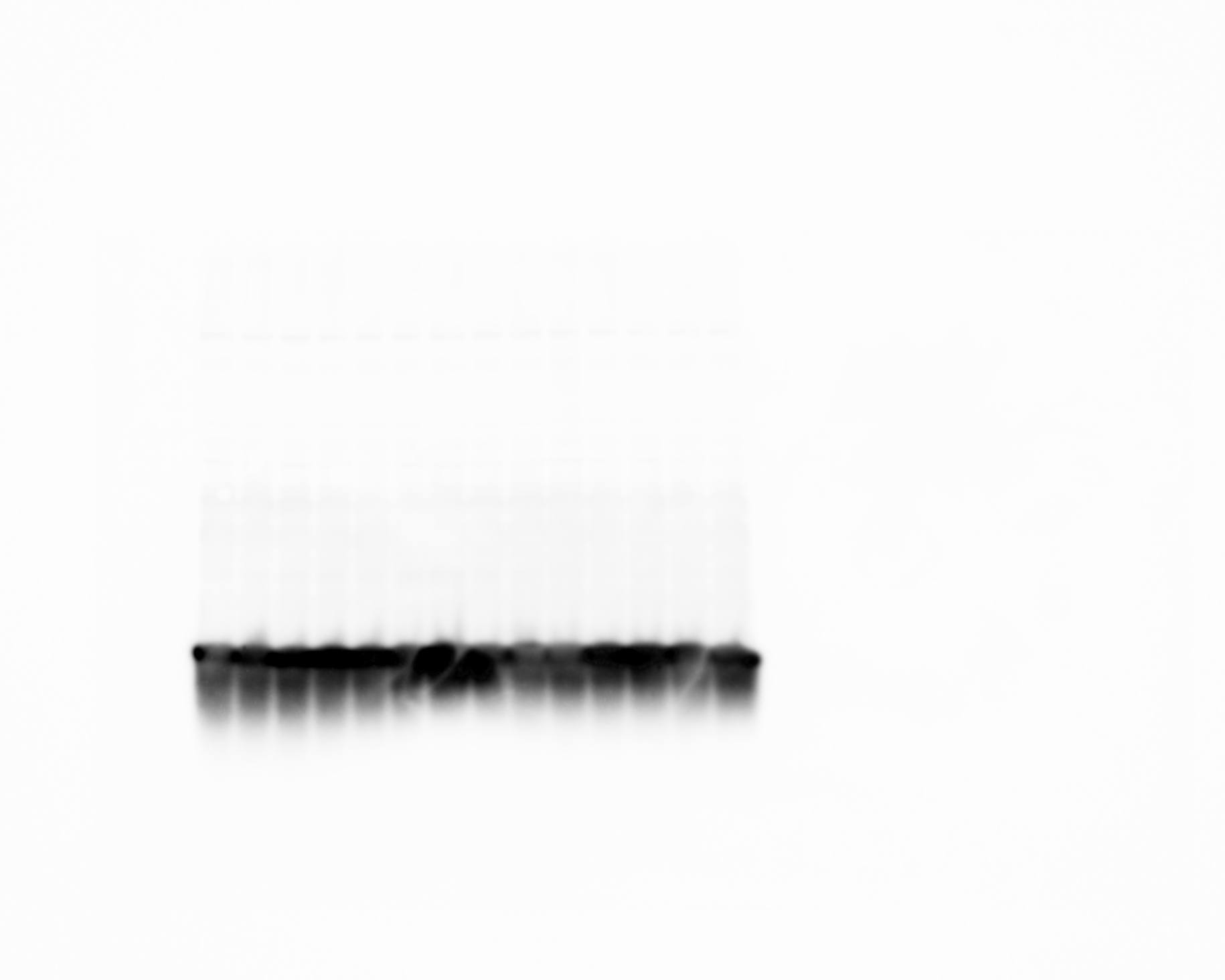

Supplement: Supplementary file 8 — Figure Source Data all EV figs [file 44318_2026_761_MOESM8_ESM.zip › EV Figures/EV4D/Western blot histone H3.tif]

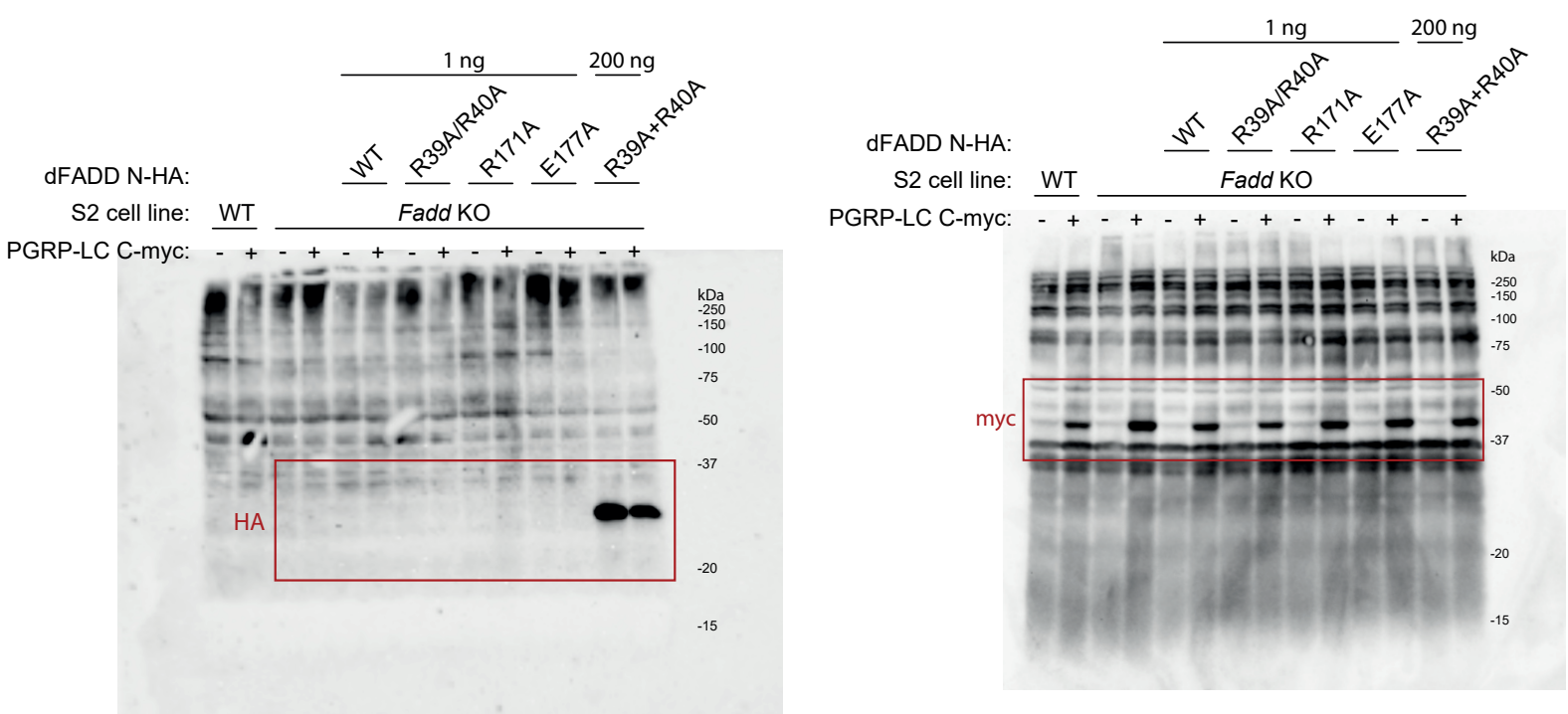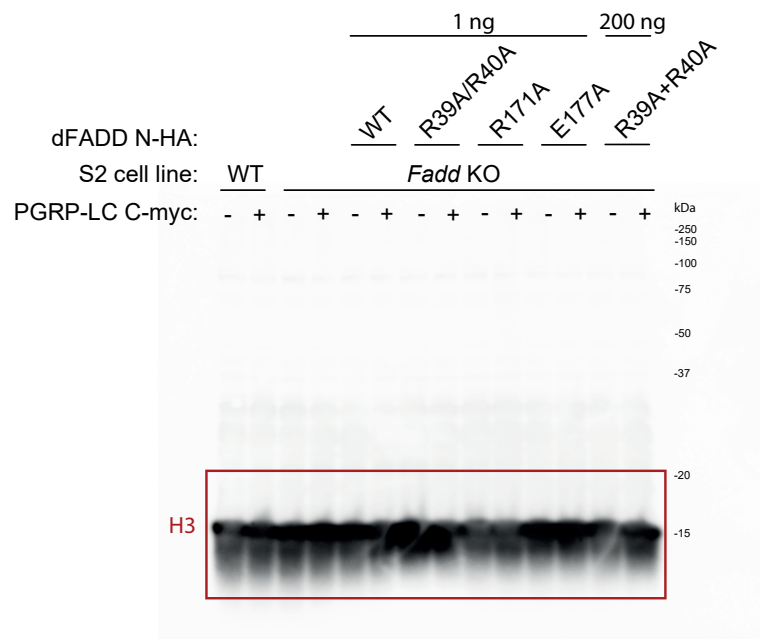

Supplement: Supplementary file 8 — Figure Source Data all EV figs [file 44318_2026_761_MOESM8_ESM.zip › EV Figures/EV4D/Annotation.pdf]

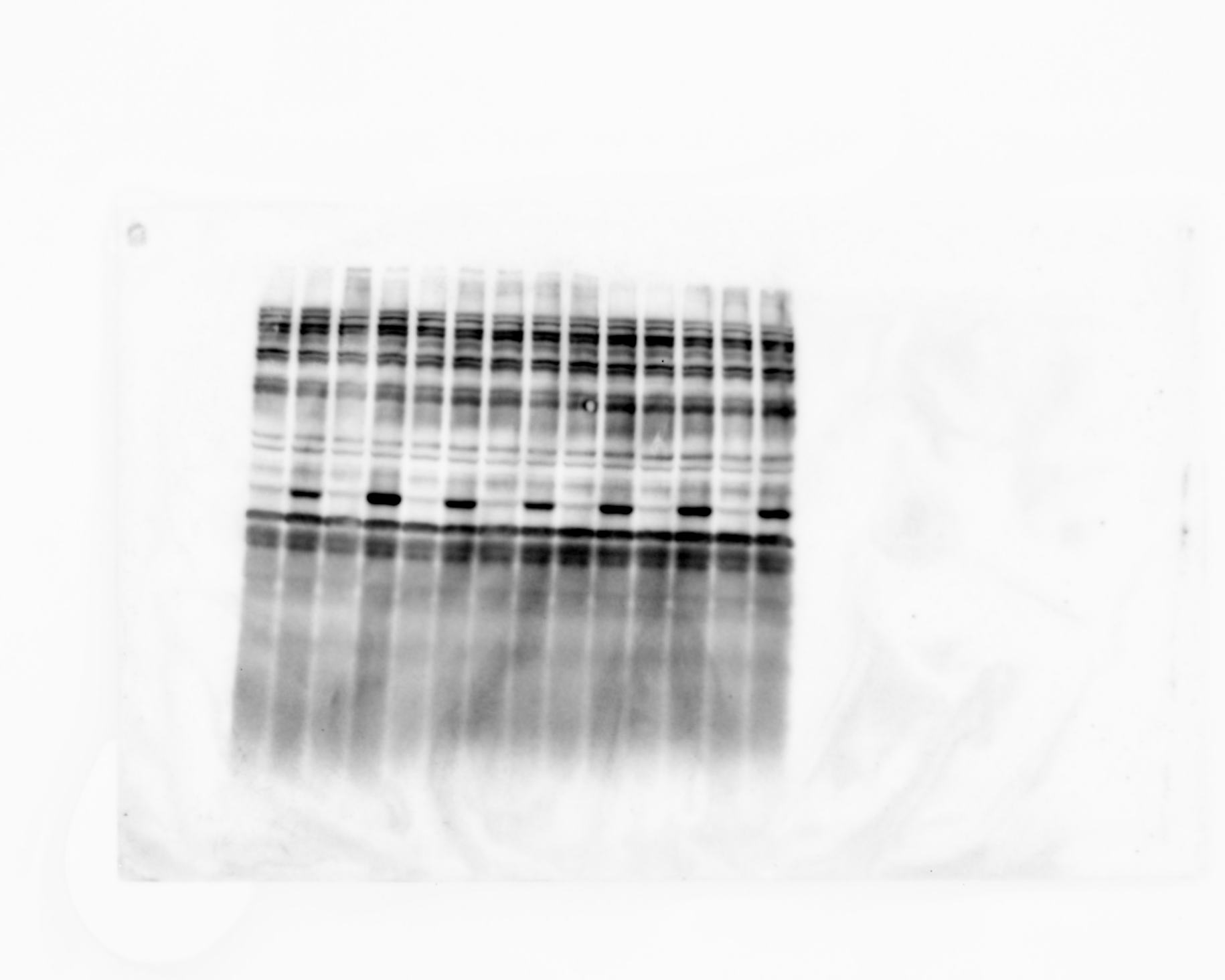

Supplement: Supplementary file 8 — Figure Source Data all EV figs [file 44318_2026_761_MOESM8_ESM.zip › EV Figures/EV4D/Western blot myc.tif]

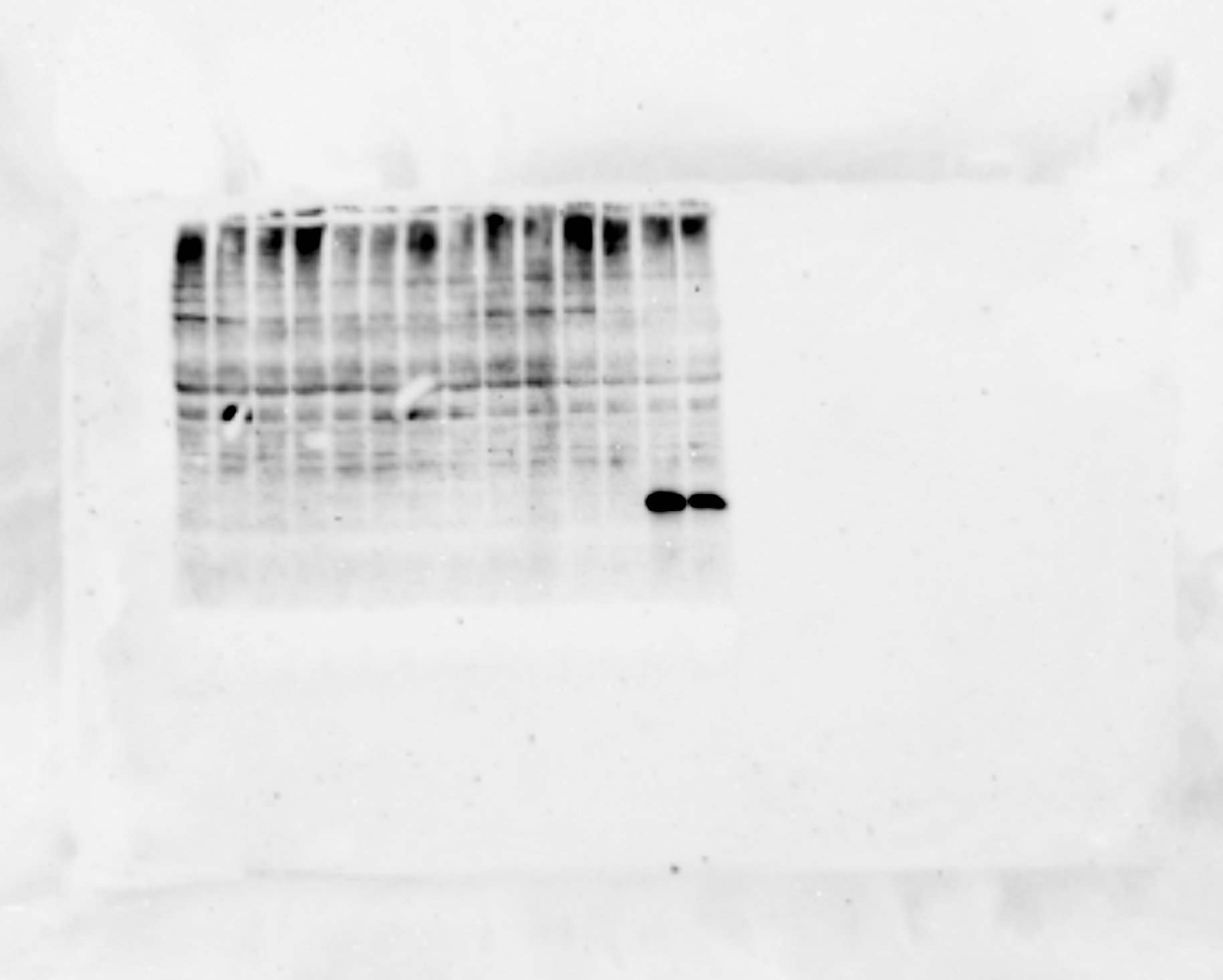

Supplement: Supplementary file 8 — Figure Source Data all EV figs [file 44318_2026_761_MOESM8_ESM.zip › EV Figures/EV4D/Western blot HA.tif]
