# Supplementary material for: FADD is recruited to activated STING oligomers to initiate caspase-mediated NF-κB activation in Drosophila melanogaster
Source: EMBO J. 2026 Mar 28;45(9):2965–90. doi: 10.1038/s44318-026-00761-9 (PMC13144350; doi:10.1038/s44318-026-00761-9)
Supplement: Supplementary file 9 — Expanded View Figures [file 44318_2026_761_MOESM9_ESM.pdf]

Expanded View Figures

Figure EV1. Signaling in S2 *Relish* KO pool and Srg-induction in *Relish^{D545A}* flies.

(**A**) Induction of *AttA* luciferase reporter upon expression of PGRP-LC in WT (gray) or *Relish* KO S2 cells (brown). Data from three independent experiments (different geometrical icons), each performed in biological triplicate, are shown with mean ($n = 9$). *P* values were calculated using two-way ANOVA, corrected with Tukey's post hoc test: ****$p < 0.0001$, ns: $p > 0.9999$ (**B**) Western blots of cell lysates from (**A**). Dotted lines indicate separate gels. (**C**) Induction of *Sting* luciferase reporter upon expression of cGLR1 in WT (gray) or *Relish* KO S2 cells (brown). Data from three independent experiments (different geometrical icons), each performed in biological triplicates, are shown with mean ($n = 9$). *P* values were calculated using two-way ANOVA, corrected with Tukey's post hoc test: ****$p < 0.0001$, ns: $p > 0.9999$. (**D**) western blots of cell lysates from (**C**). Dotted lines indicate separate gels. (**E–G**) Induction of *Srg1*, *Srg2*, or *Srg3* in w^{1118} (control) or *Relish^{D545A}* flies measured by qPCR 24 h after intrathoracic injection of the STING agonist 2′3′-cGAMP. Each data point is derived from a pool of six flies (three male, three female). Bars represent mean ± standard deviation. *P* values were calculated using a pairwise permutation test corrected with the Benjamini–Hochberg method: ***$p = 0.0008526$ (**F**) or 0.0006075 (**G**), **$p = 0.007433$, ns: $p = 0.8724$ (**E**), 0.7777 (**F**), or 0.1125 (**G**). (**H**) Survival of w^{1118} (control) or *Relish^{D545A}* flies injected with Drosophila C virus (DCV) and co-injected with Tris or 2′3′-cGAMP. Points and bars represent mean ± standard error. *P* values were calculated with a Gehan–Breslow–Wilcoxon test: ****$p < 0.0001$. Main Fig. 1C shows a sub-portion of data from this figure. Source data are available online for this figure.

© The Author(s)

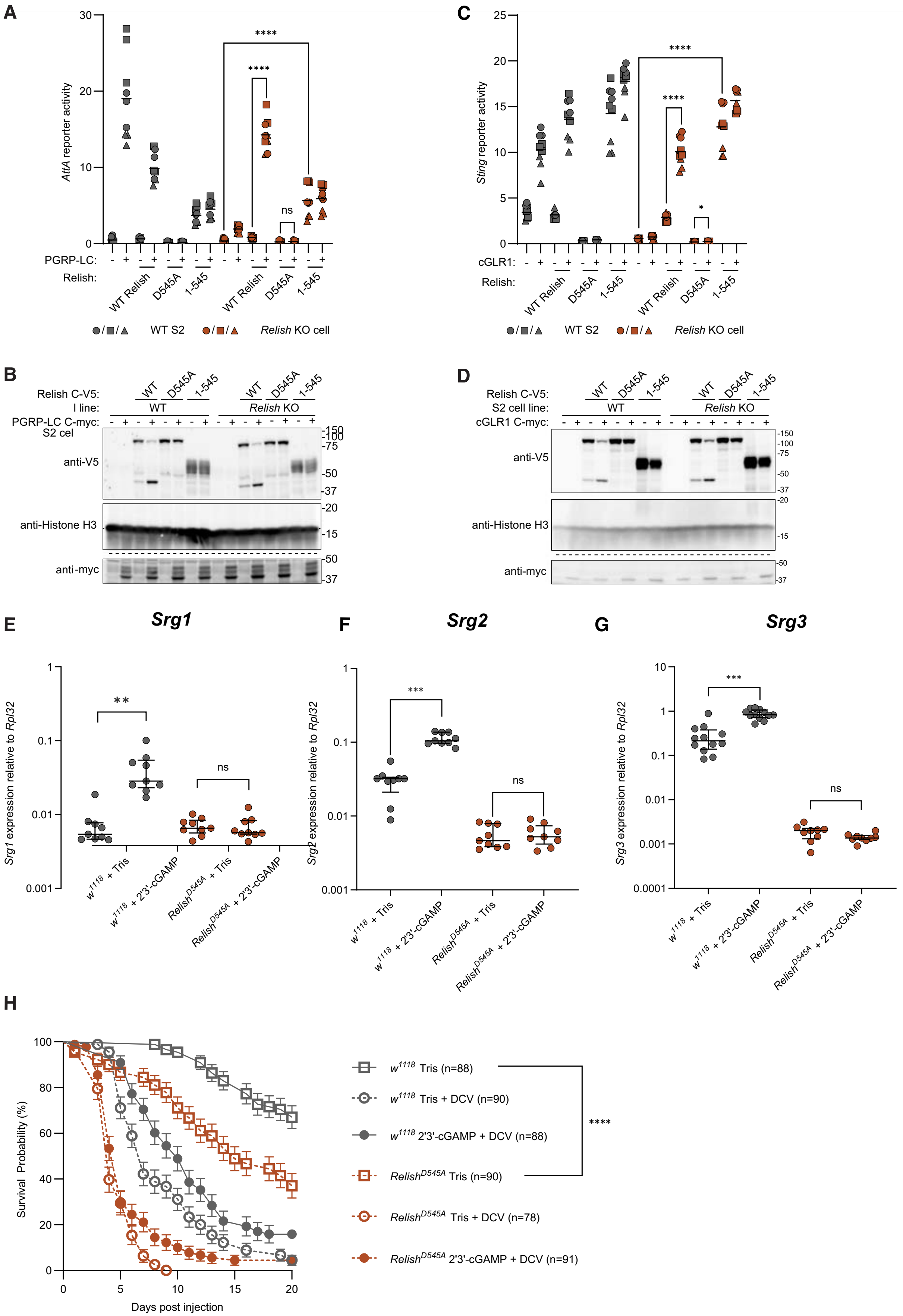
A
AttA reporter activity
PGRP-LC:
Relish:
WT Relish
D545A
1-545
ns
****
WT S2
Relish KO cell
B
Relish C-V5:
I line:
PGRP-LC C-myc:
S2 cel
WT
D545A
1-545
Relish KO
anti-V5
anti-Histone H3
anti-myc
C
Sting reporter activity
cGLR1:
Relish:
WT Relish
D545A
1-545
****
*
WT S2
Relish KO cell
D
Relish C-V5:
S2 cell line:
cGLR1 C-myc:
WT
Relish KO
anti-V5
anti-Histone H3
anti-myc
E
Srg1
Srg1 expression relative to Rpl32
**
ns
w1118 + Tris
w1118 + 2'3'-cGAMP
RelishD545A + Tris
RelishD545A + 2'3'-cGAMP
F
Srg2
Srg2 expression relative to Rpl32
***
ns
G
Srg3
Srg3 expression relative to Rpl32
***
ns
H
Survival Probability (%)
Days post injection
w1118 Tris (n=88)
w1118 Tris + DCV (n=90)
w1118 2'3'-cGAMP + DCV (n=88)
RelishD545A Tris (n=90)
RelishD545A Tris + DCV (n=78)
RelishD545A 2'3'-cGAMP + DCV (n=91)
****

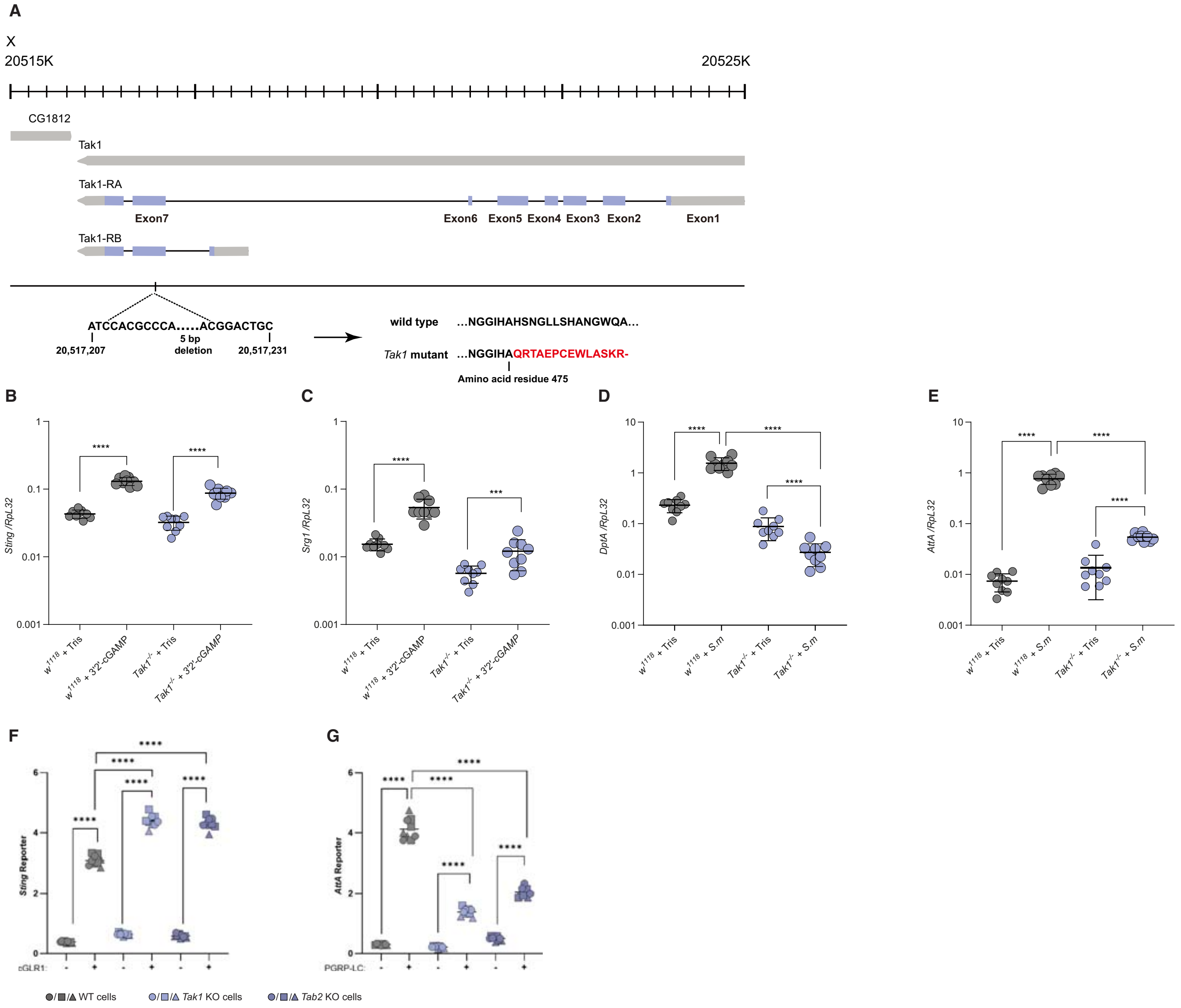

Figure EV2. The role of dTAK1 and dTAB2 in dSTING and IMD signaling.

(**A**) Generation of *Tak1* knock-out flies. The *Tak1* gene, located on the right arm of the X chromosome, is shown together with its annotated transcripts, Tak1-RA and Tak1-RB, encoding the long or short isoform of dTak1, respectively. Open reading frames are indicated in light purple. A 5 bp deletion creates a frameshift after the alanine residue at position 475 of the long isoform, leading to termination of translation after insertion of a 14 amino acid insertion (Gln-Arg-Thr-Ala-Glu-Pro-Cys-Glu-Trp-Leu-Ala-Ser-Lys-Arg). (**B–E**) Induction of the dSTING-induced genes *Sting* and *Srg1* (**B**, **C**) or IMD-induced genes *DptA* or *AttA* (**D**, **E**) in w^{1118} (control) or $Tak1^{-/-}$ flies, measured by qPCR 24 h after intrathoracic injection of the STING agonist 3′2′-cGAMP or the IMD activator *Serratia marcescens* (*S.m*), respectively. Each data point is derived from a pool of six flies (three male, three female). Bars represent mean ± standard deviation. *P* values were calculated using one-way ANOVA with Tukey's multiple comparisons test: ****$p < 0.0001$, ***$p = 0.0001$. (**F**, **G**) Induction of the *Sting* or *AttA* reporter in WT (gray), *Tak1* (light purple) or *Tab2* (dark purple) KO S2 cells upon co-expression of cGLR1 or PGRP, respectively. Data from three independent experiments (different geometrical icons), each performed in biological triplicate, are shown with mean ($n = 9$). *P* values were calculated using two-way ANOVA, corrected with Tukey's post hoc test: ****$p < 0.0001$.

 © The Author(s)

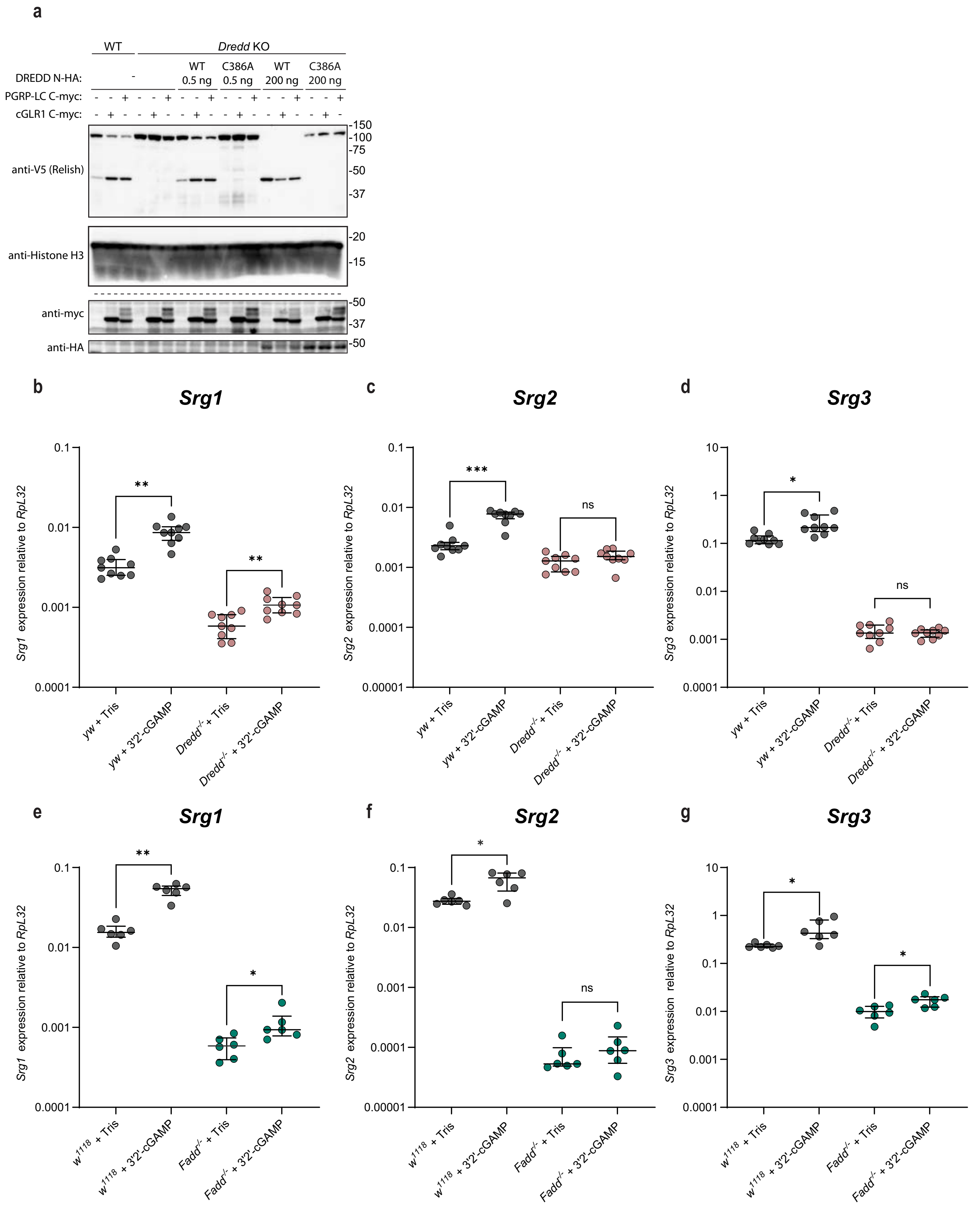
a
WT
Dredd KO
DREDD N-HA:
-
WT 0.5 ng
C386A 0.5 ng
WT 200 ng
C386A 200 ng
PGRP-LC C-myc:
cGLR1 C-myc:
anti-V5 (Relish)
anti-Histone H3
anti-myc
anti-HA
-150
-100
-75
-50
-37
-20
-15
-50
-37
-50
b
Srg1
Srg1 expression relative to RpL32
0.1
0.01
0.001
0.0001
**
**
yw + Tris
yw + 3'2'-cGAMP
Dredd-/- + Tris
Dredd-/- + 3'2'-cGAMP
c
Srg2
Srg2 expression relative to RpL32
0.1
0.01
0.001
0.0001
0.00001
***
ns
yw + Tris
yw + 3'2'-cGAMP
Dredd-/- + Tris
Dredd-/- + 3'2'-cGAMP
d
Srg3
Srg3 expression relative to RpL32
10
1
0.1
0.01
0.001
0.0001
*
ns
yw + Tris
yw + 3'2'-cGAMP
Dredd-/- + Tris
Dredd-/- + 3'2'-cGAMP
e
Srg1
Srg1 expression relative to RpL32
0.1
0.01
0.001
0.0001
**
*
w1118 + Tris
w1118 + 3'2'-cGAMP
Fadd-/- + Tris
Fadd-/- + 3'2'-cGAMP
f
Srg2
Srg2 expression relative to RpL32
0.1
0.01
0.001
0.0001
0.00001
*
ns
w1118 + Tris
w1118 + 3'2'-cGAMP
Fadd-/- + Tris
Fadd-/- + 3'2'-cGAMP
g
Srg3
Srg3 expression relative to RpL32
10
1
0.1
0.01
0.001
0.0001
*
*
w1118 + Tris
w1118 + 3'2'-cGAMP
Fadd-/- + Tris
Fadd-/- + 3'2'-cGAMP

© The Author(s)

Figure EV3. DREDD and dFADD are necessary for signaling.

(**A**) Cleavage of ectopically expressed Relish C-V5 in WT or *Dredd* KO S2 cells in response to expression of cGLR1 or PGRP-LC. *Dredd* KO cells were reconstituted with either WT DREDD or a catalytically inactive mutant (C386A) at either lower (0.5 ng plasmid) or higher (200 ng plasmid) expression levels. Main Fig. 2a shows a sub-portion of data from this figure. Lysates run on separate gels are indicated by dotted lines. (**B–G**), Induction of *Srg1, Srg2, or Srg3* in *yw* (control) and $yw^{Dredd-/-}$ flies (**B–D**) or w^{1118} (control) and $Fadd^{-/-}$ flies (**E–G**) measured by qPCR 24 h after intrathoracic injection of the STING agonist 3′2′-cGAMP. Each data point is derived from a pool of six flies (three male, three female). Bars represent mean ± standard deviation. *P* values were calculated using a pairwise permutation test corrected with the Benjamini–Hochberg method: ***$p = 0.000734$, **$p = 0.001001$ (**B**, yw), 0.00545 (**B**, $Dredd^{-/-}$), or 0.005422 (**E**), *$p = 0.01087$ (**D**), 0.04722 (**E**), 0.01735 (**F**), 0.03859 (**G**, w^{1118}), or 0.02056 (**G**, $Fadd^{-/-}$), ns: $p = 0.1291$ (**C**), 0.5239 (**D**), or 0.3662 (**F**). Source data are available online for this figure.

 © The Author(s)

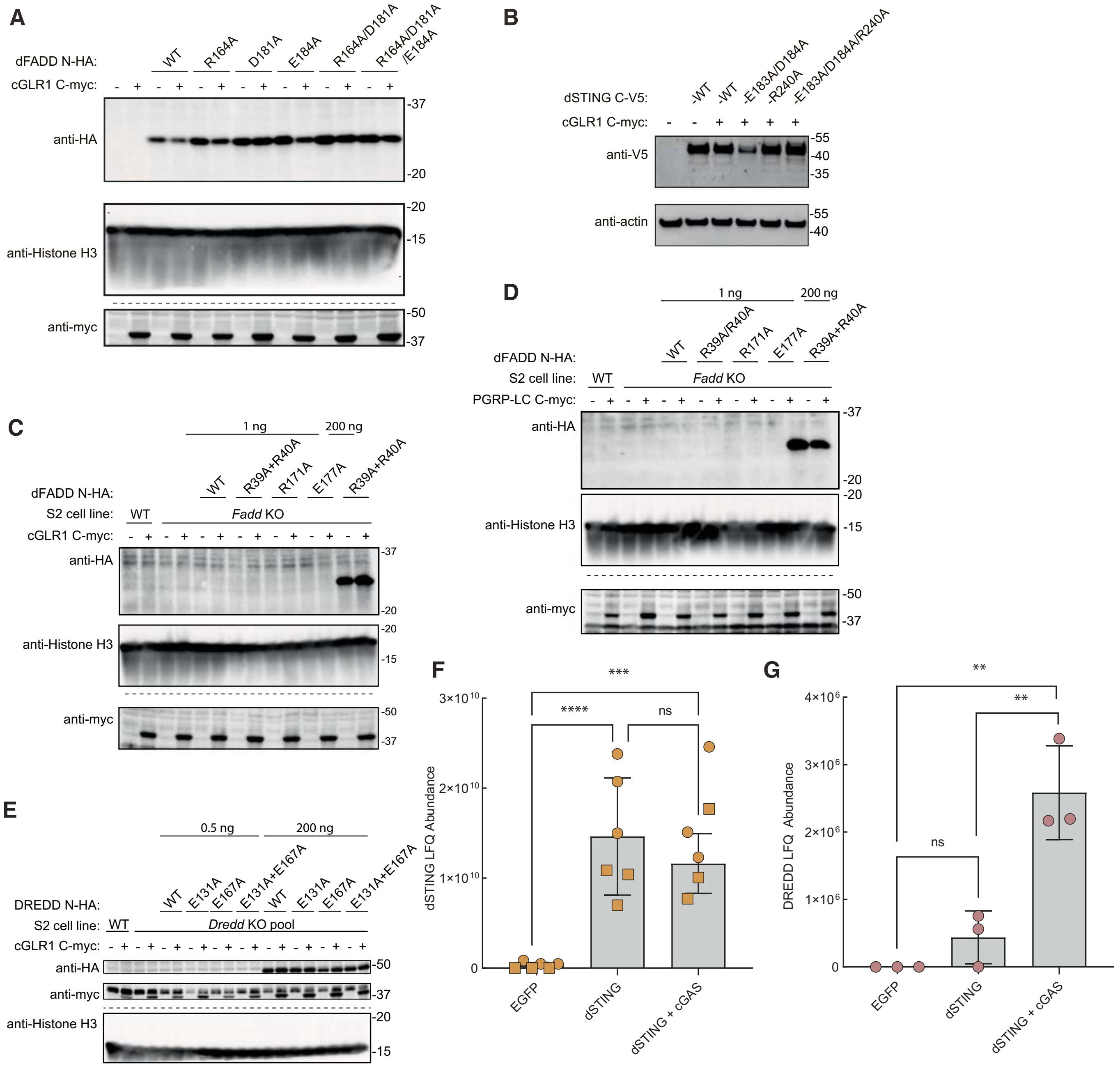

Figure EV4. Expression of dFADD, dSTING and DREDD mutants.

(**A**) Western blot showing expression of dFADD mutants at high expression levels (200 ng plasmid) to enable detection by immunoblot in contrast to expression levels rescuing signaling in *Fadd* KO cells (0.5 ng expression plasmid). Lysates run on separate gels are indicated by dotted lines. (**B**) Western blot showing expression of dSTING mutants at high expression levels (750 ng plasmid) to enable detection by immunoblot in contrast to expression levels rescuing signaling in *Sting* KO cells (10 ng plasmid). (**C–E**) Western blot showing expression of dFADD and DREDD mutants. Mutants were additionally expressed with 200 ng plasmid to verify protein expression. Lysates run on separate gels are indicated by dotted lines. (**F**, **G**) LC/MS -based detection of dSTING (**F**) or DREDD (**G**) peptides from co-immunoprecipitation of ectopically expressed EGFP (negative control) or V5-tagged dSTING in WT S2 cells with or without co-expression of cGAS. In (**F**), data from $n = 2$ independent experiments each containing three biological replicates are shown. In (**G**) data from $n = 1$ independent experiment containing three biological replicates are shown, since unique peptides from DREDD were not confidently detected in the other experiment. Bars indicate the mean. p values were calculated using one-way ANOVA corrected with Tukey's post hoc test: ****$p < 0.0001$, ***$p = 0.0009$, **$p = 0.0011$ (EGFP vs dSTING+cGAS) or 0.0031 (dSTING vs dSTING+cGAS), ns: $p = 0.4537$ (**F**) or 0.5118 (**G**). Source data are available online for this figure.

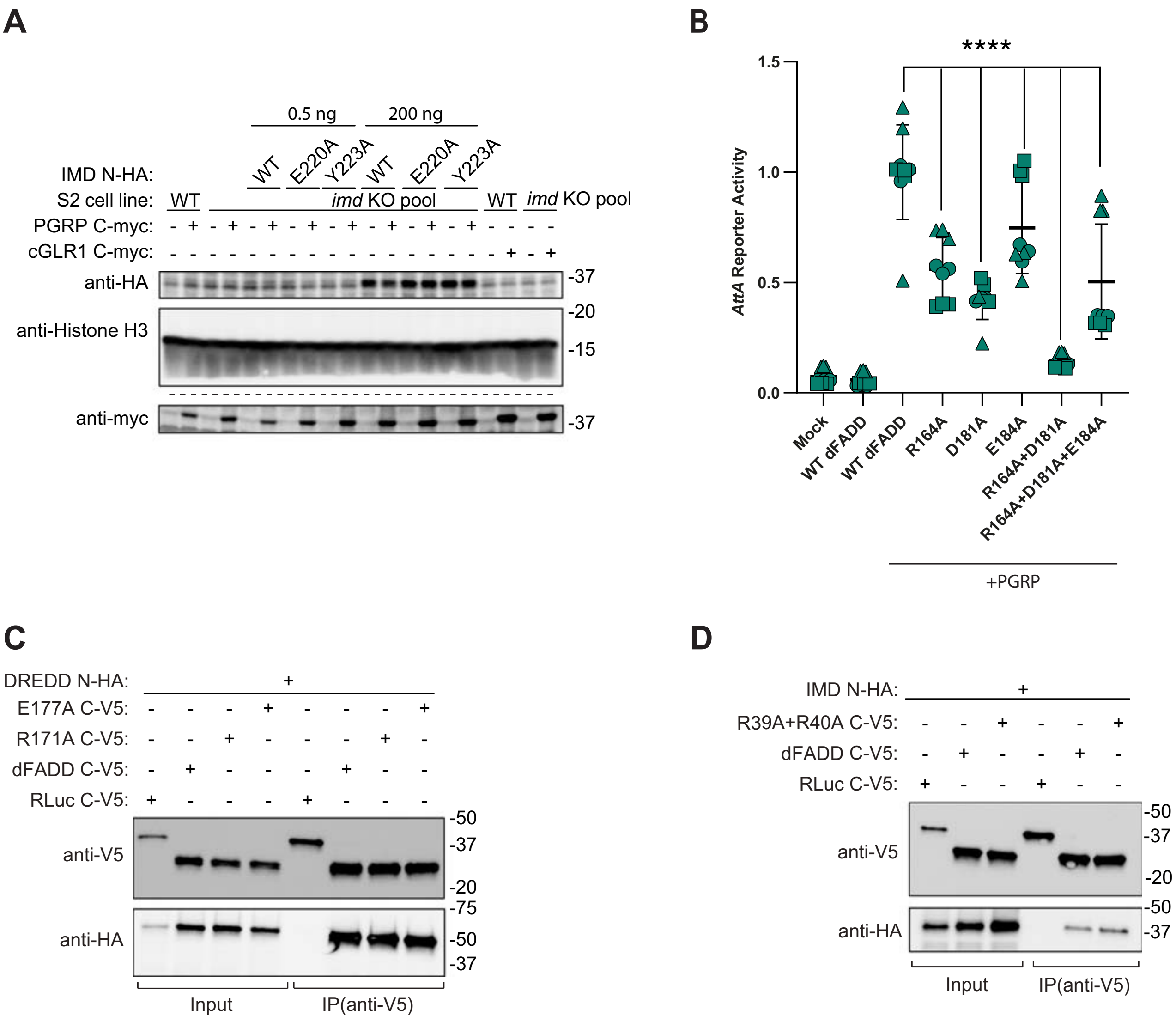

Figure EV5. Effect of mutations on IMD interactions and signaling.

(**A**) Western blot showing expression of IMD mutants. Mutants were additionally expressed with 200 ng plasmid to verify protein expression. Lysates run on separate gels are indicated by dotted lines ($n = 1$). (**B**) Induction of the *AttA* reporter in *Fadd* KO S2 cells upon co-expression of PGRP and reconstitution with WT dFADD or mutants disrupting the predicted dSTING:dFADD interface. Data from three independent experiments (different geometrical icons), each performed in biological triplicate ($n = 9$), are shown with mean and bars indicating standard deviation. For each experiment, all measurements were normalized to the mean of WT dFADD + PGRP. *P* values were calculated using two-way ANOVA, corrected with Dunnett's post hoc test: ****$p < 0.0001$. (**C**) DREDD N-HA and dFADD C-V5 or mutants disrupting the dFADD:IMD interface were expressed in S2 cells and immunoprecipitated on anti-V5 beads ($n = 2$). (**D**) IMD N-HA and dFADD C-V5 or the R39A/R40A mutant were expressed in S2 cells and immunoprecipitated on anti-V5 beads ($n = 2$). Source data are available online for this figure.

 © The Author(s)
