# Supplementary material for: FADD is recruited to activated STING oligomers to initiate caspase-mediated NF-κB activation in Drosophila melanogaster
Source: EMBO J. 2026 Mar 28;45(9):2965–90. doi: 10.1038/s44318-026-00761-9 (PMC13144350; doi:10.1038/s44318-026-00761-9)
Supplement: Supplementary file 8 — Figure Source Data all EV figs [file Annotation.pdf › EV Figures/EV4B/Annotation.pdf]

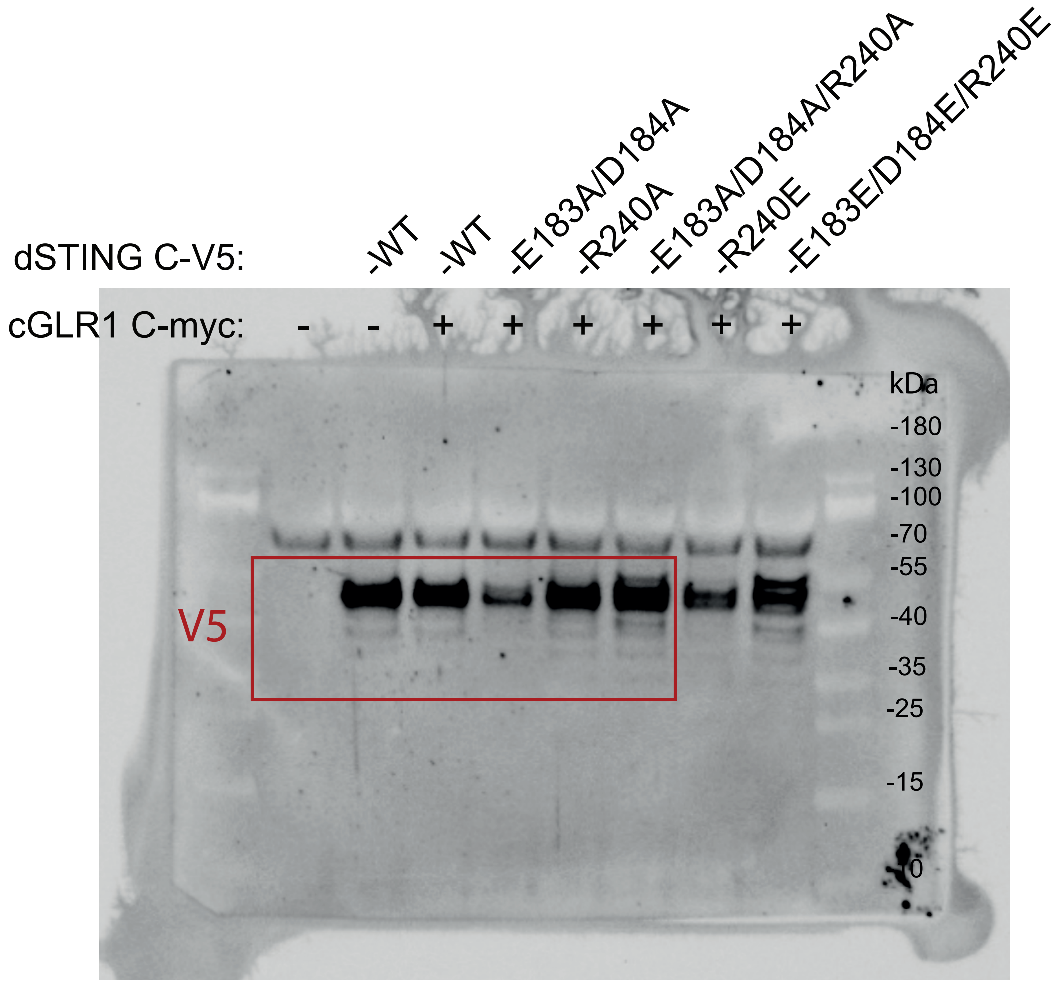
dSTING C-V5:
-WT
-WT
-E183A/D184A
-R240A
-E183A/D184A/R240A
-R240E
-E183E/D184E/R240E
cGLR1 C-myc:
- - + + + + + +
kDa
-180
-130
-100
-70
-55
-40
-35
-25
-15
-10
V5

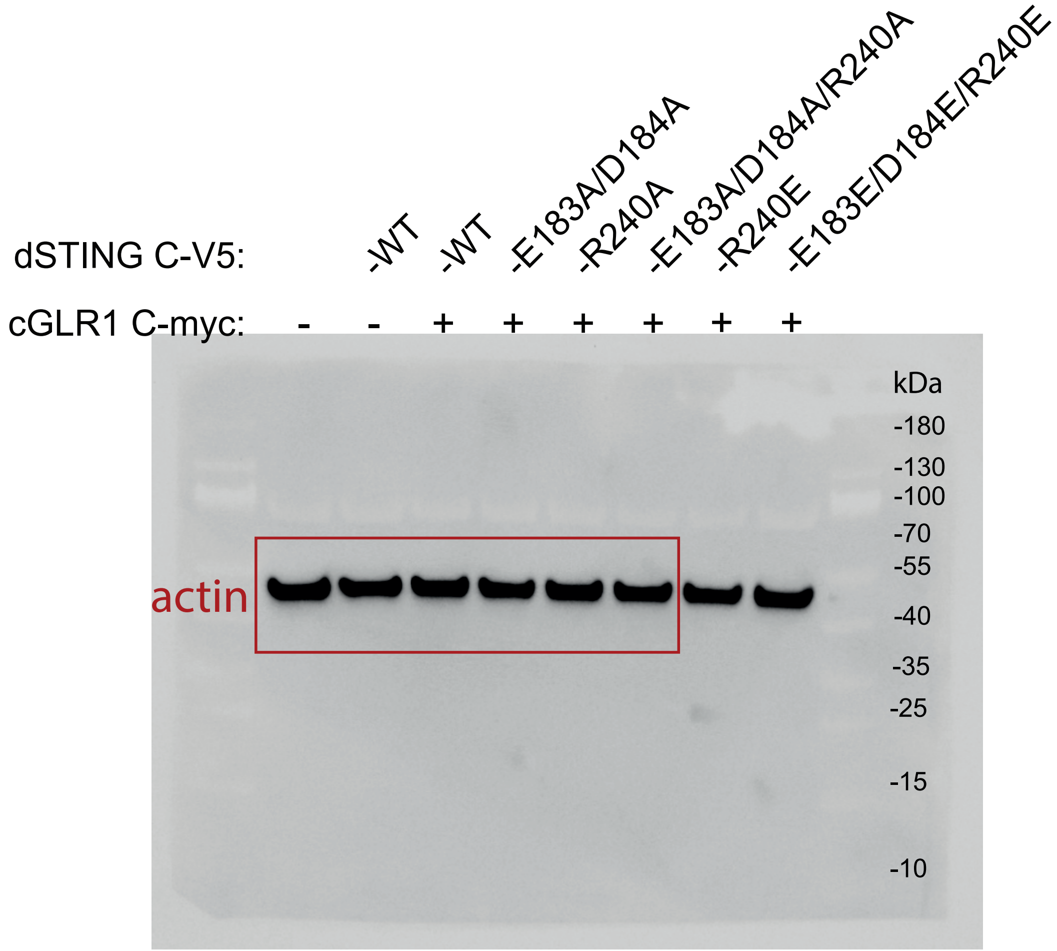
dSTING C-V5:
-WT
-WT
-E183A/D184A
-R240A
-E183A/D184A/R240A
-R240E
-E183E/D184E/R240E
cGLR1 C-myc:
- - + + + + + +
kDa
-180
-130
-100
-70
-55
-40
-35
-25
-15
-10
actin
